# Identification of chemosensory genes from the antennal transcriptome of Indian meal moth *Plodia interpunctella*

**DOI:** 10.1371/journal.pone.0189889

**Published:** 2018-01-05

**Authors:** Xiaojian Jia, Xiaofang Zhang, Hongmin Liu, Rongyan Wang, Tao Zhang

**Affiliations:** 1 Institute of Plant Protection, Hebei Academy of Agriculture and Forestry Sciences/Integrated Pest Management Center of Hebei Province/Key Laboratory of IPM on Crops in Northern Region of North China, Ministry of Agriculture, Baoding, P. R. China; 2 College of Agronomy, Xinyang Agriculture and Forestry University, Xinyang, P. R. China; Chinese Academy of Agricultural Sciences Institute of Plant Protection, CHINA

## Abstract

Olfaction plays an indispensable role in mediating insect behavior, such as locating host plants, mating partners, and avoidance of toxins and predators. Olfactory-related proteins are required for olfactory perception of insects. However, very few olfactory-related genes have been reported in *Plodia interpunctella* up to now. In the present study, we sequenced the antennae transcriptome of *P*. *interpunctella* using the next-generation sequencing technology, and identified 117 candidate olfactory-related genes, including 29 odorant-binding proteins (OBPs), 15 chemosensory proteins (CSPs), three sensory neuron membrane proteins (SNMPs), 47 odorant receptors (ORs), 14 ionotropic receptors (IRs) and nine gustatory receptors (GRs). Further analysis of qRT-PCR revealed that nine OBPs, three CSPs, two SNMPs, nine ORs and two GRs were specifically expressed in the male antennae, whereas eight OBPs, six CSPs, one SNMP, 16 ORs, two GRs and seven IRs significantly expressed in the female antennae. Taken together, our results provided useful information for further functional studies on insect genes related to recognition of pheromone and odorant, which might be meaningful targets for pest management.

## Introduction

Indian meal moth, *Plodia interpunctella* (Hübener) (Lepidoptera: Pyraloidea, Pyralidae), is a notorious stored-product pest worldwide [1]. The larvae infest a variety of processed foods, including fruits, nuts, cereals, powdered milk, chocolate, birdseed, and pet food [2], causing extensive damage by impairing dry weight, germination, nutritional value, and quality grade. It is difficult to control *P*. *interpunctella* by conventional insecticides, because it often inhabits our kitchen, closet and warehouse, and its larvae are mixed with our processed foods. Accordingly, several novel strategies have been developed to monitor and control *P*. *interpunctella*. Among these novel methods, sex pheromone is widely acceptable due to its safety and efficiency. Meanwhile, host volatiles have been thought to affect the oviposition behavior of *P*. *interpunctella* [3]. However, the underlying molecular mechanisms of olfactory recognition of *P*. *interpunctella* remain largely unexplored.

An accurate olfactory system plays crucial roles in survival, reproduction, and chemical communication for most insects [4]. Using the olfactory system in antennae, when peripheral odorants are detected, insects will activate olfactory sensory neurons (ORNs) and translate the signals into nerve impulses to the brain [5]. At least six gene families are involved in the olfactory sensory procedure, including three sensory protein families: odorant-binding proteins (OBPs), chemosensory proteins (CSPs), and sensory neuron membrane proteins (SNMPs); and three major chemosensory receptor families: odorant receptors (ORs), ionotropic receptors (IRs) and gustatory receptors (GRs). Additionally, odorant degrading enzymes (ODEs) are also classified in olfactory system, due to their integral roles in the rapid inactivation of semiochemicals [6–7, 4].

Sensory proteins, functioning as molecular actors, are considered to play crucial roles in detection of semiochemicals. They participate in the initial transduction of olfactory signals. When the odorants are detected, binding proteins (OBPs, CSPs and SNMPs) will specifically bind the hydrophobic odorants, and transport them to cross the aqueous sensillum lymph that embeds olfactory neuron dendrites. Subsequently, the odorants interact with membrane-bound chemosensory receptors (ORs IRs, and GRs) in the receptor neuron membrane, in which the odorant signals are transformed into electric signals. Finally, signal termination is inactivated by ODEs, which prevent the continuous accumulation of stimulants and subsequent sensory adaptation, and allow insects to rapidly respond to changes in environmental odorants [8–9].

During the past decade, the emergence of next generation sequencing (NGS) technology has dramatically improved the efficiency of gene screening. Meanwhile, the entomological research has also benefited from the development of NGS technology [10]. With the improvement of high-throughput sequencing methods, olfactory-related genes have been identified from antennal transcriptomes in numerous Lepidoptera species, including several notorious agricultural pests [11–22]. Such technology has been widely used to identify genes involved in olfaction of insects. However, little information is available about the function of olfactory-ralated genes of *P*. *interpunctella* due to the deficiency of the genomic data for this species.

Although several transcriptomic studies related to *P*. *interpunctella* have been performed [23–25], antennal transcriptome analysis of olfactory system has not been conducted in previous studies. To identify the olfactory-related genes, we described the antennal transcriptome analysis of *P*. *interpunctella* in the present study. The expression levels of olfactory-related genes were investigated using quantitative real-time PCR. Taken together, our study successfully identified olfactory-related genes of *P*. *interpunctella* and provided useful information for further studies on pheromone and host volatile recognition.

## Materials and methods

### Insects material and RNA extraction

*Plodia interpunctella* was the laboratorial population which was reared for more than 20 generations in our laboratory. The larvae were reared on crushed grains of wheat under constant conditions (28±1°C, 60±5% RH and 14:10 L:D photoperiod). Mature larvae were sorted by sex according to the black spot in the middle of male back. Antennae were excised from 3-day-old unmated moths, immediately frozen in liquid nitrogen and ground with a pestle. Total RNA was extracted from 100 antennae for each sex. The evaluation of RNA purity, RNA concentration and RNA quality were conducted following our previous method [13].

### cDNA library preparation for transcriptome sequencing

cDNA library were constructed following previous method [17]. Briefly, 3 μg RNA per sample was used as input material for the RNA sample preparation. Sequencing libraries were generated using NEBNext®Ultra™ RNA Library Prep Kit for Illumina® (NEB, USA) following manufacturer’s instructions. Newly isolated mRNA was further purified using with Oligo (dT) magnetic beads and sheared into 200–700 nucleotides sections using fragmentation buffer. The fragmented mRNA was used as templates for first-strand cDNA synthesis using random hexamer primers. Subsequently, second-strand cDNA was synthesized using DNA polymerase I and RNaseH. All remaining overhangs were passivated via polymerase. After adenylation of 3′ ends of DNA fragments, NEBNext Adaptor with hairpin loop structure was ligated for hybridization. In order to select cDNA fragments of preferentially 150~200 bp, the library fragments were purified using an AMPure XP system. Then 3 μL USER Enzyme (NEB, USA) was incubated with size-selected, adaptor-ligated cDNA at 37°C for 15 min followed by incubation at 95°C for 5 min before PCR reaction. Subsequently, PCR was performed with Phusion High-Fidelity DNA polymerase, Universal PCR primers and Index (X) Primer. Amplicons were purified (AMPure XP system) and library quality was assessed on the Agilent Bioanalyzer 2100 system. The cDNA library of *P*. *interpunctella* was sequenced on Illumina Hiseq™ 2500 using paired-end technology in a single run by Beijing Biomake Company (Beijing, China).

### Clustering and sequencing

Following a previous report [17], clustering and sequencing were performed on a cBot Cluster Generation System and an Illumina Hiseq 2500 platform, respectively.

### Sequence analysis and assembly

Raw reads of fastq format were firstly processed through in-house perl scripts. In this step, clean reads were obtained by removing reads containing adapter, reads containing ploy-N and low quality reads. At the same time, Q20, Q30, GC-content and sequence duplication level of the clean data were calculated. Cleaned reads shorter than 60 bases were removed because the short reads might represent sequencing artifacts [26]. The qualified reads were assembled into unigenes using short reads assembling program-Trinity [10].

The obtained contigs were annotated against the NCBI non-redundant protein (NR) database using BLASTn (*E*-value<10^−5^) and BLASTx (*E*-value<10^−5^) programs [11]. To annotate the assembled sequences with Gene Ontology (GO) terms, the Swiss-Prot BLAST results were imported into BLAST2GO, a software package that retrieves GO terms, allowing determination and comparison of gene functions [27]. The unigene sequences were also aligned to the Clusters of Orthologous Groups of proteins (COG) database to predict and classify the unigene sequences [28]. Pathway annotations for unigenes were determined using Kyoto Encyclopedia of Genes and Genomes (KEGG) ontology [29]. Finally, the best matches were used to identify coding regions and determine the sequence direction [30].

### Olfactory gene identification and phylogenetic analysis

The annotations of OBP, CSP, SNMP, OR, IR and GR genes in *P*. *interpunctella* were verified by BLASTx and BLASTn programs NCBI. The complete coding region was predicted using the open reading frame (ORF) finder (http://www.ncbi.nlm.nih.gov/gorf/gorf.html) based on the results given by BLASTx. After completing the alignments of the candidate chemosensory genes using ClustalX (2.1), phylogenetic reconstruction for the analysis of OBPs, CSPs, ORs, IRs and GRs was performed by MEGA5.0 software using the neighbor-joining method with 1000 Bootstrap iterations [31]. In addition, the evolutionary distances were assumed by using the Poisson correction method [11].

### Analysis of differentially expressed genes and qRT-PCR verification

To compare the differential expression of chemosensory genes between the male and female antennal transcriptomes of *P*. *interpunctella*, the read number of each olfactory-related gene was converted to FPKM (fragments per kilobase of exon model per million mapped reads) [32].

qRT-PCR was performed to quantify the expression levels of olfactory-related genes in male and female antennae. Total RNA was extracted from 100 antennae as above description. cDNA from antennae of both sexes was synthesized using the SMART^TM^PCR cDNA synthesis kit (Clontech, Mountain View, CA, USA). The β-actin gene (SRP05157) was used as an internal control in each sample, and it was selected as a housekeeping gene in our qRT-PCR test. Real-time PCR was performed on an ABI 7500 using SYBR green dye binding to double-stranded DNA at the end of each elongation cycle. Primer sequences were designed using the Primer Premier 5.0 program (S1 Table). Real-time PCR was conducted with our previous method [13]. Briefly, 10.0 μL of 2×SYBR Green PCR Master Mix, 0.4 μL of primer, 2.0 μL of sample cDNA (100 ng μL^-1^) and 7.2 μL of sterilized ultrapure water were mixed to form a 20 μL reaction system.After an initial denaturation step at 95°C for 3 min, amplifications were carried out with 40 cycles at a melting temperature of 95°C for 10 s and an annealing temperature of 60°C for 30 s. To check reproducibility, qRT-PCR test for each sample was performed with three technical replicates and three biological replicates.

### qRT-PCR analysis

Relative quantification was determined using the comparative 2^-ΔΔCt^ method [33]. All data were normalized to endogenous β-actin levels from the same individual samples. The relative fold change was assessed by comparing the expression level in male moths to that in females [34]. The results were presented as the means of the fold change in three biological duplicates. The comparative analyses of chemosensory genes between sexes were determined by one-way analysis of variance (ANOVA) using SPSS 19.0, with *p*-value of 0.05 considered significant.

## Results

### Sequence analysis and assembly

**Fig 1:**
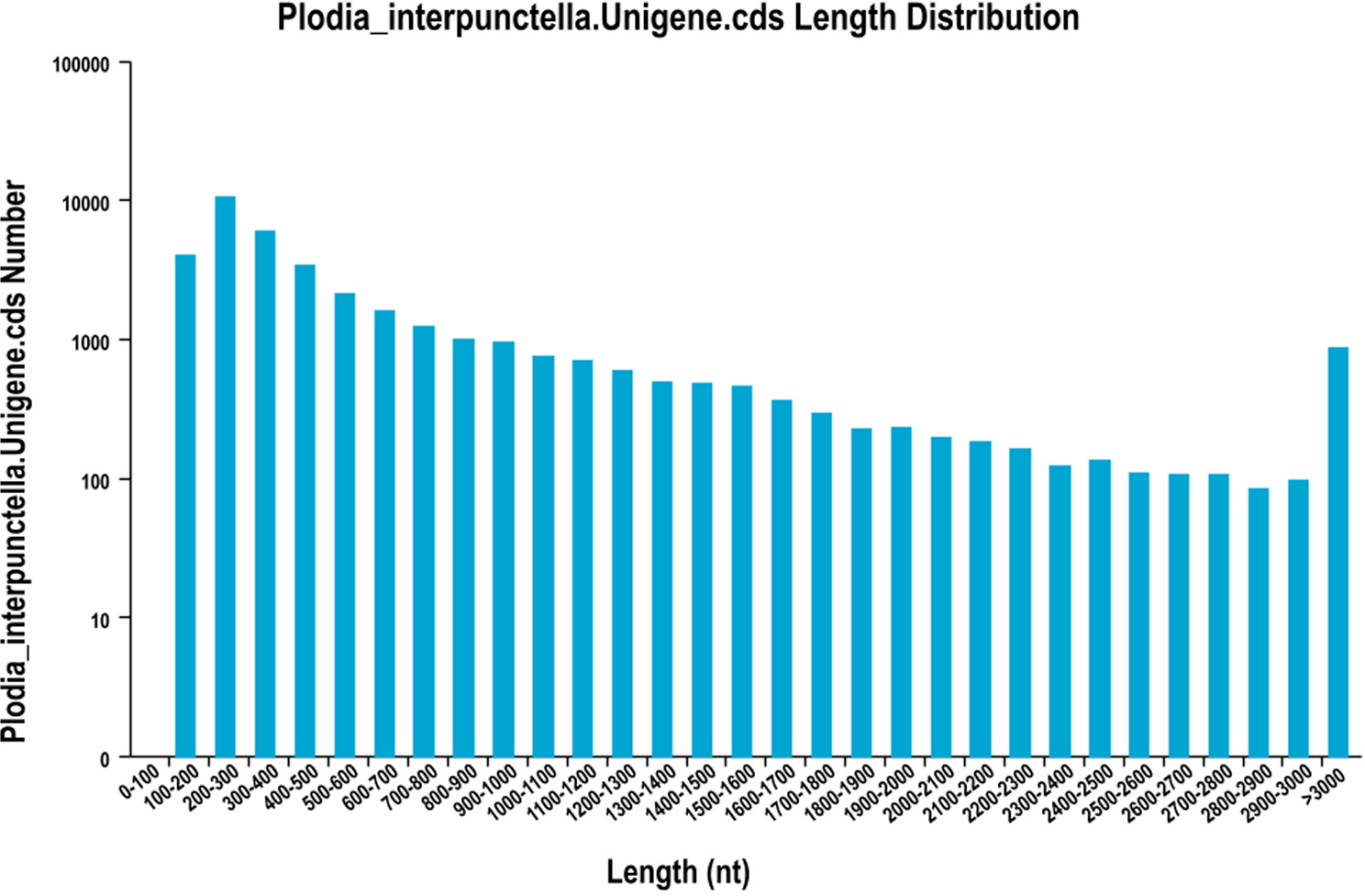
Distribution of Unigene length of *Plodia interpunctella*.

cDNA library of *Plodia interpunctella* was constructed using the TRINITY *de novo* assembly program, and short-read sequences were assembled into 150,633 transcripts with a mean length of 1,491 bp and an N50 of 3,567 bp. A total of 20,261 scaffolds (13.45%) were longer than 1,000 bp, and 36,148 scaffolds (24.00%) were longer than 2,000 bp. The scaffolds were subjected to cluster and assembly analyses. Subsequently, 87,300 unigenes were obtained with a mean length of 699 bp and an N50 of 1,282 bp (Fig 1, Table 1). The length distribution of unigenes revealed that 26,054 unigenes (29.84%) were longer than 500 bp and 12,485 unigenes (14.30%) were longer than 1,000 bp (Table 1). The raw reads of *P*. *interpunctella* transcriptome have been deposited into the NCBI SRA database (accession number: SRR6002827 and SRR6002828), and the Transcriptome Shotgun Assembly (TSA) project has been deposited at DDBJ/ENA/GenBank under the accession GFWQ00000000. The version described in this paper is the first version, GFWQ01000000. The detailed TSA sequences could be obtained from Genbank (https://www.ncbi.nlm.nih.gov/Traces/wgs/?val=GFWQ01&display=contigs&page=1).

**Table 1:** An overview of the sequencing and assembly process.

Length (bp)	Transcript	Unigene
200–300	39,574(26.27%) (26.27%)	35,516(40.68%)
300–500	31,475(20.90%) (20.90%)	25,729(29.47%)(29.74%)
500–1000	23,172(15.38%)) (15.38%)	13,569(15.54%)
1000–2000	20,261(13.45%)	6,176(7.07%)
2000+	36,148(24.00%)	6,309(7.23%)
Total Number	150,633	87,300
Total Length	224,546,425	61,027,187
N50 Length	3,567	1,282
Mean Length	1490.69	699.05

### Sequence annotation

The unigene annotation showed that 27,920 unigenes (31.98%) significantly matched in the NR database and 15,815 unigenes (18.12%) had significant matches in the Swiss-Prot database. A total of 31,921 unigenes (36.56%) were successfully annotated in the NR, Swiss-Prot, KEGG, GO and COG databases (Table 2), whereas 55,379 unigenes (63.44%) were unmapped in those databases.

**Table 2:** Functional annotation of the *Plodia interpunctella*.

Annotated databases	unigene	≥300 bp	≥1000 bp
COG_annotation	10,106	4,383	3,554
GO_annotation	15,893	6,734	5,269
KEGG_annotation	15,016	6,404	4,654
SwissProt_annotation	15,815	6,420	6,205
nr_annotation	27,920	11,530	9,415
Total	31,921	13,492	9,548

COG = Cluster of Orthologous Groups of proteins; GO = Gene Ontology; KEGG = Kyoto Encyclopedia of Genes and Genomes; nr = nonredundant protein.

**Fig 2:**
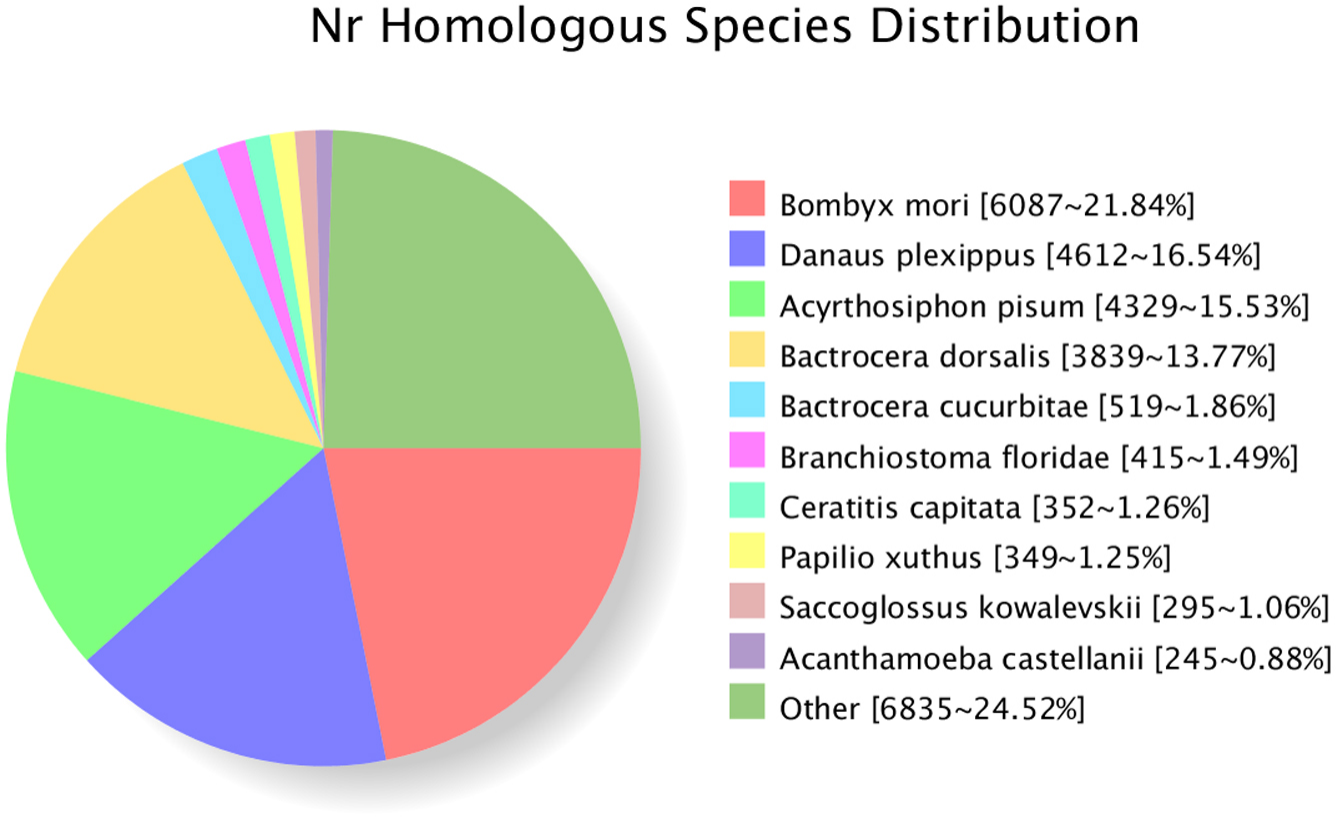
Characteristics of homology search for *Plodia interpunctella* unigenes. The number of unigenes matching the top ten species using BlastX in the Nr database is indicated in square brackets.

NR database queries revealed that a high percentage of *P*. *interpunctella* sequences had closely matched sequences in *Bombyx mori* (6,087, 21.84%), followed by *Danaus plexippus* (4,612, 16.54%), *Acyrthosiphon pisum* (4,329, 15.53%) and *Bactrocera dorsalis* (3,839, 13.77%) (Fig 2).

**Fig 3:**
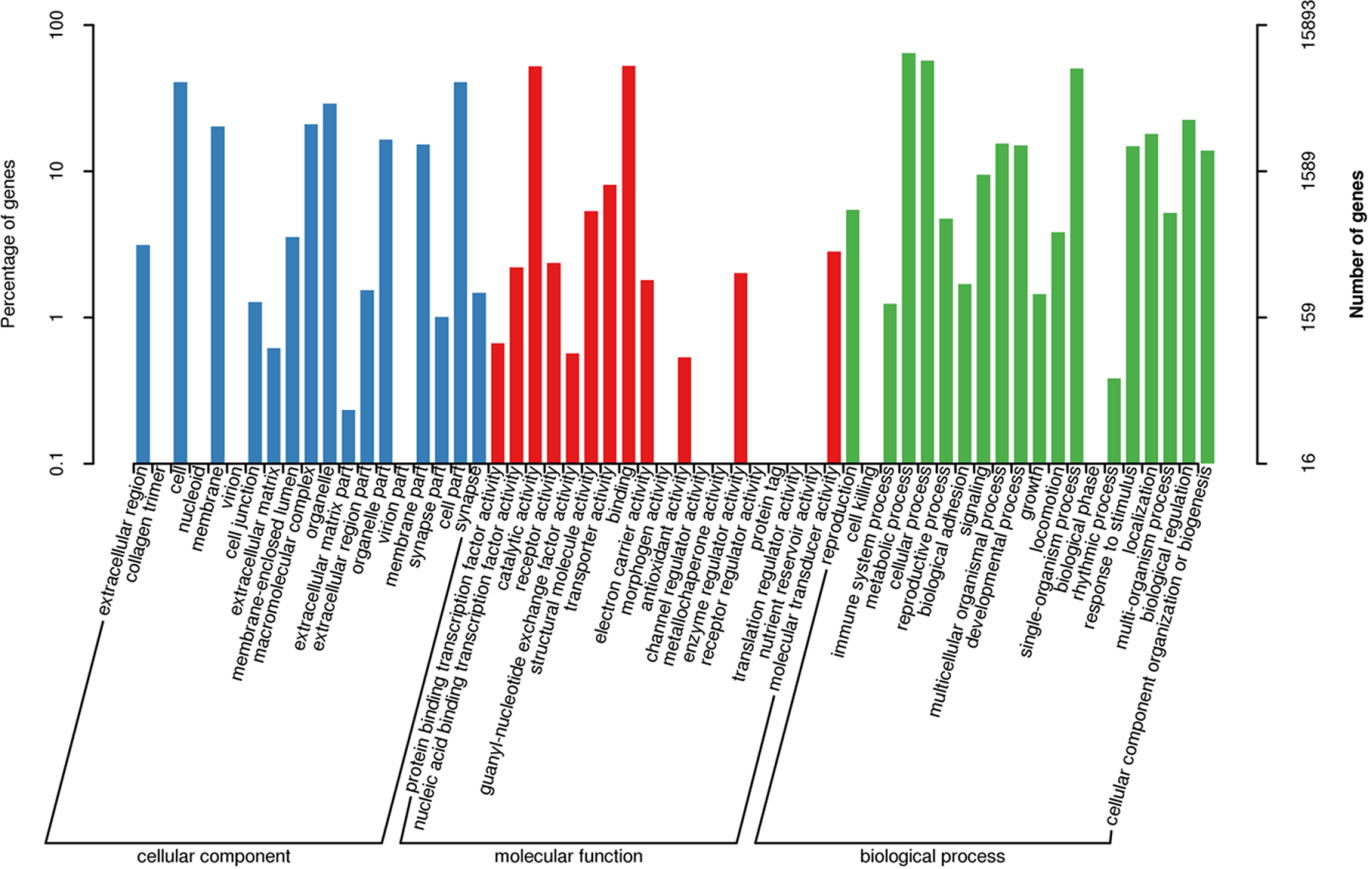
Functional annotation of assembled sequences based on gene ontology (GO) categorization.

For GO analysis, 15,893 unigenes (18.21%) could be assigned to three GO terms as follows: cellular components, molecular functions and biological process (Fig 3). The “cellular components” and “molecular functions” were most represented by 18.79% and 21.04% transcripts, respectively. In the “cellular components” ontology, the terms were mainly distributed in cell (20.71%) and cell part (20.71%). In the “molecular functions” ontology, the terms of binding function and catalytic activity were the most represented (39.91% and 39.80%, respectively) (Fig 3).

**Fig 4:**
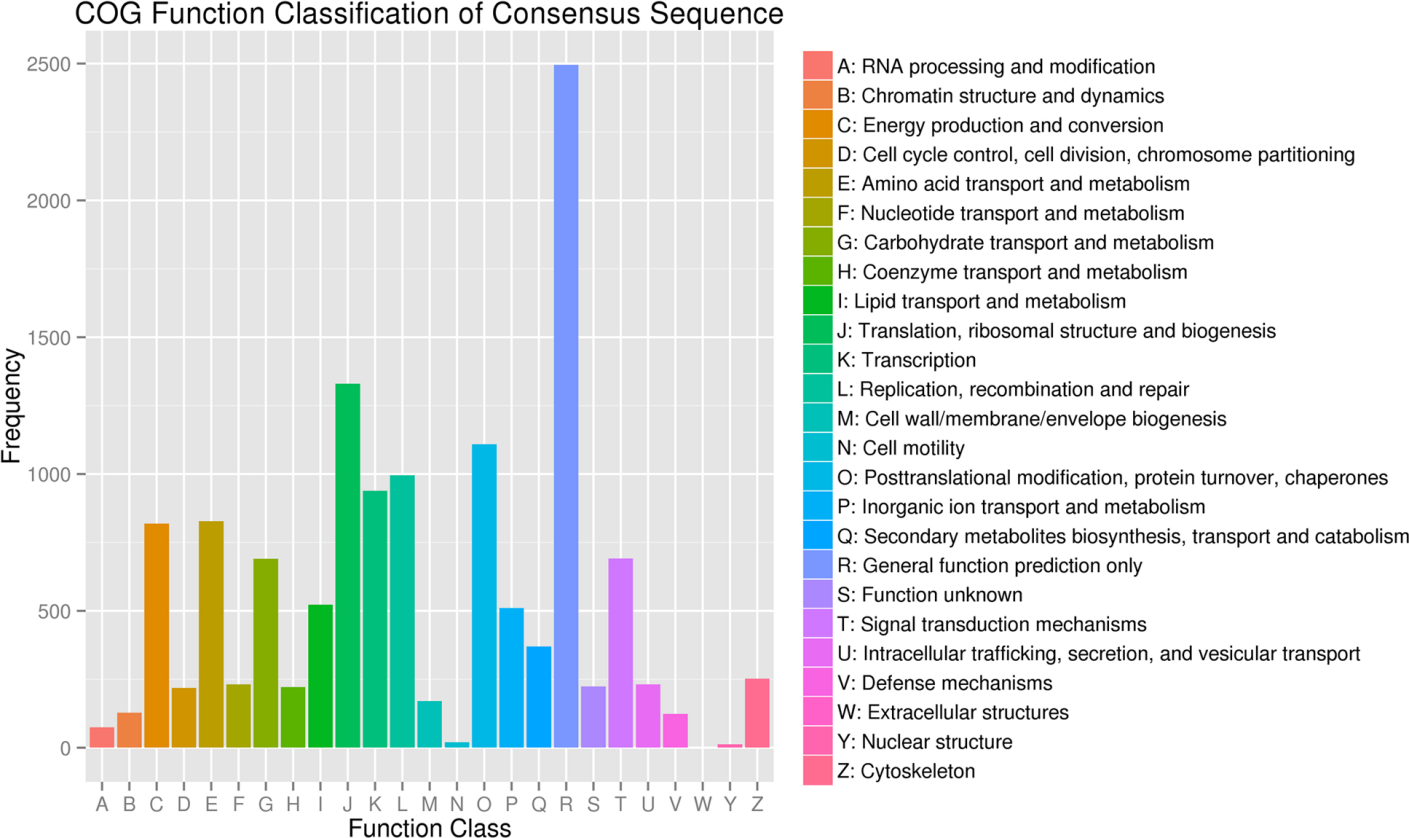
Cluster of orthologous groups (COG) classification.

To predict and classify the functional genes, all unigenes were searched against the COG database. A total of 10,106 unigenes could be assigned to 25 specific categories according to the COG classification results. “General function prediction” (2,494, 24.68%) was the largest group, and the categories of “cell motility” (20, 0.20%) and “nuclear structure” (11, 0.11%) were the smallest groups (Fig 4). In addition, 290 pathways were predicted in the KEGG database, representing 15,016 unigenes.

### Identification of olfactory-related genes

In the present study, we identified 117 olfactory-related genes from antennal transcriptome of *P*. *interpunctella*, including 29 OBPs, 15 CSPs, three SNMPs, 47 ORs, nine GRs and 14 IRs. All genes were named according to a four-letter code (first letter of the genus name followed by the first three letters of the species name) + OR + number according to the ORF lengths. Analysis of differential expression of unigenes indicated that 1,031 genes showed differences between the antennal transcriptomes of male and female *P*. *interpunctella*, including 93 up-regulated and 938 down-regulated genes using female result as the reference standard.

### Candidate OBPs in antennae of *Plodia interpunctella*

In antennal transcriptomes of *P*. *interpunctella*, 29 OBP genes were annotated based on the tBLASTn results, including four pheromone-binding proteins (PBPs) and one general odorant-binding protein (GOBP) (Table 3). Among the 29 OBP genes, 17 had intact ORFs with lengths ranging from 291 bp to 1,014 bp. The BLASTx results indicated that 24 identified PintOBPs shared relatively higher amino acid identities (>50%) with Lepidoptera OBPs in NCBI.

**Table 3:** The Blastx matches of *Plodia interpunctella* candidate OBP genes.

Gene ID	Gene name	Full length	ORF (aa)	Blastx annotation (Reference/Name/Species)	Score	*E*-value	Identity (%)	FPKM values	
								Female	Male
c34980.graph_c0	OBP1	Y	338	ALD65883.1 | odorant binding protein 9 [*Spodoptera litura*]	325	4e-107	56	40.51	36.71
c20255.graph_c0	OBP2	Y	252	ADD71058.1 | odorant-binding protein [*Chilo suppressalis*]	376	5e-130	69	302.91	437.33
c31451.graph_c0	OBP3	N	242	BAV56797.1 | odorant binding protein 10 [*Ostrinia furnacalis*]	320	4e-108	67	271.44	283.75
c44096.graph_c0	OBP4	N	210	ALT31639.1 | odorant-binding protein 9 [*Cnaphalocrocis medinalis*]	309	6e-105	71	0.18	0.98
c16901.graph_c0	OBP5	N	206	BAV56794.1 | odorant binding protein 7 [*Ostrinia furnacalis*]	156	7e-45	47	154.93	139.43
c31236.graph_c0	OBP6	Y	197	EHJ74351.1 | odorant-binding protein 2 [*Danaus plexippus*]	293	7e-99	81	0	5.26
c29670.graph_c0	OBP7	N	180	BAV56800.1 | odorant binding protein 13 [*Ostrinia furnacalis*]	242	1e-79	76	1.71	0.32
c33870.graph_c0	OBP8	Y	180	AII00998.1 | odorant binding protein [*Dendrolimus kikuchii*]	130	2e-35	44	2443.01	1957.54
c42887.graph_c0	OBP9	Y	170	JAV45894.1 | odorant binding protein 19 [*Mythimna separata*]	194	6e-61	61	0.53	0.93
c16794.graph_c0	OBP10	N	164	AGK24577.1 | odorant-binding protein 1 [*Chilo suppressalis*]	100	3e-24	36	2682.84	1629.98
c29465.graph_c0	OBP11	Y	149	JAP88618.1 | OBP [*Conogethes punctiferalis*]	155	5e-46	49	2747.16	2241.31
c33892.graph_c0	OBP12	N	146	BAV56795.1 | odorant binding protein 8 [*Ostrinia furnacalis*]	213	7e-69	83	898.18	735.77
c34383.graph_c0	OBP13	Y	142	JAI18227.1 | Antennal Binding Protein X [*Epiphyas postvittana*]	209	2e-67	68	455.66	968.61
c55087.graph_c0	OBP14	Y	142	ANC68517.1 | odorant-binding protein 29 [Chilo suppressalis]	135	3e-38	51	0	0.33
c40570.graph_c0	OBP15	Y	142	AFD34173.1 | odorant binding protein 5 [*Argyresthia conjugella*]	235	8e-78	77	4746.92	1927.26
c40388.graph_c2	OBP16	Y	139	BAV56799.1 | odorant binding protein 12 [*Ostrinia furnacalis*]	245	1e-81	83	335.28	119.16
c20185.graph_c0	OBP17	Y	137	AGM38607.1 | odorant binding protein [*Chilo suppressalis*]	207	5e-67	78	21184.85	47032.77
c47744.graph_c0	OBP18	N	135	AGC82130.1 | odorant-binding protein 1 [*Bactrocera dorsalis*]	275	1e-93	100	0.66	0
c25858.graph_c0	OBP19	Y	134	ALS03864.1 | odorant-binding protein 16 [*Ectropis obliqua*]	248	3e-83	90	146.09	90.26
c32612.graph_c0	OBP20	Y	114	JAV45893.1 | odorant binding protein 20 [*Mythimna separata*]	157	3e-47	61	3.76	18.17
c9234.graph_c1	OBP21	N	104	ALD65893.1 | odorant binding protein 19 [*Spodoptera litura*]	87.8	2e-19	63	0.25	0.25
c37240.graph_c7	OBP22	Y	97	BAV56803.1 | odorant binding protein 16 [*Ostrinia furnacalis*]	130	1e-36	61	27.47	35.78
c32421.graph_c0	OBP23	N	71	JAI18081.1 | Odorant Binding Protein [*Epiphyas postvittana*]	81.6	4e-18	47	6.5	2.27
c36217.graph_c0	OBP24	N	54	JAV45888.1 | odorant binding protein 25 [*Mythimna separata*]	108	1e-28	94	3.82	4.52
c32145.graph_c0	PBP1	N	182	AHZ89398.1 | pheromone-binding protein 2 [*Grapholita molesta*]	221	3e-71	62	3494.34	1257.15
c30184.graph_c0	PBP2	N	170	ADT78495.1 | pheromone binding protein 1 [*Ostrinia nubilalis*]	241	2e-79	69	6152.67	43181.18
c16802.graph_c0	PBP3	Y	170	AAD39447.1 | pheromone binding protein [*Ostrinia nubilalis*]	234	9e-77	67	99.62	2860.16
c34173.graph_c0	PBP4	Y	124	AAF06142.1 | pheromone binding protein [*Synanthedon exitiosa*]	181	1e-56	67	1390.9	2307.02
c34904.graph_c0	GOBP1	Y	190	AGS36742.1 | GOBP1 [*Sesamia inferens*]	254	3e-84	75	2790.14	1853.61

**Fig 5:**
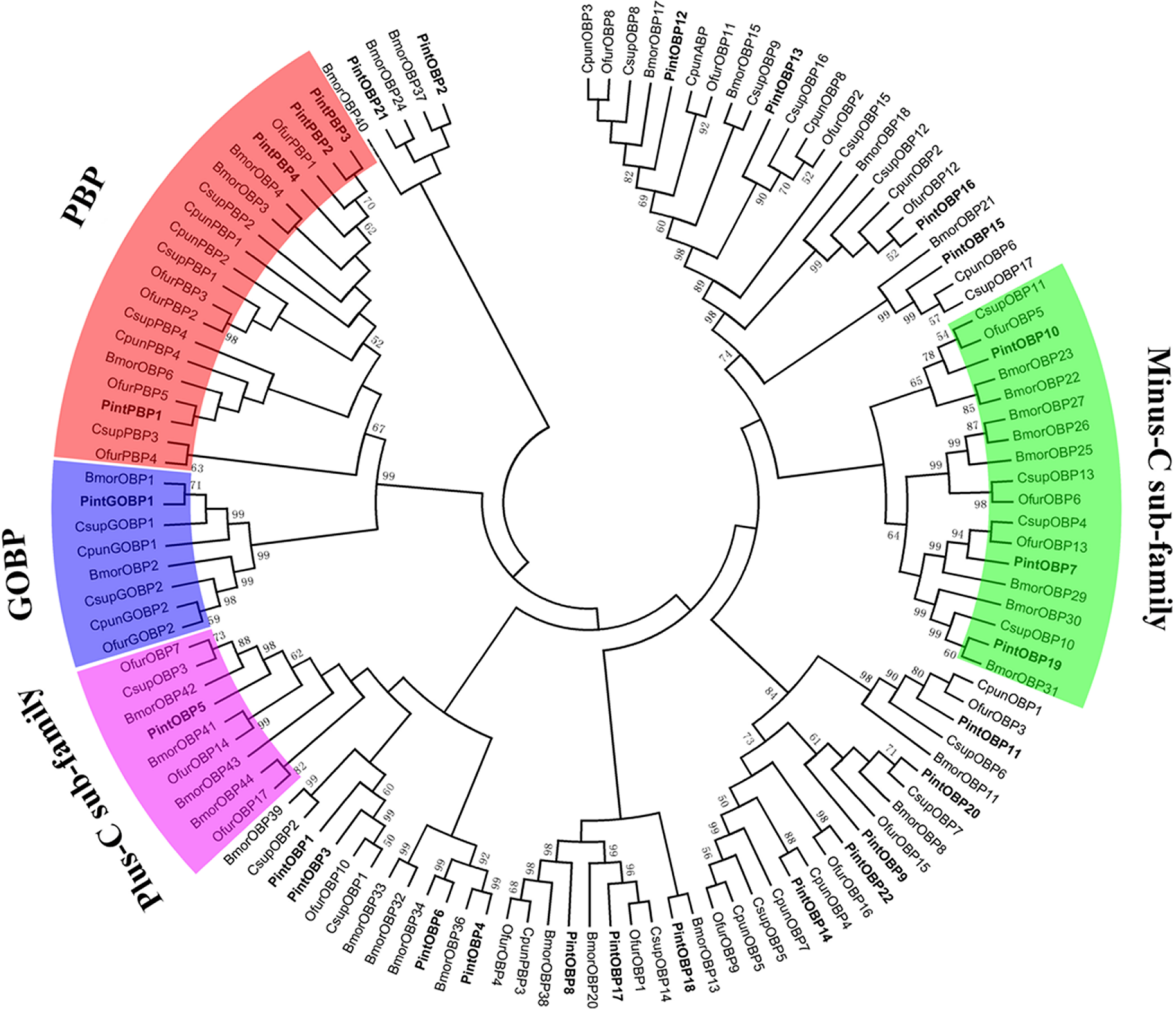
Neighbor-joining tree of candidate OBPs from *Plodia interpunctella*, *Conogethes punctiferalis*, *Ostrinia furnacalis*, *Chilo suppressalis* and *Bombyx mori*. The protein names and sequences of OBPs that were used in this analysis are listed in S2 Table.

**Fig 6:**
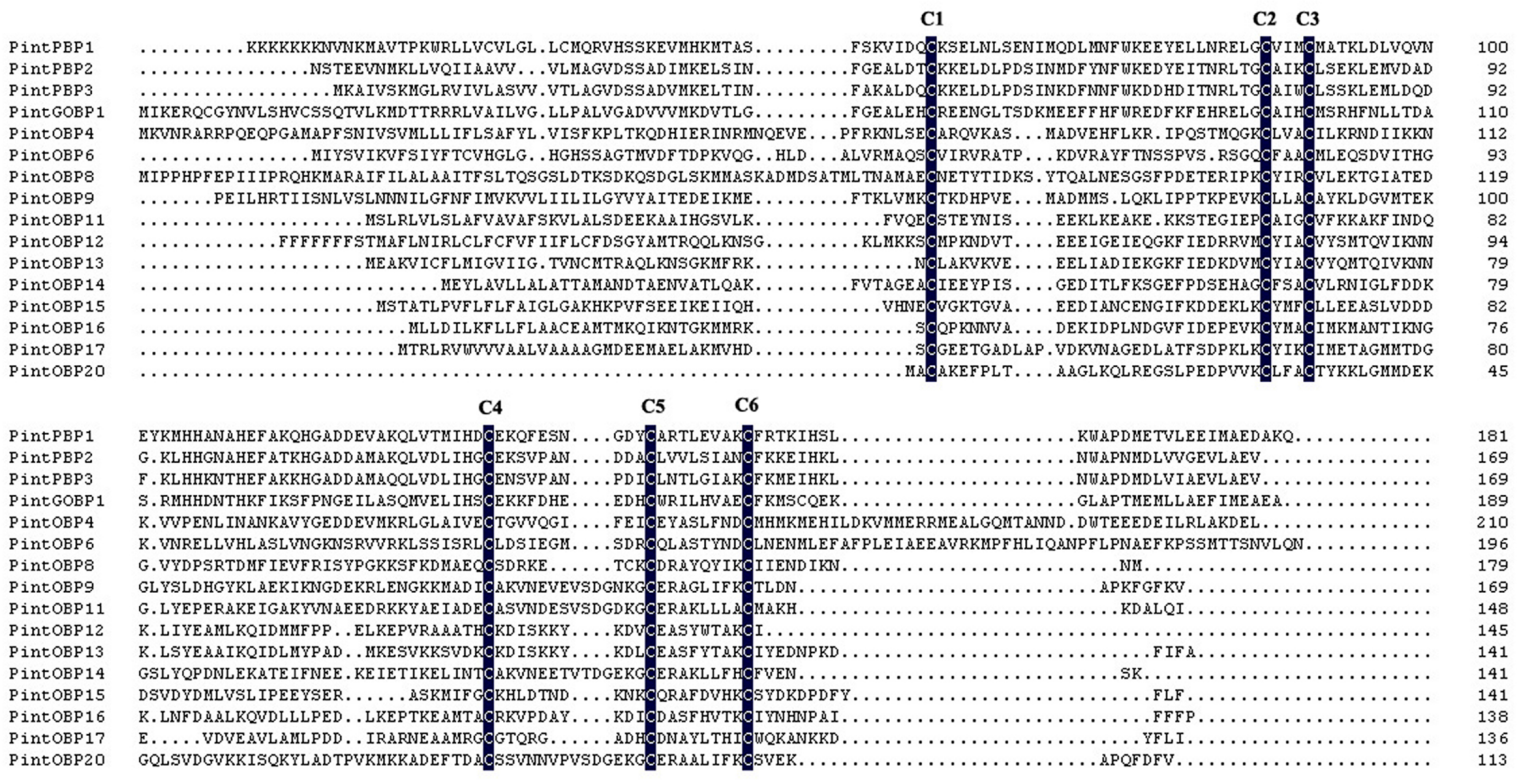
Sequences alignment of classic PintOBPs.

A neighbor-joining tree of 123 OBP sequences was constructed using OBPs of Lepidoptera species, including four species in Pyraloidea family (*P*. *interpunctella*, *Conogethes punctiferalis*, *Ostrinia furnacalis* and *Chilo suppressalis*), and *Bombyx mori*. Due to the lack of antennal transcriptome information of genus Plodia, we selected three closer relatives of *P*. *interpunctella* to compare the OBPs. *B*. *mori* was chosen to study the patterns and functions of OBPs, because BmorOBPs were widely recognized and verified. Most PintOBPs had a high similarity to known Pyralidae OBPs, which could possibly be attributed to that both *P*. *interpunctella* and Pyralidae belong to Pyraloidea family. Phylogenetic tree showed that the PintPBP2-4 was clustered into the PBP family, and the PintGOBP1 was clustered into the GOBP family. In the PBP family, PintPBP2, PintPBP3 and PintPBP4 were stretched in the same branch with the bootstrap values as high as 62 (Fig 5). Based on the number of conserved cysteines, OBPs can be divided into three subclasses: classic OBPs, Plus-C OBPs and Minus-C OBPs [35]. As for *P*. *interpunctella*, PintOBP7, PintOBP10, PintOBP15 and PintOBP19 were clustered into the Minus-C OBP family. Meanwhile PintOBP5 belonged to the Plus-C OBP family. According to multiple amino acid sequence alignments, 16 OBPs (PintOBP1-4, PintOBP6, PintOBP8-9, PintOBP11-17, PintOBP20, PintPBP1-3 and PintGOBP1) totally matched with C_1_-X_25-30_-C_2_-X_3_-C_3_-X_36-42_-C_4_-X_8-14_-C_5_-X_8_-C_6_ (X stands for any amino acid), and they were identified as classic OBPs (Fig 6) [36].

**Fig 7:**
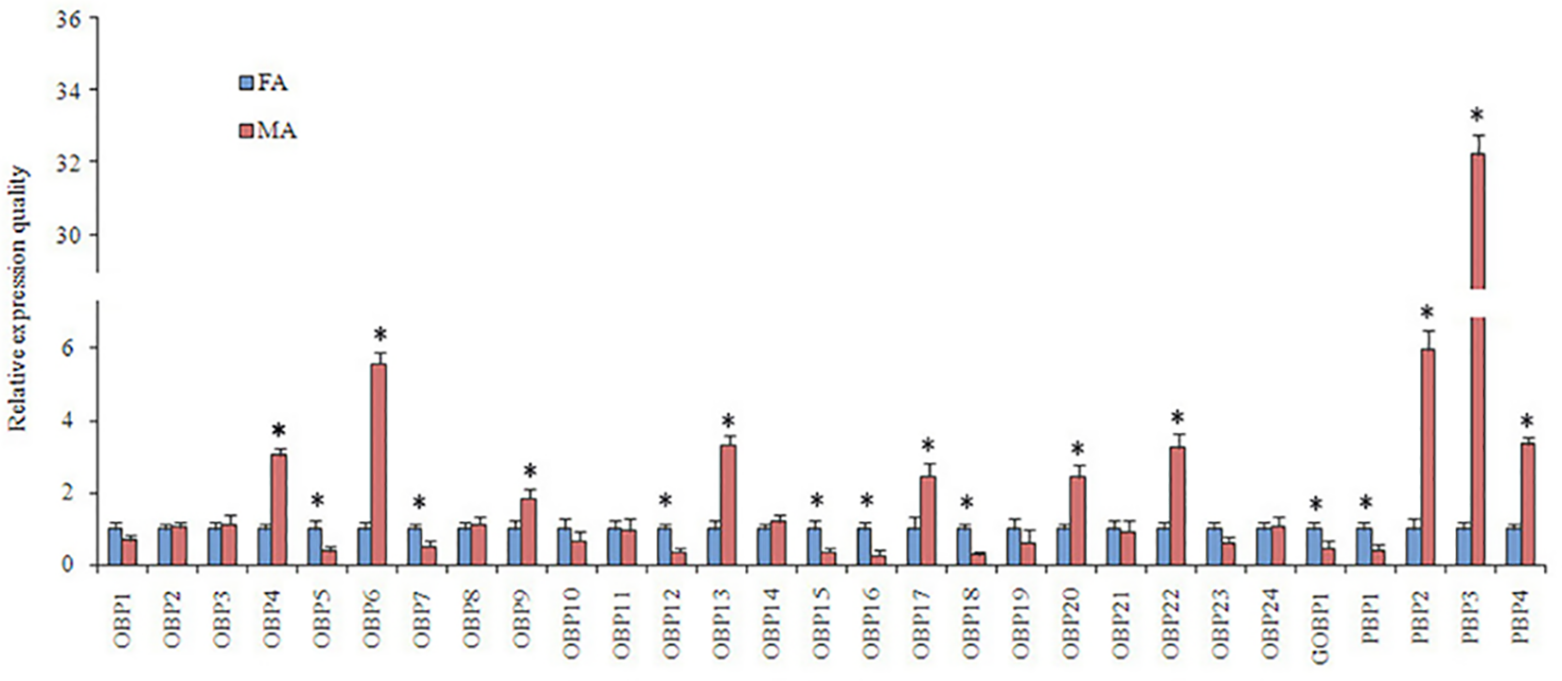
*P*. *interpunctella* OBP transcript levels in different antennae measured by qRT-PCR. MA: male antennae; FA: female antennae. The *β-actin* was used as internal control to normalize transcript levels in each sample. The standard error represented by the error bar, and the asterisk above each bar denote significant differences (*p*<0.05).

Base on FPKM measure, the OBPs with an FPKM value greater than 1,000 were defined as high-expression genes [30]. The FPKM analysis revealed that 10 OBP genes (PintOBP8, PintOBP10, PintOBP11, PintOBP15, PintOBP17, PintPBP1-4 and PintGOBP1) were highly abundant in antennae of *P*. *interpunctella* (FPKM>1,000) (Table 3). Furthermore, the qRT-PCR expression levels of 29 PintOBP genes indicated that nine OBP genes (PintOBP4, PintOBP6, PintOBP9, PintOBP13, PintOBP17 PintOBP20, PintOBP22 and PintPBP2-3) were significantly expressed in the male antennae (1.8 to 33.5 times compared with females). Eight OBPs (PintOBP5, PintOBP7, PintOBP12, PintOBP15-16, PintOBP18, PintPBP1 and PintGOBP1) were significantly expressed in the female antennae (1.7 to 3.8 times compared with males). The other eight OBP genes (PintOBP1-3, PintOBP8, Pint10-11, PintOBP14 and PintOBP21) showed similar expression levels in the male and female antennae (Fig 7).

### Candidate CSPs in antennae of *Plodia interpunctella*

**Fig 8:**
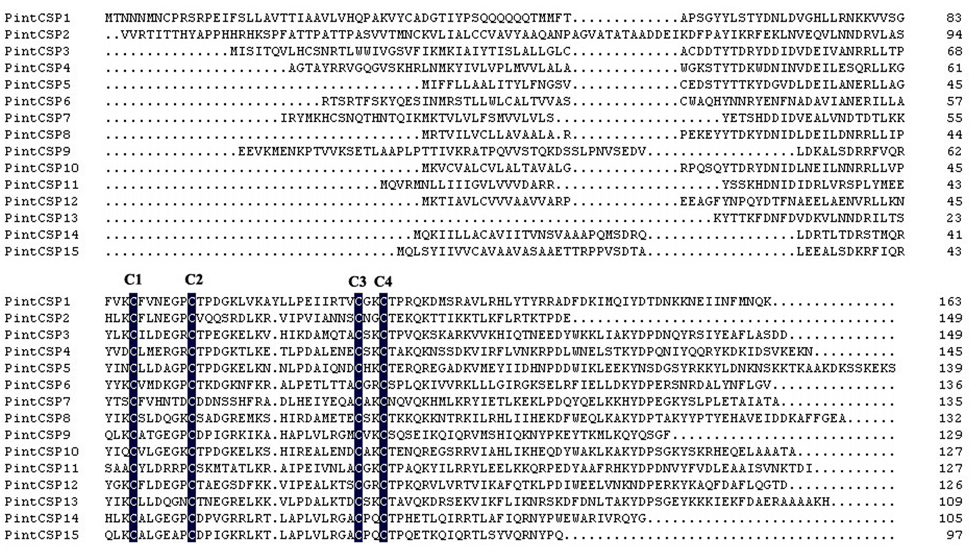
Sequences alignment of candidate PintCSPs.

In the antennal transcriptomes of *P*. *interpunctella*, 15 putative CSPs were identified with lengths ranging from 291 bp to 492 bp. All identified PintCSPs were verified according to the four-cysteines pattern C_1_-X_6-8_-C_2_-X_18-19_-C_3_-X_2_-C_4_ (Fig 8) [36]. Among the 15 PintCSP genes, eight had intact ORFs with lengths ranging from 318 bp to 492 bp. The BLASTx results indicated that 13 identified PintCSPs shared relatively higher amino acid identities (>50%) with Lepidoptera CSPs in NCBI (Table 4).

**Table 4:** The Blastx matches of *Plodia interpunctella* candidate CSP and SNMP genes.

Gene ID	Gene name	Full length	ORF (aa)	Blastx annotation (Reference/Name/Species)	Score	*E*-value	Identity (%)	FPKM values	
								Female	Male
c21705.graph_c0	CSP1	Y	164	AGE97647.1 | chemosensory protein 8 [*Aphis gossypii*]	251	2e-83	78	1.9	0
c6549.graph_c0	CSP2	N	150	APB03439.1 | chemosensory protein 3 [*Sitobion avenae*]	192	1e-60	91	0.7	0
c39316.graph_c3	CSP3	Y	150	AGR39578.1 | chemosensory protein 8 [*Agrotis ipsilon*]	177	5e-55	63	343.76	276.61
c31754.graph_c0	CSP4	N	146	JAV45874.1 | chemosensory protein 7 [*Mythimna separata*]	210	6e-88	80	1503.31	1403.08
c33894.graph_c0	CSP5	Y	141	BAV56812.1 | chemosensory protein 8 [*Ostrinia furnacalis*]	194	2e-61	66	463.43	384.09
c50839.graph_c0	CSP6	N	137	ALS03837.1 | chemosensory protein 12 [*Ectropis obliqua*]	195	3e-62	78	0.27	0.37
c25625.graph_c0	CSP7	N	136	BAV56814.1 | chemosensory protein 10 [*Ostrinia furnacalis*]	120	1e-32	45	33.2	28.58
c32629.graph_c0	CSP8	Y	133	AFR92093.1 | chemosensory protein 9 [*Helicoverpa armigera*]	162	2e-49	61	4.91	3.65
c80512.graph_c0	CSP9	N	130	APB03440.1 | chemosensory protein 4 [*Sitobion avenae*]	261	3e-88	97	0.38	0.13
c30384.graph_c0	CSP10	Y	128	AEB54579.1 | CSP5 [*Helicoverpa armigera]*	191	2e-60	69	386.2	302.77
c36418.graph_c0	CSP11	Y	128	AIX97839.1 | chemosensory protein [*Cnaphalocrocis medinalis*]	115	7e-31	44	1.57	3.43
c32937.graph_c0	CSP12	Y	127	BAV56806.1 | chemosensory protein 2 [*Ostrinia furnacalis*]	180	2e-65	65	339.32	272.62
c50413.graph_c0	CSP13	N	110	AGE97642.1 | chemosensory protein 2 [*Aphis gossypii*]	194	3e-62	89	1.26	0
c52233.graph_c0	CSP14	Y	106	JAV45868.1 | chemosensory protein 13 [*Mythimna separata*]	165	6e-51	75	0.53	0.54
c32777.graph_c0	CSP15	N	97	AKT26494.1 | chemosensory protein 20 [*Spodoptera exigua*]	168	2e-52	85	2.61	3.45
c16843.graph_c0	SNMP1	N	510	AOG12884.1 | sensory neuron membrane protein [*Eogystia hippophaecolus*]	863	0.0	79	71.33	152.34
c35212.graph_c1	SNMP2	Y	495	ADQ73889.1 | sensory neuron membrane protein 2 [*Ostrinia nubilalis*]	762	0.0	70	252.42	365.34
c74901.graph_c0	SNMP3	N	121	KPI91875.1 | Sensory neuron membrane protein 1 [*Papilio xuthus*]	146	3e-38	54	0.53	0.27

**Fig 9:**
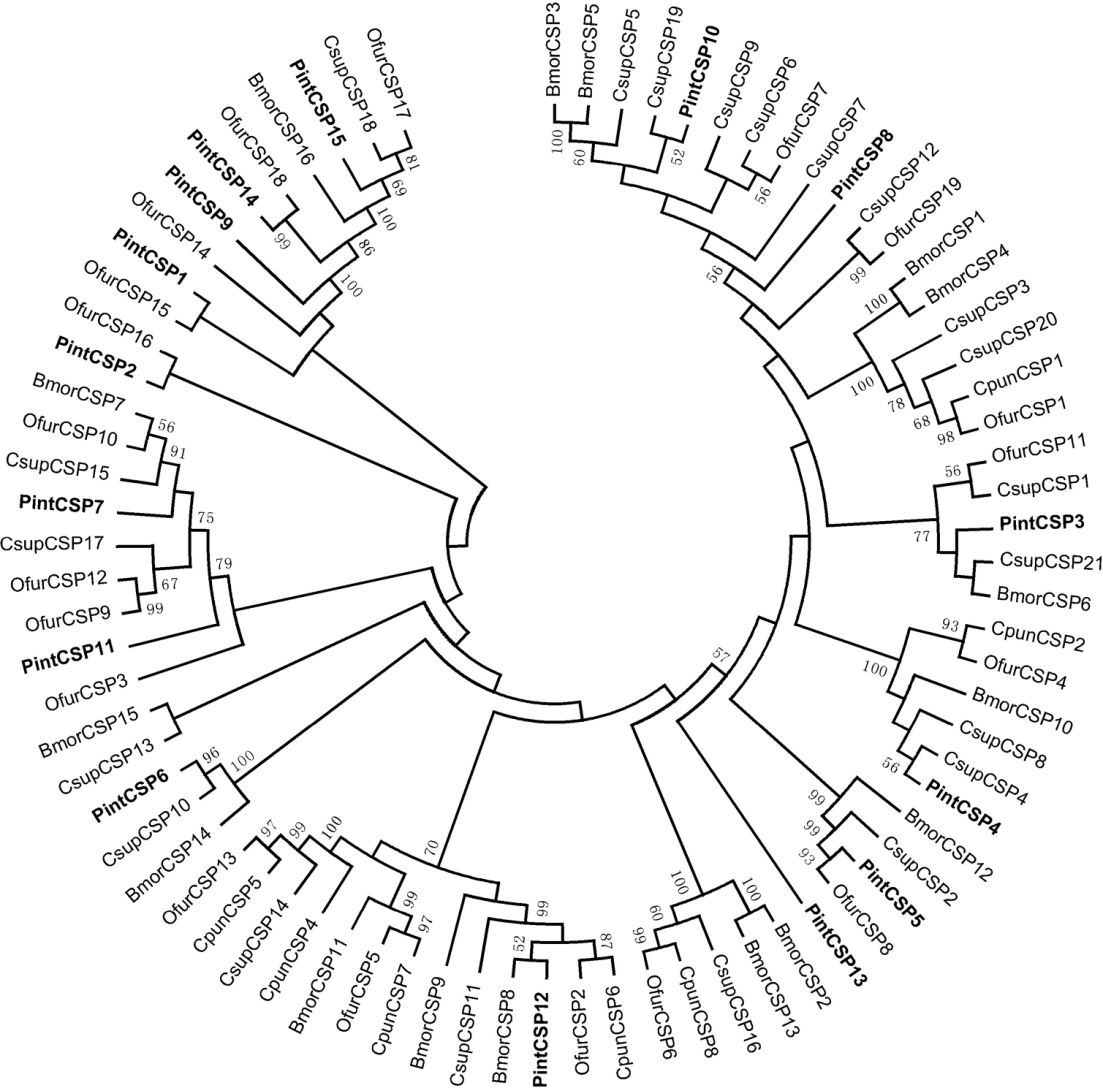
Neighbor-joining tree of candidate CSPs from *Plodia interpunctella*, *Conogethes punctiferalis*, *Ostrinia furnacalis*, *Chilo suppressalis* and *Bombyx mori*. The protein names and sequences of CSPs that were used in this analysis are listed in S3 Table.

A neighbor-joining tree of 78 CSP sequences was constructed based on Lepidoptera species from *C*. *punctiferalis*, *O*. *furnacalis*, *C*. *suppressalis* and *B*. *mori*. PintCSPs were distributed on various branches throughout the cladogram (Fig 9). The phylogenetic tree showed that PintCSP14, PintCSP2, PintCSP5 and PintCSP1 were clustered together with OfurCSPs, with relatively higher bootstrapping values.

**Fig 10:**
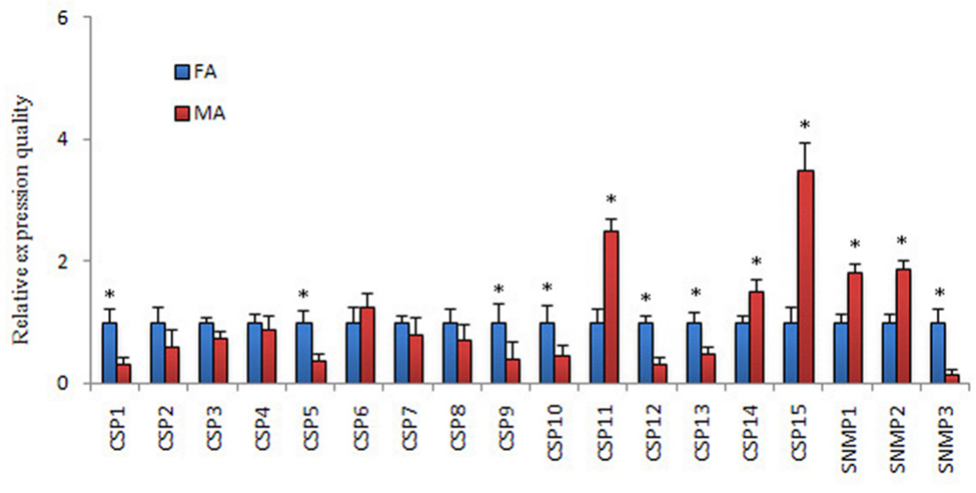
*P*. *interpunctella* CSP transcript levels in different antennae measured by qRT-PCR. MA: male antennae; FA: female antennae. The internal control *β-actin* was used to normalize transcript levels in each sample. The standard error represented by the error bar, and the asterisk above each bar denote significant differences (*p*<0.05).

The FPKM analysis revealed that only PintCSP4 was highly abundant in antennal transcriptomes of *P*. *interpunctella* (FPKM>1,000) (Table 4). The qRT-PCR results indicated that three PintCSP genes (PintCSP11, PintCSP14 and PintCSP15) were significantly expressed in the male antennae (1.5 to 3.5 times compared with females). Seven PintCSPs (PintCSP1-2, PintCSP5, PintCSP9-10 and PintCSP12-13) were specifically expressed in the female antennae (1.7 to 3.2 times compared with males) (Fig 10).

### Candidate ORs

We identified 47 OR genes in the antennal transcriptomes of *P*. *interpunctella*, in which 36 PintORs had intact ORFs with lengths ranging from 219 bp to 1,422 bp with four to seven transmembrane domains (Table 5).

**Table 5:** The Blastx matches of *Plodia interpunctella* candidate OR genes.

Gene ID	Gene name	Full length	ORF (aa)	Blastx annotation (Reference/Name/Species)	Score	*E*-value	Identity (%)	FPKM values	
								Female	Male
c40585.graph_c0	OR1	Y	474	AFQ94048.1 | olfactory receptor 2 [*Chilo suppressalis*]	928	0.0	92	430.52	576.23
c32962.graph_c0	OR2	Y	452	AIT69911.1 | olfactory receptor 71 [*Ctenopseustis herana*]	608	0.0	64	2.59	3.04
c36497.graph_c0	OR3	N	449	ALT31655.1 | odorant receptor 1 [*Cnaphalocrocis medinalis]*	702	0.0	76	8.04	2.88
c28679.graph_c0	OR4	Y	430	ALM26234.1 | odorant receptor 44 [*Athetis dissimilis*]	712	0.0	77	3.45	3.52
c39092.graph_c0	OR5	Y	429	AGI96750.1 | olfactory receptor 13 [*Spodoptera litura*]	399	3e-133	44	20	189.41
c31116.graph_c0	OR6	Y	424	ANZ03153.1 | olfactory receptor 40 [*Cnaphalocrocis medinalis*]	525	0.0	57	13.84	3.03
c38802.graph_c4	OR7	Y	417	AFP66948.1 | odorant receptor 4 [*Amyelois transitella*]	565	0.0	65	4.25	0.13
c38263.graph_c0	OR8	Y	411	ALM26235.1 | odorant receptor 45 [*Athetis dissimilis*]	608	0.0	71	4.55	5.05
c36791.graph_c0	OR9	Y	409	AOG12913.1 | odorant receptor [*Eogystia hippophaecolus*]	258	7e-79	34	6.05	2.25
c34023.graph_c0	OR10	N	409	AOG12906.1 | odorant receptor [*Eogystia hippophaecolus*]	400	1e-134	50	7.72	4.54
c31249.graph_c0	OR11	N	408	CUQ99400.1 | Olfactory receptor 17 [*Manduca sexta*]	435	2e-148	52	2.41	0
c39086.graph_c1	OR12	Y	406	AIG51899.1 | odorant receptor [*Helicoverpa armigera*]	266	2e-82	38	5.66	4.67
c36343.graph_c0	OR13	Y	404	ALM26238.1 | odorant receptor 53 [*Athetis dissimilis*]	441	3e-151	51	7.28	4.62
c40271.graph_c3	OR14	Y	404	AOG12941.1 | odorant receptor [*Eogystia hippophaecolus*]	468	3e-161	55	5.2	5.11
c16387.graph_c0	OR15	Y	400	AOG12915.1 | odorant receptor [*Eogystia hippophaecolus]*	508	3e-177	62	3.23	3.89
c40164.graph_c0	OR16	Y	396	AQQ73507.1 | olfactory receptor 27 [*Heliconius melpomene* rosina]	506	1e-176	62	13.69	7.6
c37581.graph_c0	OR17	Y	393	AII01084.1 | odorant receptor [*Dendrolimus kikuchii*]	506	9e-177	62	3.37	3.56
c36558.graph_c0	OR18	N	391	ANZ03145.1 | olfactory receptor 32 [*Cnaphalocrocis medinalis*]	439	2e-150	55	6.6	10.04
c34205.graph_c0	OR19	Y	389	AIG51856.1 | odorant receptor [*Helicoverpa armigera*]	402	7e-136	50	18.62	11.28
c39368.graph_c2	OR20	Y	386	JAV45828.1 | olfactory receptor 37 [*Mythimna separata*]	522	0.0	66	9.04	4.63
c32622.graph_c0	OR21	Y	380	JAI18048.1 | Odorant Receptor [*Epiphyas postvittana*]	459	1e-158	59	3.65	0.71
c37029.graph_c1	OR22	Y	377	ACJ12370.1 | olfactory receptor 13 [*Helicoverpa armigera*]	335	3e-109	48	3.62	214.15
c37794.graph_c0	OR23	N	364	ALM26250.1 | odorant receptor 85 [*Athetis dissimilis*]	332	1e-108	46	6.52	3.87
c37397.graph_c0	OR24	Y	355	JAI18015.1 | Odorant Receptor [*Epiphyas postvittana*]	452	1e-155	60	16.24	4.61
c29168.graph_c0	OR25	Y	345	ALM26219.1 | odorant receptor 30 [*Athetis dissimilis*]	211	2e-61	34	25.21	0.05
c37849.graph_c0	OR26	Y	335	AQQ73504.1 | olfactory receptor 24 [*Heliconius melpomene* rosina]	323	2e-105	48	4.79	2.77
c27537.graph_c0	OR27	Y	328	AIG51873.1 | odorant receptor [*Helicoverpa armigera*]	442	9e-153	68	10.2	8.72
c36736.graph_c0	OR28	Y	328	AFL70813.1 | odorant receptor 50 [*Manduca sexta*]	319	6e-104	48	38.6	11.53
c35263.graph_c0	OR29	Y	323	AII01063.1 | odorant receptor [*Dendrolimus houi*]	324	3e-106	47	9.62	8.48
c36491.graph_c0	OR30	Y	312	AIT71991.1 | olfactory receptor 22 [*Ctenopseustis obliquana*]	218	7e-65	38	12.15	0
c31465.graph_c0	OR31	Y	310	CUQ99406.1 | Olfactory receptor 24 [*Manduca sexta*]	329	2e-108	55	6.02	3.87
c34557.graph_c0	OR32	Y	307	NP_001103476.1 | olfactory receptor 35 [*Bombyx mori*]	317	1e-103	49	3.63	2.07
c31339.graph_c0	OR33	Y	289	ALM26217.1 | odorant receptor 28 [*Athetis dissimilis*]	211	1e-62	42	2.42	1.11
c40086.graph_c1	OR34	N	266	KOB71190.1 | Olfactory receptor 29 [*Operophtera brumata*]	315	2e-103	66	5.58	4.12
c34853.graph_c0	OR35	Y	253	AFL70825.1 | odorant receptor 62 [*Manduca sexta*]	276	3e-88	52	20.93	5.42
c20903.graph_c0	OR36	N	240	ANZ03138.1 olfactory receptor 25 [*Cnaphalocrocis medinalis*]	266	4e-85	58	1.05	0.67
c34868.graph_c0	OR37	Y	229	AIT69888.1 | olfactory receptor 32 [*Ctenopseustis herana*]	161	4e-44	43	10.26	5.94
c39205.graph_c1	OR38	N	219	CUQ99411.1 | Olfactory receptor 30 [*Manduca sexta*]	349	1e-117	74	12.46	27.07
c37452.graph_c0	OR39	Y	217	ALM26195.1 | odorant receptor 7 [*Athetis dissimilis*]	229	1e-70	54	1.39	2.44
c39040.graph_c2	OR40	N	210	ALM26246.1 | odorant receptor 63 [*Athetis dissimilis*]	158	3e-43	39	21.26	5.26
c39852.graph_c1	OR41	Y	209	AIT69908.1 olfactory receptor 66 [*Ctenopseustis herana*]	253	3e-80	57	50.85	94.05
c27805.graph_c0	OR42	Y	206	AIT71985.1 | olfactory receptor 11 [*Ctenopseustis obliquana*]	200	1e-59	49	0.5	1.22
c21300.graph_c0	OR43	N	167	ANZ03138.1 | olfactory receptor 25 [*Cnaphalocrocis medinalis]*	124	6e-31	40	0.31	0.51
c36361.graph_c0	OR44	Y	116	AND95945.1 | olfactory receptor 12 [*Helicoverpa armigera*]	118	2e-29	50	3.03	2.24
c49028.graph_c0	OR45	N	102	ACF32962.1 | olfactory receptor 4 [*Helicoverpa armigera*]	163	2e-46	74	1.32	0
c38973.graph_c1	OR46	Y	77	KPJ10058.1 | Odorant receptor Or1 [*Papilio machaon*]	119	4e-31	70	18.12	17.1
c29912.graph_c0	OR47	Y	73	ALM26195.1 | odorant receptor 7 [*Athetis dissimilis*]	90.5	9e-20	61	0.37	0.84

**Fig 11:**
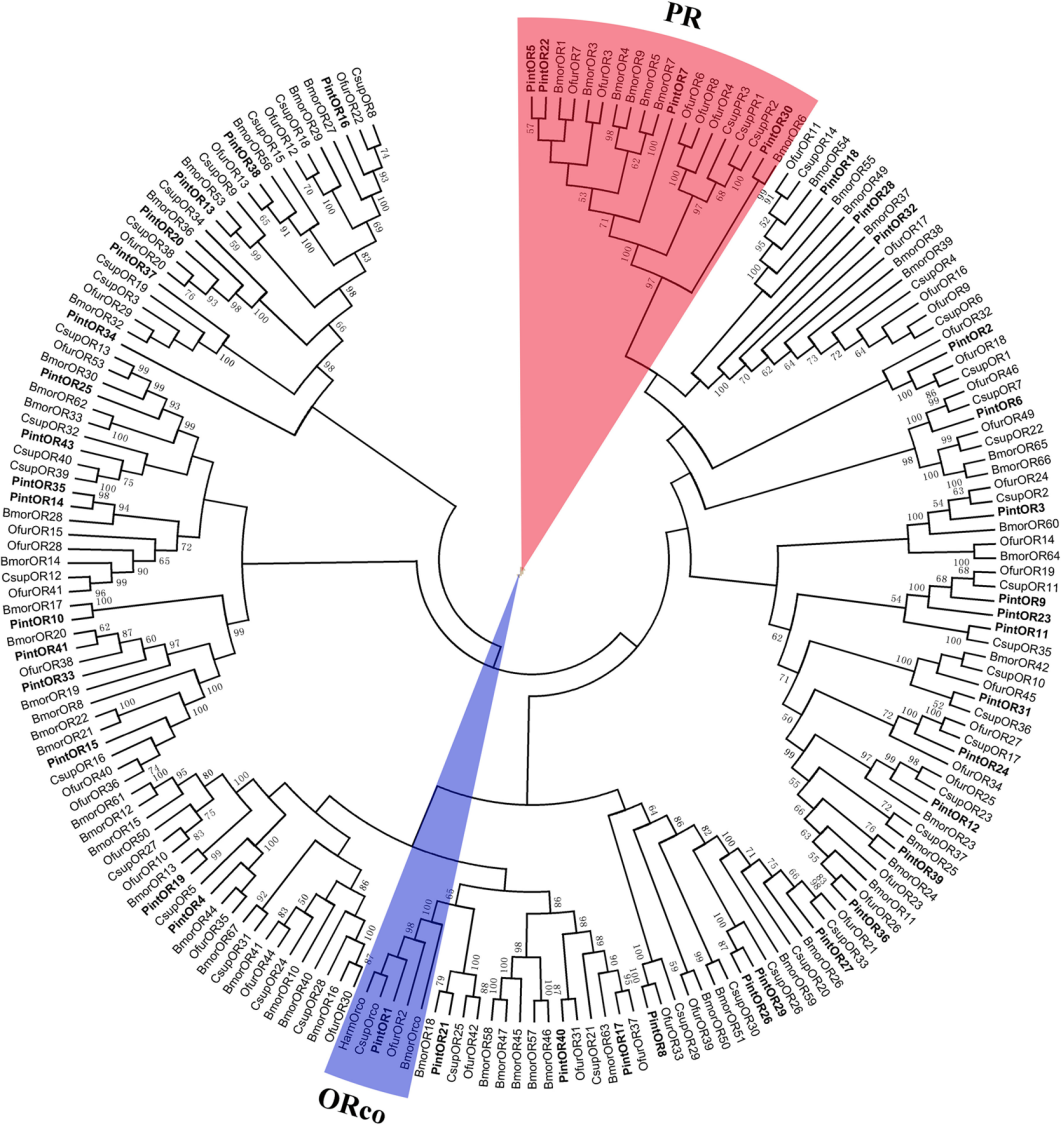
Neighbor-joining tree of candidate OR proteins from *Plodia interpunctella*, *Ostrinia furnacalis*, *Chilo suppressalis* and *Bombyx mori*. The protein names and sequences of ORs that were used in this analysis are listed in S4 Table.

In the neighbor-joining tree of ORs (Fig 11), the PintOR1 was clustered into the ORco family and four PintORs (PintOR5, PintOR7, PintOR22 and PintOR30) were clustered into the pheromone receptor (PR) family. Two groups of ORs (PintOR14 and PintOR35, PintOR29 and PintOR26) were clustered into the same branch with bootstrapping values of 98 and 87, respectively. All of the other PintORs were distributed on various branches throughout the phylogenetic tree.

**Fig 12:**
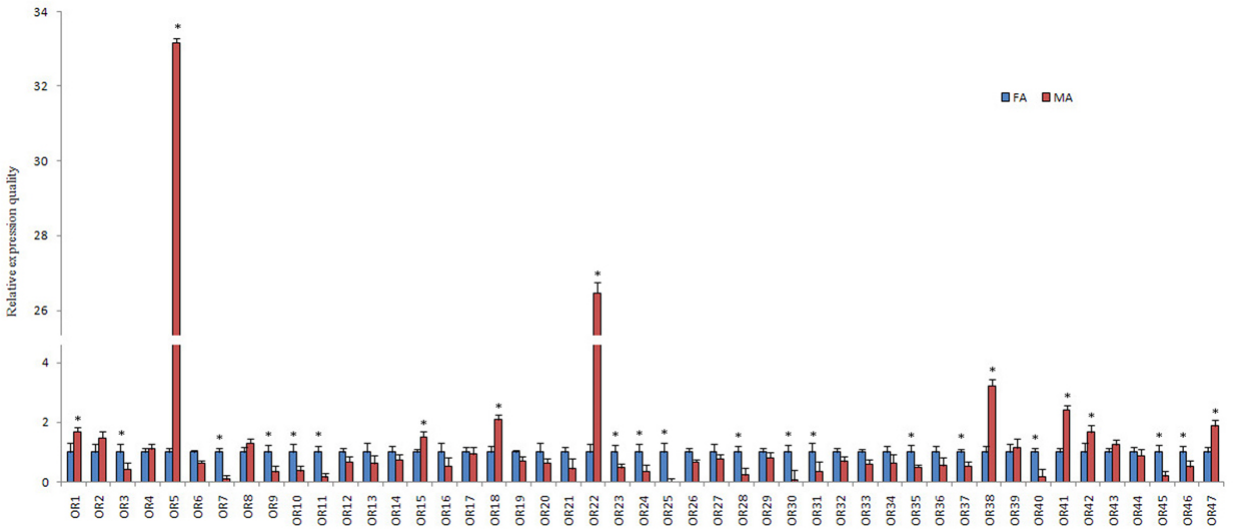
*P*. *interpunctella* OR transcript levels in different antennae measured by qRT-PCR. MA: male antennae; FA: female antennae. The internal control *β-actin* was used to normalize transcript levels in each sample. The standard error represented by the error bar, and the asterisk above each bar denote significant differences (*p*<0.05).

PintOR1 (ORco) showed the highest qRT-PCR expression level among the 47 PintORs, with FPKM values of 576.23 and 430.52 in the male and female antennae, respectively. However, the other 46 typical ORs showed a relatively lower expression level (FPKM ranged from 0 to 214). The qRT-PCR results indicated that nine OR genes (PintOR1, PintOR5, PintOR15, PintOR18, PintOR22, PintOR38, PintOR41-42 and PintOR47) were highly expressed in the male antennae. Meanwhile, 16 OR genes (PintOR3, PintOR7, PintOR9-11, PintOR23-25, PintOR28, PintOR30-31, PintOR35, PintOR37, PintOR40 and PintOR45-46) exhibited female antenna-specific expressions (Fig 12).

### Candidate GRs

In the present study, we identified nine candidate PintGR encoding transcripts from antennal transcriptome of *P*. *interpunctella*. Five PintGR genes had intact ORFs with lengths ranging from 198 bp to 1,461 bp. The BLASTx results indicated that seven identified PintGRs shared relatively higher amino acid identities (>50%) with Lepidoptera GRs in NCBI (Table 6).

**Table 6:** The Blastx matches of *Plodia interpunctella* candidate GR and IR genes.

Gene ID	Gene name	Full length	ORF (aa)	Blastx annotation (Reference/Name/Species)	Score	*E*-value	Identity (%)	FPKM values	
								Female	Male
c28105.graph_c0	GR1	N	509	AIG51909.1 | gustatory receptor [*Helicoverpa armigera*]	721	0.0	78	2.14	12.57
c36972.graph_c0	GR2	Y	487	JAI18129.1 | Gustatory Receptor [*Epiphyas postvittana*]	456	5e-155	55	11.23	10.57
c19072.graph_c0	GR3	N	410	AIG51907.1 | gustatory receptor [*Helicoverpa armigera*]	796	0.0	92	0.57	0.78
c37959.graph_c0	GR4	Y	401	ACD85125.1 | gustatory receptor 46 [*Bombyx mori]*	180	3e-49	32	33.37	29.39
c35051.graph_c1	GR5	Y	381	JAI18131.1 | Gustatory Receptor [*Epiphyas postvittana*]	183	4e-52	51	2.54	1.51
c37548.graph_c1	GR6	Y	297	ALS03938.1 | gustatory receptor 3 [*Ectropis obliqua*]	474	4e-165	76	7.56	4.78
c27749.graph_c0	GR7	N	254	AGK90011.1 | gustatory receptor 4 [*Helicoverpa armigera*]	81.6	4e-14	38	0.79	0.25
c69579.graph_c0	GR8	N	80	AJD81595.1 | gustatory receptor 2 [*Helicoverpa assulta*]	151	2e-43	97	0.27	0.54
c35211.graph_c0	GR9	Y	66	AGK90011.1 | gustatory receptor 4 [*Helicoverpa armigera*]	70.5	1e-12	57	18.78	13.56
c38794.graph_c1	IR1	Y	902	BAR64796.1 | ionotropic receptor [*Ostrinia furnacalis*]	1439	0.0	77	137.3	113.52
c37198.graph_c0	IR2	Y	863	BAR64797.1 | ionotropic receptor [*Ostrinia furnacalis*]	1256	0.0	70	12.95	13.76
c39892.graph_c1	IR3	Y	703	AOG12853.1 | ionotropic receptor [*Eogystia hippophaecolus*]	1071	0.0	75	8.91	5.81
c38347.graph_c0	IR4	Y	646	BAR64805.1 | ionotropic receptor [*Ostrinia furnacalis*]	808	0.0	66	8.16	5.18
c36292.graph_c0	IR5	N	581	BAR64811.1 | ionotropic receptor [*Ostrinia furnacalis*]	909	0.0	76	4.24	3.33
c40504.graph_c0	IR6	Y	550	JAP88619.1 | IRs [*Conogethes punctiferalis]*	803	0.0	70	22.37	21.95
c39043.graph_c0	IR7	N	518	AJD81639.1 | ionotropic receptor 75q.2 [*Helicoverpa assulta*]	525	0.0	67	16.37	17.41
c38844.graph_c0	IR8	Y	498	AOG12846.1 | ionotropic receptor [*Eogystia hippophaecolus]*	473	7e-159	61	18.83	16.13
c37486.graph_c0	IR9	Y	456	BAR64806.1 | ionotropic receptor [*Ostrinia furnacalis*]	509	1e-173	62	5.43	4.56
c37941.graph_c0	IR10	N	436	KOB72397.1 | Ionotropic receptor [*Operophtera brumata*]	459	4e-154	50	6.8	4.64
c39660.graph_c1	IR11	Y	351	JAV45789.1 | Ionotropic Receptor 7 [*Mythimna separata*]	364	2e-118	52	16.63	7.68
c35003.graph_c0	IR12	N	283	AIG51922.1 | ionotropic receptor [*Helicoverpa armigera*]	501	3e-177	82	1.32	1.45
c20608.graph_c0	IR13	N	218	JAI18093.1 | Ionotropic Receptor [*Epiphyas postvittana*]	213	7e-62	50	0.36	0.82
c36292.graph_c1	IR14	Y	128	BAR64811.1 | ionotropic receptor [*Ostrinia furnacalis*]	229	3e-68	80	4.07	3.32

**Fig 13:**
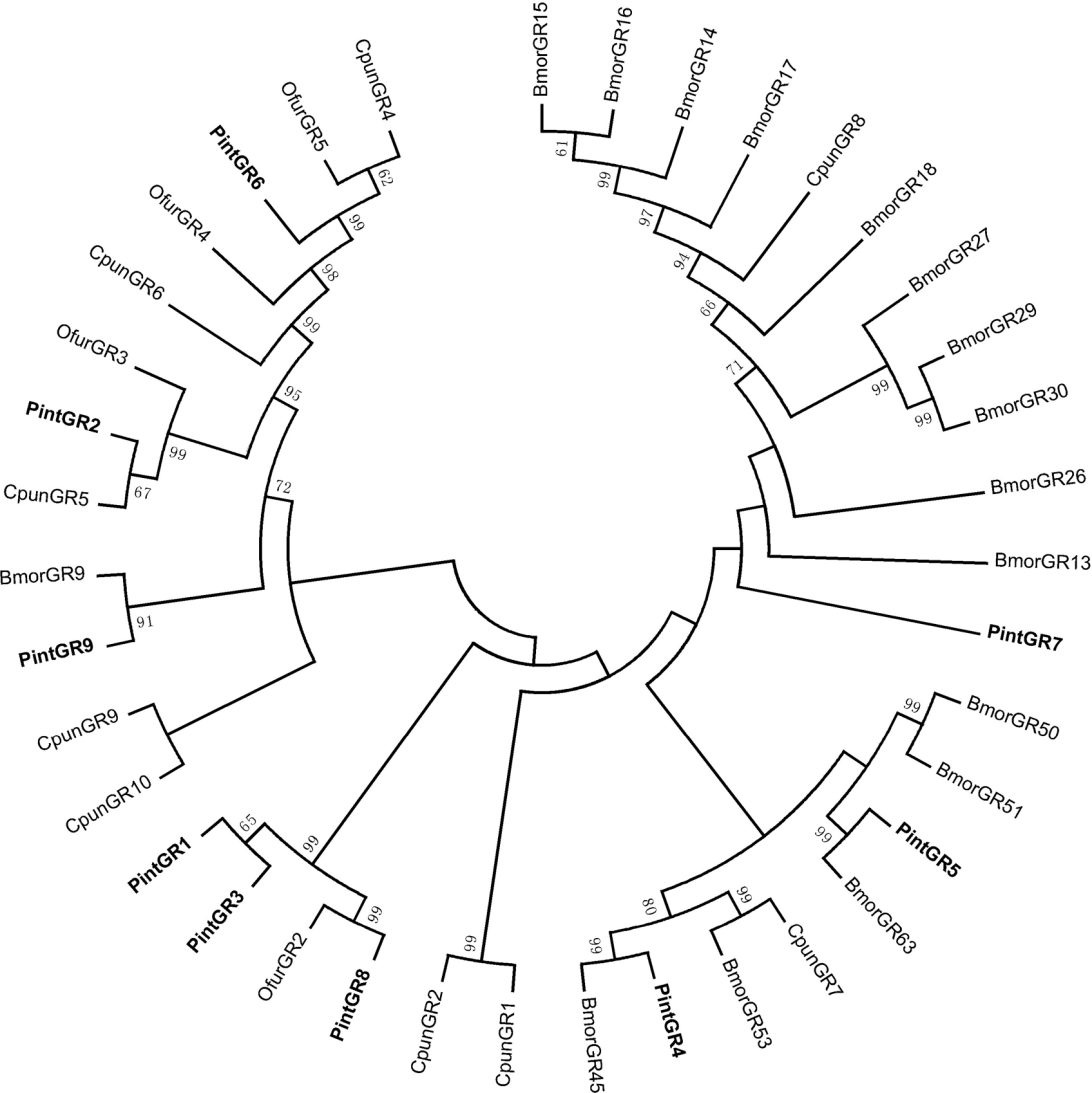
Neighbor-joining tree of candidate GR proteins from *Plodia interpunctella*, *Conogethes punctiferalis*, *Ostrinia furnacalis*, *Chilo suppressalis* and *Bombyx mori*. The protein names and sequences of GR that were used in this analysis are listed in S5Table.

In the neighbor-joining tree of GRs (Fig 13), PintGRs were present on various branches throughout the cladogram. PintGR1 and PintGR3 were clustered into the same branch, with a bootstrapping value of 65.

**Fig 14:**
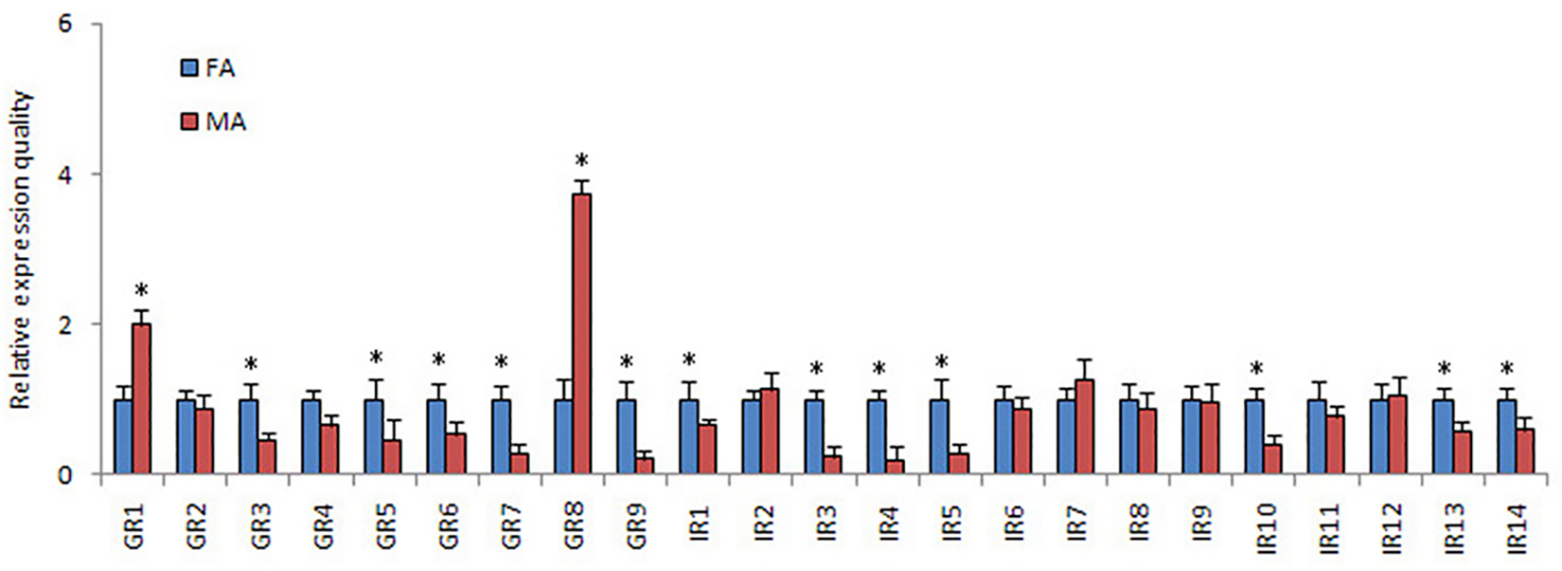
*P*. *interpunctella* GR and IR transcript levels in antennae measured by qRT-PCR. MA: male antennae; FA: female antennae. The internal control *β-actin* was used to normalize transcript levels in each sample. The standard error represented by the error bar, and the asterisk above each bar denote significant differences (*p*<0.05).

The FPKM analysis showed that all PintGRs had a relatively low expression level (FPKM ranged from 0.27 to 33.37). The qRT-PCR results indicated that PintGR1 and PintGR8 were highly expressed in the male antennae (1.9 and 3.7 times compared with females, respectively). Moreover, five GRs (PintGR3, PintGR5-7 and PintGR9) displayed female antenna-specific expressions (Fig 14.).

### Candidate IRs

**Fig 15:**
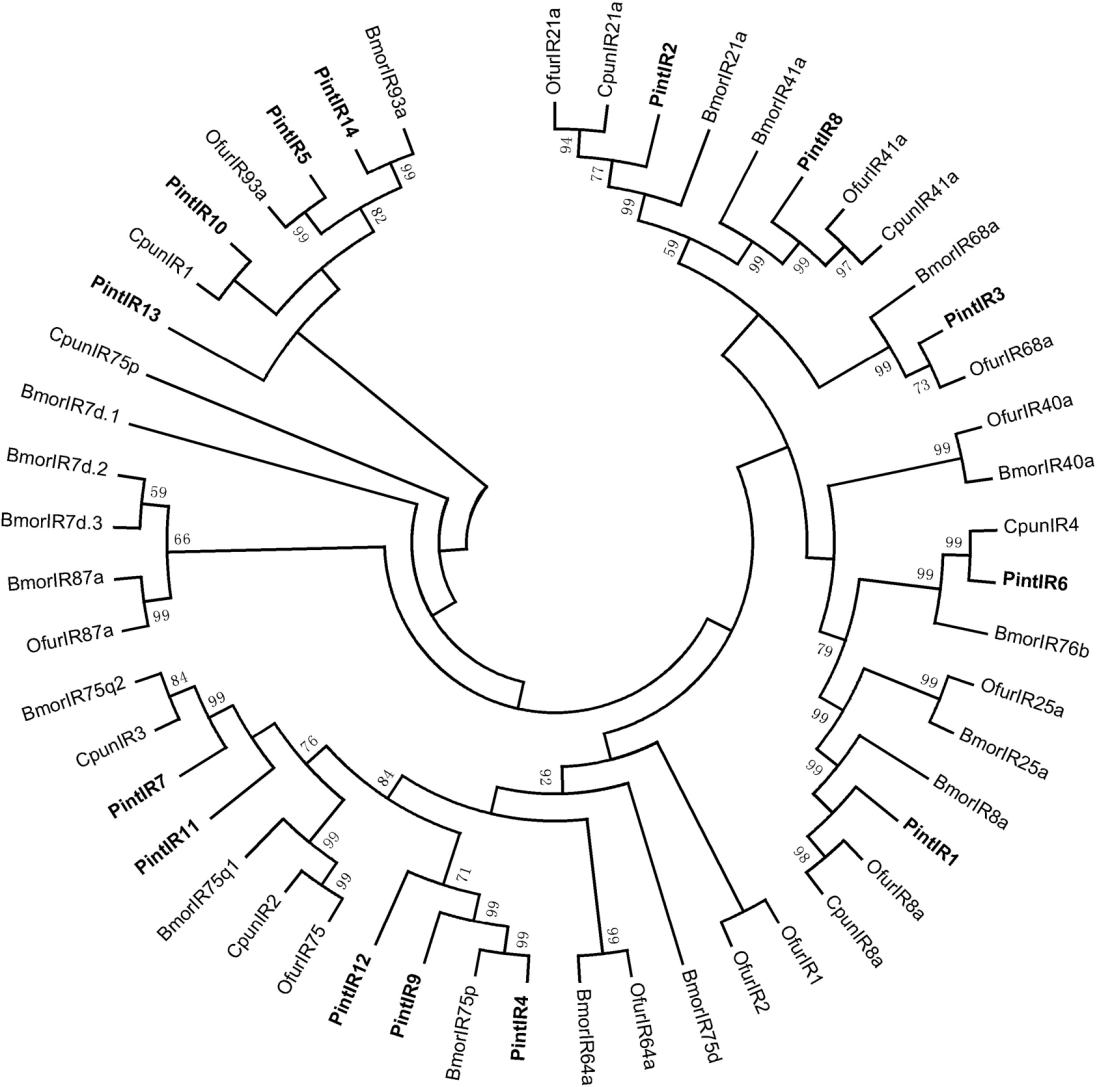
Neighbor-joining tree of candidate IR proteins from *Plodia interpunctella*, *Conogethes punctiferalis*, *Ostrinia furnacalis*, *Chilo suppressalis* and *Bombyx mori*. The protein names and sequences of IR that were used in this analysis are listed in S6 Table.

In the present study, we identified 14 candidate PintIR genes encoding transcripts from antennal transcriptome of *P*. *interpunctella* (Table 6). Nine PintIRs had intact ORFs with lengths ranging from 384 bp to 2,706 bp. In the neighbor-joining tree of IRs (Fig 15), PintIR1 and PintIR2 were phylogenetically clustered into the highly conserved IR8a and IR21a sub-families, respectively. The FPKM analysis revealed that all PintIRs showed a low expression level (FPKM value ranged from 0.36 to 113.52). The qRT-PCR results indicated that PintIR1, PintIR3-5, PintIR10, and PintIR13-14 were highly expressed in the female antennae (1.2 to 5.3 times compared with males) (Fig 14).

## Discussion

In recent years, RNA-Seq transcriptome sequencing technology has been widely used due to the development of high-throughput sequencing technology, resulting in great progress in non-model organisms [11, 37–39]. In the present study, we used NGS technology to analyze the antennal transcriptome of *P*. *interpunctella*. Sequence analysis and assembly results demonstrated that Illumina sequencing technology could effectively and rapidly captured a large portion of the transcriptome, providing molecular foundations for rapid characterization of functional genes and better reference of target genes [40].

The unigene annotation showed that 55,379 unigenes (63.44%) were unmapped in those databases, which could be attributed to the short sequence reads generated by the sequencing technology. It also suggested that the unmapped sequences could represent unannotated or new genes. In fact, fewer than 5% of unmapped unigenes are likely to represent new genes. Generally, the 5' ends of sequences show less conservation than the body. Therefore, partial transcripts (unigenes representing the 5' CDS, but not the body) may not be found matches in the databases. For GO analysis, the antennal unigenes were annotated into different functional groups [16], which were similar to those in the antennal transcriptomes of *Conogethes punctiferalis* [13], *Spodoptera littoralis* [41] and *Helicoverpa armigera* [11]. Therefore, we inferred that the success rates of functional annotation of genes highly depended on the sequence length of the splicing unigene: the shorter the length of the sequence, the less possibility of the annotation. Others reasons might also result in partial information failure, such as the incompleteness of *P*. *interpunctella* gene transcription group information, and/or the insufficiency of the sequence of partial RNA-Seq sequencing data in public database.

Olfactory-related genes might be used as potential targets for management programs of *P*. *interpunctella*. As the first step of odor detection [6], OBPs have attracted wide interests of researchers [13, 17, 42]. In the present study, we identified 29 PintOBP genes from antennal transcriptome of *P*. *interpunctella*. The number of identified PintOBPs was equivalent to that from *H*. *armigera* (26) [11], *Dendrolimus kikuchii* (27) [17] and *Agrotis ipsilon* (33) [21], and it was significantly greater than that from *Cnaphalocrocis medinalis* (12) [12], *C*. *punctiferalis* (14) [13], *Manduca sexta* (18) [43] and *S*. *exigua* (11) [44]. The small number of OBPs in above species could be attributed to that the actual number of OBPs was less in *P*. *interpunctella*, or there should be more OBPs that were not caught by the sequencing. Therefore, we speculated that the transcriptomic sequencing might not be strong enough to detect all the OBPs, especially for some OBPs with low expression levels in the antennae [45].

The OBP trees from five Lepidopteran species indicated that after a long history evolution, the Lepidopteran OBPs differentiated into several branches (Fig 5), which was consistent with previous reports [46]. In the evolutionary tree for GOBPs and PBPs, these two sub-families were clustered respectively, indicating that these genes might have the same ancestor gene and differentiate along sex isolation and speciation. The qRT-PCR results indicated that nine PintOBP genes (PintOBP4, PintOBP6, PintOBP9, PintOBP13, PintOBP17 PintOBP20, PintOBP22 and PintPBP2-3) were significantly expressed in the male antennae, suggesting that these OBPs played essential roles in the detection of sex pheromones. Eight PintOBPs (PintOBP5, PintOBP7, PintOBP12, PintOBP15-16, PintOBP18, PintPBP1 and PintGOBP1) were significantly expressed in the female antennae, revealing that these OBPs played important roles in the detection of general odorants, such as host plant volatiles [21].

CSPs represent a newly-discovered class of soluble carrier proteins with similar functions to OBPs in insect chemoreception [47]. CSPs have been found in insect chemosensory tissues and non-chemosensory organs, such as antennae [11], legs [48], labial palps [49], tarsi [50], brain [51], proboscis [52], pheromone gland [53–54] and wings [55]. We identified 15 putative CSP encoding transcripts, and found that six PintCSP genes were significantly expressed in the female antennae. These PintCSPs might play important roles in the detection of general odorants, such as host plant volatiles.

OR proteins are key players in insect olfaction [56]. We identified 47 PintOR genes in antennal transcriptome of *P*. *interpunctella*. The number of PintORs identified in this study was less than that identified from the antennal transcriptomes of *Bombyx mori* (72) [57], *C*. *punctiferalis* (62) [58] and *Ostrinia furnacalis* (56) [59]. However, the difference in identified OR gene numbers might be caused by sequencing methods and depth, or sample preparation. In the neighbor-joining tree of ORs, four PintORs (PintOR5, PintOR7, PintOR22 and PintOR30) were clustered into the PR family, indicating that parts or all of them contributed to sex pheromone detection. The qRT-PCR results indicated that PintOR5 and PintOR22 were highly expressed in the male antennae, suggesting they are highly related to sex pheromone. PintOR7 and PintOR30 specifically expressed in the female antennae. The expression profiles of these sequences showed that not all of them were male-specific [60]. Recent studies also showed that some PR genes are expressed in both sexes [54]. The OR tree showed that the PintORco (PintOR1) was highly conserved.

In recent years, 12 HarmIRs in *H*. *armigera* [11], 17 SlitIRs in *S*. *littoralis* [61] and 15 CpomIRs in *C*. *pomonella* [22] have been identified. In this study, we identified 14 PintIRs, including highly conserved IR co-receptors PintIR1 and PintIR2 (IR8a and IR21a) from antennal transcriptome of *P*. *interpunctella*. Therefore, we speculated that IRs were relatively highly conserved sequences, implying that IRs had conservative features.

Several recent reports simultaneously tested the qRT-PCR expression of olfactory-related genes in various tissues of insect, including bodies, heads, legs or abdomens [13–15, 20–21, 54]. In present study, we only focused on the qRT-PCR analysis of *P*. *interpunctella* antennae. To the best of our knowledge, *P*. *interpunctella* moths do not eat anything, suggesting they have no food demands, so location of mate partners and oviposition sites should be the main function of olfactory. While most olfactory genes related to recognition of pheromone and host volatiles distribute in insect antennae, therefore, we only compared the expression between female and male antennae of *P*. *interpunctella*, to verify the olfactory-related genes.

## Conclusion

In this study, we identified a few olfactory gene families in antennal transcriptome of *P*. *interpunctella*, including 29 PintOBPs, 15 PintCSPs, three PintSNMPs, 47 PintORs, nine PintGRs and 14 PintIRs. The identification of antennal olfactory-related proteins in *P*. *interpunctella* reinforced our knowledge on the molecular and cellular basis of insect chemoreception. More importantly, our data suggested that new methods could be developed to control this pest by interfering their olfactory perception.

## Supporting information

S1 Table Primers used for RT-qPCR. (DOCX)

S2 Table Amino acid sequences of PintOBPs used in phylogenetic analyses. (DOC)

S3 Table Amino acid sequences of PintCSPs used in phylogenetic analyses. (DOC)

S4 Table Amino acid sequences of PintORs used in phylogenetic analyses. (DOC)

S5 Table Amino acid sequences of PintGRs used in phylogenetic analyses. (DOC)

S6 Table Amino acid sequences of PintIRs used in phylogenetic analyses. (DOC)
