# Supplementary material for: Identification of chemosensory genes from the antennal transcriptome of Indian meal moth Plodia interpunctella
Source: PLoS One. 2018 Jan 5;13(1):e0189889. doi: 10.1371/journal.pone.0189889 (PMC5755773; doi:10.1371/journal.pone.0189889)
Supplement: S1 Table — (DOCX) [file pone.0189889.s001.docx]

S1 Table. Primers used for RT-qPCR.

| **Gene Name** | **Forward (5'-3')** | **Reverse (3'-5')** |
| --- | --- | --- |
| PintPBP1 | GAATCCAACGGCGACTACTG | TCCTCAGCCATGATCTCCTC |
| PintPBP2 | CTCGGAGAAGTTGGAAATGG | GGATCAGGTCCACCAGTTGT |
| PintPBP3 | GGATGACCATGACATCACCA | TTGTGGTGCAGCTTGAAGTC |
| PintPBP4 | TGAAACACGGAGCTGATGAC | TGGCAATGTCTAGCACGAAG |
| PintOBP1 | CAGGAGGGAGAGACACAAGG | TCTGCTAGAGTTCCGGTCGT |
| PintOBP2 | AAAGAACTGCGGGTCGAGTA | GGTCTTCATCTGCACGGAGT |
| PintOBP3 | ACCTACTTGCTTGCGATGGT | GCGGTGGCTACTTCATCTTC |
| PintOBP4 | GATGGCACCATTCTCAAACA | TCTGTTGATGCGTTCGATGT |
| PintOBP5 | TCAACGACCTCCTTCGTTTC | CTTCTGGGAAGATTGGTGGA |
| PintOBP6 | GGACAGTATCGAGGGCATGT | CTTTCTCACGGCTTCCTCAG |
| PintOBP7 | AGCTCCAGAAGACGGCTGTA | CTATTTCCTCGGGAGTGTCG |
| PintOBP8 | TGGCAGAGTGTAACGAAACG | CTTCTCCAACACGCATCGTA |
| PintOBP9 | TGAAGTGCACCAAAGACCAC | TGTCATAACGCCGTCCAGT |
| PintOBP10 | TTGAGAAGTCAGCGAGGAAAG | TTTGTTGCATTTGTGCATCC |
| PintOBP11 | GCAGAGATTGCAGACGAGTG | GATCTGGAGTGCGTCCTTGT |
| PintOBP12 | TATGCTATGACGAGGCAGCA | TCGGCGATCCTCTATGAACT |
| PintOBP13 | ACGAAGCGGCTATCAAACAG | AAGCCTCGCACAAGTCCTTA |
| PintOBP14 | GGGCTGTTTGATGACAAAGG | TTGGCGCAAGTGTTGATTAG |
| PintOBP15 | GTACTCCGAAAGAGCGTCCA | GAAATCGGGATCCTTGTCGT |
| PintOBP16 | TGAAAGAACCCACGAAGGAG | ATATCGCCGGGTTGTGATTA |
| PintOBP17 | TCAAGTGCATCATGGAGACG | TAAGCATTGTCGCAGTGGTC |
| PintOBP18 | ATCCCGACGTAGAGTCCGTA | CCGGCATATCGTCTTTGAAT |
| PintOBP19 | GCATGCTGGTGAAGTCAGAA | GGCTAGGCAAGCGTCAATTA |
| PintOBP20 | AGCAGCTCAGAGAAGGTTCG | TCTTCACACCGTCCACTGAC |
| PintOBP21 | AGTGTACAGGCGGAGGAAGA | CGCTTGTTGTGAATCATCAGA |
| PintOBP22 | TCAGGACAGTGCCAAATCAA | CGCATCCTTTATCACCATCC |
| PintOBP23 | CAAGAAATGGTTCATGCAGGT | GCAAGCCAACAGACACTTGA |
| PintOBP24 | CTTCCAGGGAGTGCCTCAT | ATGCACTCGAAGATGTCGAAC |
| PintGOBP1 | CCTCGGACAAGATGGAAGAG | AAGCGTCAGTGAGGAGGTTG |
| PintCSP1 | AGCAACAGCAAACGATGATG | CGTTGACGAAACACTTGACG |
| PintCSP2 | AAGCTCAACGTGGAACAGGT | TCTTCAAATCCCTGGACTGC |
| PintCSP3 | GATTGTTGACGCCCTACCTC | ACAGGTGTGCACTTTGAGCA |
| PintCSP4 | TTAATGGTGGTCCTGGCTCT | ATGAGGCAGTCGACGTATCC |
| PintCSP5 | TATCAACTGCCTGCTGGATG | CTTCCCGTTGTCTTTCCGTA |
| PintCSP6 | ACAAGTGCGTCATGGACAAG | AAGAGCTTCCTCACGACGAT |
| PintCSP7 | ATTTCAGAGCCGACTTGCAC | GGTACTGGTCGGGCAACTT |
| PintCSP8 | CTGCTGATCCCGTACATCAA | ACATTTCGAGCATTCCGTCT |
| PintCSP9 | CGAGCTACTCCTCAGGTGGT | CACTTGAGTTGCCTTTGCAC |
| PintCSP10 | AAGCCTTGGAGAATGACTGC | TCGTACTTGGCCTTCAGCTT |
| PintCSP11 | GACGCGAGAAGGTACAGCTC | AAATAGCAAGCGGCTGACTC |
| PintCSP12 | GAAGCAGAGAGTGCTGGTGA | GCGTCAAATTGTGCCTTGTA |
| PintCSP13 | GACAACTTCGACGTGGACAA | TTCAATTCTCGGCCTTCATT |
| PintCSP14 | CCATGCAGAGACACCTCAAA | TCTCATGCGGAGTACATTGC |
| PintCSP15 | GATTGGGAAGAGGCTCAAGA | TGCGGGTAGTTTCTCTGGAC |
| PintOR1 | TCGGCAACCGACTCATAGAA | TCTGACATTGTTGGCACACG |
| PintOR2 | TTGTGCACGCTGTTGTATGC | AAACGCGGTCTGCCTGTAAT |
| PintOR3 | GACGCAACTTCTCTCCAGCA | CATGGCGATGAAGAAGGCTA |
| PintOR4 | TAAGCCGACAGAGGCAGAGC | CAAGACAGCGCCGACACATA |
| PintOR5 | TGAGCTACGCTTTCCACCAA | TGGGCCAGCAGTTGAAGTAA |
| PintOR6 | TCGGCAGAGAAACTCGACAA | ATTTCCCAGCAAGCGTCACT |
| PintOR7 | CGATGTTGAGTGCGACGATT | GCGTAATATGCTGCGTGAGC |
| PintOR8 | CTGTGCTGCATTGCCTATCC | CCAGCTCGATGACCAACTCA |
| PintOR9 | CGTGGCCTCTCAGTACGTGT | GGAGTCGCACTTCATCATCC |
| PintOR10 | GACTCCCTCGCCATATGCTC | CCTCACGCTCCGTTATACCC |
| PintOR11 | GCTCGTTGGTGGTGGACAGT | TACGTCCAGTCGGCGGAATA |
| PintOR12 | CCAGCAGCTACAATGGCGTA | AGGTACACAAGCGGCCATCT |
| PintOR13 | TGACGTCCTTCTGGAGAGCA | GGCCAGCGTTGAATATCACA |
| PintOR14 | AGGCTTGTTGGACGGTCATC | AACGGTTCCAGAGATCAGTCG |
| PintOR15 | GTTGCCGCATTGTTCTTCCT | CCGCAATGGAATATCGCTTC |
| PintOR16 | ATTGGAATCTGGTGGCTGGT | CTATCGAATGGAGCCCATGA |
| PintOR17 | TCTCGTTCATGGTGCTGCTT | TGAGCCAGGTTGGTGAACAG |
| PintOR18 | GTGGCGGTACCATTCTGGAT | ACAAGATCGCCAACGTTCTG |
| PintOR19 | CCGCTTCCACGGCATATTA | CCATACCAGCATGGAACAGC |
| PintOR20 | GCGAGTGTCCAGAAGGAGGT | CTGTAACTGGCGCGGAGAAT |
| PintOR21 | TGGTTGGTCTCAGCCAGAAA | TGCCGCGTGTGCTATATTCT |
| PintOR22 | CCCTGCCCTTTGACTACCAG | ACAACCAGAGCGACGATCAA |
| PintOR23 | TTGTTCTTTGCCACCGTCAC | ACCATCATCACCACCACAGC |
| PintOR24 | ACGCCAATGACTTGGAGGAT | TGCTCATCTCCATCCACGAT |
| PintOR25 | TTCGTGTAGTGCAGGGTATCG | ACCCAATAGGCGCTCACTGT |
| PintOR26 | TATGGCTGCCGTTTGATGTC | AACCGTTGAGTTGCCCAGAG |
| PintOR27 | TTCGCGTCGATGACTCTGTT | TCGACTCCATCAGCTTGTCG |
| PintOR28 | ACGTCCTCCTGCTGACCATT | TGGTCCTGGATTGCTCTTGA |
| PintOR29 | CGCTAACACAGCATCACTGC | CGCGTATTCCACTAGCCTGTT |
| PintOR30 | AGACGCGCTTATGTGGTTCA | ATGTGTGGTCGAAAGGCATC |
| PintOR31 | GGCTTTCCGAAGAACTGGAA | AGGGCGCAAATTTCAACG |
| PintOR32 | GAGGCACTTGATGGAGGATACC | GCGACGATGAAGAATGCTGA |
| PintOR33 | GAAGAACGACGCCACATTCA | CGCCAGCATCATACTCCAGA |
| PintOR34 | AACGGTTCTCCAAGCAGAGG | AGACTTCTGGCCTGCATTGA |
| PintOR35 | AATGGACGCGGATTATCAGG | CGGTATCACTAACCAGACCAGTTC |
| PintOR36 | CTTGTGATCTGCCTCACTGGA | CGTCGCTCACATCGCTACTT |
| PintOR37 | TGTTGGAGTGGCTTCGATCA | CAGCTGCAGATCGGTCAAAG |
| PintOR38 | GGAGTGGCCCAACTGTTCAT | CTTTGCGGTGAACTCTGTGC |
| PintOR39 | CGCTGTACAACTTCGTCACCA | TGCACCAAACACATGCTGAG |
| PintOR40 | TGTTCTCCAACCTGGTGCTG | TCTCTCAGCCTTCGGTCCAT |
| PintOR41 | GAACGCTGGAGACGTGACTG | CTGGCACCTGAGCTTGTGTC |
| PintOR42 | AGTCACTACTCGACGGCTTGTT | CTTCCGCGAACCAGCTTAC |
| PintOR43 | CCATGTCGTCTAGTTGGACTCA | TGCACCAGGAGCTACATAAGTG |
| PintOR44 | TAAGGAAATTCGCGGCAAGC | GACGTACACCACGTCCTTGA |
| PintOR45 | TCGTCGAGCCGTGTCACTAT | CATGAGCTGCCTGGAGAACA |
| PintOR46 | AGCCAAGAATTACGCGACGA | GGCGAAGCTGAACCGTCTT |
| PintOR47 | TCCAGTGGCATCAACAGCTA | CGGTGAGGCAGATGACAATAG |
| PintIR1 | CGTCATCGTCGGAAGCAGTA | GACGTCCACGTTGGTTGTGT |
| PintIR2 | AATTCAAGTGCCGAGTCACG | ACTCGACCGCCTCGATTCTA |
| PintIR3 | TGTTGCTCGTCGTGTCACTG | ATGGCGGTCATGCTAGCTCT |
| PintIR4 | TTACCATGAGTGCCGTGAGC | AGCTGAATATGCCGCGTACA |
| PintIR5 | GCTGGCTGTGTCAGCTATCG | CGAGGTCTCGCGATTATGAA |
| PintIR6 | ACCACGATACAGCCGGAGTT | GTCGCGTTGTGACAGAGGTC |
| PintIR7 | AAGCGCATACAAGAGCATGG | TGCCGTAGGAGAGTATCAGCA |
| PintIR8 | GTGGTTCAAAGGCCTCCACTT | CTCAGGTCGTTCTGAAGCACA |
| PintIR9 | CACGGTAGCACCAATGAGGA | AGTGCAAGCCAACGAACAGA |
| PintIR10 | GAACCGGTCATTGAAGCAGA | CCATCTGCGTGACCGAATAA |
| PintIR11 | CATACTCGGCCAGCATCGTA | TTCGATTCCGAGTTCCATCC |
| PintIR12 | AAGTGCGACCTGGTGGAGAT | CTGATGGATGTTGGCTTGGA |
| PintIR13 | CGAGTGACCGAGGCTTCTTC | CCACGTCACTGTCAACCACA |
| PintIR14 | AACCACACCGTCAACCTCAG | TTCCTCTTGCGTCGGTTGTA |
| PintGR1 | GAGAGCCACCGAGATGAACC | AACTGCAGCAACACCACCAG |
| PintGR2 | AGCAGTCGAATCTCTGGATCG | TAAGTGCTCCGCACATCCAC |
| PintGR3 | CAGCACTCAGCGAGAAGTGG | CTGCATCAGCACGATCAGGT |
| PintGR4 | GCACTGCTAAGTACAACTGCGG | GCAATGTGCCGTCGAGCTTA |
| PintGR5 | GTTGGAGCAAGTGGCATTCA | TCGTGTGCTGTGATCCGATT |
| PintGR6 | TTCGCGCCTACAGCATATCA | AATTGCGTGGCTCGACAGTA |
| PintGR7 | TGCTGTTCGACAGTGCGATT | TTGGCAATGAGGTTCAGCAC |
| PintGR8 | GTGTTGACTGTTGACCGACCA | CTCTGTTGACGTGCGCGTAT |
| PintGR9 | CAGCCGTCTGAAGGTGTTCT | GCAGCAGTATCACCAGGTACG |
