# Supplementary material for: Identification of chemosensory genes from the antennal transcriptome of Indian meal moth Plodia interpunctella
Source: PLoS One. 2018 Jan 5;13(1):e0189889. doi: 10.1371/journal.pone.0189889 (PMC5755773; doi:10.1371/journal.pone.0189889)
Supplement: S2 Table — (DOC) [file pone.0189889.s002.doc]

>OfurPBP1

MGLSLRLLVVVAAAIFGAESSQDVMKQMTINFGKALDTCRKELDLPDSINADFYNFWKEGYELSNRHTGCAIMCLSSKLDLVDPEGKLHHGNTHEFAKKHGADDSMAKQLVELIHKCEGSVADDPDACMKVLNIAKCFKAEIHKLNWAPSMDLIVAEVLAEV

>OfurPBP2

MWLSKTLVVIAVMCSMSVVVHSSQAVMKDMTKNFIKAYEVCAKEYNLPEAAGAEVMNFWKEGYVLTSREAGCAILCLSSKLNLLDPEGTLHRGNTVEFAKQHGSDDAMAHQLVDIVHACEKSVPPNEDNCLMALGISMCFKTEIHKLNWAPDHELLLEEMMAEMKQ

>OfurPBP3

MWLPKTLVVMSVMSSMSVVVHSSQTVMGEMTKNFIKAYEVCAKELNLSEATGLQLINFWKEGHELTTRETGCAILCMSTELNLLDVQGSVHRGNTVEFAKHHGSDDAMAHQVVDILHACEKATPNEDKCMLALSIAMCFKAEIHKLDWAPNNELMFEELVLDMWNS

>OfurPBP4

MADATKWRVAAILVICFTVNLNTVMSSEELMTKMGVTFFNVLEECKKELKVTTNINEGLVRFWSQGAAPERELGCVFLCMAHKKDLLEDQKRIHHENAHQFARGHGAEDDKATEIVSLLRECEQQFITITDDCLRALEVARCFQAHMQRLQWAPSMEVMVEEILAGMA

>OfurPBP5

MKGFAGVPVTLMLVLIGVSEIEMVPEAMKQLTGGFLKVLDQCKKELNLSDGVISDLYHLWKEEYDQISRDAGCVIHCMSQKLELLGGDGRMHHVNIKDFALKHGAGDEIATQLVTLAHECEKQKAAIEDDCERTLEMSKCFRSDVKQVDWTPKMEVIITEVIEV

>OfurGOBP2

MVCSGFYLGLVVMAAVTSVKGTAEVMSHVTAHFGKALEECRTESGLSPEILEEFQHFWSEDFEVVHRELGCALICMSNKLSLLQDDTRIHHVNMHDYVKGFPNGEVLSEKMVNLLHNCEKQFDDITDDCQRTVKVAACFKVDAKKEGIAPEVAMIEAVMERY

>CpunGOBP1

MVSDARLVVAACVVAAAGLVAGDHKIMTDVTLGFGQALEHCREESGLTEDKMEEFFHIWHQEFKFVDRELGCALHCMSKYFNLITDANKLHHENTDKFIRSFPNGEVLASKLVSLIHECEKQFEEVEDHCMRTLRIGECIRDSCLQRSLAPSMEMLLAEFIMQSE

>CpunGOBP2

MLPIWLYFGLVMAAVSSVKSTAEVMSHVTAHFGKALDECRDESGLSPEILEEFKHFWSEDFEVVHRELGCALICMSNKFSLLQEDTRIHHINMHDYVKSFPNGEVLSAKMVELLHNCEKQYDAITDDCDRTVKVAACFKNDCKKEGIAPEITMIEAVMERY

>CpunOBP1

MMKLNIFVSVAVLAAVLGNARALTKEELGVIESDMIAHVKKCGEQFGVSDEEIKAAKEKKDIDGIDPCLIGCVFKSTKLINDEGVFDPKVALEHSEKYLSSDDDKAKFKDIADDCAKVNDESVSDGKEGCERAKLLLSCFAKHKDELRPSRR

>CpunOBP2

MLLVLIAKFLMLLATCETMTMKQIRNTGKMMRKSCQPKNNATDEQLDPLNEGVFIDEKEVKCYMACIMRMANTMKNGKPNYDAAVKQADLLLPEEMKQPAKEALFACKKVPDDYKDPCDAAFHVTKCIFNHNPSIFFFP

>CpunOBP5

MISNYKYIQWFGLFGTCSWKSLKMIRAVTLFCGLFLMALTPNVDAMTEEQRAKIREHFETVGMQCIGDNPLSEEDITALRSKKAPSDSASCFLACMMKNVGVLDDSGMLQKETALELARKVFQDEEELQIISDYLHSCSPVNSAAVSDGAKGCERAMLAYKCMIENASKFGIDV

>CpunOBP6

MSWTGLAIIVTVLTVCHGKDTLELSDEIKEIIQHVHNMSWTGLAIIVTVLTVCHGKDTLELSDEIKEIIQHVHNECVGKTGVAEEDIRNCENGIFKDDKKLKCYMFCLMEEANLVDDDDNVDYDMLVSIIPEEYTDRTTKMIFSCRHLDTPDKDKCQRAFDVHKCSYGKDPEFYFLF

>CpunOBP3

MFDLSFAMTRQQLKNSGKLMKKSCMPKNDVTEEQIGDISSGKFIEERNVMCYIACVYTMTQVVKNNKISYDAVIKQVDMMFPPEIKDNVKAAAAHCKDVSKKYKDLCEASYWTAKCMYDFDSESFIFP

>CpunOBP4

MFKSGVCFVFIVSLLEFSMSLSDEKKAEILAKFIKVGEKCIIDYPLTKEEIAAFKEGKFPDSRGAACFSACILTKIGLMDDKGEISITAALERAKTIFKDEEELKIVEDFLNTCAKDGGTKGEDKCDRAKEIFICFIKKSKKFDL

>CpunABP

MFLGHFYISCLLLIIFDTYHVMSMTRQQMKNSGKILKKTCMPKNDVTEDEVGNIEKGKFIETKNVMCYIACIYSMGQVIKNNKIAHDAMFKQIDMVFPPEMKEPVKAAVEKCKPVAKKYKDICEAAYWTAKCIYDADPENFVFA

>CpunOBP7

MIHAVILFCGLLFVTWMPYSDAVTPEQRAKIGLHFHAVGMECIVDNPLAEEDVLAFRNRMPPPGPNGACFLACVLRRVGAMDDAGMMQRDSLLELARTVFHDEAELRSISDLLLSCSSVNSIEVSDGEKGCERAMLALKCMLEQASKANSLDEKNMIRDKFESVGEECIEKHPLSDEDIAALENKMPPPGRVGACFVACVMKNVGVMDDAGMLQKETALELAREAFDDEEELESIADFLHECSSSINSVAVSDGTEGCDRAILALKCMNEHESKFGLDL

>CpunPBP1

MGMLVKLLLVIVASVGVECSQDILKKMTVNFGKALEACKKEIDLPDSVNAELYNFWKEDYQLTNRQAGCALVCMSTKLDLVDPDGNMHHGNAHEYAKKHGADDATAKQLVEMLHTCEKSVGKMDDNCERALAIARCFKAEIHKLKWAPDPEVVLAEILAEV

>CpunOBP8

MDGRICLLLVFLVGGSDAMTRAQLKNSAKMLKKNCMAKNSVTEDQIGNIEKGQFIEEKPVMCYIACIYQMMSIVKNNKLNYEASIKQVDMMYPNDLKESVKKSIENCKSVSDKYKDICEASYWTAKCIYEDNPKDFIFA

>CpunPBP2

MAFKMWLKNIMVVATVVMMSVKVDSSQTMMKDMTKNFLKAYGECQQELHLTDDTARDLMFFWKEDYEVTSREAGCTILCLSKKLEIIDPEGKLHKGKTADFIKQHGSDEETAQKVIDVLHACEASAVPNEDHCIMALGVATCFKKEIHKLNWAPDTEVLLEELMAEMSER

>CpunPBP3

MSRVVSVTLLALLALSVGLSVGASTGTSGSSATTSGTTSNDLSRSTDDVKGKLITPADDQGNMTSSEERALDVPDLMAVMVECNDSFRIEMGYLESLNESGSFPDEIDRTPKCYVRCVLEKTGVASEDGLFDPAQAAAVFAGERNGVLMTNLEDLASRCAADRNEKCKCERSYNFIKCLMEAEIKEYVSN

>CpunPBP4

MKGFFVTLLVVFMGGKEVEMSSDGMKQLTTGFLKVLGACKTELGLSDGILSDMYHLWKEEYEQVSRDSGCMFSCMSKKLDILDGDGKIHHDHTKEYVLSNGGGEDLARQLINVAHDCEKQQESLEDECDRMLEIAKCLRRNIKEIQWTPKVEVIITEIVADM

>PintPBP1

KKKKKKKNVNKMAVTPKWRLLVCVLGLLCMQRVHSSKEVMHKMTASFSKVIDQCKSELNLSENIMQDLMNFWKEEYELLNRELGCVIMCMATKLDLVQVNEYKMHHANAHEFAKQHGADDEVAKQLVTMIHDCEKQFESNGDYCARTLEVAKCFRTKIHSLKWAPDMETVLEEIMAEDAKQ

>PintPBP2

NSTEEVNMKLLVQIIAAVVVLMAGVDSSADIMKELSINFGEALDTCKKELDLPDSINMDFYNFWKEDYEITNRLTGCAIKCLSEKLEMVDADGKLHHGNAHEFATKHGADDAMAKQLVDLIHGCEKSVPANDDACLVVLSIANCFKKEIHKLNWAPNMDLVVGEVLAEV

>PintPBP3

MKAIVSKMGLRVIVLASVVVTLAGVDSSADVMKELTINFAKALDQCKKELDLPDSINKDFNNFWKDDHDITNRLTGCAIWCLSSKLEMLDQDFKLHHKNTHEFAKKHGADDAMAQQLVDLIHGCENSVPANPDICLNTLGIAKCFKMEIHKLNWAPDMDLVIAEVLAEV

>PintPBP4

MYQLNLPDSVNTDIYNFWKEDYEVTDRLTGCAIKCLSEKLEIVDSEGELHHGNTEEFAMKHGADDEMAKQLVDLIDDCDNFVPDNEDVCIFVLDIANCFKKGIHNLNWTPNMDLAVGEVLADV

>PintGOBP1

MIKERQCGYNVLSHVCSSQTVLKMDTTRRRLVAILVGLLPALVGADVVVMKDVTLGFGEALEHCREENGLTSDKMEEFFHFWREDFKFEHRELGCAIHCMSRHFNLLTDASRMHHDNTHKFIKSFPNGEILASQMVELIHSCEKKFDHEEDHCWRILHVAECFKMSCQEKGLAPTMEMLLAEFIMEAEA

>PintOBP1

MCWKLYVFAITSLFLYCDALNCRSDGGPKEDELKTIYKNCLKRQEGRNSSDSRRYSNDQDWNEARGYGQRNWDRDSRTSSDNNRDDRMGDRNDRYGSDRTGSRDRMGSRDDIMSRDDRIGSSRDRSNSRDDRYGSRDDGMGGRDDRGNNGMGGRNRMGYQDDEVDRYRLSGRDDFSSDDYGSDLSRQNNYYSSTQSPRRFRRERHKEINSGHRSQYNPNAKKPSEYDDNYRNDDRNSSRNSSKDEEAKACALHCFLENLEMIGDNGMPDKYLVTHVLTKDVKNEDLRDFLQESIEECFQILDNENTEDKCEFAKNLMLCLSEKGRSNCDDWKDDLKI

>PintOBP2

MKQIITIYLSILLASCCARVDIEKYLKVCDRNSVDVNDCLVEAVQDGLEALANGIEDLGVPAVDPYHQKELRVEYKNNQIIAKMSMKDIFVYGLRGAKVHDTRLRADEDRFHMEIDMTSPKVFVTGQYEGEGRYNSLRINAHGTFNATMSDLVFTWKLDGIPETRDDKTYVRINSFYMRPDVGNIESYLTNNNPETRELTNLGNRFSNSNWRALYREFLPFAQDNWNKIGIRVANKIFLKVPYDQLFPANA

>PintOBP3

PGLLTYLLAMVGAALLLLLPVLVSSAGDGNIRLLEDEVATALKACVLPGEEASGGNRHRRSEDYPRIDNTKQTNQYSHERRNASDMREQNILVLNATDYDYGGYGSGNGGEKLVTSAPRLAGAVISNASADINGDYSSNRTRRSEPLINKPDADQCLSQCVFANLQVVDTRGIPRESELWNKVQASVTSQQSRNAMRDQIRACFQELQSEAEDNGCSYSNKLERCLMLRFADRKTDKSTSN

>PintOBP4

MKVNRARRPQEQPGAMAPFSNIVSVMLLLIFLSAFYLVISFKPLTKQDHIERINRMNQEVEPFRKNLSECARQVKASMADVEHFLKRIPQSTMQGKCLVACILKRNDIIKKNKVVPENLINANKAVYGEDDEVMKRLGLAIVECTGVVQGIFEICEYASLFNDCMHMKMEHILDKVMMERRMEALGQMTANNDDWTEEEDEILRLAKDEL

>PintOBP5

LSVGRWRSLHPRSPPYIMLRLVFVAATVLQTISSQRPPSFHPNIPEQCRRPPQESGNPDECCKIPPIFPEEHFKECGFEKSGKNHPPILRGPPDCSKQLCILRKNGLLNDEDKINSDEAKDFLDKWGDSNADLKAAIEVAKENCLDKEHLPGPPHVCEENKLFLCIKATLFNQCPDSPAWVKTSACSKLKDHMEDCAPFFAPPKK

>PintOBP6

MIYSVIKVFSIYFTCVHGLGHGHSSAGTMVDFTDPKVQGHLDALVRMAQSCVIRVRATPKDVRAYFTNSSPVSRSGQCFAACMLEQSDVITHGKVNRELLVHLASLVNGKNSRVVRKLSSISRLCLDSIEGMSDRCQLASTYNDCLNENMLEFAFPLEIAEEAVRKMPFHLIQANPFLPNAEFKPSSMTTSNVLQN

>PintOBP7

VAWAVDCSPVAATVAAPATAPPPLMTSLAWLLGFIAFVQSATPGCKNCIALGKEEKAMFRAHSEACLPQSQADPKVIEAMLNGELADDPALKKHVYCVLLKCKVISKDGKLQKTAVLGKMSTRTDGKNATKILENCSEQGGDTPEEIAWNLFRCGYDKKAVLFEYMPPEGAADLENNSQ

>PintOBP8

MIPPHPFEPIIIPRQHKMARAIFILALAAITFSLTQSGSLDTKSDKQSDGLSKMMASKADMDSATMLTNAMAECNETYTIDKSYTQALNESGSFPDETERIPKCYIRCVLEKTGIATEDGVYDPSRTDMFIEVFRISYPGKKSFKDMAEQCSDRKETCKCDRAYQYIKCIIENDIKNNM

>PintOBP9

PEILHRTIISNLVSLNNNILGFNFIMVKVVLIILILGYVYAITEDEIKMEFTKLVMKCTKDHPVEMADMMSLQKLIPPTKPEVKCLLACAYKLDGVMTEKGLYSLDHGYKLAEKIKNGDEKRLENGKKMADICAKVNEVEVSDGNKGCERAGLIFKCTLDNAPKFGFKV

>PintOBP10

RGEIFFEKSARKVIAFSVLINLCKKMYIFTFIVILAVGCTNATKKLIRLPADKLYQIVPQIMKCADETQVNFDDLKYLGSEKEHRGDKTVGSFLSCSFKRTGYSDDRGNVNIDKFEELFPKDDKEAVREVVTECDALKIKNISEKLHKFITCFVRNSPVLIVL

>PintOBP11

MSLRLVLSLAFVAVAFSKVLALSDEEKAAIHGSVLKFVQECSTEYNISEEKLKEAKEKKSTEGIEPCAIGCVFKKAKFINDQGLYEPERAKEIGAKYVNAEEDRKKYAEIADECASVNDESVSDGDKGCERAKLLLACMAKHKDALQI

>PintOBP12

FFFFFFFSTMAFLNIRLCLFCFVFIIFLCFDSGYAMTRQQLKNSGKLMKKSCMPKNDVTEEEIGEIEQGKFIEDRRVMCYIACVYSMTQVIKNNKLIYEAMLKQIDMMFPPELKEPVRAAATHCKDISKKYKDVCEASYWTAKCI

>PintOBP13

MEAKVICFLMIGVIIGTVNCMTRAQLKNSGKMFRKNCLAKVKVEEELIADIEKGKFIEDKDVMCYIACVYQMTQIVKNNKLSYEAAIKQIDLMYPADMKESVKKSVDKCKDISKKYKDLCEASFYTAKCIYEDNPKDFIFA

>PintOBP14

MEYLAVLLALATTAMANDTAENVATLQAKFVTAGEACIEEYPISGEDITLFKSGEFPDSEHAGCFSACVLRNIGLFDDKGSLYQPDNLEKATEIFNEEKEIETIKELINTCAKVNEETVTDGEKGCERAKLLFHCFVENSK

>PintOBP15

MSTATLPVFLFLFAIGLGAKHKPVFSEEIKEIIQHVHNECVGKTGVAEEDIANCENGIFKDDEKLKCYMFCLLEEASLVDDDDSVDYDMLVSLIPEEYSERASKMIFGCKHLDTNDKNKCQRAFDVHKCSYDKDPDFYFLF

>PintOBP16

MLLDILKFLLFLAACEAMTMKQIKNTGKMMRKSCQPKNNVADEKIDPLNDGVFIDEPEVKCYMACIMKMANTIKNGKLNFDAALKQVDLLLPEDLKEPTKEAMTACRKVPDAYKDICDASFHVTKCIYNHNPAIFFFP

>PintOBP17

MTRLRVWVVVAALVAAAAGMDEEMAELAKMVHDSCGEETGADLAPVDKVNAGEDLATFSDPKLKCYIKCIMETAGMMTDGEVDVEAVLAMLPDDIRARNEAAMRGCGTQRGADHCDNAYLTHICWQKANKKDYFLI

>PintOBP18

IVILAVVALVQADDWSPKTVDDIKKIREECMKQVPSSDEEFQKRKENDYPDVESVRKYALCNSKGWGLYKEGKGFYPDRVAEQFKDDMPEDEIKAIVNDCDEKTKEETDDERCYHLLKCVMSTKLGDHIKDLVKR

>PintOBP19

MKTFIVFAVCLVLAQALTDEQKEKLKKHRTECLAETKPDEQLVNKLKTGDFKTENEPLKKYALCMLVKSELMTKDGKFKKDVALAKVPNAADKPNVEKLIDACLANKGSTPQQTAWNYVKCYHEKDPKHAIFL

>PintOBP20

MACAKEFPLTAAGLKQLREGSLPEDPVVKCLFACTYKKLGMMDEKGQLSVDGVKKISQKYLADTPVKMKKADEFTDACSSVNNVPVSDGEKGCERAALIFKCSVEKAPQFDFV

>PintOBP21

NDKKLDECFVVHVMFYICTIDKINIFLLSMICAHIIYFSENLISLVKKFFEKDHTMIKKLEKNLDRCIEISVQAEEECAMAAKLNDCTNDLMIHNKRKLTINF

>PintOBP22

MLPDNENARCFAACLFKKIGIMDDMGKLSAAGAQDSAKSIFKNNDEHLSKVAELIGECSSVNEEMTVDGDKGCDRAKMAFTCLTDHAPKFNFDIDF

>CsupGOBP1

MEAAKVIMAGLLVVGVVPSMRADMVVMKDITLGFGAALEHCREESGLTQENMEEFFDFWREDFKFEHRELGCALRCMSRYFNLITDTNRMHHENTENFIKSFPNGEKLSKVLVQVIHECEKKFDHEEDHCWRILHIGECFRDMCRSQNIAPDMEMLLAEFIMQAESDTNPVAL

>CsupGOBP2

MVCSGFYLGLVVMAAVTSVKGTAEVMSHVTAHFGKALDECREESGLSTEVLEEFKHFWSEDFEVVHRELGCALICMSNKLSLLHDDTRVHQVNMHDYVKSFPNGEVLSEMMVKLIKNCERQYDDIKDDCDRTVKVAACFKADAKKEGIAPEITMIEAVIEIY

>CsupPBP1

MLYKQMIILDEPQLNCLMVRDTMMLKLVVVMCLTMTVMVDSSQTVMKSMTKNFLKAYEVCAKEYSLKEGTAGILIGFWKDDFSTTSRDVGCAILCLSTKLDLIDPEGKLHHGKATEFAMQHGSGEEMAKKLVEILHNCEQTVTPNEDKCMRALDIAMCFKKELHTLGWAPDPELLFEELIAEMR

>CsupPBP2

MSLYMRIVVLALVYLFNGVESSQEIMKQLSLNFGKAYDSCKKELELPNEVDTDFFNFWKEDYQLTNRLTGCAIMCMSNKLDLLDPDGKMHHGNAREFAKKHGADDSMAQQLLDILHNCEKGASPGPDGDACVQVLEISKCFKVEIHKLNWAPSMDLIMAEVLADV

>CsupPBP3

MAASMKCCLFGILVCFNVMVSDVESSQELIKKMSISFLKVLQECKLELSVPEEVLQSLMTFWNQDTDLSHRELGCVILCVVSKLDLIELETYKLHPDNANEYVKKHGADDETASQIMNILRGCEIKNEAISDHCDRVREIAKCFHGHMHELKWAPNMEVIINELVATKAI

>CsupPBP4

MVQHIAIFALMILSAVSVREVEMVPEYFKSMSRSLLEVLKTCSTELEIKDGIMYEIYQLWKENYDGLSRETGCVLHCMSQKLDLFNVQGKFEHGNTKEFIMKHGADSSTATQLEEMVHICQHKVGEMADECLRVLEMAKCIRGNLTQINWNPNMEVAVEEIVAEA

>CsupOBP1

MTIVFVVLSLLPVLVRCSGDGNIRLLENEVEEALKSCTLLPDDSLKDNNARQRRSNEYTRIDFNDSTIGQNQYGHEKRNSTDMKEQMYVLNATYGNNDYEYGNAGIGNSNGEKFVSSAPRLAAGGDYNKTQMNANRTRRSEPLLNRPDTDQCLSQCIFANLQVVDSRGIPREAEFWNKVQSSVTSQQSRTALKDQTRACFQELQTEAEDNGCSYSNKLERCLMLRFADRKLTGAQQGQNRKT

>CsupOBP2

MVRKISALLCCFCVFGISLSDSAISTESEKRCRNPPTAPQKIERVITLCQDEIKLSILREALDVIKEEHTMPAEKRRNKREVPFTHDEKRIAGCLLQCVYRKVKAVDGYGFPTLEGLVGLYSDGVNERGYFMAVLEASRECLMRHHDHFSRTVPMDNGRNCDVSFDIFECISDRIGEYCGNTGL

>CsupOBP3

MIRQVSVILLAIGFQVISSQGPPPFPPNIPPQCRGPPQVTEKPHECCKIPPFFEDADFEECGFKKADDEHPHERHGPPDCSKQLCMLKKYDLAKEDNIDFEALAKFMDKWVEAHPDFKSSVDAAKERCIGKPLPGPPYICEANKIVFCVSSTLIEFCPKWEATDGCQKLKSHIEECAPLFNRKQ

>CsupOBP4

MTWLLALGLLAVIGDVHPATTGCKNCITLGKEEKAMFRAHSDACLPQSKVDPKLVEGMLSGELTDDPRLRKHVYCVLLKCKVIGKDGKLQKTAVLGKMTNRTDGRNATKVLDSCSDQSGDTPEDLAWNIFRCGYDKKAVLFEYMPTSIAATDVENN

>CsupOBP5

MFRSTVLLCSLYFLALTPYLARAATEEQKAKIKEELEKLGAECMADFPITEDDINDFKSKKIPAGDGVPCFVACMMKKMGVLDEAGMMQKETALELAKSVFHDEEELKIIGDYLHSCASVNTEAVSDGAKGCERAMLALKCMYTNAPKFGFEL

>CsupOBP6

MAKFVVLCLGLLAAALSVKALTKEELDHIKEATLMHFNECNKDFNVSEDDIKAAETQKNMDKIDACLIGCMMKRSHLLDGEGKFDTEKAIELSKSFMKSEDDQKKFAEVVAECAKVNDEPVSDGANGCERSKMVLVCLAKHKAEFVPARR

>CsupOBP7

MLTVSKISFLVLAFLYYVKADSLEDLKKEYTATLVECMKKYEISPADIVQLQEKKMPDNENAKCMVACAYKASGMMDDNGMLSVEGVKKISEKYLSDNPEKMQNAFKFADACKSVNDQQVNDGNKGCERAALIFKCSLEQAAVFNFE

>CsupOBP8

MMEGKKGFHVRFAVLVVLFFIHLSYSMTRQQMKNSGKMMKKSCMPKNDVTEEQVGEIDQGKFLEEKNVMCYIACIYQMTQVVKNNKLNYDAVIKQVDMMFPAELKEPVKAAAAHCKDVGKKYKDICEASYWTAKCMYDFDPKSFVFP

>CsupOBP9

MASFHFKVNCFLYFVLLSSYFVYSMTRQQLKNSGKLLKKACIPKTNVSEEQIRDIDKGKFIEEKNFMCYIACIYTMSQAIKNNKIQHDAMIKQVETMFPNDIKESAKFAIQQCRGIAKQHKDICEAAFWTTKCLYDVDPATFIFP

>CsupOBP10

STEHYPAHHVINMKAFIVLAVCIVAAQALTDEQKEKLKKHKSECLAETKVDEQLVNKLKAGDYKSDNEALKKYALCMLIKSELMTKEGKFKKDVALAKVANPADKPQVEKLIDTCLANKGNTPHQTAWNYVKCYHEKDPKHAIFL

>CsupOBP11

MRCCAVLFVLAFIGCIYAEQEIVHLPPEKVAQILPVAMQCVGESSVPPEVIFQYASGKSLGNDKKYQKFIHCVFTKTGYADETGHINIDKAMEVFPKGTDKEAVKKIMEECSKERGEDPPETSFKFAKCFRKKAPVRITL

>CsupOBP12

MLLIIIAKFLVLVAICEAMTMKQIKNTGKMLRKTCQPKNNAADEKIDPLNEGVFIDEKEVKCYIACIMKMANTMKNGRPNIEVAMKQADLLLPEELKEPAKEALTACRKVPDAHKDVCDAAFHLTQCVYNQNPDIFYFP

>CsupOBP13

MKSIVFLCLVVAAAGMDMHHGVHLSESQKEKANQYIMECVKQSGVHTDVLVNAKKGQYADDEALKKFTLCFFQKSGIVDQTGKLNVDAALSKLPESVNKHDATKLLEECKNKTGKDAADTAFEIFKCYSKGTKTHILL

>CsupOBP14

MGFSRAVLLAAFVAGAWAMDEEMAELAKMLHDNCGEETGADLSLVDKVNAGADLMPDPKLKCYLKCIMETAGMMTEGVVDVEAVLALLPDDMRAKNEQNLRGCGTQKGADDCDTAFLTQLCWQKANKADYFLI

>CsupOBP15

MLMYHYVQMTRAQLKKTMTVAKKQCVPKIGVSEDKINKIEEGVFIEDPKVMCFIACVYKSLQVIKNDRLDRDLITRQVDILYPNDMKAPVKKAIDKCFHVQDKYSDLCEAVFYGVLCMYKVDPLNFVFP

>CsupOBP16

MFLKLLMLTVLFCAIHAMTRQQLKNSGKMLKKNCMGKNQVTEDQIGSIEKGKFIEDKNVMCYIACIYQMTQVIKNNKLNYEASLKQVDIMYPAELKESAKKSIENCKHISSKYKDICEASY

>CsupOBP17

MNCSVIAIIFAFLSLTSAELELSDEIKEIIQHVHNECVAKTGVAEEDIKNCENGIFKEDEKLKCYMFCLMEEANLADDDGVVDYEMMVSIIPEQYTDRVTKMIFACRHLDTPD

>OfurOBP1

MKGALVCAVLTALAARAWGMDDEMAELAKMLHDNCGEETGVDLGLVDKVNAGADLMPDAKLKCYIKCVMETAGMMSAGEVDVEAVLAMLPDGLRSKIEAPMRACGTQRGADDCDTAFLTQVCWQKANKVDYFLI

>OfurOBP2

MTKGTDLFLLLLAFFISSSDAMTRQQLKNSGKMLRKNCLTKTGVAEDLISGIEKGKFIEDRNVMCYIACIYQMTQVVKNNKLNYEASIKQVDMMYPNDLKESVKKSIEKCKTVSDKYKDLCEASYWTAKCIYEDNPKDFIFA

>OfurOBP3

MAKFLVLCLGVLAAAISSTKALTSEELLKIEADMLVYVKDCADKFSVSDDDLKEAKEKENVDNISPCFLACVFKNANLINDKGLYDPEVVTGKLSDKYLSNDEDKAKMAEIAKDCTKVNDESVSDGAEGCERAKHLLVCFAKHKNALKGGR

>OfurOBP4

MLRVALVVSIFVLSLGGLYCTPVQTSTKPDDSSAKHDDRSAKPVDSSAKPDDSSVKTKEVMLSSGQDMSSSEDANNTVDLMTFMNQCNESFRTDMAYIDALMESGSFPDETDRTPKCYVRCVLEHVGVASEEGAFDAARASEVFAGERGGRAMTDVQDLAEACADRNESCKCERSYRFMRCLMEAEIKEYSSN

>OfurOBP5

MFRIATLLVLLCVGYLTAEKPVVHLDSEKMVEILPQVIQCVAETGVNIEALEKLRLGVPGEIKDPNLPKFAHCAFLKTGYSHENGRAKVDKVLKLFPAGDYKAALKKHVQECDKDGKDPVDTTYQFLKCLYTKSPVIVRF

>OfurOBP6

MRPFLFLCLVMAVAGMDGHNVQLSQAQKDKVHQYTMQCIKESGVKPDIISEAKKGHFTDDEGLKKFTLCFFQKSGIVDSHGKLNVEAALAKLPPGINKVDAMKLLEECKKKTGKDAADTAFEVFKCYSRGTKTHILV

>OfurOBP7

MVGFVVVALVAVFQVISCQEPPPFLANVPEQCRRPPEGVDRPHTCCKIPSFFKDEDLQECGFKKLEDEPERPGPPHGPPDCSKQLCVMKKYNLLKGESDVDHDATKEYLQKWIESNPEWKEPMDAAIERCIGQPLPGPPHICEANKMVVCISFHLFGKCVSWSDTEGCKKLKAHMDECAQYFPKH

>OfurOBP8

MAVKKSLLLCELLLVLILLLDTSFGMTRQQLKNSGKLMKKSCMPKNDVTEEQVGEIEQGKFIEDRNVMCYIACVYSMTQVVKNNKLSYEAVLKQVDMMFPPEMKDAVKAAATQCKDIAKKYKDICEASYWTAKCMYDFDAENFVFP

>OfurOBP9

MIRGLVLLCALYFLALTSYSVNAMTNEQRAVIREHFEKLGIECIGDNPITEQDINDLRAKNVPSGPAAPCFLACVLKKCGVMDDHGMLKSETALDLARKVFDDEEELKIIADYLHSCHTVNSEAVSDGEKGCERAISAYKCMKENAPKFGIDV

>OfurOBP10

MCIAVVILSLVPALVRCSGEGNIKLLEEDVTTAMKACAVPGDVAKDANQRQRRSEDYPRIDNGSNGQNLYAHERRNITDLRDQMYVLNATDYDYGGYGAGSEGEKFLSTVPRPAGRDYNSSYNENRTKRSEPLLNKPDTDQCLSQCIFANLQVVDSRGIPREAELWNKVQSSVTSQQSRAALRDQIRACFQELQSEAEDNGCSYSNKLERCLMLRFSDRLKSDASKTQSSNQKTTN

>OfurOBP11

MFFRQLHVNCLVLFILLCTSYVLSMTKQQLKNSGKILKKTCMPKCAVTEEEVGDIDKGKFIEENNVMCYIACVYTMGQAVKNNKIMHDAMLKQVDMLFPPEMKEPVKAAIEQCRPVAKKYKDICEASYWTAKCIYEADPDNFVFA

>OfurOBP12

MLLTQLAKFLVVLATCEAMTLKQIKNTGKMMRKSCQPKNNAADEKIDPLNEGIFIDEKEVKCYMACIMKMANTIKNGKPNYEAAIKQVDLLLPEDMKEPAKEALAACRKVPDAYKDTCDAAFHVTKCIYNHNPSIFFFP

>OfurOBP13

MTWIVAFGLLAVFGGVHSASTGCKNCIILGKEEKAMFRAHSDACLPQSEVDPKLVEAMLNGELTEDPALKRHVYCVLLKCKVVGKDGKLHKTAVLGKMAARADGKNATKVLEGCAEQSGDTPEDIAWKLFRCGYDKKAVLFEYMPTNIGSDIIENNS

>OfurOBP14

MDQFQFGEIKTGKMLRILGLFVVICAMCGADIPVAPPAPLRVYCGDMPANIITCGSIPQIIPHGIQSRCPGSNKCDVMKCVAKEMGWLDGSSINTAKLGKYLDDFAKEHPDWATAIAQAKSSCLVPKLPAQGYYVDCPAYDVTFCMLATFIRNVPPSQWSSSSDCAYARQYAGACAVCPDDCFAPAIPTGSCNSCRVLPRSP

>OfurOBP15

MNALRECSQEYTLSPEDLEELKNSKMPDSEKVKCYFACAYKRAGMMDGEGKFWGDNVRKMSLQQYGNDESVVQKINHFVDACNKVNEVQVSDGEKGCERAALMFKCSNEHASELGFI

>OfurOBP16

MFILISETKPIQHFLSNEIRPTTSDSSRYTVINHQPILFSFQSLSADQQAIIQSKLLTSGLSCIKDHPLTIAEIKVLKDKMLPDGKNAKCFSACLFKKIGVMDDMGKFNPDGVRKNAQQLFKNDDEHLMKVEEVITECSAVNDASTSDGNMGCDRAKLVFDCLTGYADKFGFNINF

>OfurOBP17

SVLADNDCMKYIQPHYLKECCKMGLPDPPAIHLHPIKECLSLPNEAPGYEQDICSTTKKGLASADGKLDKSKLKEHTEKFFESSPELSKAITENCIDRDISKIGPPDMCEFTRYRACVGVQLAMMCPEWSNEGECAGSKVAFAEC

>BmorOBP1

MWKLVVVLTVNLLQGALTDVYVMKDVTLGFGQALEQCREESQLTEEKMEEFFHFWNDDFKFEHRELGCAIQCMSRHFNLLTDSSRMHHENTDKFIKSFPNGEILSQKMIDMIHTCEKKFDSEPDHCWRILRVAECFKDACNKSGLAPSMELILAEFIMESEADK

>BmorOBP2

MFSFLILVFVASVADSVIGTAEVMSHVTAHFGKTLEECREESGLSVDILDEFKHFWSDDFDVVHRELGCAIICMSNKFSLMDDDVRMHHVNMDEYIKGFPNGQVLAEKMVKLIHNCEKQFDTETDDCTRVVKVAACFKKDSRKEGIAPEVAMIEAVIEKY

>BmorOBP3

MSIQGQIALALMVYMAVGSVDASQEVMKNLSLNFGKALDECKKEMTLTDAINEDFYNFWKEGYEIKNRETGCAIMCLSTKLNMLDPEGNLHHGNAMEFAKKHGADETMAQQLIDIVHGCEKSTPANDDKCIWTLGVATCFKAEIHKLNWAPSMDVAVGEILAEV

>BmorOBP4

MGLTETVLKDFYNFWIEDYEFTDRNTGCAILCMSKKLELMDGDYNLHHGKAHEFARKHGADETMAKQLVDLIHGCSQSVATMPDECERTLKVAKCFIAEIHKLKWAPDVELLMAEVLNEVSWKS

>BmorOBP6

MARYNIVVAVLVLGVVGARGSSEAMRHIATGFIRVLDECKQELGLTDHILTDMYHFWKLDYSMMTRETGCAIICMSKKLDLIDGDGKLHHGNAQAYALKHGAATEVAAKLVEVIHGCEKLHESIDDQCSRVLEVAKCFRTGVHELHWAPKLDVIVGEVMTEI

>BmorOBP8

MYTNFILIFYFGISIYDVRASSLDDLKMVYKNVIKECVGDYPITAADLKLIKARQIPNDDIKCVFACAYKKTGMMTEEGMLSVEGIKDMSQKYLSDNPEQLRKSKEFAEACSSVWL

>BmorOBP11

MSANSFVVLAFCALAVGVNALTEEQKAEITKSSLPLIAECSKEFSVNQGDIDAAKKLGDPSGLNSCFVGCFMKKAGIINASGLFDVAATIEKSKKYLTSEEDLKAFEKLTETCAPENDKPVSDSDKGCERAKLLLDCFVANKGSFSVFSL

>BmorOBP13

MLFSKAVTPEESKAFEAFAKPLIEQCQKDFGMDKESFAQKNLDEIDECLIACVVEKFGIDLWGF

>BmorOBP15

MTKQQIKNSGKILKKACISKNDVTEDQISDIDKGKFIEDKNVMCYIACVYSMSQVVKNNKFVHDAMVKQVDMMFPTEMRDAVKASIANCRGVAKNYKDICEASFWTAKCMYEFDPANFVFA

>BmorOBP17

MTRQQLKNSGKIMKKTCMPKNDVTEEEIGQIEQGKFLEQRNVMCYIACIYTVTQVVKNNKLSYDAVIKQVDVMFPAEMRPAVKAAAENCKDISKTFKDICEASYWTAKCMYDFDPKNFVFP

>BmorOBP18

MMRKSCQPKNNVDDEKINPINDGVFIEENEVKCYIACIMKMANTMKNGKLNFEAAMKQADLLLPDEMKEPTKEAIVACRKVADSYKDVCDASFHVTKCIYNHNPSVFFFP

>BmorOBP20

MAVHIFLILASYMALAAHGQLDDEIAELAAMVRENCADESSVDLNLVEKVNAGTDLATITDGKLKCYIKCTMETAGMMSDGVVDVEAVLSLLPDSLKTKNEASLKKCDTQKGSDDCDTAYLTQICWQAANKADYFL

>BmorOBP21

MPVGTFWREREEFSCSVDTDILELEDDKYNKHKQDKVKVRVYYEALCPDSKYFFVKNLAPVTEKLSEFLDVTLVPYGKATTKEINGKYIFSCQHGEEECYANKIHSCSIEAVTNMTKAVKFTTCMITDNNDADEALQRVNDDGTVDYEMFTSLIPEEYFDRATKMIFSCKELDTPDKDKCERAFEVHKCSYEKDPDVSIVTILNVREK

>BmorOBP22

MLKVFVVVVCTLGASQLCAALYTQKVAVSFPKDKTTIVVEAMKSCIAKTGANPNVIEVISSGKVSEDEKFKEFFYCACNDIGVVNPDGHIKVKECIELFPKETQPLVEPVIKNCDKEGVNKYDTLFKYLKCFQETSPVRVTLA

>BmorOBP23

MTSKVLLSCVVLAVLATTVLAEDSRKLVSFAPEVAKKLKVLIQECLNENGLGEDAIEVIRAGEYREDEPFQNLVYCAYKKFGALDENNRIISQVAAASFPKDIDVVTVIESCGKEDGNTPVEQVFKYFKCFQKNSPVRMQLY

>BmorOBP24

MLSMVHTDNKIYSRYAMADGDANFRNMMEELFPDYPQIITSLLDCYRESLHSVATTNTFAKCIQEKTQVKLLIH

>BmorOBP25

MKSVVLICLAFAVFNCGADNVHLNEDEREKANWYTAECGVETGVSTEVINAAKIGKYSKDKAFKKFVLCFFKKSAILNSDGTLNMVVALAKLPSGVNKSEAQSVLEQCKNKTGQDAADKAFAILQCFHKGTKTHILF

>BmorOBP26

MIIILSYSQNVHLAETQKEKAKQYTSECVRESGVSTEAINAAKIGKYSKDKAFKNFVLCFFKKSAIFNSDGTLNMDVALAKLPPGVNKSEAQSVLKQCKNKTGQGAADKAFEIFRCYYKGTKTHILF

>BmorOBP27

MKSVVLICLAFAVFNCGADNVHLTETQKEKAKQYTSECVKESGVSTEVINAAKTGQYSEDKAFKKFVLCFFNKSAILNSDGTLNMDVALAKLPPGVNKSEAQSVLEQCKDKTGQDAADKAFEIFQCYYKGTKTHILF

>BmorOBP29

MTGPAAAAVLLALLAAAGQILGKEERAMFRSHSDACLAQSRVEPRLLESMMNGELIDDAALRKHVYCVLLSCKMIGKDGKLLKAAILGKLAARPAGRDVTKVLEACAEQPGASPEDVAWNIFRCGYNRKAVLFDYMPAGGASSGNTENHP

>BmorOBP30

MREKENEVRALRAFQADCAEDVQVKPDLVVNLKSGDWQTEDVSLKKWALCVLMKLGLMTAQGVFKMNEAMSKIPDMNDKIIAEKLIDDCLSLQATTPHDAAWNYIKCHHQKDPEGNFSSLNIF

>BmorOBP31

MKTFIVFVVCVVLAQALTDEQKENLKKHRADCLSETKADEQLVNKLKTGDFKTENEPLKKYALCMLIKSQLMTKDGKFKKDVALAKVPNAEDKLKVEKLIDACLANKGNSPHQTAWNYVKCYHEKDPKHALFL

>BmorOBP32

MYPKNLYKYPLRIDRNDIPCIIHCVLKKFGIISNDGFINIKNYYRRVQAIHRYDPRILISDVGETCAQNINGMNLDHDVCKKAKVFNDCTQLYAISYREPEDW

>BmorOBP33

MYAHDKLSDMIADQCLNEMYPRSKRLEIEESDEPCIIFCVLKKFGIMSPTGVINLEAYRKRVQLPEQLAQRNSINDFGSACLESAEATQHKQDVCKKAKVFNECTHLYKILLK

>BmorOBP34

MAQSCVIKVRATPKDVRAYFTNSSPVSRSGQCFATCMLEQSDIINHGKVNRDLLVHLAGLVNGKNSRVVRKLNSVSRLCLDSISGMTDRCQLASTYNDCLNENMIEFAFPLDIAEEAVRKMPFHLIQPK

>BmorOBP36

MKSKTKRARENRQTANMAVSEISRILTFLTIVSFIYIVYSFKPLTKDEHIERYNKMNEDIEPFRKNLTECARQVKASMADVEKFLKRIPQSNMEGKCFVACILKRNSLIKNNKLSQENLLEVNRAVYGDDSEVMSRLKTAILECSKIVEDIFEICEYASVFNDCMHMKMEHILDKITMERRMEALGQMSSNPDEWSEEEDEMLKLVKDEL

>BmorOBP37

MFYPFRFTLLFYGLFVIYLVRAEPEKENHFTLALKKTLFSTARSCMSHVNANETDLEYLRKDPPFPDKAACIIKCLLEKINHDQTTECELGNEVVSCIFKYAPELHFKT

>BmorOBP38

MANLVLLLTFVLMTLSMARLKSTEAPKSKTALFNDQDNMGYEELDMEEIMSACNESFRIEYAYLESLNDSGSFPDETDKTPKCYIRCVLEKTEILSENGVLNPATAALVFAGERNGKPMSDLEEMAVACADRHEKCKCEKAYNFVKCLMYMEIDKYEKKN

>BmorOBP39

MIICIVVLSLTEALDVIKEEHTMPAERKRNKREVPFTHDEKRIAGCLLQCVYRKVKAVDGFGFPTLEGLVGLYSDGVNERGYFMAVLEASRECLMKNHDKFSRTTPMGNTTAMQSDLNNNCLTL

>BmorOBP40

MGLLWLFFIFNLALVQAEFGTPYLRKSRLCHQWSCINTKLGFPESLPPREQSAVVLSRILPDGAWRNLTDHILDVCYENRPRTYTNTCPGQGLLHCLMYQMIENCPEESLRKDDVCSPVSSLSGFNYMFSQSMYEDLEEHLPVEIRPEWFLRNTNLDPLDCCDMSEFIQPSWRTQCNFRLNWDNRNRLSIDISHGAATTQTPVPTTKPKALRDFMVVPQSCDKTTCVFKKLNIVSDKGVVDVKSFIKLLDKFTNSYPVWNSAKARVITTCLRKSLIAYDGGCELNNILACTFDVLSENCPLNGNNQTCKHSSRKDTVCQISSSKYRPKHRRDPCSTIPELVNTDILTECNISALSRIEFAPETPIKIKKYGLDISKYKCKGQSVSATCLMDKMEVLNKYTFMDYFKMKDKIRKFTATQPLWTIYNDGYLSAFTNMPMYKEYCSSPKKLLNVVDAMLMTCPESRRQNTQQCRKLFTELTNSIPANKQNLTEEMVNHFHRIFLANVSSPKTGHPKRRIHLKQHKNNPLYYAILNTKEAPRVALLDIPRTSVREPLIIKPVYLRQKNQNTIATPYISDNILRSSPFWLHEQIAAAHSNSTTPVSVARIVLNSTDKIPNLSPNSNVELVTP

>BmorOBP41

MLTILFLLPIVVGIYSCLGNPKIIQPEVSEKCNKPISECDKTRCIFKESGWAKNNVIDKKKVSDYFEQFAKDNPDWSAAVQNFKTTCLSDSLKPQGVDTNCPAYDIIHCALISFIKFASPSQWSTSEQCVYPRQYAGACPVCPERCFAPSVPNGSCNACLALLRTP

>BmorOBP42

MMGYACVFVILAVLQAISAEDPPGLPPFLKDAPEKCKSPPRVKNPNECCISEPFFKEADFIECGIEKPGSERGPPDCSKQNCLLKKYNLLKNDETPDIEAIKSLLDKYIEKNPSFKSSVEKAKECLREDLPGPPQICLANRMTLCIGTVLLMECPDEKWNTTDDCKAFKDHMTECQKYFPK

>BmorOBP43

MTAWGQKQVAQAAKATLKPISACCNIPELGNPEPLAECSNPKLPGPCKDIQCVFEKSGFLTENKTLIKEAYKTHLRQWAKEHEGWSVAVEKAISDCVDKDLRQYLEFPCSAYDVFTCTGIAMLKKCPNEHWTC

>BmorOBP44

MSRLVLFFTILVVLQEFIINLYFNFITEIDSCCVKKYPKLFDSEFITECYNTQRKANDKCERDMCVARKLNLLTEEDSINKDALLRFVEEGFKTEIDLVNAIKKKCFEEDISNIGKPEMCEVAKYKICITSRMAEDCPKWDSKGICSSAQQKVENFMKMLS
