# Supplementary material for: Identification of chemosensory genes from the antennal transcriptome of Indian meal moth Plodia interpunctella
Source: PLoS One. 2018 Jan 5;13(1):e0189889. doi: 10.1371/journal.pone.0189889 (PMC5755773; doi:10.1371/journal.pone.0189889)
Supplement: S3 Table — (DOC) [file pone.0189889.s003.doc]

>CsupCSP1

MQRFIIFLVLGMAAMCLAEETTYTDRFDNVDVDEIIANPRLLTAYIRCVLDQGRCTAEGKELKEHVKDAMQTACKKCTDKQKDGARKVVNHIRVNEPQDWEKLVYKYDPIGEYKPIYEPFLAGENLDKNKGTEPSEDQKPDLVEQVQAPANDDKKADLVEARKTESVEFKKTEVVDKSSG

LGFQEERHF

>CsupCSP2

MKLQAIILMLVYLRCTSSKEKPYSTKYDSVNLDEILSNERLTTNYINCLLDLGPCSPEAKELKKNVPDAIENDCEKCSDRQREGADQVLDFIIDHRPEDWQKLEIKYNSDGSYKKKYLARKQQQAQNKSDESVKKDGKSNQNSLDELIKSQ

>CsupCSP3

MMSFKYLIMLCLVAAAVARPEDKYTDKYDNLNLDEILENKRLLQAYVNCILDKGKCSPEGKELKEHLQEAIETGCVKCTESQEKGAYRVIEFLIKNELNIWKTMTAKFDPEGKWRKKYEDRAREHGIVVPE

>CsupCSP4

MKAIILIGLSVVVTIAWARPGNTYTDKWDHINVDEILESQRLQKGYVDCLLERGRCTPDGKALKETLPDALEHECSKCTEKQKTASDKVIRHLVNKRPDLWTELAAKYDPDNMYQQRYKDKIEEVKGKQ

>CsupCSP5

MNSLIVFCVVSLAALTIARPDGATYTDKYDNVDLDEILGNRRLMVPYIKCMLDQGKCAPDAKELKEHIKEALENECGKCTEAQKKGTRRVIGHLINHEADFWNELTAKYDPERKYTTKYEKELKEVKA

>CsupCSP6

MKSMTVFCMLAFVAVAYALPQQYTDKYDSVDLTEILSNRRLLVPYLNCILTTGKCSPDGKELRSHIKEALENYCAKCTETQKNGTRRVIGHLINNEADYWKKLVDKYDPERKYVVKYEEELKTVAA

>CsupCSP7

MKSAIVLCLVALAGIALARPEDKYTSRYDSINIPEILQNRRLLVPYVNCALEKGKCTPEGRELKSHIKEALETRCSKCTDAQIRGTRLVLGHLINHEPGYWNQLTNKYDSTGKYTKKYEQELRTQI

>CsupCSP8

MKLIVAVALLCLVAESWAASTYTDKWDNINVDEILESQRLLKAYVDCLLDRGRCTPDGKALKETLPDALENECSKCTDKQKSGSDKVIRHLVNKRPEMWKELSAKYDPNNIYQDRYKDKIEAVKGQ

>CsupCSP9

MKAVFLLCLVVVAVSARPEAQYTNKYDNVNLDEILVNKRLLVPYIKCALDQGKCSPDGRELKSHIREALENYCAKCTPVQQDGTRRVIAHLINHEPDYWRQLSVKYDRDGKFAVKYEKELRTIA

>CsupCSP10

MRSWLVCVLALAGMVCCSAQHFNRYDNFNADSIIQNDRILLAYYKCVMDKGPCTKDGKNFKRVLPETLSTACSKCSPKQKLVVRKLLLGIRVKSEPRFLELLDKYDPQRANREALYSFLVTGV

>CsupCSP11

MKTVVALCAVLAVAMARPETYNSQYDNFDAQELVSNPRLLKNYGMCFLGKGPCTSEGKDFKSTIPDALKTECSKCSPKQRVLIRTVVKGFQEKLPDIWSEIFQKYDPNGEYKEAFERFLNSSD

>CsupCSP12

MKLLIIAVAFAIPCVLAQTKSYTNKYDTVNLDQVLSNRRLLHAYIKCTLDKGKCTSEGRELKSHIAEALQNGCEKCTNAQRAGMKRVIKHLMTYEKAYWTQLVEKFDPAREYSQKYEKELSTL

>CsupCSP13

MAIFVMLAGVAQQVMCYDEKYDKMEVDKIIANDELFNSYLNCWLDKGPCDKENAAEMKTLMPEVISTACGKCTPVQKKNIKKLIQGLTTKQRAHQLKELMLKYDPKREYFANFAKFLLGPE

>CsupCSP14

MKAVILLSCLVVAAYAADKYNAKYDNFDVDTLISNERLLKAYINCFLEKGRCTPEGADFRKALPEAVETTCAKCTEKQKLNVRKVIKAIQQKHPKQWDDLVQKNDPSGKYRASFDKFIQGS

>CsupCSP15

MKFAIVLAVALVSVVAEETYSTENDNFDLGAVLADVEKLRPFSGCFLDKNPCDDLTSAFKKDIPEAVQQACAKCNDRQKQLMKQYLEGIKEKLPQDYVDFKKKYDPENKYFENLNKAVGIA

>CsupCSP16

MRTVIFLSLLLVAIVIAEDKYDSIGDNININELLENDRLLKSYVKCLLNKGPCTPEVKKVKDTLPEALATRCAKCTERQKQIGKQLAKEVKKRHPELWNEMIAFYDPEGKYQDAFQDYLKP

>CsupCSP17

MRCLVILSAVLAVALAETYSTTHDQVDVEALVTNPDSLKAITNCFVDKGDCTETTAAFKKVLPEATEQACAKCTPAQKHMLKRYLEEVKKTFPADMDVLKQKYDPEGKHIEALRAALANA

>CsupCSP18

MQISYLLVLCGCVCACVAQAQTQRPPVSDTALEDALNDKRFIQRQLKCALGEAPCDPIGKRLKTLAPLVLRGACPQCSPQETKQIQRTLSYVQRNYPQQWAKIVRQYAG

>CsupCSP19

VDLDEILANDRLLVPYIKCTLDEGKCAPDAKELKEHIREALENGCAKCTDKQKEGTRRVIAHLIKHKNADWQKLKAKYDPEGKYTHKYEKELEEVQH

>CsupCSP20

LENKRLLLAYVNCVMERGKCSPEGKELKEHLQDAIETGCSKCTEAQEKGAYKVIEHLIKNELDIWRELAAKYDPKGDWRKKYEDRARANGIQIPE

>CsupCSP21

DNINIDEIIENRKLLIPYIKCVLEQGRCTPEGRELKAHIKDALQTSCTKCTQKQRKASRKVVKHIRANELDYWKQLLAKYDPDSIYEKN

>CpunCSP1

MISTKYLIVLCCVAAAVARPSDKYTDKYDNLNIQEILENKRLLKAYVDCVMGRGKCSPEGKELKEHLQEAIETGCEKCTEAQEKGAYTAIEYLIKNELDIWRELSAHFDPTGKWRKKYEDRARANGIEIPE

>CpunCSP2

MKTIILVGLTIVVAVAWARPQSTYTDKWDHINVDEILESQRLLRGYVDCLLDKGRCTPDGKALKETLPDALENDCSKCTPKQKEASDKVIRYLINKQPEYWKELSVKYDPDNIYQEKYKDKIQEVKATA

>CpunCSP4

MRSCIVFACLLVSVFAAEKYNSKYDNFDVETLISNDRLLKSYVNCFLDKGRCTPEGTDFKKTLPDAVETTCAKCTDKQKTNIKKVIKAIQTRHPRQWDELVKKNDPTGKHIVNFNKFIES

>CpunCSP5

MRAIVLLSCLVVVYAADKYSTKYDNFDVDTLISNDRLLKAYINCFLEKGRCTPEGADFRKALPEAVETTCAKCTEKQKNNIRKVIRAIQQKHPKQWEELVKKTDPSGKHRADFDKFIQSN

>CpunCSP6

MKTFVALFALVAVALARPQETYNTNYDNFDVKQLVENPRVLKNYGKCFLDQGPCTPEGSDFKKTIPEALKTECAKCTPKQRELIRTVVAAFQSKLPEVWAELVQKHDPQGTYKKSFDSFLHASN

>CpunCSP7

MKYDDFDIQPLLDNDRILTGYTKCFLDQGPCTPEAKDFKKVIPEALESSCGKCTPKQKQLIKTVIKAMMERHPDFWTELVDKYDKDKKYRENFNKFIESDDK

>CpunCSP8

MKIVILTLCLATAILAQEKYDAVDDDFDISEVLQNDRLLNSYAKCLLDKGPCTPEVKKVKDKLPEALATRCAKCTDKQKQIGKKLAQEVKNKRPELWKELVAHYDPDGKYQDAFQDYLKP

>PintCSP1

MTNNNMNCPRSRPEIFSLLAVTTIAAVLVHQPAKVYCADGTIYPSQQQQQQTMMFTAPSGYYLSTYDNLDVGHLLRNKKVVSGFVKCFVNEGPCTPDGKLVKAYLLPEIIRTVCGKCTPRQKDMSRAVLRHLYTYRRADFDKIMQIYDTDNKKNEIINFMNQK

>PintCSP2

VVRTITTHYAPPHHRHKSPFATTPATTPASVVTMNCKVLIALCCVAVYAAQANPAGVATATAADDEIKDFPAYIKRFEKLNVEQVLNNDRVLASHLKCFLNEGPCVQQSRDLKRVIPVIANNSCNGCTEKQKTTIKKTLKFLRTKTPDE

>PintCSP3

MISITQVLHCSNRTLWWIVGSVFIKMKIAIYTISLALLGLCACDDTYTDRYDDIDVDEIVANRRLLTPYLKCILDEGRCTPEGKELKVHIKDAMQTACSKCTPVQKSKARKVVKHIQTNEEDYWKKLIAKYDPDNQYRSIYEAFLASDD

>PintCSP4

AGTAYRRVGQGVSKHRLNMKYIVLVPLMVVLALAWGKSTYTDKWDNINVDEILESQRLLKGYVDCLMERGRCTPDGKTLKETLPDALENECSKCTAKQKNSSDKVIRFLVNKRPDLWNELSTKYDPQNIYQQRYKDKIDSVKEKN

>PintCSP5

MIFFLLAALITYLFNGSVCEDSTYTTKYDGVDLDEILANERLLAGYINCLLDAGPCTPDGKELKNNLPDAIQNDCHKCTERQREGADKVMEYIIDHNPDDWIKLEEKYNSDGSYRKKYLDNKNSKKTKAAKDKSSKEKSE

>PintCSP6

RTSRTFSKYQESINMRSTLLWLCALTVVASCWAQHYNNRYENFNADAVIANERILLAYYKCVMDKGPCTKDGKNFKRALPETLTTACGRCSPLQKIVVRKLLLGIRGKSELRFIELLDKYDPERSNRDALYNFLGV

>PintCSP7

IRYMKHCSNQTHNTQIKMKTVLVLFSMVVLVLSYETSHDDIDVEALVNDTDTLKKYTSCFVHNTDCDDNSSHFRADLHEIYEQACAKCNQVQKHMLKRYIETLKEKLPDQYQELKKHYDPEGKYSLPLETAIATA

>PintCSP8

MRTVILVCLLAVAALARPEKEYYTDKYDNIDLDEILDNRRLLIPYIKCSLDQGKCSADGREMKSHIRDAMETECSKCTKKQKKNTRKILRHLIIHEKDFWEQLKAKYDPTAKYYPTYEHAVEIDDKAFFGEA

>PintCSP9

EEVKMENKPTVVKSETLAAPLPTTIVKRATPQVVSTQKDSSLPNVSEDVLDKALSDRRFVQRQLKCATGEGPCDPIGRKIKAHAPLVLRGMCVKCSQSEIKQIQRVMSHIQKNYPKEYTKMLKQYQSGF

>PintCSP10

MKVCVALCVLALTAVALGRPQSQYTDRYDNIDLNEILNNRRLLVPYIQCVLGEGKCTPDGKELKSHIREALENDCAKCTENQREGSRRVIAHLIKHEQDYWAKLKAKYDPSGKYSKRHEQELAAATA

>PintCSP11

MQVRMNLLIIIGVLVVVDARRYSSKHDNIDIDRLVRSPLYMEESAACYLDRRPCSKMTATLKRAIPEIVNLACGKCTPAQKYILRRYLEELKKQRPEDYAAFRHKYDPDNVYFVDLEAAISVNKTDI

>PintCSP12

MKTIAVLCVVVAAVVARPEEAGFYNPQYDTFNAEELAENVRLLKNYGKCFLDEGPCTAEGSDFKKVIPEALKTSCGRCTPKQRVLVRTVIKAFQTKLPDIWEELVNKNDPERKYKAQFDAFLQGTD

>PintCSP13

KYTTKFDNFDVDKVLNNDRILTSYIKCLLDQGNCTNEGRELKKVLPDALKTDCSKCTAVQKDRSEKVIKFLIKNRSKDFDNLTAKYDPSGEYKKKIEKFDAERAAAAKH

>PintCSP14

MQKIILLACAVIITVNSVAAAPQMSDRQLDRTLTDRSTMQRHLKCALGEGPCDPVGRRLRTLAPLVLRGACPQCTPHETLQIRRTLAFIQRNYPWEWARIVRQYG

>PintCSP15

MQLSYIIVVCAVAAVASAAETTRPPVSDTALEEALSDKRFIQRQLKCALGEAPCDPIGKRLKTLAPLVLRGACPQCTPQETKQIQRTLSYVQRNYPQ

>OfurCSP1

MISTKYLIVLCCVAAAVARPSDKYTDKYDNLNIQEILENKRLLKAYVDCVMGQGKCSPDGKELKEHLQEAIETGCAKCTEAQEKGAYTAIEYLIKNELDIWKQLAAKFDPEGKWRKTYEDRARANGIVIPE

>OfurCSP2

MKTIVALCALVAVALARPEDTYSTAFDSFNAQELVDNIRLLKNYGKCFLDQGPCTPEGSDFKKKIPEALKTDCGKCTPKQRELIKTVVHGFQSKLPDMWAELVKKHDPEGQYTESFDAFLNSK

>OfurCSP3

MKTFILICLSALVMVSSADKLDDLLNTDMEKLLADDAVRKQVVGCMTDELPCGDYQAYKDMLPDLIATNCGKCTPEQKKRYEEINKFVLEKYPNEYNAVVSKYRPKTE

>OfurCSP4

MKTFVLLALSLVVAVAYARPGAQYTDKWDHINVDEILESQRLLRGYVDCLLDKGRCTPDGKALKETLPDALEHDCSKCTEKQKASSDKVIRHLINKQPDYWKELSAKYDPNNIYQDKYKDKIEEVKSKN

>OfurCSP5

MHPQHFCMIVMVTTAAADFYSAKYDDFDIQPLLENDRILQGYTKCFLDQGPCTPDAKDFKKVIPEALETSCGKCTPKQKILIKKVIRAVMERHPDSWKELEDKFDKDKKFRDSFNKFLEEKD

>OfurCSP6

KLVIISLCLAAAVVAQEKYDSIDDNFDISEVLNNERLLNSYTKCLLDKGPCTPEVKKVKDKLPEALATRCAKCTDKQKQIGKQLAKEVKAKRPDLWKELVAHYDPEGKYQEAFQDYLKP

>OfurCSP7

MKTFAICLLALVAVVSAYPQAKYTDRYDSINLDEIVGNRRLLVPYIKCILDQGKCSPEGKELKSHIKEALENYCAKCTETQRDGTRKVIGHLINNESEYWNQLTAKYDPQRKYVVKYEKELRTVS

>OfurCSP8

MKLVAFIPTFTYLLLGANAEESPTYTTKYDGVNLDEILENDRLLTSYVNCLLETGPCTPDGKELKNNLPDAIQNDCKKCSERQREGADQVMEYIIDHRPDDWEKLEKKYNSDGSYKKKYLERKEARNQSNSAEKSQENDSKSKE

>OfurCSP9

MKFLVLSAVLALALADSYKSDYDSLDIAPIVNDPEALSKLTACFLDKGPCTPIAADFKTYLPDATETACSKCNTAQKQKLKLYLQKVKETSPDDLAALKAKYDPDSKHVDALIAALKE

>OfurCSP10

MKTIMLVAFLVGLAMADEKYTSENDNFDVEALVNNTEELQKFSGCFLDKNDCDAVSGDFKKDIPEAFQQACAKCTDAQKHLFKRFLNGLKEKLPQDFEAFKKKYDPEDKFFAALDKAINA

>OfurCSP11

MRHIIILLAVVALVTQSFADEETEKKEEKKEEKKEEQTDEKKYTDRFDDINFEEIIANRRLLVPYLKCVLDKGRCTPEGKELKAHVKDAMQTACEKCTDKQKTGARKVVNHIRDNEKEYWEELINKYDPKGEFKSIYEPFLAAKE

>OfurCSP12

MKFLVVLSAVLAVALARPDSYKTDHDGLDIEGIVNNPEALAKVTACFLEKAPCTPIAAEFKSVLPDATETACSKCTAAQKHMLKLYLLKVRETAPDDLKALKTKYDPDSKHIDALIAAIKDA

>OfurCSP13

MRAVVFLSCLVVVLAADKYNSKYDNFDVETLISNDRLLKAYINCFLEKGRCTPEGADFRKALPEAVETTCAKCTEKQKNNIRKVIRAIQQKHPKQWEELVKKTDPSGKHRAGFDKFIQSN

>OfurCSP14

MWIQLAILATFVSIVITEMGPPGIERTFSDGVTSRGYRVVYGDEDLTVINEVVGNMEKNDILKAKASLNEAIQPLPAGDVKCLMSADRYCSVEMRKVKGVLIQALKNDCEKCSNTEKDTAGRVAASMMTYDPVGWKLFLTRYDGLSKIQRILG

>OfurCSP15

MRAVLLLCACAAAVCGQNLDSNRMARMPKYDERYDYLDVDALFNSKRLVRNYVDCLINAQRCTPEGKQLKRILPEALRTKCIRCTERQKKTAVKVIKRLKYEFPEEWAKLSSRWDPTGDFTRYFEEFLANESFNTISGSADGNDAAGPSSIPPLPPVPPRLPAAPPSTPPPLPVEPVSTSPKPVILNRFGDDGELMMGSPSSAALTPRPSTARPPLS

>OfurCSP16

MSHRKVLVLSHLMVFLCVQCFAKLHNYDNFDMETLLLNTTRSRALFECVRDETKCANKEDKEMKDDIFEMVTTSCANCTAKEKQKFGDAMKALHRSMGESQIITMFINKMTNMFQGGLSDTEKTT

>OfurCSP17

MQTTLVLLLVVAACAYAAEAPRPQVTDTALEDALNDKRFIQRQLKCALGEAPCDPIGKRLKTLAPLVLRGACPQCSPQETKQIQRTLSYVQRNYPQQWAKIV

RQYAG

>OfurCSP18

MQKLIILALVCTMGWSVVVAAPQMTDAQLDQTLTDRATMQRHLRCALQEGPCDPVGKRLRILAPLVLRGTCRQCTPQETRQIRYTLAFVQRNYPWEWAKLIRQYG

>OfurCSP19

MKTLLFAITLAALACCARAQVYTDRYDTVNLDDVLANKRLTVAYIKCMLDKGGCTSEGRELKSHIAEALQNGCAKCTKAQREGMRRVIKHLIQHEKGYWQELVEKYDPKRVYTQKYENELNSL

>BmorCSP1

MKVLIVLSCVLVAVLADDKYTDKYDKINLQEILENKRLLESYMDCVLGKGKCTPEGKELKDHLQEALETGCEKCTEAQEKGAETSIDYLIKNELEIWKELTAHFDPDGKWRKKYEDRAKAKGIVIPE

>BmorCSP2

MKLLLVFLGLFLAVLAQDKYEPIDDSFDASEVLSNERLLKSYTKCLLNQGPCTAELKKIKDKIPEALETHCAKCTDKQKQMAKQLAQGIKKTHPELWDEFITFYDPQGKYQTSFKDFLES

>BmorCSP3

MNSLIAFCLFAVLAVALARPDDKYTDRYDNVNLDEVLSNSRLLQPYIKCILDKDRCAPDAKELKEHIREALETECAKCTEAQKKGTRRVIGHLINNESKSWNELTAKYDPENKFTAKYEKELREIKA

>BmorCSP4

MKVLIVLSCVLVAVLADDKYTDKYDKINLQEILENKRLLESYMDCVLGKGKCTPEGKELKDHLQEALETGCEKCTEAQEKGAETSIDYLIKNELEIWKELTAHFDPDGKWRKKYEDRAKAKGIVIPE

>BmorCSP5

MNSLIAFCLFAVLAVALARPDDKYTDRYDNVNLDEVLSNSRLLKPYIKCILDKDRCAPDAKELKEHIREALETECAKCTEAQKKGTRRVIGHLINNESKSWNELTAKYDPENKFTAKYEKELREIKA

>BmorCSP6

MKCLTIAALLFVAGLSIAEKYTDKYDNIDVDEILENRKLLVPYIKCVLDEGRCTPDGKELKAHIKDGMQTACAKCTDKQKVSARKIVKHIKQHEADYWEQMKAKYDPKDEFKEIYEGFLAGQN

>BmorCSP7

MKGFYVLCFALFAAVYCKETYSSENDDLDIEALVGNIDSLKAFIGCFLETSPCDAVSGDFKKDIPEAVAEACGKCTPAQKHLFKRFLEVVKDKLPQEYEAFKTKYDPQGKHFDALLSAVANS

>BmorCSP8

MKTILILCALVSVVVCRPEEYYSSQYDNFDVEQLVGNLRLLKNYAKCFLDQGPCTAEGTEFKKRIPEALRTKCAKCNPKQRHLIRTVVKAFQTKLPDLWEELAIKEDPKGQYKHEFTAFINAMD

>BmorCSP9

MKFVLALIALAVVVAARPNDDLFYDKKYDNFNVDEIIDNPRLLKAYTFCFNDKGKCTAEGNDFKKWIPESLQTSCGKCSEKQKYLVAKFVHAIKDKMPDEFDILRKLHDPKGEYTENLDKFLETYGH

>BmorCSP10

MKILIIVVMACVAVTWARPESTYTDKWDNINVDEILESNRLLKGYVDCLLGKGRCTPDGKALKETLPDALEHECVKCTGKQKSGADKVIRHLVNKRPDLWKELAVKYDPDNIYQARYKDKID

>BmorCSP11

MKLTSFLLVGMAMVSAEFYSSRYDDFDVKPLVENDRILQSYTNCFLDKGPCTPDAKEFKKVIPEALETTCGKCSPKQKQLIKTVIKAVIERHPEAWEELVNKYDKDRKFRPSFDKFINEDD

>BmorCSP12

MFMLFIISFIIVPVLKCCGTETSTYTTQYDEVDIKEIMGNERLLVAYIGCLLDKNPCTPEGKELKRNIPDALQSDCSKCSDKQRENADAWIEFMIDNRPEDWTKLEER

>BmorCSP13

MKLLLVFLGLFLAVLAQDKYEPIDDSFDASEVLSNERLLKSYTKCLLNQGPCTAELKKIKDKIPEALETHCAKCTDKQKQMAKQLAQGIKKTHPELWDEFITFYDPQGKYQTSFKDFLES

>BmorCSP14

MKSSLFCVLVLTVVVSSSRQQSYPRNDNININAILQNDRILLGYFKCVMDRGPCTKDGKTFKRALPEALPTACARCSNKQKAAFRTLLLAIRARSEPSFLELLDKYDPSRSNRELLYTFLATGL

>BmorCSP15

MIENFYSKCTISKSVLFLCLIFLPYALNQKYYDSRYDYYDIDHLVQNPRLLKKYLDCFLGKGPCTPIGRLFKQVMPEVITTACAKCTPTQKRFARKTFNAFRRYFPETLMELRRKFDPESKYYDAFEKVITNA

>BmorCSP16

MIEWKRFKILHFLSYLGLLVLVVVCAAQQNRPQVTDTALDEALNDKRFIQRQLKCALGEAPCDPIGKRLKTLAPLVLRGACPQCSPQETKQIQKTLSYVQRNFPQHWAKLVRQYAG
