# Supplementary material for: Identification of chemosensory genes from the antennal transcriptome of Indian meal moth Plodia interpunctella
Source: PLoS One. 2018 Jan 5;13(1):e0189889. doi: 10.1371/journal.pone.0189889 (PMC5755773; doi:10.1371/journal.pone.0189889)
Supplement: S4 Table — (DOC) [file pone.0189889.s004.doc]

>OfurOR2

MMTKVKAQGLVSDLMPNIKLMQAAGHFLFNYHSDNSGMTTLLRKVYSSVHAFLIVINYLCMAANMAQYSEEVNELTANTITVLFFAHSVIKMLFFAVNSKSFYRTLAVWNQSNSHPLFTESDARYHQLALTKMRRLLYFICGVTVLAVMSWITITFFGESVRMIANKETNETLTEPAPRLPLKTWYPFDAMSGTMYVVAFVYQVYWLFFSMAIANLMDVMFCSWLIFACEQLQHLKAIMKPLMELSASLDTYRPNTAELFRASSTEKSEKMPDTVDMDIRGIYSTQQDFGMTLRGAGGRLQNFGQPNPNNPNGLTQKQEMLARSAIKYWVERHKHVVRLVASIGDTYGTALLFHMLVSTITLTLLAYQATKINGINVYAFSTIGYLSYTLGQVFHFCIFGNRLIEESSSVMEAAYSCQWYDGSEEAKTFVQIVCQQCQKAMSISGAKFFTVSLDLFASVLGAVVTYFMVLVQLK

>OfurOR3

MFKIGNENDINARHPMDLRYMKFLRMLLRMIDSWPHQQLRDSKPVRFRDSRYLFIEGAGVGIGGLFYVRSHYKVVPFLEIGQTYLTIFLSVVATQRVTIAWFKSFREVITEFVLKIHLFYFRHKSNYTENVYQRINRLCSVFVAFVAVEVTIGIFLFNLMPFLNNYKKGMFNQELPANKVFEHSINYSLPYVDCYTNLIGYIVMTLINIICSYDCGMFFSSVDVCIAVIVFHIWGHLKILDHRLRTFPTPVQMRGHQPGEPGNDLMYTKEENMKAAAMLRDIIEYHGMIMRFMTKTSEAFGPTLCLYYVFHQVSGCILLLECSSLDPESLGRYAGLTVTLFQLLIQVSVIVELLGTQSETLKDAVYSMPWECMDTSNRRTVLFLLYNVQEPIRLKPMGIVSVGVQTMATIIKTSFSYFMLLRTFT

>OfurOR4

MPAVHQNPSTLSYIITVKNALGPSGIWPSNIFEDKLQPLFFRIHRETLPYHTMLIVFGGLYYLSDNFRIMSFLDMGHIILSTFLAMVTAMRSVVPNLKIYVALLTKLGREIHLMHFAHKGPYYEEINKTVDKASHIYTKFIVVFMYMTMMMFNITPIYNISKNILSSKTENSTQEYALYYSFPGINPMNYYPTTTVYNFYLSYNCGIMMCGLDLVLFLMIFQLIGHVYILRHNLENFPSPKNKVVLNIGDLPRYKNKENCIVEMFDAKENEEVRVRLAECIEHHKIIIRFTDEISVVFGPILAFNYMFHMVGCCLLLLECSAGNQIIRYGPLTTVVFGQLIQISVMFEMLGAETEKLKDSAYFVPWECMNISNRRTAQIMLHKMQDKISIKALGLAAVGVNTMMGILKTTFSYYAFLQTMND

>OfurOR6

MQQESPLQLGYIKTIRFFLRPSGSWPSDVFEGYLPLPIRIHRATLPFHTTIIVMGGLYYITDNFHRLSFLDMGHMIITTFLAMVTALRSILPNLQTYNSLLCKFIQEFHLMHHAYKGDYFEEVNKTVDKISSYCTKFSTIIMYLAILFFNITPTYNNIRHTLISKTENYSMEYSVYFSFPGFNPLDHFASTTVYNIYLSYNCSTLFCGFDLLLFLMIFQIIGHVYILRHNLENFQSPKNKITLNLRGDALITNNTCTYEVFDAQENEEVRLQLAECIEHHKIIIGFTDDVSGLYGPLLAFNYFFHMIACCLLLLECTEGSYDAVLRYGPLTILVFGQLIQMSVMFELLGSETEKLKDSAYCLPWEAMNTSNQRTAFIMLHKMQYKISLKALGLAAVGVNTMVGILKTTFSYYAFLQTMGDR

>OfurOR7

MVIIRSAFRVVGAWPSKFIGDVQTTSDVVVKYIQLVLNVVCQVAGILYLRENMDKLSFFELGHSYITVLMSVVSMSRIITYCTEAYQEIFSLYVRKIHLFNVRNDSEYAMEMHTKINKLCYFLTFFIHAFMTLGILMFNLIPMYSNYISGKFNRETGAFSGVSNATMEHAVYFLWPFNDTTDPIGYAIIVVFNWYISLVCSINYCTFDLFVYHLVFHIWGHLKILIHNLETFPRPIGAINEEQNDYTEEESKQIYERLKKLVQHHNLIIDFIARISDTFGLSLFVYLCYHQVCGCILLLECSTLELSALIRYGPLTAITFQLLIQVSLVFELLGSITESLMNAVYELPWEYMEVRHRRTVHIMLRQSQVSLNTRALNMVDIGSRTMIAIIKTSLSYFVMLRTFATDD

>OfurOR8

MSNILKYFNTRNSYELSFFREGDPLALNYFKIIRIFMVAPGAWPADVFGEKLSLLVRVHRALMPYHTSVIVIGELYYLYIHKEELDFLNMGHMIIFSFLGVLIAIRSILPQLRKYHLLLTKFVKVMHLMHFKNKGPYYKQINETVDKISYYYTIFVALLVTTAMINFNIVPLFNNVTNVLIYKTENFTLEFALYYKYPGFDPLDYFTSTTIYNVYLSYNCSIMVSGIDLILFLIIFQIIGHVYILRYNLENFPSPKIKVVFKLKEILKHKGNEDISSEMFDAEENREVRLKLQECIEHHKLIIGFTDELSELFGPILAINYFFHLVCCSLLLLECSEGGAWIRYGPLTVVIYGQLIQMSVIFEMLGSETEKLPDSAYFLPWECMDTSNRRTACIMLHKMQYKISLKALGLAAVGVSTMTGILKTTFSYYAFLQTMGE

>OfurOR9

MLYYSQLTVRRKVIKNIVDGYLACDAQTLKSDRFRQNLLKGLRIVKKRGLIFWMVIIGNGTIYIMKPIVTPGRHIMEDLFIIYGLEPMFESPNYEIGFLLTAGGVICTCYLPANITALLTVLIGYTEATMLALSEELVHLWSDAQEHYNKYLLETQVDNAGALVTPNDDIKNQIINKYIKQKLEEIVKIHTTNINLIQQIEHVFRGAIAVEFLLLITGLISELLGGLENTYIEMPFALMQVAMDCLTGQRMMDACDKFENSVYDCKWENFNVANMRTVLLMLQNAQKTMVLSAGGMTQLSFTCLMTVIRSIYSAYTTLRSMMA

>OfurOR10

MFRLKEKDVIASNTQSQVFKPNIFFWKIFGWWPEVTSTIYYRCYYISFLSLTSVVYLFLFTLSLLYSPIELEIIIAQAMFYFTEISGLSKIFMIVIRREDIQKAFKMLDSEEFQGDDVIPREIINKNKVYYLKYYRACATFYYIGSFFLLFLPIIEYVAGHADLELPLCQYYFLSEHVRDKYFNVIFIYQFFGLFVLISGNVNIDTFICGLLLMAIAQFRMLNWKMSNLKMNPLDLENESDDEETIMMRKLNKCLKHYDLILEYCDHIQDVLSAAIFAQYGTAAATMCLSMCTVLMPMTSEDWLFMGCYIGAMTLEIFLPGLLGAELMNESQKLVAAAYSADWIPRSESFKRSLRLLVERANRPIVITGLKMFTLSLETFTSIIKLAYSFFTLLKNVQETEIA

>OfurOR11

MEFIKRNYREFRSRMQDYSYDSLLSIVNFVPSLVGFSILGNKISAPFWILHLSLLFYIYGVGCTVYQVKYAGDARDFIKCYVNVTLLLLIANNSHWFLLKRPLLKSILQEISQSDALATANEAFRGKHKRAVQRIKRILFMFYGFNLTNAMFVYLPNRMDVKNSYSMTPCYGMEPLTASPNKEICSALLLIQEISIMMVVLNYQALLVVLIAYTALLYRLLSEEIMTLNNYDRQTYFNNPIAKTMLHELIKRHVILLSIIDQLKSLYSGSIGINFGSNAVCMSLFFYLPFQEWLQFMPVVVYCFLVFFLYCFLCQRLTNAAEYFEQCVYSCGWENFDVKEKKAIYFMLRQAQRPVEILAADIIPVNISTFATTLQAMFKFVTVVKV

>OfurOR12

MIIKFLQSFEDPDKPFYGPNFWILTKTGLILPENKIAKALYILMHEIVAFFVFTQYMELYIIRSNLDLVLTNLRISMLSVVCVVKANTFVFWQEKWNKIIDYLTEADSVERYSNDPERKKIIDKYTNYSRRVTYTYWVLVFITLATTIGSPFIHFVSASYRESLRNGTELFPHILSSWMPIDKNHSPGIFITIVWHFTVTSYGALIMSSYDTSIMVIMVFFGGKLDVLRERCKQMLGTGEVELSDDEVAARVRELHNTHVLIMKHLRLFDSVLSPVMFVYVVMCSLMLCASAYQLTSATNAAQKLLMAEYLIFGIAQLFIFCWHSNDVLVKSENVMLGPYESRWWDANVRQRKSILLLAGQLRISKVFTAGPFTNLTLSTFITILKGAYSYFTLLRE

>OfurOR13

MLNVLKRLENPKRPLLGPNVKALKFWGLLLPENIYMKYFYLLMHVLVTIFTATEYVDIWFIKYDLNLILNNLKITMLATMSVLKITTFLYWQQHWKDIIEYVTRADLAQRTTDDVEKNVLITKSTRYCRKITLFYWSLMYTTVVIVIFQPIFKYFLSRNYRENVKNGTDSYLQVVSSWVPWDKSTIPGYLIASAFQSYAAIYGGGWITSFDSNAMVIMVFFKAELELLKIDCSNMFGTETKPVSDEVALKRLKDCHRRHVELLKYSRLFDACLSPIMLLYMFVCSVMLCVTAYQITSETSAMQQFLTTEYLVFGIAQLFIYCWHSNDVYYASLQLSQGPYESLWWYRDVSHRKNLYILTAQFSRVVVFSAGPFTKLTVATFLSIIKGAYSYYTLLSKSQTK

>OfurOR14

MTILNSIWRKLTNTKALEKSSGCLETQFFETVYRVSYLTGISMADEDIPYLIYSSVVKLLIFLLIVGEFWHLATEVTSFDEMADMVNITVIQYIAIFRYRSMLYHKDVYKKLAISMESQYFDISTKERRDVVDYWVKRNANNVKLLLVLGNCTLIAWFLYPLVDDLEYNVFIGIRLPFPYYSPVCYAFVYLLLLIVFSYISHFVMANDLIMQAHLLHMVCQFDVLCNCFENLMEDCAKGFKGIDRESLLANANYREVFKARLGDMITQHRYILDHAMELRHTLSGPMLGQLAASGTLICFIGYQATTSGAYNITKCLMSLFYLCYNLLVFYIICRWCEEISVQSQRVGEAVYCSNWECGASNIPGVKVSLLMVITRANKPLTLTAGGVYDLSLMTFSSILKTSYSALTLLLRLKSTE

>OfurOR15

MALMVYQKVLEEKITENDEEIFFKPFEETFTILIFSMVFGMIYPSDRMKNWQILGFFGFIIIMIPALSAVYYDMYLAYLDRDMDTIFRHLIVIGPFNALYLKWVYMYYYRQQSKDAIEEMNRYFANLNFKPITHKRIAKKWLIRSFFLEKSWAYCLIVGSFSFPVMAICKTTYSTLFDEEPRRYFIHELRSPQGPGMNYEFPFFEVLFVNTCIASCMYFLNFSGYDGFFVQLILHTCMRMAICGEAVKDSFKIDDKAMRRSALHKVIDEHIAICHFMDNINCIFVQWMSLFTVALTIHVCICVFHLSEGTYQDMEFMFAFTAASIYLCMITSCGGLVEEESENLADAFYQSGWERVLDTHCNYLLVFMIARAQKTFRVRTLFHVHVNHELLIAIVKMAYTLLTFLKQT

>OfurOR16

MSLWSTIRKFGLGYCDLPTMLWNVSFMLRALTLNIDSRYKKRIPLIFYIIFALVAASYFYIYLISMAWFVFWHSRETGDLVAAMVVASLGISSEIGTAKLIYMFLYRNKVRELVDMYLDCDALVKPGSRFANNLTKTLRNVKKRAMIFWIVIMGNGVVYVLKPLLISGRHIMEDLFTPYGFDPVYESPNYEIVFLLMTAGVLFTCYLPANITAFLIIITGYTEGQMLALSKEMLNLWSDAQQFYLDHRTFDLDTTRPVVTLDSEQITKKKIVNEYVKKRLHEMIKIHTTNINLLNHVERVYRGAIAIEFGILVLGLIFELLGGLENTYLEVPFALMQVAMDCLTGQRVMDASKAFEDAVYDCKWENFDVANMKTILLMLQNSQKTMRLSAGGVTTLSFSSLMMVFRSVYSAYTTLRTTMNK

>OfurOR17

MEKSPNFEFGYALVAASVWFLCYVPANVTSVLIVFAGYIEAQMLALTQELLHIWADAEQHYANINLNTLKRGTFVDAKYKKRVINEFITMRLHDIIRKHATNVHILHLLEEVFKGAIAFEFLFLIMGLIAELLGGLQNTILEMPYAFVQVAMDCWTGQRVMDASAEFAAAVYACNWEMFDVPNMKIVLLMLASAQKTMKLSAGGVTMLSFECLMSVVKNIYSAYTTLRSAFTINTHAH

>OfurOR18

MKFPWRRGGMRPKEPLSTLDSLPINNYTHFLEIPLKIVGCWDWYDHPKSEKEIIINNVYFCMVLFVLINVPATLYIHLHTEWVDVMTSLDKLADCLPFVVSIIIVVYFGLYRKELYELTKFMQRKFHYRSANGLTNMTMLNSYKTARNFGYFYTACTMFSVSMYMIPEIVNRLKRQPLQSYMYMDVTRTPFFEFTLLRQCVAQAFVGLAMGQFGVFFASNAILLCGQLDLLCCSLRNARYTALLRCGVSHRSVAAAHSDIQGDELYNYIYNIAEMRQSIYHYDQRMSYEIMNKRSSFDIYSSEFDAATCEALRDCARACDVINTFKAKFESFVSPLLALRVVQVTMYLCMLLYAATLKLDMVTVEYLVAVALDIFVYCFYGNQIIIQADRVSTAAYQSAWPTMGVRPRRLLLNILLANKRPVVVRAGNFLSMDLHTFVVIIKTSFSYYTLLVNVNEK

>OfurOR19

MEGLEAYPEEFVNSLKLSLEYYKRVNITFFGSKTSFWDKYRHFFVFGIPFAFFYYTVTMYMVKVVAEGLDPFAKPDMIALWLISTQVIFKYILFTKNKEGVRLVIEHLGAVWRMTDLTKEQILIKNSSLKFLKYGLYIYNKSCMTTAWQYFLYPFISMLFKHIFWGNEIEMVLPFPCEYPFAVDNWPVYLAVYALQIIGALQMVHLYLAPNFLLTNLSIHISTQFRLLQDDLINIKPTNNKKTKYQYDMEITKYYEGKEYTIEDFVRRHQDIILLTRQLNDAFNKMVFVNLVISTVVVCFFAVAVKTTIDPAYKLTNGAALVAYMANLLIVCYCSEMLSISSTGIALSAAKNMWYDGDLRYQKIICIIIMRSQKPCTLLALNYYSISMKTFNKALKTTYSYFSLASHIYDGRKERKYTTEY

>OfurOR20

MWNIRFLKEKRFTILNVFNFLEDPRYPLVGPHLRLLGLTGLWHPNLNSKTRFKQYLFFITIAFFFSQYVKCAVKLEPSSLMLILQYAPFHLGIIKSCFFQKDHKKWESLIDYISGVERKEIANGNKDSNDIISEYISRSRKVTYFFWALAFFSNFTIFTEPYQKNQINVNGTSVYLKIFDGYTPFSEVPPGYYASMLTQTVLGHIVSAYVVGWDTLVCTIMIFFAGQLKISRLNCANVIDINNAERSHENIVNCHSFHTILVKNQKLFNSLISPAMFVYLIVISVNLGVCIIGIVQLQDDLTTLISSCVFVMACLIQLLLFYWHSNEVTEESTLVSYGSFECDWVELDQRFKKEVALLGMATRTRLVFKAGPFNEMSLTTFIAILRLSCSFYTLLSKTM

>OfurOR21

PRLARAHALYCRFALAATSVYLAQECVYAYQVRNDMDKLARVMFLLLCHVTSITKQLVFYMSADKIDEMINALDDPLYNQPAAWQRALLAATARSAGRLLRAYSGTAVVTCTLWIIFPILYYSQGLPVEFPFWTNLDHSKPTFFVILLMYSYYVTTLVGIANTTMDAFMGTVLYQCKTQLRILRMNLENLIERATTVVKENSDEIFDKVLDRLFLECLEHYRQISETNRRLQDIFGTSILVQFGIGGWILCMAAYKMIGLNILSIEFASMTLFITCILTELFLYCYYGNEVTVESDRMVEAVYAMEWLHAPLRFKRSLVLVMERAKRPLRPAAGHLIPLSLDTFVTILKSSYSFYAVLRQTK

>OfurOR22

MLRNFLLSLENDNHPLLSPTLWGLQKWGLWQPNKVLNSNISNFIHFAATLFVISQYVELWLIRDNLNYALRNLSVTMLSTVCVVKAFTFVTWQDQWKDVIDNVSLLEKRQLSKKDKITDKIISEYTNYARRVTNFYWTLVAATVFTVILAPLVCFLSSPDTRERIRDGYEPYPEIMSSWVPFDRSRGLGYWVTVLEHILICFYGGGIVATYDSNAVVLITFFAGQMKLLSVNCSRLFDDEKEMTYEDDMEKIRACHYHHLMLIKYSKILNSLLSPVMFLYVIICSLMICASGIQLTTEGTTTMQRIWIAEYLMALIAQLFLYCWHSNEALVMSNKVDDGVYASAWWSRSIQVRRCVLLLAGQLRKSVVFTAGPFTKLNVPTFIAILKGSYSYYTLLNNKDD

>OfurOR23

MAEQPIDKSLRKIRFIFRYAGMNLEERPRTWCQSFIYVVNFLWIATDIIGEINWIFEGVSKGTSFVELTHVAPCLSLGTMSEFKTAFVVAHEKSLFRLIGNVREMERKRLVGPIAHKIVKEESKFLYNLVFAMKMVNWVLVVVFDFGPLVWIVVKYFIYGELELLLPIIDIYPFDCYDLRIWPFAYIHQIWTAWVVVTEILGVDCLFYICCTHVMIQFKILNHEVTNVIAESRSAKRIEVTQLREKFNELVKWHQDIINSAGLLEDIYSKSTLVNFLTSSLIICLTGFNMTALDNVRMTVAFAFFVVAMLQIYFLCFFGNMLMDASTDVSTAVYNSRWYLSDAAFGKSALIMQIRAQKPCIVTAAGFAEVNLRAFMKIISTSWSYFALLRTVYQDI

>OfurOR24

MFSLSFVITHDLTLIKLCIFYFKNDQIQDIVRTLEIDLYNFYQNNRKNRATVRITRIMSASFVFFGWITIGNTNVYGTIMDFRWKAEVAKLNASSIKPPRTLPQPIFIPWEYQTDQSYISTFVLETVGLLWTGHIVMAIDTFIGSVILHMSSQFTILREAIVTAYDRTITKMYINAKHQYDSLEAVSSNDEENSSIDQSTQDGMEALVLARFSKKEVELALQETLKNCFQQHQVLIRCVEKFAETYSYGFMTQLLSSMAAICVVMVQVSQDASSFKSIRLVTSLAFFIAMIIQLAIQCFTGNELTLEAARIADAVMQCKWERMPPSLRRMLIMVMMRAQRPLRLSAAGFAYMNNDCFLAIMKAAYSYYAVLSQKTK

>OfurOR25

MAKEAFENSLRLTKLFLLLSGIRITRRKWRKSVENFFDYYLYYISLSWLYTDVCGELNWLIEGILTGKSFIDLSLTAPCITISMLATSKSIFLYWNRDVVAKIVDKLRDIHPEDKEFDEYKQLGLYQVESNEPDVEKEIVEESRKFLSFVVHLLFYICAVVICAFPLMPVTSMAYDYYTTGSTECKYPYLVKYFFDPYTMKMWIAVYFHHVVSTAIVGANVFGSDSLFYVVCIYIQMHFQTLCHRCECAVVSSREGTRRNVANAVKRHQELIDLVNQVELLYSKSTLFNIVTSSVLICLCSFIITVLDEIIVVVTFATFLVMNLSQISLLCYFGDILMRSSTEVSSAVYNSLWYETDQSVKKSMLVILMRAQKPCKLTAWNFADLNLTAFTTILSRSWSYFALLKTMYK

>OfurOR26

MEEESLFDKSLKKITFAFRLTGLNIENDKRNLKQNCVYLFNFLWLNTDIVGALQWVLYGIASGKNFTELTYVAPCLALSILGDIKGVFMILNEKKVHILMDNLRSLELKAKEFENSEREDMIEPEIKFLNIITSVLNVLNCLMIVVFDASPLILIAVKYFTTGQLELMLPFLDVYPFDSFDLRYWPFAYIHQIWSECIVLLEICATDYFFFTCCTHIKIQFKLLQHQFQEIIPSRSVSAVDSIDQAAIRTKFQELIKWHQEIIRSANMLEGVYSKSTLLNFCTSSLVICLTGFNVTTIDDKAFVMTFIIFLFMSLLQVFFLCFFGDILMSSSMDVSNAVYNSRWYLTDVMMGRNVLLVQTRAQDPCKLTAAGFADVNLRAYMKILSTAWSYFALLQTIYC

>OfurOR27

MSDITLSEAKREIAESLTLNTFCMRRIGLSFEEPKNASSYFAQKFMLVLSVMSICYHVFSEIVYIGLTLSNSPNVEDVVPLFHTFGYGALSIAKVFALWYKKDVFKQLISELAGIWPMSPLDDDATVIKAKSLTALRIAHQWYFVINVLGVWFYNLTPIIVYAYRVWQGQDVEMGYVWVSWYPFDKHQPVAHVAVYIFEIFAGQTCVWIMVGTDLLFSGMASHIGLLLRLLQRRLETLATMEQTEEDNYRDILASIKLHQRLIRYCNDLEVAFSFSNLVNIILSSVNICCVVFTIVLLEPFLDISNKLFLGSALIQIGMLCWYADDILHANADVAAAAYTSGWYRTSARCRRALLFLIQRAQKPIAFTAMGFTDISLVTYSSILTRSYSYFALLYTMYNDK

>OfurOR28

MIITYHQDLSTVVEFNTIPYNSMEDHTIRMEKEFGPFHDTYRLNMYSMSYGMIYPNPATNKWRLLAIPILCFTTIPMTVLVFLDIRRYWIDGEILEVIRHVGLIGPFITGILKMCLLYYKEEPTSQILALINRDYASFNQLPESYKPLVRGYVKNTRFYQNIWIACVLMILSTFVLTASVMNICETLFSSEPKRHMIYDVRLPIDRPGAQFETPYFEILYIYMLYIAAVYTINFTGYDGFMIACVYHACLRIELFCKYVHDAMGYEGDELRRRLGEAVNHHCETFKLIEKCESSFNFYLGLVYVVVTTELCLCLYLVMEGFEFDYKFSSFSIGTILHIYVPCLVAEKLKCVCENASDLIYCCGWENNYDLSMRKFIPYMMARAQKPVAMKALGLITFEMSLFASTMKTAYSMYTIIKTQ

>OfurOR29

MKWKKNKYGSRIVSTKKDNEFLQKNNLVTMITHRIKNIGLTFCVSDGIKIHWLAIFAIISFVLTQGLQIIGLFNAKDDIDKVFEYFSVMSFCGMGILKLLSLCRNHKQWKILLDNIKQLEKTQCQNETSNVEYESDGENDTFTFPSYIESYTKKFKIVSTVLSRMYGFTAIVYILSPFAEFTLLIMTGNEDYEKPHVLPGWAPFDSRSFVGYLTNVAVEIISVTYCVLVHITFDLTSIGVMIFICGQFSLIRDYSSNIGGSGASCTLSKRREDRAHHRIITCHKIHCLLMNTCDELGKQLQNILGVYFSVATLTLCSVAVRLNSELSRMELASLLQFMCATLTQLYLFCHFGHNVLHQSSIGMGDGPFGAAYWCLSPRIRQELVILGMGMMMPRYFKAGPFISVDLPSFVQVLRTAYSYYAVIRK

>OfurOR30

MATFNSEDLFLSRAKFVMKFLGVWMPPVDETLPRKLFKIFMLTLQYLFLIFQTIYITQIWGDLEAVSQPSYLLFTQACLCLKITIFHVNIDNLRELLKQMGSEVFLPQSRVHEEILKTQAARIKRFLLAFMISSQIVCTIWVVHPMLQKTGPRKFPFDMWMPVSPDDSPQYEIGYAFQLLTICMSAYMYFGVDSVALSLVIFGCAQVEIIKDKILSISPVQHRLKESERKIIFEKNHKILVECVIQHQAVVTFTQLVEDTYHWYLLFQLTGDVGVTCMSALNILAQEVRSLQFVTILIYVIVMLSQLFICCWSGHELTATSEGLHTVLYQCIWYEQDLKFKRDLRFVMMRMSRPMVLRAGHYIGMSRQTFVAVLRMSYSYFAVLNQANRVEQQ

>OfurOR31

MKFFVVNMYTDLKISLTILLYTGFWTRQKEVTNILSYCYPFLTFMFMAGISIIAQFVDLLHVWGDVSLMTSSSFLLFTNVSFGLKMFNILWKREEVRAIIDDCDQVLRAVDTSWGYEIVKSGIRKSFLLFSIYTFLANISVFGWAISPEKGELPTRAWYPYNTTSSPGYELTYLHQVSAVLLGASVNASLDTVVISLMAQCTCRLRLLAAALRMLGGDMLVTNMFEAEQERAIREELQRCVQQHRSVLQVAGLLQQYFSTSILAQFSVSLVIICVTAYQLAFVSSNVLTILGMTTYFMCMLMQVFLYCYQAHELSTVSSQVGDAVYESLWYEMSAPLRKDLLVLMVRSQRVIKITAGGFTTLSLNTFMAIIKTSYTLFTVLQRED

>OfurOR32

MKIIPKGIIEKCTRTVGDRSEIDEVMRAILITQRAVGNQILDPYWSWMKSLPHQLVLGAFMFYVLIGTWEFVGGTDDVKLIAEGSFTYIVAALIPSRYYFFLMARKDFQKLYIAFKTTVCKFITDDSEEKMEQLLKKTRSLVKFMLFSSNFPMAIYFLAAMWHYVQGEKRTISKTTSILMPMRSPYHEIGLFAHSFFIFEAGFLILVPDMWFVVIMLFFCSACDSAAKFLIVEERRNESKLQYATRLNDSLRKFYVAHVKLIDFLDVLNSVFKWLALLPLISVGVGICIILLLITQGVDWAFLSNILPVMGELFVYNWFGEQIINKAEKWSLALLNFDWINLSAKDKKCYYIMVSYMQKKFRMKTAIGSEFSLLTMSTCVKGGYQAFTVLQSATHKRE

>OfurOR33

MEDTIKVFHRVLSFAGILIYAEGNWDSKLWLAFQIFNFIIGSLSFIFTTGFVVVNCSDLLIFIQGACIWTTGVIMTISLGVCLIFRKKFRIFLGEMVFKDEILEMPLIQFVLKLESGKKLIELKQMVNDSQEKLFRLTKVLLKCYVTSVWLVATMYLCSPIYEMLSRGDKSLRLLAFDMWFPWSLENFKVYVISFVFHAYAGYLCCVAYPGLQLTIILLIGQVIRQLRILTFVMENLDELVMEIIKEKGDRWQMCCTAVLSQCVDHYIKLKRFSNRLNVICQPFYLTLILVAIMLVCMCSVKIAISEKLSPDTIKYYVHEFCFIMVVLMFCLLGQQVDNECANLERAVTENWYIFDKKHKIHVRIFKMALSQRMRVFIFGSIPLSLPTFTWFIKTGMSFFTLVMSVLED

>OfurOR34

MTSTQANGNRVYSRNDYDETYKLIITNILAKVGIRMTRKDSKYARLGWNVFFCFGFGNMVVTLFLDLVTFQDVVRSGVGEDGYIVFMMLPCMGYMALAMLKTYKMVYKRDVFENLISELREMWPEGLVTEEEHTIISRALNELNIIVKGYYWCNLGLGVSFMAPSFVVAIRRIFGADIPPSLPYFYWLPYDQSQPVAYEFTLVMNTYHTLLTLWYMLAGDLLFCVFLSHITTQFDLMSVRITRLFQVPVDQQLIPEYPLGQQIKDFPENGHLPRLSNNEINSKQENELQKIIVRHNALIRLSGDVEDLFSFAIFINFFNSSIIICFCGFCCVMIEKWNSLMYKTFLATSLSQTWLLCWHGQKLLESSERVADALYNSGWYTAANGIKKSILIMIHRSQKNVYVTTYGFSIICLASYTAIIKTAWSYFTLLLNTYNP

>OfurOR35

MGIVMENVKKRLTILQPILPYGVIEPWDDLNPRLYHAIHIYWLKFYGMWYNNASPKTIVFWLQLIYTATVLWLVCFLPGIGEVVYLLKRRGNIGDVAEGLYLFLSEMYTYFKVAVFWLNKDKVINLLRYLSCEEFKPVEMEHREIIRKSIKAARFVMTYYSTMCVGAVSVGIIMPLTENFDILPTNVEYPYFDVYKSPVYQTLYIHHVYYKPATCIIDGVMDTILAAFVASAIGQIEILAFNLRNFDVVAERRRKRAVAENKPSAAWTQERHIRAVLKDCILHHNSIIKYVSMIEGTFSLASALQFMLSVMVLCLVGIQFLSIENPSSHPMQIMWMAIYLTCMLIEVFILCWFGDELIWKSTALRQAAFDGPWLETNHKTMVFIVIFLERCKRPLRVTAGKIFTLSLDTYTILINWSYKAFAVVSNMKK

>OfurOR36

MKDYEILKKHCKRIYLIGSGDFWYEDGTIGDDKSWYYKVYSWSLLSVYGFMTILEIMAAMIGDYPEDEKRDSVTFAVSHTIVMLKIFSVHSNKQMIKAMNKNMVYICEAHEEPTLMAEKYKIVKINVLAYFSIVYGSGLFYVFEGIRKIFAGSHFVTIVTYPPSYEDDSLYSVAFRVSTTVILFMLLLTMIVSVDSLTMTYLIMFKYKFITLRNYFERLTEDFYKMNDVNPREAADKLTNGLVEGIIMHKELLRMAKDIDQAFGTVIALQLCQSSGSAVSLLLQIALSDQLTFVASMKIIFFVAALFFLLGLFLCNAGEITYQASLLPDAVFYCGWHACARQPPRRSARRIVLLACAQAQRPIVMKAFKMIQLSYSTFLQVLRGTYSVFALFYAQNK

>OfurOR37

MIVKNVTTSVSMSLTALRLVGFWMPEHFGGNKRILYDCYGFFSFMFLLGTYLIIQAVDMCMIWGDLPLMTGVAFILFTNLAQATKIFFMVWRRKQVLTIIRGADEVLRAVESDEAKAIVKSCSRETTFLHIVYNCLTLVTMVGWGTSAEKNQLPLRAWYPYNTSKSPAYELTYMHQIGALCVAAFLNVCKDSLVTSLIAQCRCRLRLLGLSLRSLCKDLHATGKQYTAEQEAIVRARLCACVREHQAALVAAQQIQDVFSEQTFAQFNVSLVIICVTAFQLVSQTGNLVRLMSMGTYLVNMMYQVFLYCYQGNQLSEESAMIAGSAYECPWYLMSISLRRSLLIVMIRTRRVSKITAGGFTTLSLASFMAIIKASYSLFTLLQQVEGKK

>OfurOR38

MENPGTSDETNKISRFFFQVCRLIYLLGLPNFWSKDFIFSKSFITFYDSFTKIMNVTTYVFVVTEWGAFYTQQNLNEKQHSDRLVFCISHPILASYRLILTHHGEKLKELVYLLCLDLKEKANDEKIEKDTIRKAIIYATALVGLCIISIVSYGIDALANSISSDATFTTVVTAWPDVGDRSTLAGMARIMFYVIWWIFMSRVFGAFIIMISIIVALEHQYKNLGNYFRNLSKVFEQNLSPIEKEKKYEESLKVGIQLHAKTLKCIELAQASFGPIFGAQIILNTYVIVLLLFQMVSSERTLGNVLATIFTGLAMLLSTGFLMWSGGDITIEAASLSTDMYWSGWQNCQASTTGTRKLLALAMLQAQRPVMIKGLGVITISYPAYLSIVKSAYSVFSVLY

>OfurOR39

MDELRLKVMVWSGIYKLHTKNRFLGICHDVYRVLMILYMSIYTVQHFVFIYMNVTRGDAINWQVAVFSIGMLNMVVKGITIYMHPESIDEIHDLIKDPMFAATCKEDEDIIKKNEYHIGLFIKITYVTLTVCLFFWVASIIVTRLVDDTAMPPSYFPFATNPWPQYIIATFVETVGSVLWFGYGHFSIDCSVACYYGRATAQLRIIRYHLEHFFDNGGAQGRFQYKDVVDRSLDEKFVYYVQCYQYVNRMIDNVSDAFNWGIAFHLCIVTTGMGMCVFIISTRDIFSLDMLFTVTIFVLLLLQNFMYCYCGDLVKSESDQVCTSMYFSDWTAVSPRFRRKMLIAMTRWARPIEPRVTIVPISLTTFASILRFSYTLYTMMKTRTM

>OfurOR40

MHCLLQMLNENIVRVIFIKVNSTYYPLSVKIPKTMNNYDILKNHCKKIFFSGSGDVWYEEGTIGDDKSWYYRLYSWSLFSMYMFMTILEIMAAMFGDFPEEEMRDSVSLAVSHAIVMLKIYFLYSNKNLLKTMNQNMVRICEAHEEPSLMAHKHRIVKITLRVYFGIVYGSTFCYVIEGIRKLFDGSHFVTVVTYYPSYEDDSFWANGFRIFNTIVLLMLMMTMIVSVDSLTITYLIMFKYKFITLRHYFKTCSQDFFKLNDVDPRLAADKLTDGIVEGIVMHNELLRMVKDFDQAFGTVMALQLFLSSGTAVSLLLQIALADQLTLVASLKMIFFVTALVFILGLLLCNAGEITYQASLLPNALFYCGWHACVWQPPRRSVRRLVLLACAQAQQPLVIKAFKMFELSYGTYLQVLKGTYSLFTLFYGQNQ

>OfurOR41

MNLLNFFKKYTEDDLINIQEHHFESFNKTYQWIAFTLTLGIMFPNPATDRFRIISINVLLVCVFPLAMMVLIDMYKCWMVKDIFNIIRHSTIVGPFLGAFFKMFLMYYKRAQAKEILDEINRDHASFNFLPRKQQDIAFLNVKKGVFNVERLWAPIVSIAIMTFPGMAVVMTLYSYAFSDNPKRYMIHEVKPPNSRDPEDMLKSPYFEILFVYETGSAIICVLNYTAYDGLFGIATNHACLKMSLCCMKLKEAFRCDSTEDMYKGILTFIEEQKKMFRFVDLIQDTFNIWLGTILTSTMIQIGSLLFHISAGYGFDLRYTLFSFTSVVHIFLPCKNAATLKDMSTEMSTMIYSSGWERSRERRILRMIPFMVARAQVPNYITAFNLFIFDMELFVFILRTSYSMYTLIRS

>OfurOR42

NRLSNRLYRIYQYSLFIGSFIFLISTGIGTYMSKDDVIRLLSNVDKVTITYNYFFKIMIFLIKHEQIKSIISSILHSGDQIDVNRKYLMKIHVVMVTLLVTSITGAFQFLAQIKGELIMDAWFPFEPKKNKLTFLAATLIISILFVLPFMFRAIAIQGIVCSVVMYLCDQLVELQRRLKALKYSVESETYLREEFKDILKKHIRLMEYSKSIKSAFNEFFLVQNLAITAELCLNALMMSLIGLEQKNHLVSFMAFLMMALFNAFIFCHLGNNLIDESAGISLAAYESTWTSWPVDLQRDLLIVITVAQKSLSLTAGGIADMSMQTYAQALYNGYSIFAVLRDVVN

>OfurOR44

MNIVLVTTTNVYRTSCTKFVQLNTVAPSHSKIDSILNESSLFHVTMTHTFLHRPKTALTMLGLWLLPENYKVPYLIYRSFQLSIQFTFLLFNFIYMGVVWGDLEESSEGFYLLFTQATLCLKSTTFVMNRTRLIRLLRFMESDIFATNTPKHKRILAVQAVKMWQVYMFFMTCATCNVLEWAVVPLLESRGPRVFPFKIWMPADPAMCPDYVYTICYVYQAVTVYLSATTFLTIDFMTVSMITFASAQLEIIAEKIKQIPPVATSSENLKAEEVKSRVQHNNKILNECIQQHQAVIRFVGLVENMFNVNIFFQMSGTVAIICIIGFRITIEPPNSFHFYSTLNYLVTMVAQLYLYCWCGNELTERSQVLRDTLYTSQWYEQDRRFGSTLGIAMECMKRPIIFRAGYYIPLSRPTFVSILRCSYSYFAVLNQANNK

>OfurOR45

MDIPTFEELFKQIKLNLWFFGIPFNGRKIELRFYFMVVVVIIMLIGEISFFVSRYAPENFMELTQLASCICVGALSLLKILPIAHKKQKIFELTESLDGLYNTILENPKKKAIIRRQMILVKILMKYLFIVNIALFVIYNISPLIFMTYNYIATNEVEFILPFALGVPFSIESMATWFPVYAYSVFSSFVSVSYFVTVDALYCILTTHICSNLSMVSEELQNVDTSNEDELKELVKNHQYILKLSENLEEIFSLPNLFNVMMSSLEICAVGFNLTMGPVSEIPRSVVFLSSVLLQILMLSVFGEKLIEESTKVGDAAYNSKWYEVDQKTKKTILIIMTRSSKPQQLTAYKFSVISYGSFTKIISTSWSYFTILKTVYKPPE

>OfurOR46

MLLKKWKEFYNKEDFDYSTGYVDPYQFHRTFYFVQSAFQVTDEPFKPWTYVSKTITLICGIGVLTDACFSFYHAIDIFDMGLITEAGTYVLMLLYKMMTLTITKVNLPSYIHFMKCMKDDFGYICTKSEKYRKVFFETQLATWKLCVSTCLFMFCLANSLVLFAIGSLFFYLATHEPGDGTNRPLVFPFWAPGIDYTSSPAYDIAFNFANIGVLACTYNYTFVLQTNIVWVRQIASKAEMIEMCITDLLEGIQPANNEEEKRHYANLINYRMREIISQHQKMLLDSYASVFKKCLMFEQLVSSPVICMLAYCSAEKLDAGEIHTVMILLCVGAILILFLPCYLCTYLRMKVTRICDACWEIRFWDAGPNIRPYLILIMQRCLRPLPLQAPGFQEVSIKTFSSKMTSAYSLFNMLRQADLDL

>OfurOR49

MFVYHGIEVEDYSFATECFCYFVMLSVIPILYGSVLMNNRNVVLLLDKMDKDFKFICKLSFKYRDHFLKRQLLIWQLCFTWLGFLCCVAVLYVLMTLAPLTYQSLFATQDEHMIRPLIFPMWLPKDDPYRTPNYEVFLFLQMNFLLIFIQSFGVYVYIQFHVLLHNFILLELVILDFDVIFEGLDESVVGLSRYDPRRASIQHVFNKRIERIVTWHDSVFKSIATLSTVQGPVIVYQVMFSSLGICLMMYQVADKLDSGTFDILFMLLTVATTLQLWIPCYLGTLLRNKAFDVGDACWNCGWHETSLGRMIRNEILIIIMRAQHPISIKFTGLPNLSLETFSSIMSSAYSYFNMLRQYNK

>OfurOR50

VFVTLTTALHFVFYTLNLIYTPRKIEIFATQAVYYFSSVSGLFKIGTVLTKQQEILSTFEIIDCKEFLGNCSETKKYLQQFRRTYFRYFRLYLTFCMCCAVVFLCGLPLVNYFFRHEDLKLPVCEYYFLTDEVRKNYIFYWFDYQILGLIVTIVYNSTTHTFLCGLILMGITQFKILNFNIANIRLDEDIEINNQEEREQALINKLNQCLKHYDIILKYCENVQNIADVFFFVQFSLAAITICFCMYMLILDLSDKDKVFTTCFISAMLLENYTPSFLGSHLTAESDNLRIAAYSCNWTPRSHSFKKSLILLMERAHRPVVIVALKMVPINLETFASMVKTAYSFFTLLSGAQE

>OfurOR53

MFPKTLAPTKINRNNFKFDNMFVVTAMAMFINRSHPSIPRNLFWVFQFLIILTLSTTTFLFLGNSVLLYDIPAGRYAEASKNGTMAIVAFTITIKYSFLLYFQKYMKNLISVVDRDYKLAMDFEEEEKEIVIMYAKKGAKVSWYWLLAALSTSIAFPLKAIFKMGYSYWQGDFKYIPMFDMRYPDRLDILKDIPAMFILLFVLCLMFGCYATTMYIGFDPLVPIFLLHICGQLDILSKRILKIFSENYNEEEINEKLKNVNIKLQDLYGLIENIKNKFTVLFEYNMKTTTFLLPLSLFQVVEDLKRSQLNMEFLSFFVATILHFYMPCYYSDNLLERSYYLREAIYSCGWETHPNTRARKTVLLMMTRTTAPLVLSTIFYTICLDTFAEMCRQSYAIFNIMNAACA

>PintOR1

MMNKVKTQGLVSDLMPNIKLMQAAGHFLFNYYSDESGMSMLLRKIYSSVHAVLIVVNYVCMAVNMAKYSDEVNELTANTITVLFFAHTVIKMLFFALNSKSFYRTLAVWNQSNSHPLFTESDARYHQLALTKIRRLLYFICAVTIFAVISWITITFFGESVRLIANKETNETISEPAPRLPLKAWYPFNAMSGSMYIVAFAYQVYWLFFSMMIANLMDVMFCSWLIFACEQLQHLKAIMKPLMELSASLDTYRPNTAELFRASSTEKSEKVPEATDLDIRGIYSTQQDFGMTLRGAGGRLQTFGQQNTNPNGLTQKQEMLARSAIKYWVERHKHVVRLVTSIGDTYGTALLFHMLISTITLTLLAYQATKIDGINVYAFSTIGYLSYTLGQVFHFCIFGNRLIEESSSVMEAAYSCQWYDGSEEAKTFVQIVCQQCQKAMSISGAKFFTVSLDLFASVLGAVVTYFMVLVQLK

>PintOR2

MFFRKKQHKSTINTEKITNYTHFLEIPLKIVGCWDWYKEPTREWQIIINNVYVCMVLFVLASIPMTLFVNLYIEWLGIMSSLDELADGMPSLVSLAVVSYFVIYRKEMYDLTGFMRKNFEYYSANGLTNMTMLKSYRAAKNFAYFYTACTMFSVTLYVMPEIINLWMKMPLQNRIYADITRAPFLVFAFIRQCMGQAFIGLAMGQLGVFFASNSILLCGQLDLVCCSVRNVRHTALLKGGATHSALTAQADVLLDEMHSYMYNTAEMAVSGYNYGETMYAHFVDKTAQIDIYSSAYDAGTVDALRRCARVCQVINVFKDKFENFVSPLLALRVIQVTLYLCTLLYAATLKLDMVTVEYLAAVALDIFVYCYYGNQIILQADRVSTAAYQSAWHTMGVRPRRMLLNILLANRRPIIVRAGRFLPIDLHTFLVIIKTSFSYYTLLVNVNEKHA

>PintOR3

FVIMEAEAVARPTRYFAFHFYLLRFLGLGWWHQPDENDTRNFPSWYLYYSIVTQLVWVAGFVGLETIDPFVGKKDIDRFMFSLSFVITHDLTLIKLYIFFFKNDKIQEIVRILEIDLYGYYQNNAKNRTTIRTTRIMTSSFIFFGWLTIGNTNVYGTIMDLRWKAEIAKINDTALYPSRTLPQPIFIPWDYQRDFSYISTFVLETVGLLWTGHIVMTIDTFIGSLILHMSSQFAILREALVTAYDRTILQMYENVRFNADGSSLITIDRRMDIDDVDKIVRTRFQDTEVEKQIEKTLLSCIHQHQLLISCVENFARTYSYGFMTQLLSSMAAICVVMVQVSQDASSFKSIRLVTSLAFFIAMIIQLALQCFTANELTLQAARVGDAVMQSRWERMSPRLRRLLMMVMMRAQRPLRLSAAGFAYMSNDCFLAIMKAAYSYYAVLSQKQA

>PintOR4

MKILTENMKKMLSPLQPAIPYGVLESWEDLNPRLYHAVHIYWLKFYGMWYNNFSPKNILFWVQLTYTIVVLWLVCFLPGIGEVVYLLKRSENIANIAEGLYLFLSEMYTYFKISVFWLNKDKVMSLLEYLHCDQFKPTEAEHREIIRKSIKTARFVMTYYSTICVGAVLVGIIMPLTEKFTILPTNVEYPFFDVYKSPAYETLYVHHIYYKPATCIIDGVMDTILAAFVACAIGQIEILSFNLRHFDLVAQRRLQRAIAAKDPSGAYHRDYHIHSVLKDCVRHHNSIIRYVSMIEGAFSLASALQFMLSVMVLCLVGVQFLSIHEPAKHPMQIMWMAIYLTCMLIEVFILCWFGDVLIWKSIDLSHAAFEGPWINTDQKSMKYIVIFMERCKRPMKLTAGKVFTLSLDTYTILINWSYKAFAVMSNMKK

>PintOR5

MLLFSDGSDLRGITRTQDIKYMKYLRGTLSIIAAWPGYVIGESKGIGVGYKVYMDVIALVSLVCEALYVRQNVGKISFFELGQTYITTSMAVMCAYRTLAIWRENYSEIFKKFITVIHLFNHRNKSAYAMKIHLYVHKMCHFFTIYMIAVMVWGIILYNIIPLSNTIAAGGFQWPPPDNVTYDVSIFLTLPFDYQHSLIGYIVVFIFDWYECYLASSFFCIVDLYLALMMFHLWGHLKILVHNLEHFPRPAPRPDNMTQSIELCERFTDEEMQIVRQKLKEIVDHHNLILNYTDEVSETFGLSIALSYAFHQVSGCVLLLECSQMEPKALMRYGPLTLILLQLLAQMSVIFELISTMTDRLVNATYDLPWEFMDSSNRMTVLIILRQIQNPLGLKAVGMVEVGCRTMATILKTSISYFVMLRTMTMME

>PintOR6

MWKKIKAYYNKEGYDYSKGFGKPTEFHSSFYFMMQSFQVMHKPYPKFSYFTKSFTMICGIGVFIDAYLDLYHAVVMDIFMLTEAGTYAILLTYKAMIMASTWINKDNFLHLLQAMTEDFEYINTKNERYKKKFFETHLGTWKATLWCYFFMFALGMGQIAFACLYLMWYYMTHTAGDGSVRPLVFPFWAPGIDYTKTPAFELAFVFANLGVAAYSFHYLFILQTDVVWIRQIASKAELIILNIHDLLDGIKPAATVEEEEHYSAMIRFRMREIISKTQSMYVLIENYAAVYKKMLMYEQFFSSPTICMLAYCSAEKLDKGEIQAQMMLLCTGAMITLYIPCYLITFLRSKISDVSDACWEIPFWDAPPVTVRVYINLIMQRCLRPLPLQAPGFEEVSIKTFSNKMTSAYSYFNMLRQADFEL

>PintOR7

MKIAAKDAKTPMDLRYIKILRLALRTIASWPGQELGEHVWMISNYYIYYLLLSLLIALIPVIRYVIQNAKTMHITVLGHICISVLLTFTSTAKIPLLCLRKEYRKFGKMFFTEIHLVYTKNKSEYAMKTHLRVHKLSEYCSIYLISMLSATIIFFNLGPMYVNYSQGLYRDGIPENSTNVYAHAAYYAGFPFQCDYLRDFDCYVVVSLFTWYISYFVGTFLSILDLYIYITVFHIWGHYKILINDLETIAEPNSNGKYSAEESHNITERLKNCIKYHHIISEYTKKMSNLFGATLFANICFMQLVSCLLLLECSYMTAETTMRYGPLTVMVFQELIQFGLVFELTGTAGDDLRKQVYNIPWEYMDTKNRAMVLFFLMNVQKPVRVKALGLTEMGLATVASVIKTSFSYFAFLRTID

>PintOR8

MDETFEVFHRVLSFGGISIFTKNNWDSKSWLLLQIFNFVIGVFCFIFTTGFVMENYSELLIFIEGACIWTTGVIMTISLGVCLIFRKQLRQFLEEMVFNDCVLEIPIINNVLKRDEGEKVVELKTMVVESQEKLLKYTRVLLKCYVGSVFLVATLYLCSPLYEMFVSNDDSLRLLAFGMWFPWSLDDINIYIASFVFHAYAGYLCCIAYPGLQLTIILLVGQNIRQLRILTFILLNLDELVIELGHKNDNWQMCCTDVLAQCVVQYAKVKSFSNRVNVICRPFYLTLILVAIMLVCMCSVKIAVSDKLSPETMKYYVHEFCFIIVVLMFCLLGQQIENECEKLEQAVTEKWYIFDRKHKINVRIFKTALSQRMPIYIFGTIPLSLPTFTWFIKTDMSFFTLVMSVFEKQE

>PintOR9

MEYLNKYPEEYARPLVSLFAYLGRLNVKFFNHDMPWFYRNWRFFYIVPILVFHFITMSVYIVEVFVEGVDVFTDVFMIPIYLVHIHSFSKMTILNINKERFEKVIDELGQTWRTDNLNDVQKDIKRSSEKELWMVQLVFMKLPICVASQYVLLPILDTLFCNVILREDDEVRLPLRCSYPFDPSSSWVLYGITYCFQTYCIFNLTFSYVATDFIFLCLSAQLSVEFMLLREDLLHIKPTKVVDNDVTHIVYDNGKIRDFVIYHQKLLRLAEELNCASNKIMFVNLFFTAVNVSLFGLAVLVANNASDKITNLIVILTILVLILTVCYSSEKLKSASEEIFNSACENLWYEGDKEYKQIILFIMKRSQKPCYLKSLDVPITHATFTTVMRTTWSYFSLMNKMYENEETN

>PintOR10

NTFAGELHAMAERKSLKEVYPQKYSLKLICRLLYYSGFGDYWYEPMDRSALEKRLYPIWVVISNGFLAFGIFNEFMAYTRADLTEKEMNDLMQHTFAHSSVYSKFVIIYLQRKRIRAVLKRFVEETRSIYSSSEIDKAATNKSFRFCFAMGFILFVTLTSAEIDGVRAHFQEDIPIRGEVIYYPSKNQTGILVNLMRFSAEFHWYYLVAIMTVADSLAICSMIYIEAKFKLLQLYFQNLGNTLKDETKGITEREEEYKKRFIDGIRLHEDALWSANSIQYALGTIYSVQIFESLSLLVVCLIKLVASELTLLRVIAILAYTTCMITLTGTYMIAAGDITHEASMLSTSIFHCGWELYLKKSEILPLAVVAIQRSQVPVYMTAFGVIELSYVNFISVLRSSYSFFAVMY

>PintOR11

SDATYKLVKHEFILELDYLSYIGRKIFIHPFLNRSKTSVTCYAIITVLISLTALQLFITLCVTALTTTNWFELINIAPNLGVVLMTLTKYYKIHQHRSLYEEIFEHYRKDIWTVEMADKDYQNIISQYTKITKIVTRFLFYYCIPLIIVVNSFPRIMMYYEVNVAKKEKEYLLPYDGWYPFDKINWYYIAYLWEGSMTVLVIGIVSISNSIHVAYTYFICMELKVLGQSIENLITVDNVNDLIENIDVRNTLRVINNELKDIARRHQVLAGLSSRLNTVLGDTMLLTYFFGSVFMGLTFFTATVEDNIYKALRYFFMCCSLVVDSFYQCMIGQVLINHSENLAHSIYSADWTYADSNTKSSLLILMIRAQKPFQYTAKGYLSMNLNTFSAICSLSFQLFNLVRRVYE

>PintOR12

MALTFQSTLKRTKIALLLSGIKLEDVKFGRILEFLFKNLFYFNFFWLGTDLVAETFGFIEGVQMGKNVIELALTAPCFSISLLGTAKSIFLFKHQNIVYKIILKLKNIHPESATENDKLIAIDNSDVKAIGTTEIDEIDNNNIEDKIVENSMKILNQINAVFNLFCSSVVIAFCLLPAATMAYTYYTTGDLVYEYPYLTKYFFNHYTLERWPLVYLHQVYSTAIVAANLLGADSLFHALCLYVEMHFQILCKRFEKSTVGDENTVHNTFMACVERHQELIELVNNMEILYTKSTLFNIVMSSILICLNGFILTVTSDVTIMIMFLTFLSMNLLQIFLLCYFGDLIMKSSVAVSSAIYNSPWYNTYSSSKKNVLFVIKRLKYFLSSNILLVSEVVNRELQYLFVYF

>PintOR13

MIRDKLTHFVQFLENPKHPLLGPNLKGLSFGGMWLKGPNLWICFQKFVNFFTFFFVVSQFMEAYNIRNDFNRLLQNLSITILSFTCYSKCMAFVFRLKRWSELFHSISEEEISCIQSGDKKVIVLMKEYIKYSRFITYSYWILVSVTNAAMMIAPVFKLFSSANYRKEVQQGILPYPEMLSSWFPFDNTKMPGYFFACLVHVSMCSKGAAITAVFDTNAVAIMVFLKGQMIILEEKCKNIFHNCKSRKEALRRIKECHRQHNFILRTHQTFDALLSPIMFLYVLICSMTICFSVVQYVSAGVTISYKIWIIEYTIAQVSQLFLFCWHSNDVLLESKAISESVYLSDWWKTDKILQKQTLILAGKMNRVVIFNAGPYTTLTIPTFIEILKGAYSFFTLFSQIQE

>PintOR14

MDTTRKVGIKIANENKFKTFSETYKLCAFALAIAYIYPNKATARKRLKMLLITIALNIIELYWYMCYLIVCIIRMDIYNFTRQITIGIIISLYLFKAFYGIFVTEKFEHILQEITDDLLRGNELSMDLQEIYFIYIKRAKIAQACWTVIPLLLGTQFTIYSAVCMIYEYMTTDLWNRYMVHEMELQHIQHLQYRTPYFETIFAYNCIQSLILAPNYSGFDGSFCIATTHMCLKLKIVGYSVQKAFKDINDRKDLRKKMKSLIEEHQKALLFHRKMQNVYGEWLFMVFLLTSILISFNVYQIYCIGRIDPKYTIFTLSAVLHMFLPCYYASDLIQANDEFQRDIYNARWETTGDPVVSRYLTFMIARSQQRLILTGKGIVTFDMQLFVTILHTSYSFFTLISTK

>PintOR15

MDYNIILYSYCKNIYLVGSGNFWSNENEVGDDNSISYRLYSLSLFSIYIFMTILEIIAVLYGNFPEDESSDALTFAVSHTIVLIKMFSVISNKKLVRSMNESMIKVCKKHEQNELVLEMYRTVKINVIGYFVTVYGASVFYVFEGLRKMYSGSHFVTIVTYYPSFEDNSMVATLFRVFTTAVLFMMMMTMIVSVDSFTMAYLIMLKYKFKTLRHYFERLRLDFDKIKKSGDQRLAAEKLTDGLVEGIVMHQKLLKLGKNIDQAFGMVIAMQLILSSGSAVSLLLQIALSDQLTFIASMKIIFFVAALFFLLGLFLCNAGEITYQASLLSEAIFHCGWHACPPLPPPHRNHQHLVRHACAQALRPPIMKAFKMIELTYSTFLTVLRSTYSVFALFYAQKK

>PintOR16

MISKFVSNLEDKRRPLLGPTLWGLKFWGLWQPEKGIISNIYNLIHIAAILFVVSQYIELWNIRSDLTLALQNLSVSMLSTVCVVKASTFVCWQKSWREIITYVSSLEVSQLSTKDKNTERIISNYTKYSRRVTNFYWNLVAGTVLTLILAPLATILISSRNGSTSYPEIMSSWAPFDRKNWFGYWVVYVEQAMSCFYGGGIVAAYDTNAVVLMNFFAGQMELLSINCSRLFENSQELSDSEAIRKIQEYHMQHLLLIKHIKILNSVLSPVMFLYVVICSLMICASGIQITMESTTKMDMIRIIEYLVALIAQLFLYCWHSNEVLVMSSKVDDGVYKSDWWKRDFKIQGNILLLGGQLRKTVCFQAGPFVNLNLSTFIAVIRGSYSFYTLLSNKDS

>PintOR17

MKWFVKNQTQSLAIALTALRLVGFWAPESLVGRKKTIYDAYAGFSFMVLLGIFLIAQSIDLFVIWGNIPLMTATAFILFTNLAQAAKFINLAVREKKIRAIVDSADTVLRDADTHEAKSIVKNCDRQTRIQLVAFFTLTLVTITGFATSAEGANLPLRAWYPYDTTKSPAYELTYAHQVYALFVAAFLNVAKDTVVTSLLAQCHCRLQLLSLSLRTLCDDLPVTGLNQLTPHQEKSLKSRIQRCVVHHQTALEAATLMQKYFSEPTFAQFNVSLVIICVTAFQLVSQTDNMVRLVSMCTYLVNMMFQVFLYCYQGNQLSEESSEIANAAYLSPWYTMSPPRRREILFIMTRSRRIARITAGGFTTLSLASFMAIIKASYSLFTLLKQVDDTN

>PintOR18

IDLLCTRVRKMFSKIKQRLCLIKKCLTENTCENLLKIVMTVPNLAGFSILKDKVAVPFWIIHISLLTYVYGVGSIVFQTVDAQNVGDLVQNFVSVSILFFTGNISYWFLRKRPLLKELVRKVREADQMARTAEFLLEKHERALGMIKIIILTFYFLNCSNALFVYIPARIDVSSQYSMRPCVGMPLNTSIGRHICQTILLAQELSIMTVVLNFQALLVLLVAHTATMYQMMAEEMISLASTEAIDSKQRLPFMIKRHVLLLSIVDNLKTLYSVPIGINFFGNAICICFFFYLPINSWLPFMPILVYCFVVFFLYNVLGQRLINATEVFERGIYACGWESFELKERRAVYVMLRQAQKPVELLAADIVPVNIYTFATTLQGMFKFVTVVKF

>PintOR19

MGAYKQIDCFNTNIKFFRFLGLWPTDSGKYYRYYALSFIIFFIVIYDFVYTINFLFLPRELVVFIDDTMLYCTVISIMTKTFTFYLLHGKIIEILSVLESDMFQHDSDEAKTIIAGAKKLTIKLWKIITTFSLVANGSHIASPILAHLMTGEELVLPVFSCDFLPISVKNLLIYPLYFYQSIGMHFHVLYNMTTDGMFFGLIILLIAQLDILDLKLRNVTTSDKFNYEKEQEAIRNLNDCIIHYDEVAKFCQLIQDVFSVMLFVQFGTASMIICLCLFRLTLPASTAYYVYIMSYVFVMIFHRAVPCWYGTLIIEKSTSLGCSIYDCDWTPRSGPFKSSMRLFVERVNKPLIITSGKMVMLSLKTFTSIINAAYSFYTLLRHMQSREN

>PintOR20

MVFRKFLSYFEDPKYPSVGPHIRLLSLTGLWHQSNSKIEKVKLYLFYLTVAFFCSQYIRCLMKFDAESLTLILQYAPFHMGIVKSCCFQKDYKKWENVIFYVSSLERDQLLKKNEQSVEITKYIKQSRRVTYFFWALAFFSNFSIFSEPYRKNHENEDGNKIYTQIFDGFIPYSSEAPGYYVSMAIQTVLGHIISAYVVAWDTLVVTIMIFFTGQLKVARINCRNIIKNGEGHENIVSCHRHHTKLVKYQKLFNELISQVMFVYLVVISVNLGVCIIQVAELQDDITALISSCQFVIACLIQLLLFYWHANEVTTESVLVSYSTFESNWTEAPASVQKEVALLGITTNKRLVFRAGPFNEMSLRTFIAILRASYSFYTLLSNTNK

>PintOR21

MPAHESNDICAPGKYYFMFNLQFLTLVGLWRNDSWPKRWLFFYDIYETTLHILPMIYIVITSIGTYQQRNELTLFLANVDKNLVAVNFITKYIIFMMKRKQLSMLITEIIHSGDKITKKCVKLMSIHVIVVTIFSLSVVTAFCSQAIMNREMIVEAWLPFDTMKNLKQYLTAFQILATMFTPSVLRGIAMQGLICSIIMYLCEQLKELQRRIRSLKFSLENEVQMRNRFKDIIKKHVRLMKYSQTMKVIFNEYFFVQNLAITLELCLNAVMMTVVGLSQKKLLISFLAFLCVALLNAYIYSYLGNELIIQSENIAHAAYDTEWTSWPVDLQKDLLIVIRTAQKPLVISAGGMANMSMQTFAQALYNGYSIFAVLADAVD

>PintOR22

MNLRKGATLTFGGEIHSLASKNNIFQLNRCILCFFQTRFITLLASRKLDDVIVDFVHDVHLFNHRNNSEYSSKTNLFIHKLSHFYTIYTLGLVSLVAAFFNVTPLINNISNGGFSGHLAANVSYEHAVYYALPFDYQHDFTGFMIIFTFNWYISFSCGMIFLNYDLIVALVVFHLWGHLKILTHSLQYFPRPGFIFGKAAENDDENAVANTETHYSDEEMVEVGKRLKNIINHHKNILKHSANISEAVGINVAMYYMFHQISGCLLLLECSQLDPKALIRFGPLTICLIQLLTTVSVIFELISSMTDHVVRAAYYTPWECMDTKNRKLVITLLRQTQIPLGLKAMGMVEVGVQTMASILKTTLSYFMMLRTVADNE

>PintOR23

IVPCLVLHCISLVYYIIRITSENKNVVDLAYTVPILMITTQAIIKAAFIIKRKGEIKQIIEELGAMWRTTDLTKIQLKKKNAVLSKIYICQRVIYISSMVGSSLYTISPLLECLLRRVILKQDFEWLLPFPCSYPFNPTETCFRYIILYIFQSYCMFMCVFSYIGCEFLMVAICSHLIAEFTILREDFLNLKPIKNSSKRQIGGEETNKIEMNNFIKAHQKLIDLSRRLDESFNKTNFVILFFATVTMCFFAFTAKVATGALFMLNNYTAVVVMMVQLFTLCYFSELLAKESSGIAKSVYENLWYESGTNYQKSIRFIIARAQRPCCLTSLKYAPINLASFRKVISTSWSYFSLILRIYEDIR

>PintOR24

MVVGQYAYIAYLLMNNVSLEQLIGSYLHIAGYDTMSFGKLLTIWYKQSSFRQVVNELADIWPVSEEDQKAVAVKDNSLTSLRRRQALYLFWNVLGVWLYNLTPVVIHLYRLGRGMSSDLGYIWQIYYPFDKTKPFVHELVYIFETFAGVASVCCMLGSDVFFISMANHISLLLRLLQERIRRLGMSDVDTNGVLITGTQDCYEQIVGVIKIHQRLISYANDLEDAFSVVNLINVLLSSVNICCVVFSIVFLESWMEMSNKFFLGAAMTQMGIVCWYADDIYRASMGVSDAVYESGWYRCNTRSRRGLLAMLQRSQKPLYFTALKFKTITMTTYTSILTTSYSYFTLLYTSYRQE

>PintOR25

MLVSDLNFQKQFFISMIPLKLNGSHPNIPRNRFWVGKTFVLMINLAIIMWLLFNAVFNHDIPDRNYSDMCGNSIMLLIGTNTIIKFWFLHYNRKYMAELFQFVENDYELSKYLSEEEKEIVIKHVKISITVTKHSLFSFFNGFIFFIRAVSLMSYYTWKGQFRLVHMYDIALPGQYNERRNDPMVYFFCLYCATFFGVYASTMNTAFEPLVPITLLHIKGQVQICSNRMKELFTKNKNPEEVYREIKWVNLKLQKLYNVLEGLNTKFGPLYEFTLKVNTGLFPLISFRVVQGIVDRNVRIEFLSFFINLIFHSFVPCEYSERLLGEVSLFIFYINNTWSSRKKN

>PintOR26

MESQLSYLSLHRNVTFLKMLGIYPIDPKSSKIKKFFHGIYRYIMFFFILLYTVQEIMKIYEGRHNADKVIETIFLLLTYTDYIFKIIVIRMKSDQIEDMLLITKGPIYNQGEVEHRAPLLNTIRAGLLFVRLFNLMTLFTCFLWGLQPTVQHLQGKSIELAIWLPFDVNVNPYFYFAVFYMWAQTSLLALGNSTVDSFVVYLLEQCKTQMTILRCDLENCVEKCKFQSKETPISFSENLEIRLIKTIIHHREIVKMMNQVQAIFGKAIFYQFVVGGWILCTSAYRMVSINPVSVEFISMIFYMSCILIELFIYCYFGNEITIEVSFCLPNYNLN

>PintOR27

MDKLARVMFLLLCHVTSIVKQIVFHTKAHRIDLMVISFDEPAHGGAAPAEWRRALLEHTARSMARLQRAYAGTAVLTCTLWIVFPIMYYMRGQNVEFPFWTGVDYSHLGKFSLVLVYSYYVTTLVGIANTTMDAFVATVLFQCKTQLSILRANFETLSERAVEKSYNREQYDFILNQLFLECLDHYKKITVILSLLQDIFGSAILIQFGIGGWILCMAAYKLVSLNVLSVEFASMTLFITCILTELFLYCYYGNEVQVESDKLMESMYRSGWLGAGLRFQRALLLAMERAKRPLRPAAGLIIPLSLDTFVKILKSSYTFYAVLRQTK

>PintOR28

MVVLSLVISTEIGPIKYYYLFWNRLATRVLLDNYLSCDAKLDPESKMSKYVLQHMSKVYKRAAIYWVIIIGNGVVFCARPLFHPTERRIVDDNFPFYGLEPSTESPNYELSIIAMVLAVIFTVFPPAQVNVLLLTIVGYTESHLIAMSSELRTLWKDAEQYCEDVKSNPGPANDVDDKVIKNYYIRNKLRIINYFHVTNIDLIRETQSIFQNAFAGELLVLIVALITELLGGLENTYIQIPFAYIQVGIECLAGQKLIDAGIIFENALYDSQWENFDVSNRRTIVCMLMNAQKTLNLTAGGISLMNFELLMRINKGIYSAYTALRTK

>PintOR29

MEIMCLFITNVNFFYKIMVLKVKYDKVEEVLQITKGPIFNKGEKEHRVPLMRTIRDSLRIISSFNYISLFTAVLWSIRPVLERYQGKEINIQIWLPFDTDVTCYFYICVLYWWIQVSILAAGNSIVDGFIAFLFEQCKTQIKILRLDLKNAVEKSKVAEIETSDSFSNVLEEKLKNIIIHHKEIVKMTNIVQDIFGSSVFNQFLISGWILCTSTYSLVSANTASLQFVQMLFYICCMLIELFMLCYYGNKLTFESNRLVEYAYESDWLEIPVKHRRLLIAFMERIKRPIQPTAGSFIPLSNNTFISIIRSSYTFYAFLKNSN

>PintOR30

MKILTAQRDVYRVCVTNFMKEFHLYHFQHENKYNKKKAIKTERFSLFIQCFLILSIFLDALMWFITSLAKNTIHLEEIHNKTMILQGGTYIWMPFDHTYNFKNWILLEIFFMTYLQFGIGLVIFCVDIMTFTFMLHLIGHLDILKNDIESQDWIGLSDLAVRQKLVDIHNRYTFIKRNFKTFEAAYGINIGAIFLFNLIADSLLMYLLMFNEKENQMLYFIMAFYFFNILIALSYATEKIRKLSEDVFIAIYSCPWESMALSNQKMLLLMLVQSQELLDFKACGYSIGYQPMISIIKATFSYYVMLKETNE

>PintOR31

MDVPSYDDLLKQIKYNFWLLGIPYDDPKIYIRYFIMYISLVLMATEEVLFILSKISTEDLLVITQLTPCSCIGILSLLKMVCLTVKRKKIFSLTQSLKVLYVNIINDKEKKDVISGNIKVVSTLSKYFFVLNVVLVSVYNFSTPIFILYDYITTQKVNYKLPYQVIVPFRTDTWATWLVIYIHSVLCGFICFLYFTTVDVLYCILSSQICNNFTLLSKELLKINKNNVTNVRNIIMQHQHIIRLSEELEDIFNLPNLFNVLVGSVEICALGFNLTITLFLSTKINEVVDSATSNTKHPDTYHYFIIIVG

>PintOR32

MSEYLSYDSKADPNSKSTRRILEILNRVKTAAVTFWCIVMGNGLVYCTKPLLMSGRHLMEDTEFLYGLEPLFMSPNYEIGYILSLNACIFTVYATANISAFFIVATGYIEAQMLSLSEELVNIWDDANYYFHQTNNITVDIFIDLTEEERKDRDSIINKYVKEKLRTIIHKHSTNIYFLNKIEYVFRNCIAIEFALLIISLIAEFLGGIENTYILMPFALVQVTMDCITGQKLLDSSDIFQRAVYDCKWENFDVTNMKTVLMILVNSQKGLTLTVGGLKNLCYICLMDVFRLIYSTYTALQSTLND

>PintOR33

MYNKTAQYFMNITSLIYWLGLPDFWKRDTPRSKKIVQIHEIYCYSMVVLSVVMIIFELGAFFTQPNLNERQSSNMILFGISHPFLLTFILSTLYYIKEMRCLLYTLAVELRAEYNDKEIETSMVKKIKVYTASFISFFLVACVFYGIDALVNVVKNDATFTTVVVAWPEVHDQSPLAGAVRVLFYLVFWSMMLALCGVYTIVIATTICLNHQYKNLQSYFCNLAGIFEENISQYQKERKYVEGFKLGVTMHSKTLWLVVDTVLVRFDIIVDDHRGVRVSQFFSVRESL

>PintOR34

LKDVWLAVQLKRERERERGQPQDVMYKVYDIDGIGGDEMNEDDKLSTSDNGSPSRGGDRKNTVKSSKQILLNAGQKSEFDSGMNNCQLSQNPSRDNRNPNSDTTYCTPVYGQIVSSRENHNQINDIQQKFSQNETTKPSSSFRKDWNPVHGKTTEGWDEFSKRNISSISSEDDAKKQKCSKNEKSNKPVSNGKHYDFFPVFDNKSADDSETDEKMPSPERTSKDKDIRNEDNSKIIIDPMQKLVLHSTDNKICDNKTSALSSISS

>PintOR35

MEIKVKDVASMQFKPFHETFPYCTFGLALSSIYPNKYNIKKRILTFVTIITFCVFQIFWFLTYTINCLLKLDILNFARNMTLWIIVILFFFKTIYAIKYTEDYAAILNMISKDFVKANEMDADYQEIFKTYLKKSKFGELVWLVIPIILSSQFPIYPAILMIMENLETDSPNRYMVHEMDMIYVEHIQYTTPFFEIYFAYNMTQCLFLSPIFTGFDGSFCIATNHLCLKLKLMTHKVRRAFKDAKKLGAIEK

>PintOR36

ALILPFLDIYPFDSFDLRIYPFLYFHQIWSEAIVLFQVCSADFLFYTCCTYLYKQFTHLQYQFADIISQNEVKSNPDELRQNFTILIKWHQDLISNALKLEVITSKSVLSNFLMSSLVICLTGFNITTTDDFSAMITFMTFLFMSLIQVFFLCLFGDLLMKSSSDVSDAAYNYKWYLSHVSTGKNILLIQTRSQTPCKITAAGFADVNLRAYTKVNNVKTVIAVFSAAGSILLDTLPQG

>PintOR37

MATVAFVVCFFLQTIELVTAENSARLFECFSILSFCSMGMLKLYFLRIYRKIWLHMLAQMRKLEKHVGHNEMPEEEYESDDDTLDLTVFVKNYTQQFESTSTLLSKIYRSTVTGFITSVFVEHALLKGTDRWHILPCWSGFDHVHVAIYMATILMEFIASIYCVSVHLAFDRSAAGVMIFVKGQFLLLRRYCENIAGKGRKCNISNNRDRRALYRIKYCHQRTLILNE

>PintOR38

MYTTVIIVIMTPIFKYFFSPNYRDNVKNGTETYLEVVSSWVPFDKNNFAGYLIASVYQSYAAIYGGGWITSFDTNAMVIMVFFKTELELLRIDCGDIFGSEKVRVPDKMISERLKNCHKRHVELIKYSTLFNSCLSPIMLLYMCVCSVMLCVTAYQITIEKSAAQRFLTTEYLVFGVAQLFIYCWHSNDVYFASQDLRMGPYESTWWLHRVHRKDLFIL

>PintOR39

MNLSNLSTLFIITVIVVFVYAGAADYLFYTCCLYISIQFKMLQLQFEILIPDDRSQIRLKFEDVENKFVELVEWHEKLISLTNMLEVVYGKITLYNFVTSSVLICLTLFDVTVIKDVAFALTFLFFLSMCLVQISLLCYFGDKVTQASTEVSDAVYNSLWYKCDISVGKHLLIVLMRSQKPCKLTALGFADVNQRAFMRIMSNAWSYFALLKTMYH

>PintOR40

AGTIADVKMKLLVSNVTWSLRLSLTALRFGGFWWPKADGTWKILYNVLTAFSVVYMIGIYLIVQTAEFICVLGDVSQLSRVGFILFSNLVLGIKMYNLVIRADEIRGLVDEMDRRLRDAKGDDEIAIIKSMDKLTMWQLYVYCFASFNTVYGLAISVRKPYLPLPARYPFESTISPAYEIVYTHQVSAITLGATINMSLDTLVTTLMAQC

>PintOR41

MWWIFMFRVSGVYVMLISTLVCLGHQYKNLQSYFYNLNNIFEIAELTLRQKEEKYENGLKLGINMHARTLWCTRECEASFMWVLSGQILVNVTVIILLMAQMLSSERTFGSVSAIVSTMTAMLISTGFFMWNAGDVTVEAAALSTAMFNSGWQNCQGDSTVRVRKLLVIAMTQAQVPVSIRALGIVALSYQSYVSIVKSSYSVFSVLY

>PintOR43

MDDENNSHTQPIELQTLDESLGNISLPCRLVGLNIEARERKLKDNLVYWFNMIWMNLALVACFYWTYKRIVEGEDITEITYVAPGAILSIIGDFKGIFSLAYENETRLLLANLRRLEKNINVNKLTEMELDIAKSSLIFLNRVVKIMNVLNIMMLLMYDLSPIVLMA

>CsupOR1

MRFRIFQRKLSLEENKSTLQSEPITNYLHFLEIPLKIVGCWDWYMKPETECQIILNNIYYGMVLFFLINVPATLCVHLSTEWKDVMTTLDEIADCLPYFVSIVIVIYFGVYRQEMYDLIQSMGEQFKYRSANGLTNMTMLNSYITAKKFALFYTICTLFSVSMYVVPELISWWTNKPLQSFMYMDITKSPFFEITFLTLYLSQAFVGLAMGQFGVFFAANSILLCGQLDLLCCSLRNTRY

TALLQSGVHYRSLRLSHSDIKSDELHNYIYNVAEMEESSYHYDDKMEAITRPRKTKFDIYSSEYDEATERAVRDCARMCRVVNSYRERFERFVSPLLAMRVVQVTMYLCMLLYSATLKFDMVTVEYLGAVALDIFVYCFYGNQIIIQADRVTTAAYQSAWATMGVRGRRLLLNVLLANRRAVAVRAGYFLPLNLHTFLVIIKTSFSYYTLLVNVNEK

>CsupOR2

MASSTRPRHYFYLHFLLFRVLGLGWWHQPDERDTRNFFGWYLYYSIVTQFVWVVGFVGLETIDPFIGEKDIDRFMFSLSFVITHDLTLIKLYIFYFKNDQIQDIVRTLEIDLYNFYQNNAKNRATIKISRLMTASFVFFGWITIGNTNVYGTIYDIRWKAEVAKLNNTDLAPPRTLPQPIYIPWNYQTEEAYISTFVLETVGLLWTGHIVMGIDTFIGSLILHMSNQFSILREALITAYDRTMIRLYEGVRQDFIAITNSDIDKKEQYTQDNIEEVVKSRYSKEEIEVALTETFKNCFRQHQALIGCVENFSTTYAYGFMTQLLSSMAAICVVMVQVSQDASSLKSTNLVTSLAFFVAMIIQLALQCFTGNELTLQASRIADAVMHSKWEKMSPKLRRLLMITMMRAQRPLRLTAAGFAYINTDCFISIMKAAYSYYAVLSQKQST

>CsupOR3

MCFNIASVRHTTDNSTQISFEKKSLGALFTIVNLKVKDIRKMARSQRLSIIASIRHILSTAGIKFTDTMHVHWMAKVAMICLIFTYVLQASALIQIRHNWEFFFECSGDLFYRGMSLVKFYIFRRNYETWCSLIEQADKIEEDELSNERDRENGNFLFSEHIQAYSVRYEKIKKIITTIFRTCTVMYVSSAFIEYGIKKQTVDGSVDLPHILEIWSPLDVSIVGYIITVSFELISAVYDTSTQIAFDLTSIGAMIFISGQFSLIRHYSEAIGCKEQIYPSKEQDDLAHKQIIICHQIHIQIKHLTEMLKGLLTNILWLYFIMSTVMLCSIVVRLNLETSLVQLMTMFLYMCGITTQLFLFCYFCDDIQNKSAIGMGEGPYGAAYWSLSPRIRKELMILARGMSIPCQLYAGPFIPVTLPSFVQILRTAYSYYAILGNRG

>CsupOR4

MKTVLTPSQFAIFVQFVMLERVRKFGLRYCDLPTMLSNVSCMLRVLTLNVDSRHQKGIPIIFYVLTATAAASYFYIYLVSMVWFVFWRCSDTGDTIPAMIVFSLGIASEIGPCKLIFMFLYRDVIMKIVDGYLSCDATVIKGKRFSENLLKTLRVVKKRALIYWLVIIGNGITYVMKPIILPGRHIMEDQFILLGLEPMFETPNYEIAFTLNTMGVYFTCYLPANITAFFIVIIGYTEGTMLALSKEIRHLWDDAQQFYQETFNNAEVAINGGTIDPTFKKRVINQFIKERLQKIVVIHTTILNLIHQVEYVFRITIAVEFVLLSAGLIVELLGGLENTYIQMPFTLIQVAMDCFIGQRLMDASVVFEEAVYDCKWENFDTKNMKTVLLILQNSQKTMALSAGGVTTLSFSCLMTVIRSIYSAYTALRSTMT

>CsupOR5

MKILKRNIKEKLAILKPILPYGVLESWDDLDPKLYHAVHIYWLKFYGLWYYDFAPGSFMFWIRFLYTMLVMWLVCFLPGIGEIVYLLKRRDDIGDIAEGLYLFLSEMYTYFKMSVFWLNKDKVLNLLQYLTCEHFKPIEAEHREIIKKSIGTARFLMTFYSTICVGAVSVGIIMPLTENFDILPTNVEYPFFDVYQSPAYGILYFHHAYYKPATCIIDGVMDTILAAFIVSALGQIEILTFNLRNFDVIAKRRHKRALDGNKPEASWSNERHIRAVLKDCIIHHKSIIRYVSMIESAFSLASALQFMLSVMVLCLVGLQFLSIENPASHAMQIIWMAIYLTCMLIEVFIICWFGDELIWKSRGLVQAAFDGPWLKIEQKDKIFIVIFLERCKRPLRVTAGKIFTLSLDTYTILINWAYKA

FAVFSKVKK

>CsupOR6

MWKTIRKFGLEYCDLPTMIWNVSSMLRVLNLNIDPGNTKPIPTTYYIITAIVTASYFYVYLVSMVWFVFWRCRETGDLTAGMIVLSLGITSEIGTTKLMFMMIFRNKLREIVELYLECDSHVNPDSRFLHNMMKTLRHVKKRAMIFWLVIIGNGVVYIVKPAIQPGKHLMEDVFTLYGLEPSTEWPNYEITFVLMALGVVQTVYLPANITAFIIIIIGYSEAQMLALSEEVLNIWNDGLHHLNDHVIIDACADSNDQLTSLEEIISANRNNYDRINEFIKIRLREIIKVHMTNINLVQQMEQVLRGAIAVEFGLLIIGLIVELLGGLENTYMEVPFALMQVAMDCLTGQRLMDASIIFEKSVYACKWENFNVENMRTVLLMSLISQKTMKLSAGGVTMLSFSSLMMVIRSIYSAYTALRPTMS

>CsupOR7

MFWNKIKAVYNKEDFDYSKRFVDPKIYHRIFFAVQRAYQVIDEPFATWTYITKTITALCGVGVLTDACLSLYHAIDIFDMSLITESGTYVLMLLYKMMTLITTKVNLSDYIHLIHAMKEDFNYIETKKEKYRKVFFETQLGTWKACFVVISFMFSMGTSLVLFASGTLVVYHLTHTPGDGSHRTLVFPFWAPGVDYTTTPAFEIAFTFANIGVMACCYNYAFVIQTNIVWIRQIAAKADLIGMCINDLLEGIYSTDDEEQRQHFASLINFRMKEIVSQHIIMYRLLECYAAVYRKCLMFEQFIASPLVCMLAYCSAEKIDNGEVHVVMMVLCLSAILVLFIPCYLCTYLRTKLSGIHDACWNIRFWDAGPNIRPYLVLIMQRCLRPLPLQMPGFQEVSIKTFSSKMTSAYSLFNMLRQAD

LDF

>CsupOR8

MERMIKNLCDILISVSYREVTTMLRPFFKRLENNNHPLLGPTLWGLARWGMWQPRLGINTKIYCILHIVATLFVISQYVELWIIRYDFNLALRNLSVTMLSTVCVVKAGTFVIWHDQWQEIIEYVSKCENRQLSKRDKITSEIINNYTVYSRRVTYFYWALVAATVFTVTLAPLAAFWSSKEYRARIRAEQIPYPEIMSSWLPIDRTRGIGYWLSIVEHTLICFYGGGIVATYDSNAVALMSFLAGQLKLLNTNCSRLFEENYESRNNTVAKIREYHHDHLRLIKYSKILNGVLSPVMFLYVIICSLMICASAIQIATNGTTSMQRIWIAEYLMALIVQLFLYCWHSNEVLIMSHKVDDGVYASSWWSQSQSVRRSVLLLGGQLRRPIVFTAGPFTKLNLPTFLAILKGSYSFYTLLINKED

>CsupOR9

KRRRQWLSALIQFQLNQQKSTMLLDRLISFAKRWEDPESPLLGPNLKALHLFGLWKTDIRVRSTLLIIVVVFVITQIIDLYLSREDINKALCNFSLTTLSVICIAKSYSLIVHPVLWKKLVENISQEEATQIKKQNPETLSVIGNYTRYSRFISYTYWIMVAMTNFALIVAPLIRYLTVSKYREDIANGIERYPHIINSWFPFDDNAMPGYVYASAIQIIMSIVGSGSLAAYDTTAFAIMIFMKGQLIILKNNCKELFRWETKENNIEFFAKIKECHRHHDFLKRQFNQFNDLMSPTMCLYVLLCSITLCCSVVQLISKEATASQRLWIVQYSSGQILQLFLFCWHANELFLESQNIDGGVYASDWWKADVRMRKQILLLAGKVNYPMLLRAGPLSVLSLSTFFNIIKRSYSFFTLISRMQE

>CsupOR10

HPSAKSPIQRKSFDIKGVLLERVLVSSISMDLPTYDEIFKGIKNIFWLTGIPLDAPHKKLRFYVACLSLIITLYGEIAFFTSKISSENILELMDLAPCFCIGALSFFKGIFLAWKLNKIIVLKNSLEILYDTIFKSDSKRKLLHREIMKVHKLVKYYFGVNTALITVYNFSAPIFMTYHYLSQGKVKFMVPYAVIYPFAIDNWPAWIVAYTEQVFSGFVCILFITMSDALFCVLTSQICNNFYVISDEIKRLKNGNHIGLGEIVKQHQYILKLSEDLEDIFRLTNLFSYLVGSLEICALGFSITIGDWSHFLGYILFLVSVLLQILMMSVFGENIIRESGRVGEAAFLCEWHEINEKAKRTILIIMIRSHKHQKLTAYKFSVISYGSFTKIISTSWSYFTILKTVYKPSEVNNI

>CsupOR11

MNVISINPAEYRNTLILSLNYLKICGITLDRNDSFWEKYCHLCVVSIIMMLHFVSASLYIVQELVQNILQEANFISLWLITVQVFLRGIIILTNKTSIRGIIEQIGCNWRSSDLNEEQIRIKKDFLNRLLYTQKVIKIIGWCAGSLFLLPPLLETVFRSFVLHQDSAFVLPFPCYYQFTVTGWFTYFIAYFIQIYCSSKLIFMYLGADLLLIVLCAHLSNEFELLQVDLGATIKPTKNENEILEDEITAFGREERSIGDFVRRHQKVILLVQLLNISFNKMIFINLLFAAIAIVFFALGGRASRDPTNVANNYMAILVILINMFVLCYSSEMLCTSSSGIADYAYNNIWYEADMRYRTDIYFIIMRSQKACSLSSLNYLPISLSTFGKVLSTTWSYLSLANTFFEN

>CsupOR12

MKILTTQNENSEKEIRIKPFHETYKKITYGLTLGFMFPNPRTAKIRIVTIVIMLVLFQPIVVTVLIDMYSCWQKSDMFNIIRHSTILGPFLGAFYKMFLMHYKRAEVKRIIDEINDDYLTYNNYNHELKQIALESIKSSVFFVEQLWTYTVTACIMAFPVMGIVLTFISHLTQSEPKKYMVHDLKIPFRPPEDRFETPFFEIMFVYMFLAAIICVLNYVSYDGLFGLACYHACLKMRMFSKKLEYVFQCKDGDAYSRLVQVIEEQKATYEYNALVQNSFDIWLGTIVISTMIQLGSLLFHISAGYGFDFRYMLFSCTSVVHIFLPCTYASKLRNTSVETSTLMYCSGWERSRERRIVRVMPFLLARAQIASRITAFYLFDVDMQLFVTMMRTSYTIFTLLRT

>CsupOR13

MKKNMKEFEYDFEKAFRITTKALHLNRAHPFIERNLFWCFQFLLILTLSVMTFVFTFNSLLFYDIPAGEIAEASKNGTMAIVSLTITFKYTFLLYNQNYIKRYIAIINKDYELSKGFVAEERAIVIDYSRKGAKVSLYWLVATTATSILFPVKALVQMVYYHWEDEFRFVPMFDMRYPTTIEIMKNVPAMFCLLFLLCLMFDVYATTMYVGFDPLVPIFLTHICGQLDILSQRIMDIFSDESNLNSQEVNYKLKCINVTLQDQYNMIKEIKSKFTFLYEFTMKTTTILLPLSMFQIVEDLQRRKLNLEFISFFFATILHFYMPCYYSDMLMDRSQKLRDAVYACGWEKRHNARARKTILLMLTRTTVPLALSTVFYPICLDTFAEMCRQSYAIFNIMNAAEV

>CsupOR14

LHSCVLFRVTKMDILTSIGGVFRRIVTRFREDSFDSLLGIVDTVPSLAGFSLRKDKIFVPFFIFHLSLLTYIYGVGSFEYQAKTAKSAGDFIKSFVNVALLVLIANNSHFFMMKRSLLRSTLTEMQNSDKLARCNPASRLKHKKLCNRIKYIILIFYFVNLTNASCVYLPSRSNVDVNIYGVTPCYGMGSLTSPKREICKAMLFGQEVTVMIVVLNFQALLIFVIGHTSLLYQILSDEIMALNDYDKSMFFNNPVVKDILPVLIRRHAMILSIINKCKVLYSVPIGVNFGSNAVCMSLFFYLPLREWIDFFPILMYCFIVFFLYCFLCQRLTNAAQLFETSVYACGWENFETNEKKAVYFMLRQAQKPVELLAADIIPVNISTFATTLQAMFKFVTVVKV

>CsupOR15

MFYLLKKLEDKNRPLIGPNVKALKFWGILLPKNLYTRYLCILMYLLVVIFVGTEYVDIWFVKADLNLLLNNMKITMLATMSVVKVSTFYRWQQHWLDILNYVTRADLTQRKTNDVNKIEMINKFTTYSRKITYAYWSLVYTTVIFVVGYPIFKYVFFSSYRQNVLNGSEPFFEIVSSWVPFDKSTIWGYILASIYQAYSSIVGGGWITSFDSNAMVIMVFFRAELELLRIDCANIFGTEKAQVSDEVAMVRLKDCQRRHAEVMKYIHLFDECLSPVMLCYTIICSVMLCVTAYQITTEPSFVQRLVFTEYLVFCVTQLFIYCWHSNDVLYASRDLSLAPYESIWWSRGVEHRKNLFILTAQFSKVVEFSVGPFTKLTVATFIQILKGAYSYYTLLSKSDE

>CsupOR16

MKDIFILKTYCQYIYRVGSGNFWYEERIVGNDRSLSYKIYRGLHFFLYGCLTILEIMAAIFGVFPSDEKRDAVTFAVSHTIVMIKLFSVISNKALIKQLNKNMTELCEEHEEQQLMAEKYKIVKINVAVYFIIVYVTAVFFAFEGLRKLFNGVHFVTVVTYYPAYEDNSALANSFRIFTTIILQVMLMSMIVTVDTFTMTYLIIFKYKFITLRHYFDSLRENYLKMSKNGNQEIAAEQLTNGFVKGIIMHQKLLKTAKNIDTAFGLVIALQLCQSSGSAVSLMLQLALTDQLTFLASIKAILFVLALFFLLGMFLCNAGEITYQASLLADAIFYIGWHEFAPQPPPKRSLRRLVLLAIAQAQQPLIMKSFKMIELTYGTFLQVVRGTYSVFALFYAQ

>CsupOR17

MSEEAKREIAESLSLNTFCMERIGISFESPKSNIANVRQKLMFVLSVWGICYHVFSEIAYICLTLTKSPRVEDVVPLFHTFGYGALSITKLFVLWYKKNVFKQLIFELAGIWPLPPLDDDGQSTKNKSLAALRMTHRWYFAVNVLGVWFYNLTPIGIYFYRKWQGLDVEMGYVWVSWYPFDKHMPYAHFAVYIFEMFAGQVSVFIMVSTDLLFSSMASHISLLLRLLHRRLEALATTNKTEHEQFDEISANIKLHQRLIRYCNDLESAFSLSNLVNVVLSSINICCVVFVIVLLEPFLNVSNKLFLGSALIQIGMLCWYADDIFQANLKVSAAAYNSGWYHTSPRCRRAILFVIQRAQKPIAFTAMGFTNITLVTYSAILTRSYSYFALLYTMYNKG

>CsupOR18

MLKRFFNSLEDPDRPLLGPNYWILKKLGLLLHFGKLGNIFTILIHNMGLLFVSTQYVDLYLIRSDLDLVLQNLKISMLSVVCVLKVNTFLLWCSKWKEVINYVTEADKYERNTDNPDNVQIVKKYTKYCRRITYNYWILVFTTAFITVVTPLLQYAYSSTFRESVKNGTEAFPHIFSSWVPINKNDFPFNWVTVAWHTYICVQGALVVMAYDTNVMVTMVFIGGKLDLLRERCKLMVGVNGVTTSNVDLAEKLRELHKTHVLILRYSRLFNSLLSPVMFFYMVMCTLMLCASAYQLTSATDTTQKLVMAEYLTFGIAQLFIFCWLSDDVLTKSEKVMLGPYESQWWLANVKQRKIILLMAGQLRIVPVFTAGPFTKLTLSTFLNILKGSYSYYTLLR

>CsupOR19

MPPAERHFLGSIILQWMSNRGFLMNNNCYTYWLNGVTMICCHVTFVLQCVAVIDARDDPERLMQCFCDASFGGMCVLKEFSLRKHRHCWISLLSKISHLEEEELEFETKSINQDEDAHNIVFSGHIKEINKKVKKLNNILSRLFSVTAIGYMLTPFIEYGIRKLIGAETAGLPHITQYWSPLKTNLLGYILAITLEILFVINNYAVHTTFDFSVFGIMIFISGQFRLLHDYSEGDCGSTLCISETREDLAHAKIKKCHEIHVKLIRITNKLSKLIKNILGVYFTLSTISLCAIAIRLSTETNIIKVMVLALYTLSAFLQLFLFCYFGDNLQNMSSIGMGKGPYAAAYWALSPRVRKELILLAKGMSRPCHLYAGPFTRLNLPSLLQVKKSYISSR

>CsupOR20

MSLASSSVSPHLTLLRRVGYCRLSPGVTGSSSTRYLHEIYRKFALAAILTYTSEQAIYAYQNRRDIEKLAPVLFLFLCHITCIVKKLVFHLNAPRIDQLIAELEDAAYNSQTPPHRAMLRSTASSALRLLRAYVGCAIFTCILWVMFPLVDRLQQQDIEFHFWIPVDYNRPLTFPLVLIYSYYVTTLVAVGNTTMDAFIATMLSQCKTQLSILRMNFEDLPLRAMEMAYNSTSYEAALSKLFLDCIHHYQKISETMNELLRVFGLAVLVQFAVGGWILCMAAYKLVSVNILSFDFVSTTLFLVCILTELFLYCYYGNEVYVESDLMVQSLYSMEWVHTPLAFKRSLLLTMERAKRPLRPAAGHLIPLSLDTFVTILKSSYSFYAVLRQTK

>CsupOR21

MFIKNPNKSVGCSLSAMMMFGFWLPKNLTEIERILYQCYGCFWFILILGCYAATQIVQLYFDLGILHLMISASFLLLTISTEIIKLLNIVYRRRMIKSMIDDFDQVLRSTDTEEARAIVKRCDRETTILLAIYAIVTIITMVGFAAAADKGMLPIRAWFPYDVTKHPNYEITYTYQILALSVDAFLNVSTDTLVSSLMAQVRCRFQLLGLSLRNLCQGIRINEPLLASDQVVIVKDRLRLCVEQHCATLEAAQKLQDYFSFPTFMQLSVSLLIICVTAYQMNAVIGKPMAFIGVAAYLLDMMLQVFLYCYQGSMLSEESIAIADAAYECPWYVMPVPLRRSLLIVMTRTRRVAKFTAEGLTTLSLSCFMGVIKTSYSMFSILQGME

>CsupOR22

MLYLIKKFLVPAICLGLTFMATGMEMMFVLHGIQIKDYSFATECFCYCVMLGIIPVFYATNLHKKKYLLQILEDMAEDFVFICKLDTKLRNHFIKGQLLIWKLYLSWIVFICVMGILYVGMTLLPLTYQSLFATLDEHMVRPLIFPIWLPKDDPHRTPNYEIFLFLQLALVLLYMKAFGFYVYIQFHVLLHNKVLLELIIMDFDTLFDGLDEFVAMLPNNDMRRIAVQHTLNKRLERIVTWHNSVFKSIAALSSVHGAPLMYQVGFSPVAVCLMLYQIADKLDHGSFDIIFTALFFAACIQLWIPCYLGTLLRNKAFDVGDACWNCGWHETSLGRLVRMDIIIIILRSQKPLSIKFIGLPNLSLETFSSVGITNTRLHIIL

>CsupOR23

MLDENGKKCFENSLRRTKLFLSLSGIRISATKWPKALEKLFDTYFYYFQVFWLYADVLGEISWLIEGVLNGSSFLELSLAVPCITVSCLATSKSIFLYLNRDVVVKVIDKLREIYPESDETLKYHSNADLSHDKELDIFEDDSNKSDINTDIERDIKNESVDFLNLVVKVQYYICSAVVVAFPLMPVSTMVFIYYSTGVLEYKYVYMVKYFFDPFKMALWPFVYFHQVMSTVIVAMNVFGSDTLFYAACIYIQMHFRILCHHYENAVSASSIQTRLNLKVAIRRHHELIDLVNRVEILYTKSTLFNIVTSSFLICLSGFIITMVEDIIVMVTFATFLFMNLSQISLLCYFGDMLMSSSTQIVNAIYNSLWYDADERVKK

>CsupOR24

MVHIYFQHPRLALLLTGLWTPPKEKKFRLLYIAYRICVISMQYAFVTCNLVNMVMLWGDLEQISDACYLFFSVATCSLKNTNYLLSQKKFLSLLDFMENEVFVSQSLVQDKIISAYAKKMGRIYLIYIVCGFCNCVEWALVPVFEKEGHKIFPFKIWMPMDAASAEIPEYLLGYVLQLFGIIFSVSTYLTMDIVAISLLMFVPVQLDIITCKIKEVQHVSILFDPKQRRDLIEHNSALLKDCIRRHQALLRYIEGVADIFDIHIFFQLSATVIVVCIIALQMTIEPPNTFHYYSTVNYLMAMLVQLCFYSWSGNEITERNNVLRDGLYECLWYEQDLSFKRTLWIAMEFMSRPLIFKAGNYIPLSRPTFVSVGLQTWL

>CsupOR25

ETGGTWYTRADYLDFNLKCLFYAGLWPNEKWSRNKQKIYKIYEVTLFIMSFTFMFITSIGIYMAKSGDTIFFFADVDKNIVTYNYIFKIIAFFTKRNEVKTLINYIIYSGDRITDQRKKLMVTHVIVVTGMILALTGVFQILALMKGELIIVAWFPFDPMKNQWSLFLAEQLLVILFAVPCVFRAISIQGIVCSIIMYICDQLTELQSRLKNLNYSVETAAETKEELKLIIKKHIRLMGYAQSLSYAFKEYFLIQNLAVTAEFGLNALMVSIVGADQKKHLLSFIAFLMLALVNAYIFCFLGNKLMYESTCIALAAYESSWISWPVSMQKDVLLIITVAQRSFKLTAGGMAYMSMQTFAQALYNGYSMFAVVRDLVN

>CsupOR26

YLRVCGFYRLDQSSSKSVKILHRIYRRLVLSFFILYTIQQLLKIYDARSNVDKVMGTMFLFLTNTDCIYKAVILWKKADHIEGILEVMKGPIFNKGEPGHRLFLQDTIRKTLLVFRIYNYMSLFTCFLWVLHPTVLHMQGKLIDLPIRLPFDPNTKYYTAALYVWIQTSWLAYCNTAADVFISILLEQCRTQVTILRYDLENVVQKSKEEATETHGNYGDILERKFREMLLHHKEIVKTAGEILDIFSGAVFYQFLVSGWILCTSAYKMVNMNPASIEYASMISYIICVSIQLYVYCYYGNEINYESRRLTDSAYAVDWLEIPVRQRKTLIIFMERIKQPIEPMAGTIIPLSNSTYVSILRSSYSFYAFLKNSSN

>CsupOR27

RDEESNKIYQCYRVVLLTLFKFVFFISFTLDLFFTPINVGLIVSQSLLYFSELAGLFKIFMVLFKRNSILEVFEILDSKEFLAEDEASQQIIQKSHIFFRKYRTACTLFYCTGVIIFFVPVIKYWTTTGAQLKLPSFQYYFLNDNVREKYSMYLFLYQHCCLVLVVLSNSASDTLICGLIMMATTQFEVLNWKMSHLALRPSEKHYDPKEEEIIIKDRLNKCLRHYDVILRYCKEVQETTGLSLFAQYTTGAITICISLSSFLIPMTHEDFVILVCYIAGMTVEIFYPAYLGAELTEKSENLIFSVYCGDWISRPESYKRSLRLMLERANKPVVITCLKMIELSLITFTSVMKSAYSFFTLLKCLLERQQ

>CsupOR28

VWIPPTNKSLLHKLYRSLMITLQYLFLIFQIIFIIQVWGDLETVSQAFYLLFTQACLCLKVSVFHVNVDKLRELLKQMNGEIFQPQSDRQKQILSKQASRIKALLLAFMVSSQFTCSLWAMKPLFDDVGSRKFPFDMWMPVSPEASPHYEIGYAIQVLTIGMSAYMYFGVDSVALSMLIFACAQCEIIMDKIMSVTSINYAMKNKERQKIFAKNRKKLIDCVKHHEALYAFTKLSEDAYHSYLFFQISGNVGIFCMTALRLTVVEWKSVQFFSMATYLYVMMGELFVCSWSGHELTSTSEMLHTAMYDCPWYEQDVRFKRDLCFAMMRMSRPLVFRTGHYVSLSRQTFIAILR

>CsupOR29

RGSCIWSSGVPMAITFVLCLIYRFELRGFLEEMAFKDEMQAMPLIQHVNSLTEGNLLYELKELVRMSQMKLANFSRIFLKVYIMSVLVIATLYPWSSIYEMCVTEDDTLRLIGFDMWFPWSLDDISVYVMSFLFNVYLGCLCSIAYPGLQTTIVLFLGQLIRQLRILNFILSNLSDLADEIVGDQNHNDIWQEVCNSLLCQCVDHYVKLKSFSNRINLTFHFYYLALLLMATVLVCMCSVKIAISDKLALDTMKYYMHGFCFIMMVLLLCTLGQQVDNECEKLEESVTNKWYLYNKNLKVNIQIFKMALDQRMPISIFGSATLSFPTFTWFIKTGTSFFTLVMSVLDN

>CsupOR30

FIKGIQDRHSTDELVNTLFLFLTTVTAIVKQVAFTVRMKRIKQLFDTTDGELFSPKNSAHLELVEQNEKYMRRLHFLYICTVLSTCAYMSLYPLGNKAFGQDHQDINYRIYFPFDPRKSPIIEVFACSYFGIALTLQGYVNSIVDCTIVAFYGQCELQLKLLRYNLEHLTDLDDVDLQEGTINENTLSYIDDNLIKKRLVHCVKHHQKIIWFLSETQSISNEFVTLQLSVACWTICMSVYKLVTVDMFSGEFFLTIGYLNCMLMQFFMYCYHGSQVLVESEFIAESAYCSNWVDISPRSRRLLLIFMMCCTRPLVICAAKIVPINLESYLAVLKASYTLFTILHKK

>CsupOR31

LHKMDTSFEVDLHRHTSLHIAMMKFFQIWLYIPHPRTSYKYWLSVTLRFFVGLFIFVIPTTAQFMYLFSIIRSDDFEIQEVASIINLVITEMLTSLKLLALHLRREDFLDLMKQLDGKQFVCHTKNHKKIVERSIHFSRGIYIVLSICTFIDVLVHMVVVPAVHRFQELPLKMDLIFFDVNDPSYFPYVYAFQISYKPAMVTTFVTLNTLCWACMCCAISQLDILINKLENMKKLIKDTKVDWHYDENEAFDKIFGGIVQHHLAIIRFTKVLQSVLGGQLTLSLFMTAIIVCTTAIEILSIESPRKHITELLWMLVFVSIIVGNLFADCYFGNAITDKSV

>CsupOR32

MFVEKPGMIKNGIRYKPECSVSGALGLVLRLSQLVGIGPLSFRKRNRGWFVSLSPSLCLVSYVAATVLNTAALTGILLDLQARPSKSARVSSPTLKFVWVSDYIVVLVIASVAAYGAPRRLTTTILCLARIQKINTGVSSKSSNDWKTSLLLMAFLLYVACVLTADYCIFLRAVFLSDRAFTAACLYSFYYFAYFLLVLLEMQYVFSALEVSKTMGRLNKLIGEVEHMLTMHYASLKKIESNDPIKLPLKYDNLMIDSMDTFKFDSGMTVGLASKSISETIRRLALTYMEVCEVVRQLDSSHGVGVLLLLLSFLLHLVITPYHLIVKITCLY

>CsupOR33

VSCPHTTVELGTREQQQKDLTNTTFFMPMKQEMSLAGRSVAPHLLLLRWCGFCRLRPRHASAGHPSLLQRAGRRLHAAYCTFALAATSIYLMQECVYAYQEHNDMDKLARVMFLLLCHITSIIKQVVFYTDADRIDDMIAALDDRLFNPKESSAQALLQGTARSAKRLVRWYSSTAVATCVMWIIFPIMYYVSGHQVEFAFWITVDHSGPLMFTVLIIYSFYVTTLVGIANTTMDAFMATILYQCKTQLRILRLNFENLVETANKIVAANPQESYENVLMKLFLEYLEHYQQISETNNCLQDIFGTSILVQFGIGGWILCMAA

>CsupOR34

IYRKIINYISHVEQKQIADGDLEINQIVSEYISRSRRFCYLFWALPVFVDPIMFLQVFITSIRFGKSTGTYPKILDIYIPYSDYPPGYYFSLLIQTTIGFTMSAYIVSWDSLVCVTMIFFAAQLKITRLMCSRMIDPQNPQKSHDNIVECLKFYTTLIEKQRIFNKLISPVMFVNLFVTSINFGISIIEIARVEDDFATLASGCTYLGACLIQLLIFYWYSNEVTVESAKVSYGVFASDWPLISNKYQREVALLGVATAKTLVFEAGPFNEMTLSTFLGIIRASYSFYTLLNKTN

>CsupOR35

SRFVTYNSLPLIIVVDSFPRIIMYYEHEILGEELVYLYPFDGWYPFDKINWYYTIYIWESFMTCVVIFIYGFCNIIHAAYTALICMELEILGNHLENLITADDVKNISRGRNAQKTHENIKSKLKNIMDRHQFLARIAKELDNALGDIMLLYYIFGAIIICLAMFTAIAVDDLYKTVRYFFMCCYLLWEVFFQCVNGQILSDHSLELSTAIYTADWIYADKDTKTMLHMLMARAQKPFLYTAKGYTTMNLNSFSGICKISYNLFNLLRTAYS

>CsupOR36

GHRRVTPIVTVRFCSKMDIPAFEELVKQIKINFWLIGIPFNDVKLHFRFYLLLFSLMIMVAEECGFLFVEYSPENLLEITELTPCTCIGILSALKIISITPYRHKIFKLTESLNELYSETLENQAAKKLITKKIILMKNLVTYYFVLNVVLVSVYNFSSVVIMSYTYIKTGKTVFYLSYAILVPFSIDTWPTWSLAFIHAISSGYICVLLFTTIDALYYVLSLHICNNFSLLTEDIRCLNETNSQNIRDIVKKHQHLLKLSEDL

>CsupOR37

GVLYKSSGKLVMQIPFVAWYPFDETDIRYWPIAYFHQLWAGFFDASSVHGSDSFYSLSCVFLQIQFKTLQYDIEQIIPEETNINTPELYKSFRKRFMLIVMRHQELIRCVNVLEVIYSKSNLCIIAVSSIVICISAFNFTTSDDIIWRTIFLGFFIMWLLQVFSLCYYSNLISLSSTEVSNAIYNSYWYKANAEVMKDLLFVLRRGQKPCKLTAWGYSDLNLAVFSKIVSTSWSYFALLQTMYSE

>CsupOR38

HSRHVTYFFWALAFFSNFSIFSEPYQKNINSDNGDPVFKKIFDGYIPYSDYPPGYYISMFIQTVLGNIVSAYVVGWDTLICTIMIFFAGQLKVARLLCSRVINVQNPELCRKYIADCHRFHTTLVKNQKLFENLISPAMFVYLIVISVNLGVCIIEIAKIKNDTPTLISSCLFLLDCFIQLLLFYWHSNEVTEDSVLVSYGVFESDWYQAENKYQREVA

>CsupOR39

MSEETKMYEKLLKRVNTLLIMAGCHFDEADAKRTVIQRFSSRRYFCFNMVMLTIHLICDFTWLALEYKRASLIELTYFIPCVTLSLLACTKSYLLVKNGEHVIDLIKSTKILQAISGRFELRTKTERESKKSVTILTAFMNFNLVLYILGYILFAIGPLILT

>CsupOR40

MSEETELYETLLKKVDTFLIVTGCYFDEADEKRNVVQRFLSRRYYCFNILMLTSHLICDFAWLALEHKTASLIELTYFIPCVTLSMLACAKSYLLVKNGNHVTDLIKSTKKLKAISGRFE

>CsupOrco

MMAKVKAQGLVSDLMPNIKLMQAAGHFLFNYHSDNSGMSTLLRKIYSSVHAILIVINFLCMAVNMAQYSDEVNELTANTITVLFFTHTVIKLLFFAVNSKSFYRTLAVWNQSNSHPLFTESDARYHQLALTKMRRLLYFICTVTVLAVVSWVTITFFGESVRLIANKETNETLTEPAPRLPLKAWYPFDAMSGTMYIIAFAYQVYWLLFSMAIANLMDVMFCSWLIFACEQLQHLKAIMK

PLMELSASLDTYRPNTAELFRASSTEKSEKVPDPVDLDIRGIYSTQQDFGMTLRGGGGRLQTFGQQNTNNPNGLSQKQEMLARSAIKYWVERHKHVVRLVASIGDTYGTALLFHMLVSTITLTLLAYQATKIGGINVYAFSTVGYLSYTLGQVFHFCIFGNRLIEESSSVMEAAYSCQWYDGSEEAKTFVQIVCQQCQKAMSISGAKFFTVSLDLFASVLGAVVTYFMVLVQLK

>CsupPR1

MDFELKENRFRTIEFLHNRIVRNFLMPLGGWPCEVFQEKTPLFSRFFRRFIPIQGSCMIYGELNYIIQNYSRLNFFLLGHIYVTMFLTGVMIIRAILPNKKEYNDLVNFFYGEFDLEHFKHKGSYYQKASEIVYKFSYYYSLVMAGMMIYGMLLYNALPLYHNYNAGVLHRSNRVANVTIEFSVYYSFPGFMPEDHFWFVTFTNLYLTYSCTVEICIFDLFMALFVFQMIGHIMILINNIKNIEMPKTCHNIEGFKTQTNVTVELYDYEENEILRNKIVEIVNHHRFILRFVSDVSFLLGPALASTYLCHLISCCLLLIECSQLDPDALAQYGPITVIMFYQLFQISVLFELLGAKSEKLIDAVYELPWECMDVRNQRLLCFLLQRVQTPVQVTALGLTKVGVTPMVAILKTTYSLFAFL

RSTV

>CsupPR2

MIYGELNYIIQNYSRLNFFLLGHIYITMFLTFVMLVRAVLPNQQLYKDMVEFFYGKFDLEHFKHKGPYYQKASEMVYKISYYYGLVMTGMMICGMFLYNALPLYHNYNAGVLHKRNRVENATLEFSVYFIFPGFMPENHFWSVTFVNLYFTYSCSVEICIIDLFMALFVFHMVGHIMILLNNIENVEMPKTHYNIEGLKAQTSVTVALYNDEENEIMRSKIIEFINHHRFIVSFADDVSSLFGPVLASTYMFHLISCCLLLLECSQLDPDALAQYGPLTVIIFNQLFQISILFEFLGAKSEKLIDAVYGLPWQCMDVRNQRSVCFLLHRVQSPVQVTALGMTNVGVTPMVGILKTTFSFFTFLRSIV

>CsupPR3

MLESTPLKNLETRKFYLLAVFLLSTFQMYETVDLFSRLYTILMINMMVGGMILFNLTPLYSNYKNGVFSKNPPENVTYAYSVTYSVPGFNFYEHFTLTTILNWIMSYDVSVNVCVKDLYLSFLVFQIIGHIQILKYNLEHFPKPKNQATNRFDAEENKQIRKTIAECVDHHRLIVSFADDVSDFYGPMLALNYMYHLISCCLLLLECSQKEPDALARYGPLTVIIFGQLISVSVVFEIVETKVKSTKYGANCLTLTLSMVPFSK

>HarmOrco

MMTKVKAQGLVSDLMPNIKLMQMAGHFLFNYHSENAGMSNLLRKIYASTHAILIFIHYACMGINMAKYSDEVNELTANTITVLFFAHTIIKLAFFALNSKSFYRTLAVWNQSNSHPLFTESDARYHQIALTKMRRLLYFICGMTVLSVISWVTLTFFGESVRMVTNKETNETLTEVVPRLPLKAWYPFNAMSGTMYIVAFAFQVYWLLFSMAIANLMDVMFCSWLIFACEQLQHLKAIMKPLMELSASLDTYRPNTAELFRASSTEKSEKIPDTVDMDIRGIYSTQQDFGMTLRGAGGRLQNFGQQNPNPNGLTPKQEMLARSAIKYWVERHKHVVRLVASIGDTYGTALLFHMLVSTITLTLLAYQATKINGINVYAFSTIGYLSYTLGQVFHFCIFGNRLIEESSSVMEAAYSCQWYDGSEEAKTFVQIVCQQCQKAMSISGAKFFTVSLDLFASVLGAVVTYFMVLVQLK

>BmorOR1

MLLSFKDDSRSPDIQKPQNFQYMKILRFNLKIICAWPEKQLNEIRSLGHSIHRVILPIQSVVCLACGILYIHFHFNEIPFFILASTFITVMMNLVTCSRTALVMLFERYLVLTGRFITVMHLFNFQKNSDYAYKLCTFVNRMSHFYTLYVLFSMFMGLGLFNLLPLYNNYVSGAFSDPYGPNVTFFHSVYFAFPFDYSHNFRGYIIMALFNSYVSVTCSIGLVMFDLLMCLMVMHVWGHLKILSHNLINFPRPKASHVITTPNGPTNVETYTEEESKEVFARLRECIKHYGTVDDFANDMSETFGVILLVYYGFHQVSLCMLLLECSDLSTKAMLRYGPLTLIMIQQLIQISIIFELLGSVADRIPDAVYQLPWECMDVKNRRVVYGFLRRTQNPVRFKAMGMLDVGVQTMASILKTSISYFVMLRTVAT

>BmorOrco

MMTKVKTQGLVTDLMPCIRLLQAAGHFLFNYHADTSGMNMLLRKIYSSAHAVLIVVHYICMGINMAQYKDEVNELTANTITVLFFAHSIIKLAFFAFNSKSFYRTLAVWNQSNSHPLFTESDARYHQISLSKMRRLLYFICGMTVFSVISWVTLTFFGESVRMIASKETNETLTEPAPRLPLKAWYPFKTMSGGGYVFAFIYQIYFLLFSMALANLLDVIFCSWLIFACEQLQHLKAIMKPLMELSAALDTYRPNTAELFRVSSTDKTEKVPDAVDMDIRGIYSTQQDFGMTLRGAGGKLQNFNAENNPNGLTAKQEMLARSAIKYWVERHKHVVRLVASIGDTYGTALLFHMLVSTITLTLLAYQATKINGINVYAFSTIGYLVYTLGQVFHFCIFGNRLIEESSSVMEAAYSCQWYDGSEEAKTFVQIVCQQCQKAMTISGAKFFNVSLDLFASVLGAVVTYFMVLIQLK

>BmorOR3

MIFVDDAVIGIKDPREYRHLRVLRTSLRLLGAWPGHYLGEETGSKYECAPMFLLMFIKIACLYLTIVYLRNNADVLGFFELGHVYLTIFMTFVTLSRGFSLTWNPNYHKVVKKFITEMHLLYFKDNSEYAMKTHRRVHKISHFYTVFLKVQMIAGLTLFNVIPMYNNYRQGNYASDRPANITYDLSIYYETFDILNTPNGYIFICVFNWFASYICCSFFCSFDLILSLMISTVSGHFRILIHNLLTFPLPEAITASKKFVDKHRCNGNRSEFVLEEAKLYSPAEMWQVTDRLRQCIDYHRKLVEFTGDISEAFGPMLFVYYLFHQVSGCLLLLECSQLNTAALVRYGVLTVVLYQQLIQLSVIVESVGTVTGRLKDAVYEVPWEYMDTSNRKTVAIFLMNVQEPLHVNALGLAKVGVQSMAAILKTSFSYFTFLRTVSE

>BmorOR4

MFKIIKNIIVENDALKQVEKPQEFQYMKWVQYHLKYIDGWPNMDMNKKNVSKIRFHKRHLLVVEQTITFLSQMFYIVKNYGKLSFFEIGHSYITALMTIVIFSRSVVTALGRYRKIARYFVSSLHLYHYKDISEYALQTHLLVHRLSHYYTVYLISLVVTGMLLFNITPLYNNISSGVFNSPRPENMTFQHAVYLGLPFDYTTDIKGYFVVFILNWHLSHIAASYFCTFDLFLSLLILHLWGHLRIILNNLKTFPKPYTNNSMYTEEENQVVLLKLQECIRYHNFIISFTVMMSNVYDVVIIVYYLFHQVTGCLLLLQCSTLDWESLSRYGPLTLIIFQQLIQVSMIFEILGFLSDKLPNAVYSIPWEAMNVTNRKLVQVLLQKSQKPIQFKAMNMMSVGVQTMASIIKTSISYFIMLRTIARD

>BmorOR5

MLLYYPNTQVKEKVNNVEEFTYIKFLKSFCKIMDFWPEREEKNSKTRIFRLRYILVLQFCFTLVAGVLYLTNSVGKQTFYDLGHTIITVLMNVVSLSRLILRCFKKYDVVGQQFINKIHLYHYRNDSEYAMKIHTVVHKISHNMTYIFSFCIIFGTVTFNLTPIFNNIGSDAYKNPRPDNVTLQQCVYYALPFDYTGNFKWYLLVAIFNVQKTFFCTSLFILFELSLSLMIICLWGHLRIFIHNLNHIPAPRNSFEYTKEERQEVDDTLKKCIQHHTLIIGFVRIMSETYGLAVLIYYAFQQVVGCLLLLQCSQMELKTVTRFGFLTLVLNQQLIQISVIFELLGYMSDKLQDAVYCVPWEYMDTSHRKMVYMMFRQSQIPLQLKAMNMLSIGVKTMVSILKTSVTYYLILKTVTTD

>BmorOR6

MKEEYYLQHPRTQLFYKVLAHVSTIESTIDLTWWGYTFPKYVGWFYHLQCNVVRLFGKCVVVSQILFIILNYQTIDKSVFIIAITITPLGALVGIKAESAKAECYVNLMKNFMDKVHIHSIYRKNENNEFVKKKVIQIERVSRFTAYFLVILIAINCLSWMLKPTLHNIKHFEEIMNKSMEFQYYIYFWTPLDYKYNLRDYIIIHTLCIYLGATAVTVIVTFDIFNFIAVFHVVAHIQILKNNVKSNWSDDFNESEKKGYLVSILEYHAYIIRIFGEVQSAFGLNVASNYLQNLIEDGLFLYQIMNGEKENVLMYGLMIILYLGGLIFLSIVLEEIRRQNYDLCEYVYALPWEGMSLENQKIFVVFLQRTQPDLEFETVCGMKAGVKPAFSIVKSMFSYYVMINSRF

>BmorOR7

MLLYHPNTQVEEKVNNVEEFTYMKFLKSFCKIMDFWPEREEKNSKTRIFRLRYILVLQFCFTLVAGVLYLKNNFGKKTFYDLGHTIITVVMNVVSVSRLILRCFKKYDVVGQQFINKIHLYHFRNDSEYSMKTYKAVHKISNNMTYIFSFSIFVCVVTFNLNPVFNNIGSGAYKNPRPDNVTLQQCVYYALPFDYTGDFKWYMLVAIFNVQKTFFCTSLFILFDLLLSMMIIHLWGHIRIFIHNLNHIPAPRNSLEYTREERQEVDNTLKKCIQHHTLIIGFVRIMSETYGLAVLIYYAFQQVVGCLLLLQCSRLDLKTITRFGFLTTMVNQQLIQISVIFELLGYMNDKLQEAVYCVPWEYMDTSHRKMVYMMFRQSQIPLQLKAMNMLSIGVKTMASILKTSVTYYLMLKTITANEA

>BmorOR8

MSLSTRCLLKDFCKYVYYAGAGNFWYEDIYKETVPYKMYVVISFFTYTVMIFLENLAALFGKLPEVEKNSAVMFAAIHNIVLTKMFLLLYHKRSISKLNCEMAAVGENLEEASIMRRQFRKMRLGTALYFISVYLSLVAYGVESARRTIVEGAPFYTVVTYLPDYDNTTVLASFLRIFFYITWLYMMLPMMSADCMPIAHLITMTYKFVTLCRHFDQIREKFQINVKIMAKTEATEILKLGFIEGIKMHQKLMYLADEIHRVFGIIMALQVCESSAVAVLLLLRLALSPHLDLTNAFMTYTFVCSLFLLLALNLWNAGELTYQASLLSNAMFYSGWYFCDFEKDWCRDIRRLVLIGCAQAQKPLILKAFGVLDLSYETFVSVARMTYSVFAVFYKRGD

>BmorOR9

MVARRPLQFHQGRNVDNVEDFKYVKWLRNHLKTVDAWPVHSKSKRKIQKRYVLPIFSAACFISQTVYLKNGIGTLSFVVLVHSYICFLINGSCLCRGILIATERYKRLATCYLKTVHLFHHKNRSEHAMKIHVIVHRLSHYYTIYLISLVFVGMVLFNFMPIYNNINSGAFKSPRPENVTFQHAMYLALPFDYTTNIKGYFVVFILNWYISLVTTSHFCTFDLFISLMIIHLWGHIKILMCSLEDIEGFVPGSSFKFTIEQNRKIYLILQECIRHHQFTIDFTNEMSSTFGLVILFYYFFYQVSGCLLLLACSQMDIESLSRFGPMTFILFQQLIQLSIVFELISSLSENLPNAVYNVPWEFMDKNNRKMIQVLLLQSQKLIQFKATSMMNVGVQAMATILKTSVSYFIMLRTMYQEH

>BmorOR10

MRTNAKSFLFVPSKVLTLCGVWPVEKTSIFSLIYRSIMLSSQFCFLVFNGIYIGLMWGDLKAVSDALYMFFTQTTCCSKAIGFYFNFMKIKRIVASMDDVLFTAMSIEDQATIFSHSRTVNKLYKGVLGFTGFTLVQWTVLSLIGSGRTLPFNEMWVPTDISKSPNYEITFVVELWMMVISAALFMSVDTITVATMMFSCAQLDIIMKKTQQIQEIPLSPDLSSRNRSELHEKNNGILIDCIKQHQAIVRFSELCEGTFQVHSFFHLGGIVFMICVIGFRMAGESPVSAQFWAALSYLVIILGQLYLYCWCANELTTKSEQLRDKLYLTPWYDQDVKFKRNLCIAMECMAKALTFRAGSYIPLSRAMFVSILRSSYSYFAFLNQANEQ

>BmorOR11

MDEHSHFETSLNKIKVLFKYSGMNLENTVTNTYEFLNHRWVYILNHAWTLAAVTFICIGISNGQNFIEMTCIAPCVAMTVLAVSKSFFHYINENAVKSLLENLIELERTDFERTKSVQRTEIVATEKQLLNMVINVLYVLNCSMILVFDMTPLIIIAIKYWTTNKFVRLLPYLDIFVFVPYKFEYWVMAYILQIWAECIVLLFIGAADCLFFTCCTYIRIHFRLLQYDFERLTSSRRESDGLRDDEDFRETYTNLVKRHQGLIESSSILEMIYSKSTLSNFVLSSLVICLSAFNVTVVNDVTIVMTYLIFLAMSLMQVYFLCFFDMLMSASEEVGNAVYNCSWYTEKASTGKDLLFTITRAQKPCELTAAHFAYVNLKAFMRVSFTSASITTLPTI

>BmorOR12

MTRITDVFSLNFIFWKFLGLWGKSAPSKYNMAYTVFYLFASLFVYDIFLTLNLIHTPRKLETLVRETMFYFNHLVAVTKILMMFIMRKKILVIFDLLDCEEFKPNDENSQEIMKRKTDFYYIYWRIVAVTSNLSCFMLVIGPLIKMLIWKIELGLPVCKFYFMSDELRNKYFVIWYIYQSFGIYNQMVNNLNLDTFNCGMLWMAVGQLQILKTKFVNLKLNDFENGLDLKSRDDMQIERLRKYLTHYEIILKYCAIVQDILNITIFVQLGMSSIVICVGLCGFVAMPSNTETAIFMFSYLTTMTMQIFVPSWMGTQISFECGELMSAAYSCEWIPRSKLFKRSLILFVERAKTPVRITGLKIFTLSLDTFTSIMKTTYSFFTLIRQLQVDEVN

>BmorOR13

MAPKQIDCFEINWKFWKFLGIWSENKPHRYYKYYSKIFITFFVILYDVLYTINFYFVPRQLDLIIGEMLFYLTELSVLSKVFTFIIMRHKLKIIFEILESDAFQTDTEEELKILHRAKVFIKRYWKIVALVSITANLTHISSPLLKNLIFKVELVLPVCSYSFLSESFLKTFEYPLYFYQIVGIHFHMLYNLNIDTYFLGLMILIIAQLDILNVKFRNLKSGKDHTQLNESIMGLNKNLDHYNEIERFCSLVQNIFSFTLFVQFSMASCIICVCLFSFTLSVPVEYYIFLATYMFIMIIQIMVPCWFGSRIMDKSILLSSAIYNCDWTSNSKDFKINMRLFVERANKPLSITGGKMFSLSLATFTSIMNSAYSFFTLLRYIQTRE

>BmorOR14

MSNYIFKPFHETYRIITFTMIAAMIYPNPATEKRRLIYIGLMLLSVIPLAFMIVTEMYEFFMASDLNNTIRHSTVIGPFIGGFVKVALMYYKRRQANELVSEINRDHLAYNGLKGEDREIAASSIRNCQIYCELGWTLIVMSCGLSFPVIAILLKIHSFTFKLDSTKHMIHDINNPFTDDPEDRFESPFFEIMFVYTFFSSFIYIINYVGYDGFFGLCINHACLKMKLYCRALEDAMRSDSRRHEKIVAVIEEQRRTYEYIALIQDTFNIWLGLIYVATMIQMCTCMYHIVQSFNIDVRYIIFVISIIHIYLPCRYAANLKCMAAETPTLIYCCGWESVSDLRIKRMMPFMVARSQVIVEITAFNMFAFDMELFVWIMKTSYSMFTLMRS

>BmorOR15

MMTLVYQTDIFKPNVFFWKMFGIWADRKSSKTYKYYSFVFLFITLIMYNSLLAINLLYTPLKIELLIREVIFCFTEITVTTKVLMILFKRNKILDAFDLLNKNEFRGNSEESSAIIQKNNSAYKTYWKLYAILSNFAYSSQVLGPLIVKLIWKTKLELPICNYYFLNEELRHDFFSGWYIYQSFGMYGHMMYNVNIDTFISGLLMMAVTQLKIIQTKLLSLKLNPRERKMDRGLMNITEVLKLNEILKHYELVLKYCSTVQSILDVAMFVQFGVASAIICVAMCGLIMVRSSTETLLFMVTYLFAMTLQIFVPAWMGTQLHFQSQELVFAAYNSEWIPRCQSFKRSIIIFVERAKIPITITGLKMFPLSLATFTSIMKTAYSFFTLIRNMQTLQEE

>BmorOR16

MSFNSEDLYLNRAKFVMKYLGVWVPPENENFARKFYKIFMMSLQHLFLFFQIIYIVEVWGDLEAVSQASYLLFTQACLCFKITVFQINMNKLKELLKQMNGYVFQPKNINQQNIIKVQATRIKRLLFAFMISSQLTCGMWALKPLFDDVGSRKFPFDMWMPVSPERSPHYHLGYSFQLVTICMSAYMYFGVDSVAFSSVIFGCAQIGVIKDKIMSIKPLGIYRNHKTYTKISRYNRKTLIECVKHHQAVISFTELVEDTYNSYLLFQLVGSVGIICMSALRILVVDWRSVQFFSILCYLSVMISQLFVCCWCGHELSATSEELHTILYNCAWYDQDVKFKRDLNFMMARARRPILLRAGYYISLSRQSFVSILRMSYSYFAVLDQTNK

>BmorOR17

MREDKMEINNSQKFYTKMIFRYLYSVGLGDWWYQHEDRSDSHRKLYCLWAVISNAYIFLNICNELLANFRKDLTDVEKNDAIQFSFAHPLIFAKIASFFFNRKKIREVFGRLLEENRSVYSCGELEKESMKQIKRYSLAFIGVSYMTLVMSTIDGLRAHFKEGIPIRTEVTYYPSPSNSGVIVNILRFLVEFHWWYIVSVMVAIDSLAVASFVFVTFKFKLLQRYFKDMGLTVRRDQSNMTDEALADKFRRDFIVGVKLHENALWCAENVQKAFGWVYSVQVFETVALLVMCLVKLVTTNHNMIFLLANFAFMLCVIILNGSYMMPAGDVTYEASEVPTSIFLCGWELVRQTDLRFLVVVAIQRSQVPVIMKAFGIMTLSYSNFIAVSLFKFYVQFQINLF

>BmorOR18

MGDRMVTRGHFFDFNIKYLFYVGLWPSNEAKRIEKIAYKIYEYQLHVLSLIFLVTTGIGTYKNHKDIIALLTNLDKTLVAYNFVFKVIVFVYKREELRKLIEQIVQSGDQITEDRKALMAKLVIVLTGISTVIITAFSCLALFEGEMTIDAWMPFDPMKSKMNLFAASQILAATFVVPCGYRAFAMLGIVCSLILYLRDQLVDLQNKIRDLRFATGNVEKLRDDFKLIVKKHVRLLGYSKVIEMIFKEYFFIQNMAVTAELCLNAMMVSVVGLEQKTLAASFLAFLSVALLNAYIYCYLGNELIVQSEGIAMAAYESSWILWPVDMQKDLLIVITAAQKPMKLSAGGMAVLSVQTYSQTLYNGYSIFAVLNDIVN

>BmorOR19

MHEFVINVQNETTKLYDQLNIILYILGLQGIWVDEIKLSRRFHVFFKVVTFILHIMCGMFAGLQFFAIFTQNSLNSQQKSDVIVIGISNPMAYIFCINFIRNRNEIKDLFYHLAVVLKIYYNDVEIEKSMVNKIKSYLSTYVFASITILVSNGIIAFFQTINSDEPFLGIITAWPDKTDTSKTASYARIGFYLFWCIHFFRISTVFAVIVCILISIKYQYKFLCSYFESLNKIFDDETSSHEVKEAEFENAFCNGIKIHTQIIWCVRRCQIMCRTVFSANIMLDTFVLVILMLAMVNSENDFYGLCSQMSSVLVTVVLMAFFMWTAGDINVQASQLPDAIYGSGWYNCRGKSSARIRSLVTISMNKAQQPILMWALGFVELSHKNFVAIIKSAYSVFSVFY

>BmorOR20

MIQASKYPNSKTKELFRKIAHIAYICGLPNFWIEELNLPKSFIRVYDKIVRIFNVATYFFLGIEIAAHFTQHHLTNKQKFDLLLYSISHPILNGYGVIVSRQVGNVKKVLLDLIVNLKVKYNDPVIEEAMIKISMTYSVSFITNCVLSMLTYTFDALLMVYKKGVTFNVIITAWPDVEDTTTEASIGRIGFHIFWWLFVTRPFAVYVLVINLTTCLSHQYMNLQSYFFHLEDIFKENLSQNEKEAKYEAEYKIGVMLHANTLRCTRRCHMVWNGVMSGQIIFNISLIVIIMAQMMNSDRTLVNTFGTVLTASAILISTGFFMWNAGDVTVQASRLATAMYCSGWQNCRGKSSVSIRNMVMNTIAVAQRPLVLRGLGVIDLSYQSYLSIVKASYTVFSVIY

>BmorOR21

MNKNHYILKTYCDKIFLVGSGNFWYQKTESRNDKTLLYKIYSCVLFFTYGFMTVLEIMAAMMGDFPEDEKRDSVTFATSHTVVMIKFISIIKNKELLKTLNRKMMMICEAHEEQTLMDEMYRTVKINVVAYCVAVYGSATFYVFEGLRKFYNGSHFVTIVTYYPSNDDDTLAATIVRIATTLVLLMMLLTMIISVDTYTMAYLIMYKYKFITLRHYFKRLRENVDELVAAGKARLAAEKLAQGLVEGIKMHNELLSLSKDIDKAFGTVMALQLCQSSGSAVSLLLQIALSDQLTFTMGMKIFFFLAAMYLLLALFLCNAGEITYQVCTSIV

>BmorOR22

MNKNHYILKTYCDKIFLVGSGNFWHQKTESRNDKTLLYKIYSCVLFFTYGFMTVLEIMAATMGDFPEDEKRDSVTFATSHTVVMIKFISIIKNKELLKTLNRKMMMICEAHEEQTLMDEMYRTVKINVVAYCVAVYGSATFYVFEGLRKFYNGSHFVTIVTYYPSNDDDTMLASIVRIATTLVLLMMLLSMIISVDTYTMAYLIMYKYKFITLRHYFKRLRENVDELVAAGKARLAAEKLAQGLVEGIKMHNELLSLSKDIHKAFGTVMALQLCQSSGSAVSLLLQIALSDQLTFTMGMKIFFFLAAMYLLLALFLCNAGEITYQASLLSDEIFYCGWHKCNSPVLSTQRNIRDIVLIAILRAQSPLVMKAFKMVELTYATFILVVRSTYSVFALFYAQNK

>BmorOR23

MRAKTEFEKTIKLTKTALFLSGINIFLGEWNHWTRTFVDSIAYYLNIVGLYFVLIGEMYWLIDGTITGKSFVELSLIVPCLTISVLATAKVHYLYHNKESLLDVVDKLREIYPDEIEETANDNDQCLNDKKETVYDNDVTEVGIVNEANELLKFVNFLLSTVSFVVTMTFCTMPLFGMAGEFMETGKFVVLYPFAVKYPFDVYNTSFWVIVYVNQFWATIIVCTNIFGVDTLFYALCSYIGMNFRLLSYKFEHLEIKRNDRIINEIIVLIKRHQELIELVNKTQSLYSLSTLFNIVTSSLLICLSGFNITILSRSWSYFALLKTIYS

>BmorOR24

MPEELFLDRSIKKIESYFRWMGINIRSGDNNNKKDVFKIRCIYFINFVLLNTDVLGAIFWFRSGLEQGKTFTEVTYNAPCLTFSFLANFKMLSLIFYEKTVHELIAALQKLEIKHFLRQNCAEELKMLKDEKNFLHAVFKGSKIVNYASILTFGCSPLVLIASNYYKTGRMDYLLPLIVLYPFDVDNITVWPIIYVRQIWSVITAVIGVCATDYLFYTFCVYISTQFRLLGHSIERVVPNNGLSVRTRLNGNLRMKFVENLKWHQELIRAASLLEQIYTKSTLYNFVTSSVIICLTGFNVAVVEDFAVILSFLFFLFMSLLQIILLCFFGDKLMKSSTNISDAVYNSKWYLTEKNVGKVLLMVQIRSQRACRLTAYGFAEVNLRAFMKILSTAWSYFALLQSLYSSHE

>BmorOR25

MFEKALRSANFYMRVIGIPTDIRDGNRTLMERLRNRWFYCINFLWLNTDVAGEITWFVKGLLSGSSTLIENTYLIPCLTLCILGNVKTFFTIKYANHIIDLVAILKDLEIKNNAARKNETEIVKERLKFLTTSNKFLLFVIGTGIIAFGIGPLMLTASIYFSSGDMKLKLPFLIWYPFDSSDIRYWPFVYVHQVWSACIACCAVYGPDCFYFTSCTFIHIHFIHLQNDITNVIVESSRARRNGLYRGCHQAFLELTNRHKDLIRCVNLLEIIYSKSTLVNVVSSSLLICVTGFNVMAIDFLPLIAPFTSFLALGLVQTYLLCYYGDTIMCSSTEVSDAVYNSTWYGTNISQMRDYLFVMKRAQKPCKLTAYGFSDVNLRTFSRILSTAWSYFALLITIYRGNGQQ

>BmorOR26

SLSGSSVFTHLFLLRCCGFCRLSRSSTARRGLSVAHEVYRALTLTLTVVYLLQECVYAYQERTDMDKLSRVMFLLLCHITSVAKQLVFYLDADRIDYLIATLDDPSYNEMSHQRLLVDASRWASRFVWAYSGCAVVTCTLWIVFPIIYHVQGQTVEFPFWIQIDYTKSSMFVVVLLYSYYVTTLVGIANTTMDAFMATILGQCKTQFTILRIKFETLPTRAKQALRCDSEQNYDEVLMRLFHDCLKHYQKIVSAILIQFGIGAWILCMAAYKIVNLSVLSIEFASMILFISCILTELFLYCYYGNEVSTESERLVTSIYSMEWVGARLGFQRGLLVLLERARRPVRPAAGLVIPLSLQTFLKIIKSSYTFYAVLRQTK

>BmorOR27

MPSSFFLPNLENPDYPSLGPTLKGLKYWGMWQSGGIKRILYNSIHAFATFFVITQYVELWIIRNNVELALRNLSVTMLSTVCVVKAGTFVCWQKYWSGIIGFVSNLEKEQLSKNDAATQAAIVKYIKYSRRVTYFYWSLVTATVFTVILAPLVGFLSSPERELIANGTLPYPEIMSSWVPFDRSRGFGYWVTALVHTLICFYGGGVVANYDSNAVVLMSFFAGQMKLLSINCSRLFDDGNEVISNNEAMKRIKECHYHHVFSTIFNSLMSPVLFLYVIICSLMLCASAVQLTTDGTSNMQRIWISEYLMALIAQLFLYCWHSNQVLYMALEDRLGGLFEACLESGRFPSKWKTGRLVLLRKDGRPADSPAGYRPIVLLDEAGKMLERIVAARIVRHLTETAPDLSAE

>BmorOR28

MHTLALVFALLYPSNCNIIKRAIGITLIIALSGGQLFWCMTYTFNVCVLILNYSGFDGSFCIASIRLCMKLKLVVYKVQKAFAESKSVSELKHQLNDAIKDNLDALKFHEQIQNVFFIALVGRRAYGPPDGEWLPSPMDFSNTRGRTKPLSTVYEPWLFLIFLLTFLII

>BmorOR29

MFDFLQNLEDSERPLLGPNFWLINKTGLLLPKTNFGKLAYILVHEIVTFFVVTQYVELYVIRSDLDLVLTNLKISMLSIVCIVKVNTFVFWQTSWREVLEYVNEADKFERNQTDETRGKMIETYTKYCRRLTYFYWSLVFTTFLTTTNTPLMRYWSSPIFRENLRNGTEDFPHIFSSWMPFDKNHSPGSYCTIVWHVLLCAYGAAIMAAYDTCIVVIMVFFGEKLNLLRERCKKMLANDLYNHAFVIGQLHDIHVQLIKQSRLFNSLLSPVMFLYILMCSLMLCASAYQLTSATSTAQKLLMAEYLIFGIAQLFVFCWHGNDVLFKNANVSLGPYESNWWSSSPRVRADVLLLCGQLRVRHVFTAGPFADLTLSTFIKILKGAYSYYTLLRK

>BmorOR30

MSVSNLKFEVLFKPTTMSLHMNRSHPSIKRNKIWLLQFISLMTLTAFCATGLITSLLFHDLKFGKYMEASKNGTIAMLSFTTTFKYSLLLYLQKSLNRLIAKIDMDYEIAKGLTPQEKVTVLNYAKKGVIVSKFWLFTAFAITFCFPLKAFIIMGYRFIIKDEFRLEPMFDMTYPEPIESYKTSFPVYFILFVVCFLFGCYASSLYVAFDPLVPIFVLHACGQLDLLSVRITKLFSDTKNPRIIAKELKVIIIKLQELYSFVNFIKVNFSILYEYNMKITTISMPLSAFQVVESLRRGEFNIEFTYFFFGCILHFFMPCYYSNLLMERSENFRFAIYSCGWENHNDKNIRQMLLFMLTRAAEPLGIATVFTNISLDTFAEVNTFDTVLLA

>BmorOR32

MTTRHAEPCAEAPRLSPAAGGMVGLAPVPQPSSNEMLVQERGRPPGLEGEYVANRPFRKSLRGSQEEPRANGKSENVRFLINSHILHCGLRFNETNCHTHYIAKVAIFCFIVTYMLQVMELYWSKGDQEKLFECFSILSFCGMGVMKLVILRVYHQRWRFLLNQVSILENRHLDPGPLSYDSDNDNDDNEIVTFITKYTDKFKRTSSILIKMYASTLVIYVLSPFVEYIFRQFRGDLNIAYPHILPAWTPLDEFSVTGYLIMVSFETVACIYCVFVHVAFDLTCVGLMIFACGQFYLLRYRSERIGGKGRICRLLKSTEVRAHYRIVFCHGIHVLLVQLIEELDRLIKHILGVYFFLATLTLCSVAVRLKTEDMSITQLVNLLQYMCGTLTQLFLYCKYGDSVYNEADEPYGLPDACLESGRFPKQWKTGRLVLLRKERRPADSPAGYRPIVLLDEAGKLLERVVAARIVQHLTGVGPDLSAEQFGFREGRSTIDAVMRVRALSDEAVGRGGVALAVSLDIANAFNTLSWSVIAGALQYHGVPAYLRRLIGSYLEDRSVVCTGHGGTVLRFPVQRGVPQGSVL

>BmorOR33

MELNFDKIFKIAIISQKFSGTYPYTKRDKKWATHFILMHGELTIICMLFIYNIIEFDLKAADYSQMCRNMCLSFVYLVITLLYINMLYYQSKLKMLIETMKAEYELAKTMSEEEQNVILEYAKKGRWLCRAWAILTTCGMAQFFLKSIVCTIYSAIQGNFRIVQYYEVICPEVIERHRNNPVIFITLYFCTFFYSLYTSALYTSVLPLGPIFLLHGCAKLEIVRLNIKNLFDNDDYVVQERLKKTVLQMQDIYCYSHEINECFQILYEFLLKATSLVLPITIFAVIQALGRGQFIPEFFAFIFGAFMVGTTPCYYSNMLMEK

>BmorOR36

MVFNSKKNIISLFSLLEDSRHPSVGPHLRLLSLTGIWYPNSKTNITLLKRACFYVIVLFFVSQYLKCIIKFKIDSLQLILEYAPFHMGIVKTCFFQKDYNVWQDLVSFISKTERDQIAKKDPKSIKTIQSYISRNRKITYSFWALAFIANIGVFSKPYQNNQSDVNGTVTYNHLFDGYTPFSEEPPGYYFSMGIETILGHVVSFYVLGWDTLVVSIMIFFAGQMQMSRLQCSRMINGSPERTHKNIIKCHKFHTDLIKYQKQFNSLISPVMFVYLFVSSINLSVCIVQIAEIEDDFATVLSSFIFLLACLIQLLLFYWHSNEVTVQSELVSYSTFESNWTSTQNKLQKEVALLGLTTSKTLVFTAGSFNHMTLATFISIIRASYSFYALLNSTKY

>BmorOR37

MELGCSRHLKLPCSLHPIGISKHGNTLSELLIYFPAIPKITYAILAVLLTVYYYIYLCSITWFVFVRCPQTGDLAAASIVFSLGVSSEIGAIKLFIMYVYRAKLRDITGEYLQCEADMAPGRLRARVGRSLRTVRRRAFVYWLVLVVNAFAYDLMPAFLPGRHLSEDVFVIYGFEPMFESPNFEIASTLMGVSVVFICYTAGSISAFLIVIVGYSEATMLALSDEISCVWDDACASECQQPNDFIRARLGKIVAIHTKQIRLIREVEVVFRGALAGGFACVAFGLIAALLGGLENTFLQLPFCVIQISVDCFVGQRLRDANVAFETAVYNCKWEYFDKSNMKTVLLILQNSQKTMGLTAGGVAALDFTSLMTIFKSVY

>BmorOR38

MVVFSLGISSEIGSTKFFNTIIYIKELRKLFKDYLLYDATCPAQGRLRLHLLTTLRYVKRRAIIYWLVIIGNGFIFAIKPLLVEGRHLAQDDLVLIGLEPMRQSPNYEIAYAIMTMGVCFICYPPAHVTMFLIIIVGYTEAQMLALSEELKHLWNDAIEHYEKHSRTEREADAAMKSKILNSFVNFRLVQIIKSHSTNVNLIGRVENVFRGSLAVGYVFLIVGLIAELLGGLENTYLQVPFALIQVAIDCFIGQRVNDANIDFEKAVYDCKWENFDKRNMKIVLLLLQNAQKTVSLSAGGIAKLNFSCFMSVIKSIYSAYTTLRTTMK

>BmorOR39

MLWSVFSYFTRADDVLAGIVIFSLGVSSEIGLVKLCFMYANIDKIQKITEGYLKSDAASARNSRFSKNILHTMQSVKKRGVIFWLVIISNGVVYLVKPIVTPGRHFMEDQFIILGLEPKYETPNYEIGFFMMAVGVCVTCYLPANITAYLITVAGYSEAQFLALGHELANLWPDAQLHCRAMNLSQSVNEQANEYVKMRLRELVKIHSTNVNLLRDIEGAFRGAIAVEFLLLIVGLIAELLGGLENTYMQVPFALIQVSVDCLTGQRVMDANLALERAVYDCRWEEFDASNRRVVLLLLQNAQKVATLSAGGIATLNFSCLMAVIKSIYSAYTTLRTTMK

>BmorOR40

MTGAGAGTFRTGAGPGRGDGVARRGESGETTTLGRDAFAALGCFGAADGSTARARFFPRVTVLNPSEVPGSGLAADSNSISDSESEPELDAAQDAIDAGAGVGGDIGESRARTVFGIQGHDASDSALRMHNNVAIYAKTTMSGNSQLTFATAATIFLKNASGPNGVAIGTDYAICVVSLSLFFCYRFTELVEDTYNSYLLFQLVGSVGIICMSALRILVVDWRSVQFFSILCYLSVMISQLFVCCWCGHELSATSEELHTILYNCAWYDKDVKFKRDLIFMMARARRPILLRAGYYIGLSRQSFVSVSIPRIRFNAILVI

>BmorOR41

MMGNSTDLFLDRTKSILNFFAMWRSFEKPIPLKVYMAFIMTTQYLFLIFEIIYIVNVWGDMAEVSEASILLFTQASVCYKITSFISKTNNFVILLGLIESEIFSAQTELHEKILILKARKIKRLCMFFLVNAVTTCSLWAVIPLLDISSKMLPFKIWMPASTGESPHYELGYLYQMITIYISAFLFIGVDSVPLSMIMFGCAQLEIIMDKIGKVKSRPLDQQPMQRQAVLNSNYELLVECVRRYQSVVRFIELTEKTYHANIFFQLSGSVLIICNIGFRIAIVDSNSLQFYSMLTYLVTMLSQLFQYCWCGHELTIRGEELRETLYQSPWHEQDIRFRKVLIITMERMKRPIIFKAGHYIPLSRPTFVAILRCSYSYFAVLNRVRNE

>BmorOR42

MDIPKFEELLKQIKMNFWLMGIPFDNPKIQIRYYVLLLPLSLMLIEEIAFFGSRMSSENFLELTQLAPCICIGVLSVLKILALTAKRQKIYELTQNLECLHKIILNDTRKTELVRKNLVLIKFITKYFFVLNAVLIFVYNFSSPVIIAYNYIVSNEVQFVLPYAVLLPFKTDSWIPWLIVYVYSIFCGFTCVLYYATVDVLYCVMTSLVCNNFSLISFKLQKVNRNTAHLLKEVVKEQQYVLKLAEDLENIFTAPNLFNVLIGSVEICALGFNLMIGDLTQIPGCILFLSSVLLQILIMSVFGENLISESSRIAEAAFLCKWYEMDQKSKKTILTIMIRSHKPKKLTAYKFSVISYGSFSKIISTSWSYFTILRTMYTPPGTKFQDDL

>BmorOR44

MYTYFKVLVFWLNKDKVISLQKILHCKEFKPKEPEHKEIIRKSIRKARFVMTSYATMCVGAVSVGIILPLTENFDILPTNVEYPFFDVYKNPTYAYLYLHHIYYKPATCIIDGVMDTILAAFVASAIGQIEILAFNLRNFDVLAERRRKRAISGNKYIGKYTNLYFTKRILKECILLHNSIIRYVSVIESAFSLASALQFMLSVMVLCLIGIQFLSIENPTSHPMQMVWMAIYLTCMLIEVFILCWFGNELIWKSNDLRQAAFDGPWRNLNRKTCMFIIIFMERCKRPMRLSAGKIFTLSLDTYTVLINWAYKAFAVMRNMKK

>BmorOR45

MKVLDNVNHAVKVTMNCCRLYGLFVSDDLTKRQLIIMRAFSLMLYLFFVGFFITTQSALIITMWGDLNLMTNVGLVLGTHLTLSAKVFTLHYKEKEITNVIYKNEVRLRAETREQGKYIISEYPCNTTKSPAHEIILAHQGIAVILTATLEIAIVLLMTSIVAVCRCRLKLVGLSFETICDDLPSNIMNKLTADEQVIVAKRVRENVIEHQAVLECINDIQDCFSSAMLVHIAISTMIICATAYQLAVEKSLDLTQRMTMASFLGGMSTEIFLFCYQGGHLSIDSMEVATAVYSCPWYTFPTSLKRSLLVIMIRAQQPALLTAGGFAPLLLDTFVSIMKASYSFFTVLQNASE

>BmorOR46

MAFFIRNKMLGLTITLNTLSWAGLIMRDQYTKTQRIIVRVYGWLVFLYLFVAATYVQIADLIDIWGDLDLMAETSLLLFMELAVISKILTLIFKYDKIMEIINGTEDILCSENRLEGQKIIASIDKETTRFFQYYTSSVIFTTFFWFLGEHSSTFFIRAKYPFNELKSPGYEFALIHQCMMMVFTGYFEFNINIFFASVVAGCRCRLKLVALSLRNICINIPVNKKNLITPEEEKLITERLHCAISQHKYALDAAEDVKHCLSKVLLVQLTVSIVIICTTAYQMAVNKSTDTIQKLSMAGYLLGASFEVFLFCFQGQSLSNASEDIADAVYECPWYTLTQPLKRTLLIIMMRAQSPAILTAGGFVTLDITEYMAVLTGHGGFGDFLHRTGAEPMAECHHCGCDLDTVQHTLLVCPAWKGWRRDLVVKIGNDLSSVLWHRCSAATSRGRRCLTSASAPSRRRRRGA

>BmorOR47

MKLVFDNFISALKVTLNWSRYIGIFIPDELTGRRQKLLVQAYSVFMYYLFIGFFITTQIISFILVWGDLNLMTDVGLVLGTNLALSAKIAVFFFKREDLANILKKNDDTLRSETRAEGKKIISETSCESARVGTTTLPISAVVTILETLELISQASLLITVDIIMLSMIAVCRCRVKLVGLSLQTICDDLPCNVKNKLTSDEEVIVAKRIREYVIEHQAILDCISELQNHFSPALLVQLLTSVVIICVTAYQLAVEKSSDLLRKFTMASFLFAMSTEMFTFGYQGGHLSHDSMEVATAAYSCPWYTFPTSLKRSLLVIMIRAQQPALLTAGGFTTLSLETFVTIMKASYSFFTVLQEATD

>BmorOR49

MLTCFATIFSAVNQTGYIVLFINLLAHELGHFYVITDVLNGIFEKNDADRDPVFIDRKLKFCAKHYQYLLKFHNEIKNLYKIIFGAHFLMMTIVLVTTLQTMNSWDIRNTVLTAVTGIMPLFIYCFGGELLITAGMDMSTAIYQCGWEKMGVKQAKVVSVILCLSQRPLCLTAANVFVMNRETFGGIAQVVYKIYAVFN

>BmorOR50

PIFAAKTLKDKQHLIQNKKEVTRFARLLLTYVTVGGFIWPMSFCFRRIKDPNTVVPFYVPFTPDNWTKLINEVTDIFNPCLTFQFFTSSVAICMVIYKLSDTYIVSLEFVFLLNFIFVLLTQMFIYCYYGNVVSYESKYINTSLYLSDWSSASPGVRKMFLIVMPRWTRPLVVRIARVVPLSLDSFVSVRKYKCSIEHFIYSHVKQTRARDIFSGASNKP

>BmorOR51

MDCTIVAFYSQAKTQIKMLRYDLEQLGKIDNIETKFTENIFERSSHIWKALKDEKIKIHSKLVFCVEHYRQIVWFVKEVESIFGEAMTVQFFVMAWVICMTVYKIVGLSIYSAEFVSMGVYLGCMLAQLFIYCYYGTQLKVESESVNTSLYCSNWLSSLPKVRRQMLIMMQYCSKPLTPRTAYVIPMSLETYISVLKSSYSLFTLLNQKH

>BmorOR53

MALKKMLALTKGLEDPTHPLLGPTLKALSVFGLWQTGSQKSTVIYNTFHFLTFLFVITEYIDLYTVRKELSKMLNNLSVTVLSTICMIKTLSYVCRQSHLKVLVREISELELELMKTTDKNIVKRLRQYTVYTRAVTYVYWFLVVGINVVLLTSPLLKYASSEIYRSEIKNGTEPPPLILCSWFPFDSARMPGYFWATMVHIIMSIQGCGVVATYDMNAVAVMSYLKGQTSILKDKCKAIFDETASSRDVLNRIRDCHRHHNILLRHYYMFNSLLSPIMFVYMLICSFTICCSIIQLDSSETTISQRIWIIQYSIGQISQLFLYCWHSNEFAAKVKKKHFPLFPINLF

>BmorOR54

MGLNTIKEFFVNVKRRFQDVSIDSLLWIVNIVPSLAGFSIRSDRVSAPFWIVHWSLLVYVYAVGNAVYQWKFANEAIDYITSFINVSLLILIGNNSWWFLANRRLLKSVLHKIEVNDELSRRSEQSRLKHKKLLKIIKRIVLVFYMSNYVNASFIYLPNRVDVLNNYAMTPCVGMEPLTVSPNRELCLTILCMQEFSIMTVVLNFQALLLCFIAHTAVMFQILADEIMALNNYENLEEHQAYVKEMLPIFVKRHSLTLSAVDNYKSLYSVPLGVNFGSNALTILLILYLPVLEWFKFIPIFVFCFMLFFLYCFLCQKLVNASEAFETAIYCCGWENFALREMKMIYVMLHQAQKPVELLAADIVPVNMNTFATTLQAMYKFVTVVKF

>BmorOR55

MCFLKIKQQIIDIQKHFKDYSLNGSLWIVNLLPRLMGFNLRADKVGVFFWTIYILLLVYVFGIGIFVYLWKHVDTMSGLMKSYLNLSLILVIVNNSCWFLSKRSLLNKVLKKIHLIEDLSCESEHALAKYRRVFKIVTHLLLASYVLFYFTEIYFMFLFRNYDLLEDYSLAPCVGLEPLSSSPNSEICLIIVLIHEFISTTVMMSFAALFLVLIAHTAVMFLVLAEDMTKLTDLINLADHRKMIRESLRSLIHRHSLLLQIVYELRLLYSVPLGINFISNAMSILVLLCLPIHEWPSFLHIIGYCFFAFFLYCFLGQNVINASEKFIDAIYCCGWEHFGVAEKKLVHVMLRQAQKPVEIIALGMISVNMNTYVEALQLIYKFVTVLKI

>BmorOR56

MKLLEKLEDPDRPLLGPNVKALKFWGLLLPESRSKKYFYLFMHFAVTVFTATEYIDVWFVKSDLALLLNNLKITMLATVSVLKVTTFLLWQNAWRDLIGYVSRADLEQRATSDSRKLALINGFTGYCRKITYYYWFLMYTTVAIVTVQPIFKFFSSAAYRLDVQSGNGTYLQVVSSWIPWDKNTLPGYLLASIYQTYAAIYGGGWITSFDTNAIVIMVFFRAELELLRIDCAALFDDEKSFGDMAFMRRLKECHRRHTELVKHSRLFDSCLSPIMLLYMFVCSVMLCVTAYQITIETNPMERFLMTEYLVFGVAQLFMYCWHSNDVLYASQDLSRGPYESAWWSRDVKYRKNLYILVAQFNKVIVFSAGPFTKLTVATFIRILKGAYSYYTLLSQSQMNKT

>BmorOR57

MPSLIKNRIFGLTLTLNTLSWAGLILRDDYTKTQRIIMKVYGGLVFLYLFVFTAYVQIADLVVIWGNIDFMTETSLILFMQLAVSAKVLTLMLKSKKIMEVTNEADAILISEKKVEGQRIIASIDKNTTLFLKYYGFFVAFTIICWFMGENTSTFFIRSKYPFNELKSPGREFAFVHQCIVVIFTGSFDFNVDIIIISLVAVCRCRLKLVALSLRNLCLDIPMNKRNLITSDEEKVITERLRNIISQHKRALDAAEAIKHYLSGALLVQLMVSIVVICTTAYQLAVKKSTTMQSLTMAGYLFGTSLEVFLFCYQGEFLRESSEEIADAAYECPWYTLTRPLKKTLLIIMTRAQRPATLTAGGFVTLDITEYMAVSLISNT

>BmorOR58

KLVFDNFIFALKVTLNWCRYFGIFIPDELTGRRQKLLVQAYSVFMFMLFIGFFIITQIILFILVWGDLSLMTDVGLVLGTNLALSAKIAVFFFKREELASILKKNDDTLRFETREEGKKIISEYPCDTKRSPAYEIIMIHQTIAVAVIASLAITADLLMLSMIAVCRCRVKLVGLYLQTICDDLPCNVKNKLTSDEEVIVAKRIREYVIEHQAVLDCISELQNHFSPALLVQLLTSVVIICVTAYQLAVEKSSDMLRKFTMASFLFGMSTEMFMFGYQGGHLSHDSMEVATAAYSCPWYTFPTSLKRSLLVIMIRAQQPALLTAGGFTTLSLETFVTVS

>BmorOR59

MDTNPSAAGDSVAPHLRRLRQVGFCQLDPTSQSRRPILALMHRVYHRLVLAATVLYIFEQLTYAYQARNDMERLSRVLFLMLCHLTCIAKQFVFHSDADKINQLVVGLDDALCNQPVETHRLLLLETSRRAARLLMLYSGCAVSTCILWAVFPLLDQLRGRTVEFAFWIPIDYRHNAFQFAVVLAYAFYSTSLVAVANTTMDAFIATVLYQCTTQLRILRMNFESLPERAYALSRKTRQDYHTVTHELLVDCLLHYKKITETCNLLEQIFGKAILVQFGVGGWILCMAAYQIVDMEILSIEFASTALFMGCILTELFLYCYYGNEVTVQSGLVSESVYAMSWLSLCPRERRALVVVLERARRPLRPAAGRVVPLTLNTYLKILKSSYSFYAVLRQTK

>BmorOR60

MVRPCRYFAIHFILLRFLGLGWWHHPHENETRNYPGLYLYYSILTQLVWVVGLVGLETIDPFVGEKDMDRFMFSLSFVITHDLTLIKLYIFYFRNVEIQDIVRTIEIDLYRYYQNDDKIRATIRISRIFTAAFLFFGWVTIGNANIYGIVQDLRWKDIVKNLNETTSKPLRTLPQPIFIPWPYQEDKHYILTFILETMGLLWTGHIVMTIDTFIASVILHMSTQFAILREAIVTAYDRTMIALSEGALQSGVLCENSNGNEENNQIFLESFYSKEHIESVLESTLLSCIRQHQLLIGCVEKFSKTYSYGFMTQLLSSMAGICVVMVQVSQGASSFKSVRLVTSLAFFFAMVIQLAIQCFTGNELTIQAERIADAVMESKWEKMPVRLRRLLLVTMMRAQRPLHLTAAGFAYIDNTCFLSILKAAYSYYAVLSQKQG

>BmorOR61

MARITDVFRLNFIFWKFLGIWGKSAPSKYNMAYTALYLSASLFVYDIFLTLNLIHTPRKLETLLRETMFYFNHLVAMTKILKMFIRRKKILVIFDLLDCEEFKPSDEDSQEIMKRKNEFYYIYWRIVAVTSNLSCFMQVVGPLIKMLIWKSELGLPVCKYYFMSDEFRNKYFVIWYIYQSFGIYNQMVNNLNLDTFNCGMLWMAVGQLQILKTKFVNFKLNDIENSLDLKTRDDMQTERLRKYLTHYEIILKYCATVQDILNITIFVQLGMSSIVICVGLCGFVAMPSNTETAIFMSSYLITMTMQIFVPSWMGTQISFECGELMSAAYCCEWIPRSKLFKRSLILFVERAKTPVRITGLKIFTLSLDTFTSIMKTTYSFFTLIRQLQVDEVN

>BmorOR62

QNVILEYAKKGRWLCRAWAILTTCGMAQFFLKSIIVCTIYSAIQGNFRIVQYYEVIYPEVIERHRNNPVIFITMYFCTFFYSLYTSALYTSVLPLGPIFLLHGCAKLEIVRLNIKNLFDNDDYVVQERLKKTVLQMQEIYCYSNEINECFQVIYEFLLKSSSLVLPITIFAVIQVSSLHICFIFLIPNTQWNVCSQNKHFLGSLEMSCRNCHSK

>BmorOR63

MKLWIRNANFTISLSLTLLRCLGFWSPDGLAGNKRLLYNCYSFVFFMFLLGIYILIQVVDMIKIWGDLPLMTGTAFLLFTNFAHATKVINIVIRKNRIQRVIQQANAVLMGVQSEEARRIVKSCDFETSIQLCLYFLLTFVTTVGWATSAEKHQLPLRAWYPYDTSKSPAYELTYIHQVAALLIAAYINVAKDSLVSSLIAQCRCRLRLVGLALASLGQDLKIDYQSQLSPAQENILNLRLKTCVLEHQTVLAAVTELQACFSKPTFAQFTVSLIIICVTAFQLVSQTGNLVRLLSMGTYLMNMIFQVFIYCYQGNKLSVESSEIAGSVYFSPWYLGSVKLRRALLIVMVRSRRVAKLTAGGFTTLSLASFMAIIKASYSLFTLLQQVKQKK

>BmorOR64

MGVSNGRGTVKPFLYPLVDELDYNLIVGVHLPFEYKTPSRYPLAYITVVIAFIYVSYFVMVTDLIMQAHLLHLLCQFNVLADCFENMLNDCVKGFEGPLVSLHEYIHPLIDEFEYNLMVGLRLPFSFDTPLRYLFTYVIVLIAFNYTAHYVMVTDLIMQSYLIPLICQYAVLADCFENILIDCSNDYGDHARRNDIVYSRSMELRAILSRPMLGQLASSGLLICFVGYQATTSISVNIVKCLMSLFYLGYNMFTLFVVCRWCEEITNKSLNIGNAVYCSGWESGMTVVPTVRSTILLVILRANKPIVFTAGGMYNLSLTSYTSLVKGSYSALTFLLRIQHE

>BmorOR65

MRLGFEVSISEYLYRNIFYIYTLFHILLHFYYILHMIKLDLEAIFDDIDESVALLPHRDTRRIEVQKILNGRMKRVVTWHISVFKAVEAVSSIYGPPLAYQVMFTSIAICLIAIQITQKLENGILDIRFTMLGVAACLQMWIPCYLGTLLRNKAFGVGEACWNSGWHQTPLGRMIRQDIIIVLLRAQQPVTIKFPGLQSIQLETFSSVIFNLYGYYYFLLLRWVDELTAHLVLSGYWSP

>BmorOR66

MRFGLKVYIYTLFHILLHFYYILHMIKFDLEAIFDDIDESVALLPHRDTRRIEVQKILNGRMKRIVTWHISVFKAVEAVSSIYGPPLAYQVMFTSIAICLIAIQITQKLENGILDIRFTMLGVAACLQMWIPCYLGTLLRNKAFGVGEACWNSGWHQTPLGRMIRQDIIIVLLRAQQPVTIKFPGLQSIQLETFSSVIFNLYGYYYYYCLDG

>BmorOR67

MRFGLKGGAAVVTILETLELISQGGFIETIQVTFGGQLSSMLFISACIICSTAVQILAIESPLDNLTTVGWILVYLSLCILILFVDCYFGNTITVKCAYLPTAVFSIPWLDQPKNIQVSTLLFMAKTQQPVQLIAAKLVPVSLTTFTQVSYCPPLDLKCLQGGVIAHLAKD
