# Supplementary material for: Identification of chemosensory genes from the antennal transcriptome of Indian meal moth Plodia interpunctella
Source: PLoS One. 2018 Jan 5;13(1):e0189889. doi: 10.1371/journal.pone.0189889 (PMC5755773; doi:10.1371/journal.pone.0189889)
Supplement: S5 Table — (DOC) [file pone.0189889.s005.doc]

>PintGR1

LKYDNPKAIRLILSTASRTVTSIETTHFKMSLYSSNSLFPTSMIPIPNGIASQMDEKPKNKIVYLDVTPARTPNMKPYSPNAVIPLNNNLVDPQISRDIIYENIKPVFSILRIMGVLPITRPKPGISQFLIASPAMAYSAIVYISLVSYVLYLSLNKLQIRRTAEGKFEEAVIEYLFTVYLFPMIAVPIMWYETRKIAGVLNSWVDFELMYRQMSSRPLPIKMYKKSLAIAIIIPALSTASVIITHVTMVHFKLIQIIPYIFLEILTYILGGYWYLLCDILSVSANILAEDFQQALRHIGPAGKVAEYRAMWLRLSKIARDTGISNCYTFTFVNLYLFLIITLSIYGLLSQISEGFGIKDIGLAVTALCSIFLLFFICDEAHQASHNVRTIFQKKLLMVELSWMNADAQTEVNMFLRATEMNPSQISLGGFFDVNRTLFKSLMATMVTYLVVLLQFQISIPDENQGQGGEYEESAANVTSAVTEVMTTTTTVATTVLTTLAKKKKNKH

>PintGR2

MKSKSKTDGSIGWILFVDIASFCLPQPKNKLLIWKTYIAFKNFKIAILKFLNAIYVFQINNLFFGCGKQKLAISTKSIQPMLPSVLDLDFIQLVGKVFHYSCWFGVVGSGRPLWRVWSVVVLIMLVAIQISAIWKVIRALAGWTVDVSGSRSVTARLAGTMFYSNAILCHILTSRIAASWEKQSNLWIAVERAMTLNIPPDATIRRRLIIVIIFMTVCGCAEHLMSVVSQIDFDQPISAILKQYTLNSHGFLLMNSTYSHWLALPLLFMSNIATILWNFQDMIIILISLGLKSRYHRLNQFVAKICANEKQKSQCDIFEPIRVYTWRKIREAYVKQAMLVRTVDRRIGAVVMLSCFVNFYFICLQIFLGITQASQGLFKQIYYMTSLLWIITRISSVVLVAADVNVYSKMALKYLYGCHFKHYNIEINRLQTQLTRDYVALSGLGFFTLTKTILLQMASSVITYELVLIQFDNNESNDSVNSTKFD

>PintGR3

LLDKHDSFYITTKSLLVLFQIMGVMPIMRVPKDTKTTKRTTYNWISKATLWAYIIWSLESIIVLKVGRERLSTFQQSSNKRFDEVIYNIIFLSILIPHFLLPIASWRHGPQVAIFKNMWTHYQLKYLKITGTPIVFPNLYSLTWGLCFFSWGLSIAVILSQHYLQDDFELWHSFAYYHIIAMLDGFCSLWYINCNAFGTASRGLAMNLHKALEAEHPALKLAQYRHLWVDLSHMMQQLGRAYSNMYGIYCMVIFFTTTISLYGVLSEILEHGLSYKEMGLFVIVGYCMTLLFIICNEAYHASRKVGLEFQVRLLNVNLGSIDRSTQREVEMFLVAIAKNPPIMNLDGFTNINRELFTANISFMSTYLIVLMQFKLTLLRQSARKALRTIVSAVFNTTAMMGDDDDDDDDD

>PintGR4

MHFRTENNTNESVVDNLVKAFAPFHRLQSYLGITRVNIRNKLVTPLTMSQKLYTCAFFMTASIFLIFMMLLYINRYSNDMILVKVIIIGGFGVWMSLVCTITNLRFINNDGNVNFYVKIHQIDRLLRTDKNEFMTSVLFNNNRNTMLVLLVAYIVLFGLIILFNEFVIILTTVGICYVQLTYMVEFLYFSSIMYYFCQRIKYLNAIILNHLEPEFVRVNFANKLRISDEMLMRRSAADAHDFVYSDVDNYVKELICCFYKFQNIFQYQILFFSLRLFFGCMCYIEFIVVSLKDRTFRITDDYIMIFLLIIDVLLLSLACVRCETFFREAKLTKRLSLSILAIYKDGLLRGRANRILKILEEHPPIFSVYHMWNLKASTLIQLISILTTIFFNILTLNYLE

>PintGR5

MQIHDVTITKNVQNIFSSFSTLNIFLNIFALHSNGESGTIPNKYYVIFRSVAATTILGSINFYTLYFKVSNVYDQINASIKYTDAVQMVYDFWQYTIDLYFVYQYGREMSVEYFKLYGNIDKTLGVHYYSALRRKLLKLISIFLCIWLLSSICDFAAWALSIGWIIPTMFSVAYFYLLIKILTTLDLSAHAFHIECRLRSICDLLQDCYRRVDCSSGAVDYIHNKDWLYSDACTRVLKLQMQYDPNKIPYNISRDVKSLSKVYLMLLEQVAFINTMFGVRILLNILSFLIDMIRFTNIAVRVLIGSQHTTYDTGYWPGLSSTLRLLTCVAVLLSLVCQCENVYRQNDNLLNVIDQQEAYQQETIRSLTARRNATPRPDQI

>PintGR6

MCIYRLTCSATSLKGATPIIFYGTTCITMMMFFHMAKAWPSMVQHIARAEQLDPIYDKNLTKRCNVTCLVVLLLALIEHILSLLSAVAETMICHPEKAFYEGFVKQFYPWVFNFIPYSPYLAGFTQFLHFQSTFIWNFSDLFVICMSYYLTSRLQHINEKLLEAQGKYLPESFWKSTREDYCRATQLVRRVDNVISGIVFISFANNLFFICLQLFNTLEDGIKGTGECSSRSKRTTLLGGYEAASYFVFSLVYLISRSVAVSLIASQVNSASTVPAPVLYDVPSPVYCVEVYIRLR

>PintGR7

NYMRKLKHLVVVYVLKYIRYTYKHSPIITQYIFRIVAVTLYYMLTDSGYTKGKISKETDFMHILLFISFAFHVVVNIGGIFAAPFRMRRLLRCMSTIASIDQSIDAQYSEAKEHKLFVCLAFFIIFFVFIDVFEYFSAVVRFRNPNINWQFLLFDSAINITKFLNMMLTIQFAFVAMSVRVRFTAINRVLNLIAKHLSVPYCLYFLFISCFMESFYSRTARTSLTWIITQYLARSGTRHELTVWSNVTTIPLR

>PintGR8

AGVQDTLLSIDVLTVDRPTQKEIDHFIQAIEMNPAVVSLKGYAHVNRELLTSAISMIAIYLIVLLQFKISLPKDPQGST

>PintGR9

MMKECPSQGALVSRLKVFSLQLKMQHVGYKPMGFFTLNRTFLASTLGIMTTYLVILLQFQNSHSR

>OfurGR2

AVYGALSEIVDHGIGFSFKEIGLFVDTVYCSTLLFIFADCSHNSTQKVADGVQETLLSIDVLSVDRPTQKEIDHFIQAIEMNPAVVSLKGYANVNRELLTSAISMIAIYLIVLLQFKISLPKDPQMPAT

>OfurGR3

LVRMVDAQIGALILLSNINNFFFICLQLFLGLNKTGGSLMSYFYYFLSLGWLLFRACSVVLAAADVHIYSRRALEYIRLCPDSGYNVEIKRLNNQLSHDFVA

LRGMGFFWLSRQTLLEVAG

>OfurGR4

LFRGYEQAIYFVYSCVFLVARSLAVSLIAAQVHTASTKPAQALYRVSSSAYCVEIQRLLDQIHMDTVALSGIQFFNVTRGLVLTVAGTIVTYELVLMQFT

>OfurGR5

KTTREDYCRATQLVRRVDEVISGIVFISFANNLFFICLQLFNTLEDGIKGTGECSSRSKATSVKLLGGYEAATYFLFSLVYLISRSIAVSLIASQVNAASMVPAPVLYEVPSPVYCVEVQRFLDQVNGDNVALSGLQFFSVTRGLLLSVAGTIVTYELVMVQFTTPSPPAVSNSTSGVLANLTSTAMPTIATG

>CpunGR1

RKYFTAENNDLSFFGLFKPLYVMLSFVGIFPYSLRHKTKHKYAIIPSTIYVRIILIVSLLTVLIVFLVLRTIYKLNEKDMFFGFIVFANNIVEMLFGLPCCMVGYFCVYRNRYKVIRILNAMESAWFQLPMTQNDILKTLRSRIVFNFIVMSCMLVAQIIVTLLRSEGFWKVLLVLMTFIYPQIILFLMLMFYDVYVLMLVALLANVEESIKLLGRNKCIGSVLGSITIVESKVTVVSLRAIERIFVNAYEIKRDINDVFQASLLMMSLQSFHAIISASHIIYHAFVFKWGLTLPQSLIYTLWTVYHCMKIYAVSYSGNLLSTAIQHFTSLISYQANEITVYGFFSIDGTFIFNITASTAMYFIILVQFDTS

>CpunGR2

MFSSAKKYFTDKNQDLTLLRLFKPVYIFFTMFGLFPYCMKFKTKHICVIIPRSIYLNILCAITVNIVLCIFGGMDIRNKTIEIIAGEGATVTQVNYILEVVLLQISCFSSYISIYYYRHRLVHILNDMASTWLELPSTNNRILENLRSHIIVTIIIPLFLTFLQIIMNCVWRVEWSTTLITIAYTCLEAVQLIALMFYYLLVLMLVALLSNVEEAIKTIHKTKPVSDNFTKIDGRVSTIQVRSIELTCVKAFEIKREINDAFQATILLNCLQSFHSIVSETHGIYYGLLVDKSLETDVVINVTLWVLCSLMKIYAFAYSGNLINLVVSGKPLR

>CpunGR4

MGQKHFKKNLSLWIPAKNNKVHAAKLKPVRKQATFQKSLRMSLIIGQLFSLIPVNGVCSNSAANVRFILLSWKSLYSFLSLFGQGFMTVMCIHKVVHSTTSLSNNALVVFYGTTCITMTMFLIVARAWPTLVQHIARTEELDPNFDSSLSYKCNLTCAIVLMLALLEHILSLLSAFAGAMVCQADKDFYEGFVTHFYPWVFNVIPYSVALGALTQFLHFQSTFIWNFSDLFVICMSYYLTSRLEHINEKLLAAQGKYLPEIFWKNTREDYSRATQLVRRVDEVISGVVFISFANNLFFICLQLYNTLEDGIRGTEQCSSRTKTAAHLLGGYEAATYFLFSLIYLMSRSIAVSLIASQVNGASTVPATVLYDVPSPVYCIEVQRFLDQVNGDHVALSGLQFFSVTRGLLLTVAGTIVTYELVMVQFTSSSPSSTNSSVNTLPNITTTMISSTAMT

>CpunGR5

MAGKNSKKTLQTNLILPMQPHHDEFLETMTKIFKWSQFIGIVGRGSRIWKVWGLLLLLVLLAIEGGAIWKVIKALAGWAVDTTGQRSVTARLAGTMFYSSVVASQILCTRFAWSWGNLSSFWVSVEKTMALNIPPDPKLRKRMLTALGIMALCSIVEHTISVISLVGFDCPPELILRRYTLRSHGFLFLRTEYSVFFAIPLLLISNIATVLWNFQDALVVLICMGLTSRYCRLNQYVAKFCVEENKLAKGNNMKAEAVRLYSWRKIREAYVKQATLVRKVDESIGGIILLTSFGNFYFICLQLFMGITEGLFGTVVIKQVYYFVSLFWIVIRFTWMVLAAAEVHVQSRNALQYIHTCHSRYYNVEIERLQNQLNKDYVVLSGMGFFSIDRNILLKMATAVVTYELVLIQFDNKGGADSAPANGTGQQ

>CpunGR6

IIRLVKLINEQIGIYILICFGSNLYWICTQLFYSLNKSQTGHFVSCSSKGLDAFKAIHGIEHTVYFTYSFSFLVTRTLLVLLLAARVNSASTKPLVLLYEIPSSRFNIEVERFIAQINNIKVALSGLDFFYVTKTMILTLLGTMVTYELVLLQFNK

>CpunGR7

NLIGRSSPQNMKIRDLAVTYDIIGETCSMINEVFNFQIFMTLVATFTYIVITIWSSLYYYRTAGDNSISLATIIIWSITVIVLIAFMSLTCERLLLTRTDTKILVNKVIMDYDLPKEMRVQAKAFMELIEAWPLRIFIYDMFSVDITLMLKYISVATTYLIVIIQISHFI

>CpunGR8

LYKNIVEIAENGRKAFDVMMVMALVFNTIDVTLTVYVTFATDDIRLGNPLHKSLAYVLVVHELVMMFAPAFTAGLVSSQVENLRLVLFDRLVKDQKHTKDIKRFISYIDARPLRFKVWMVVPLDWSLPVIVVNISVTYLIVMIQFTH

>CpunGR9

SQDLERKLQLTKQESSGLHAAIRLTLFSARLMGLLPLEGLMNLRSYGLRFTLRTPYALLYLIGLFGQVLMFVMSFYWLLHNKMSLSNMTNFLFYSSSAISLVILANIGRYWPTLVMKVEALE

>CpunGR10

PLYCNLTMVFILLAALVEHLFSIFYGLTVAKACDPNNTAETFFNYGWPWIFTYTSYTLWKGILIELFNIQSTFIWTYNDLLIMVISIYVTEHFKIFNFLFKESLKQEHYSCDEFRTQ

>BmorGR9

MPPSPDLRADEPKTPCLVGGAHAFILKISSFCGLAPLRFEPRSQEYAVTISKGKCFYSYILVTFLVICTIYGLVAEIGVGVEKSVRMSSRMSQVVSACDILVVAVTAGVGVYGAPARMRTMLSYMENIVAVDRELGRHHSAATERKLCALLLLILLSFTILLVDDFCFYAMQAGKTGRQWEIVTNYAGFYFLWYIVMVLELQFAFTALSLRARLKLFNEALNVTASQVCKPVKKPKNSQLSVYATSVRPVSCKRENVIVETIRVRDKDDAFVMMKTADGVPCLQVPPCEAVGRLSRMRCTLCEVTRHIADGYGLPLVIILMSTLLHLIVTPYFLIMEIIVSTHRLHFLVLQFLWCTTHLIRMLVVVEPCHYTIREGKRTEDILCRLMTLAPHGGVLSSRLEVLSRLLMLQNISYSPLGMCTLDRPLMVTVLGAVTTYLVILIQFQRYDS

>BmorGR13

MEDSFNRLLSIRNMIIFQNVCGFYHMCTEKLYISRIIKMYCVALAIVLSVFCFQNPDITYLSWDVVWVTFGYTLNVIICLRYNGNYFFQYWNGLHEIDIKMNLTSIDKEKVPISRAVFTVFLILRSTAFAMTIFVFGYLETGILSNTIISIYSINLTEFYRNMSNIPMILMFETFYVRIKILKEQLCSELSTVLGCNNDARQLKLILKYLRNYRSLVRHLMDTTLPFKILILVILVGSFLRSLLIGYAFVYNSDQIILLSLPVMFSTKILSEVVEIKLICTKELLKNKNEGLVLLDLDSKKPTFLTSKACGEQLQDALSFLNNRSYSYTLLQVIEFDCSLAFVFTSFCITHLIVVVQFTHVLD

>BmorGR14

MNLHKNIIPIRNNLFANKVTAIALPKTLSVLFKLIHIFFLLDLGVYEYKTFKIKCIVKFLTISGSLTISVVCFSFMVSNLSEHTFVGWYGFFISTYIFVVLFFNLSNRMTFVEFYKTLLRFDANYGIDSNEYKFNFKIIFVNILFIANRMVLSFVYCSYYPQNCIRPRYAQILFMLPWLTLDVLLTTNMFLFYATYCRIAKFPMLIKNSMNIVALRNSYKLIVDSLEKTQTSFDIVFIIALVFSVPEIMMSIYSTLLEVISKHFLEVASILSLNYVAIAQSLLLTLAPSLCAGVLPWKTNNIKIILHEKLFTEKDKASAREIELFIKYIESRPLKLRACNLVPLDFSLTIIVLNICVTYLIVIIQFTHLY

>BmorGR15

MISSSDINHKRNKVFAYNVPGIALSKTLTVLFKLLHYVLLLDVGIYEYKTFKNKCIVKFLTIATGVSVSIVYFCLIATVLRKNAFFYWFYVLFISQYMIIVFIFTLSNGMSFTDYYKMLLRFDAKYQINSNNYYFNIKIILVIIISILNRIGMAIIYCSYYTKNCYEMSFSQIIFVLPWLTRDVILIMNVFLFYVTYCRITKFPALLENTKNVGSLRNSYKLIVDSLEKTQKPFDFVFTISLVFNIPEIMLSIYFTLLQVIHSHFLEVAPTLSISYFSITHSVVLILAPSLCAGVLPWKTNTIKIVLHDKLFLEKDKNSARNIKLFIKYIEARPLKLRACNLVPLDFSLPVIVLNLCVTYLIVIVQFSHLS

>BmorGR16

MIMNLTTDRISKRNKVFAYNVPEVTLPTTLKVLFKLIQFTLSLDFGVYKYKTFKMKCVAKVLTLAGCLAASAACVSLIISNIFENQLFFGWYTLFVCQYTIVIFMFTFSNGMTFIDYKMMLLRFDAKYQIDSNVYHFNIKIVLVVVISVTSRLFLCAVYCIYSTENCIKPWYNQLLFFPWLSLDIVLIMNMFLFYATYCRLAKFPSLFENPKNVVPLRNSYKLIVDSLEKTKKSFDAVLIAALIFNIPEIMMSIYYTLFQVMNKHFQEVAPVLSLSYFTIILSVLLILAPSLCAGVLPWKTRHMRLILLEKLFAEKDKNSAREIELFIKYIEARPLQLRACNLVPLDFNLPVIVLNLCITYLIVIIQFTHLF

>BmorGR17

MGFSLGTTALSMFFFEKPVVFTIIQITMIIVKPAKYKLSDPFRPKDTSKLSESIIMYFKLFHIFLGIDLGGFRYQNRQVKYAVRLISLIQPLAIYGLCIYALLKIIANTEFLWYTISFTEYVAMSVAITLFSNEMTYCNFMINLKFIDTKLKIGDESFRIGVKLISSTILIGVTRCFTTTTYCLLGFCAKPTAAQILFQIPWLTIDLMLLQYMFIFYACYCRLVKILRILKKRNTDIEEMRRIYKTLVDVLDRARAPFDLAYLLGLLFSIPDVLYSIYESIIKVGEINTAKALSMSIIYITNIQSLALMFAPALTAGFLPSLTMKMRIILHDKLLEEQDKKTYRHIVLFIKYIETCPLKLKACQIIPLDFSFPIIILNIVVTYLIVAIQLTHFL

>BmorGR18

MRRSTKVISMVNQSDKGEIKTCSRFMKIYFFVIYILTGFNFGFYTGRGLNFLRVIQASVLLLRFIIASNCIYIAFHFRLLEAIWYSLTFSESLAIVVCFMLSRSALSCKNLFEYLYSVDQELKKSVGPSIEVKLALYTVVVSVLRLTVYVFCAIAYYETLHEGFCVELVYNTPCYCSDLYLVIHFTIFHSVYCRLKALRISMNEKFDVYKGTLIYKSLIDNLEEIKKSLDVPFFVILLNAVAIAMINILVTLEISYGQTMKFIRTAPRYLETVLLFSSAFAPVLAADMMASEAQKIKVTLNNILQRDDSLLEDDRRKVKQFAGYVSARPFRLRACRVLSLDCTLPVTVLSICVTYLIVVVQFTHLY

>BmorGR26

MNKTKIYRKKLDKNERLVCSVQPAMFARLIVGLYYDIKVSNRVKWMIKSYCISLSSFICYLIIFRDDNFSLHPKLTSVMEYITYVTFSFLTCDKYLFRYLRFNPRTDGYPIFLYLCKKFEKFFKIIICLFVSFKILGVVLMMQSWPILSTPKYIWGTLALHFLWLASHMGRLVFILVYGILFCRMRTIRIIFENRGFQNTPQNRLTPKRYILMYEAVLNSIESVDFPVKFLIFTFICCFAPKLVVSLFEIMEEMKKGELSLTTFIWFLVELSPSYLFLLLSAIALDLVSEDVQELLSITIDRRLNCKNEKERSEIQEFFQYLRNNPFNYTLWQVVSLNLRTLLVATSFSIANVIAIMQIKNSKI

>BmorGR27

MVFKYKIMTKAPKSLPVLKILMLFRLVFGNYFRLSSNRYINFLVKSYCSTFTILLSVMCGKRLKNDSPYMLSLTEYILNKILNYATSEGYIFKYCNSIKTCDKIMGFKKLPIITIDVFIAIIITVITRTAITIYFGFLFPFDKYQVVLYVGCIVFSNDLNSLTIMNVFGLLNNRMNLLRKSLEAMTVPINIIGKNEVAPKVRLVRNAFRYYSNLLDNLDSVNHCVQYSLSVTLLLKFPKAVLLCYDSIKTYFVKIDNNFAMDIVDPTEIILSIVVMSFPAMLCEMITNEVEKIKAILTKHLIQCSDNSLRFELNITLLYICHRPFKYILWRAIPLDTSVPIGIVSLIITYVIVLIQLLHFST

>BmorGR29

MYLRSKKSRFKLFSFERMIKILLMICGHYVQTDSSNVVSSIHRIFSIVITICLCPYFQFNPFFFHVIESVWYSILSQFTQYGFFFRYCSTIKTFDLLSGFKQIPLYTKRVCFFLLITLLVRLIIVLIHFSAHQTKLKTFCAFLIILSANTGHILMTIMFSILNTRMTLIQKLFANNPIPVNIVGKNQNASHIKRVRKGLICYNNLLDTLKVAEKEIQFTLTVTYLCHVPTIICYVYFVITVIYKSKFSGYNLIPMLDMILACMAVTAPALFAELTKNTVDKIKKILGSQLLRCSDESLRYELEITLEYVIQRPFSFSIWRAVSLDASLPVAMTSLCITYVIVILQLTQLRP

>BmorGR30

MYLRSKKSRFKLFSFERMIKILLMICGHYVQTDSSNVVSSIHRIFSIVITICLCPYFQFNPFFFHVIESVLYSILSQFTQYGFFFRYCSTIKTFDLLSGFKQIPLYTKRVCFFLLITLLVRLIIVLIHFSAHQTKLKTFCAFLIILSANTGHILMTIMFSILNTRMTLIQKLFANNPIPVNIVGKNQNASHIKRVRKGLICYNNLLDTLKVAEKEIQFTLTVTYLCHVPKIICYVYFVITVIYKSKFSGYNLVPLFDMILACMAVTAPAVFAELTKNTVDKIKKILGSQLLRCSDESLRYELEITLEYVIQRPFSFSIWRAVSLDASLPVAMTSLCITYVIVILQLTQLRP

>BmorGR45

MKSPEYLSKDILDEDFVRVFSFPFLVQMALGSCRVHLKARFITVPTLGQKLYTVMCIIICSLMYFNMTKLYLPLYYEHSIVYYIFVTVTGLDQLSFFANLIHLRFLNGETNTAFYIMMQRIDRNMKIDHNNIFNKTVTLANILTITLIILHYVGLVISTIILKEYSLLSLFGLLYGQLMLMVEMALCSNLIIFFFMRVRFVNAIIKNHVHPENQNQPPKLVRYFITNRITRYLAAQTHDFIVNDTDVYLKQIFEGFSMFIDIYRFQVCPLCIKLVVLTLLNFEFCLVAIQRNVLGPNHIGNYYIIVNSVMGFFTALYVSGRCELFFREIRETKRLSVAVLLQYQEGPLREKATRMLKIIEESTPQFSIYDMWQMDGYTFVKICSLVTNLIVTLLQFAYL

>BmorGR50

MAGIRTISSKVKPLELPDVSENNFADDGLKIVQPFKFFIYIQAITGINRLYLLKCNKFVLMFSYLYAIFLISFVALVYWTTEPKKNSHLVIRLFTFFEYTLLACISVFLKKKKMIKFFENLSLLDKMLKINKNVNSTCCMKQVFFWVTGSIVYNLIEFYAMEFYDNTNKGLKTIICTYAIALAHDCEQIFFFTLQRVVYLRLLVVKRHIQEYFKVDEDSSRKKPNKYEMLSNNVQLNLTALHEVYALLHNCAEKLNTVMSIPVLLMLFTSGLSTTILLKFFVRVIQLTDPSNPGSAIGVCMYLIVRCIKYTLLVVISCYYSSITATQVSLIRITIHDAINTVPLGKLQRRKVKAFYLMTKEYSFVYALAGVIKLNMSLPLSYISLCTTYLVIIIQFSKFLD

>BmorGR51

MAMGIRTILSKVKPLELPDVSENNFADDGLKIVQRFKFFIYIQVLTGINRLYLLKCNKFVMLFSYLYAIFLISFVASVYWTKEPMKNSHLVIRLFSFIEYILLICISVFLKKKKMMKFFENLSMFDQILKIDKNVNSTFCMKRVFFWVTGSIVYNLIEFYALEFYDNTSKGLMTIICTYTIALTHDCEQIFFFTLQRVVYLRLLVVKRHIQEHFKVDEDSNRKKPNKYEMLSKNVQLNLTALHEVYGLLHNCAEKLNKIMSIPVLLMLFTSGLTTTILLRILVRVIQLADPSNPGSAIGLCVYLIVRCIKYTLLVVISCYYSSITATQVSLIRITINDAINTIAFGKLQRRKVKAFYLMTKEYSFVYTLAGVIKLNMSLPLSYISLCTTYLVIIIQFSKFFD

>BmorGR53

MAHIKDENQSKQQQKEHETLNKNKLKKVVYTLKPALMLENWFGLSDFLLVNEDELVLLMQTEKFGVILSIFFIVMFAVFVDFPDTETESIMELMDEVPSMVVLSQYFIASITTSSCLSAIAIRIFETFADLDSMLLITTTQDFYNKSRYQTNKYLIILGVSHIISSTLDLLTDDEIVWCKFFVLPIYFLQKLEVLTFCKLIVMIQCRLQIINKYLTNFIEEQEKNKALVFTLAESNPKKTDKFNWIGCPSPNNMKIRDLATMYDVIGTICSLINDLFNIQIFMTLVSTFTYIVIAIWSTLYFYRAPNFTFGTLTTIIIWCITIILSVVVMSFVCERLVSVRNNTKILVNKVIMNYDLPKTMRVQAKAFMELIESWPLKIMVYDMFSVDISLMLKFISVATTYLIVIIQLSHFV

>BmorGR63

MQIGNAVIHLKSTKLTTMNTISPTTKLLKIFALNSNIEEIDLKCSTKLRITMTAFVLCSLIFYSLYYKFIYVFDYVNISIKITDCVQMVYDFCQYIVDLYFVTNYGRNISSEYFQQYKIIDKILEVVCYEIIKHRIVKLLWVFMCIWFSSSCFDFIAWFLNYGWITPLVYSVAYIFLLIKILTTLDLSAHIMNVEIRLKMIADLIHHYYMSCEDNFQAEETLCHKNWLNSKERAKYYELQFRIHALKQLSCNNNEIKLLSRCYLMLTEQVEIINRMYGFRILLNSLSLLIDMVRFTNISVRIMIGSQNLAYNCGYFPAVSSIFRLLTCGAVIINLVSHCERVYYQRTRICNVIDHMIVNKNLSRESTEALQEFRNLVQNHPIEFNMANFFQLNYSLLVSIASVVVTYTIILLQSVN
