# Supplementary material for: Identification of chemosensory genes from the antennal transcriptome of Indian meal moth Plodia interpunctella
Source: PLoS One. 2018 Jan 5;13(1):e0189889. doi: 10.1371/journal.pone.0189889 (PMC5755773; doi:10.1371/journal.pone.0189889)
Supplement: S6 Table — (DOC) [file pone.0189889.s006.doc]

>OfurIR21a

MTRLKCLIANIFIFVSFVLCEDVEYYPSQAALNSYSNIAKRSVNEPELEKGKVAIKWRHFNENKNETQEVKTKRAVDPIFHGHPKTREELWNERFLNKSSAFDQTPSLIKLIHNITLRYLNDCIPVILYDSQIKTRESYLFQNLLKDFPVSYVHGYIDDNNKLKEPELLIPVKQCLHFIVFLTEVKSSAKVLGKQSESKVVVVARSSQWAVQEFLASSYSRVFINLLVIGQSFKDDDDNSLEAPYILYTHKLYTDGLGASQPKVLSSWTHGKYSRDVNLFPPKMTEGYAGHRFIVAASNQPPFVFRKIKTDLDGGNPRVIWDGIEMRLLHLLAERNNFSIEILEPQEPHLGSGDAVTKEIAMGRADIGVAGMYLTVDRTKSMDMSFSHSQDCAVFITLMSTALPRYRAILGPFHWHVWVALTFTYLIGILPLAFSDKHTLRHLLHNSGEIENMFWYVFGTFTNCFTFVGKNSWSKTTKITTRLLIGWYWIFTIIITSCYTGSIIAFVTLPVFPETVDTIEQLIAGFYRVGTLDRGGWERWFFNSSDAKTNKLFKKLELVPNVESGIRNTTKAFFWPYAFLGSQAELEYIVQSNFTATKSKRAMLHISNECFVPFGVSMGFPTNSLYSAKLSGDLRRMFQSGIVDKIVDEVRWEMQRSATGKLLSAGSGSLKITSAEEKGLTLDDTQGMFLLLAAGFLMGASALVSEWMGGITRRCRIGRKKPSSANSKEELIATPELESEIKVISDCTESRLNFDTRCSSACSRDTLEGQVINVTEENIVVHETLDAATWDSRRSSSVDLDREVQEIFEKDLRRRRIVTGDIEEAAEVKRELTASNGAFGDHLN

>OfurIR25a

MKPKESRFSLKLLLLFSFVRVAIFQTTQNINVLLINEENNALAEKAFEVAKEYVRRNPSLGLAVDPVIVVGNRSDAKVFLENVCRKYNDMLSAKKTPHVVLDFTMTGVGSETIKSFTAALALPTMSSSFGQAGDLRQWRSLDANQTRFLLQVMPPADILPESIRAIVTKQDITNAAIIFDELFVMDHKYKSLLQNIPTRHVITPVKSFNKDEIKTQLRSLRELDIVNFFVVGSLRTIKNVLDAADENQYFGRKTAWFALSLDKGDITCGCKDATIVYMRPTPDAKSRDRLGKIKTTYSMNGEPEITSAFYFDLSLRTFLAVKSLLDSGKWPNDMKYITCDDYDGKNTPNRTLDLKAAFQEIKETPTYAPFYIPEDDPMNGRSYMEFNTDITAVTVKDGASIGSRVLGSWKAGLSNPLSLTDPDNMSDYSAQLVYRVVTVEQEPFIIRDDEAPKGFKGYCIDLIEEIRQIVKFDYEIVLSPDGNFGTMDENGNWNGIIKELIDKRADIGLTSLSVMAERENVVDFTVPYYDLVGITIMMKLPRTATSLFKFLTVLENDVWL

SILAAYFFTSFLMWVFDKWSPYSYQNNREKYKDDEEKREFTLKECLWFCMTSLTPQGGGEAPKNLSGRLLAATWWLFGFIIIASYTANLAAFLTVSRLDTPIESLDDLSKQYKIQYAPLNGSAAMTYFERMAHIEVKFYEIWKEMSLNDSLSDVERAKLAVWDYPVSDKYSKMWQAMKEAGLPNSIEEALQRVRDSKSSSEGFAWLGDATDVRYHVLTSCDLQMVGDEFSRKPYAIAVQQGSPLKDQFNNAILQLLNKRKLEKLKENWWTNNPNAMKCEKQDDQSDGISIQNIGGVFIVIFMGIGLACITLGVEYWWYKIRKRSTIGDITQVEPAKSSRINTDFKGEGFTFRSRNLGLSNLKPKF

>OfurIR40a

MKFVPFFLFLNTAHCFFDIQDIISQTMTKLPKDFAVAIKDIAEGLPAKTITVVRGESTKIRSQDIFQLLCLLSEHNIQVINLDITTKQNKDKYYSFVKQALDISEDRTSLILCEPFECEKILTELTDNNLIHRTILYIFYWPYGTVSDKFLNTMKEAMRVAVLTNPRESVFRVYYNQATPDRLHHLSLVNWWSGRLYKSPVLPPAGKIYQDFKGRMFDVPVLHAPPWHFVRYNNDSSVNVTGGRDDKLLSLISKKLNFRYQYYDPPDRSQGSSISGNGTFKGTLGLLWKRKADFFIGDVTMTWERLQAVEFSFLTLADSGAFLTHAPAKLSETLAIIRPFRWEVWPLVCATVLVTGPALWVVIAAPSLWQRRQRDQLRLLNNCCWFTTTLFLRQSSSKEPSKTHKARLVSVLVSLGATYVIGDMYSANLTSLLARPARERPIGTLQALEEAMRDRGYELVVERHSSSLTILENGTGVYGRLARLMRRQRVQRVRSVEVGVRLVLTRRHVAILGGRETLYYDTERFGSHNFHLSEKLYTRYSAIALQIGCPYLETFNNVVM

TLFEAGILAKMTTDEYKNLPEQSRRSEPVTESDKPNNDITGDSPAASQGGTTPGESTKALEPVSLRMLRGAFCLLGIGHLLAAIALGVEIQIHRRSKKFIKIVEPNGGKNVPGKRALRKANKFIRQGIGRMVRAFCRSVDRALGPGNQ

>OfurIR8a

MMEIPLLLLFLINLGCVLSEISLRFVFITEVHDSDLAHQIGRALRNAEEQRSGVKISDYMVQLDRENEDESYRRLCSGVSKGASLVIDLSWAPWDMAEQLCAESGLPLVRTLLGSQQLVAALDEYLESRNATDAAILLESESDVDKTLYELLGRSNVRLWVHAGLTRDSAKALKSMRPEPSFFVIVGESGFLMDTYRRAVKEKLVRRDYRWNLVLTDYSGDSIDVTQLPLPTMILHVDQVECCRLLGLREECSCPSDLKRKQLIISALVLYLSETYSKLERELPVLSTKVDCDNVLASEMNVTRDRLVRQFGEDVEINNDTLFYWDDDRSGLFLRSSFVLSVYRPDSGLETVASWSANDEYKLLPGVTLDPLKLFFRIGTSPAVPWTLPKLDPETGEPEVNEDGQPVYEGYCIDLISKLAETMEFDYEIITPKSGSFGKKLPNGSWDGVVGDLMRGETDLAVAALTMTAEREEVIDFVAPYFEQTGILIAIRKPIRKTSLFKFMTVLRTEVWLSIVAALVLTGLMIWLLDKYSPYSARNNPQAHPYPCREFTLKESFWFA

LTSFTPQGGGEAPKALSGRTLVAAYWLFVVLMLATFTANLAAFLTVERMQTPVSSLEQLARQSRINYTVVEGSTIHQYFINMKFAEDTLYRVWKEITLNATSDQAQYRVWDYPIREQYGHILLAINASGPVPDAKTGFEQVNEHTDADFAFIHDSAEIKYEVTRNCNLTEVGEVFAEQPYAIAVQQGSRLQEHLSRALLDLQKERFLEQLASKYWNESARQACPDADESEGITLESLGGVFIATLFGLGLAMITLAWEVFYYKRKERNKIQGIDAKVEKAAFVDPKKKDKLGVRLRKGKSKVAKLDVVGKGKGVTIGDTFKPAAEKMGVSYISVYPKGEYRP

>OfurIR41a

MLQDTVLFFPVEILLTSIVNLYLNSSYCLTIVSEKSLDLSISNSFTSMVPEDGDLLVNQLLQVSEMGCSDYIVKMRDPARFMAAFERVNHLGNVRRSDRKVVFLPYEDNNITRTDLLQLLTLKETSFLANILLILPSLESGLCSIYDLATHKYTGPDDQVDQPYYMDRWNSCSLKFEKDANLFPHDMTNLHGKTVKVACFTYKPYALLDLDPLEEPLGRDGTEVRIVDEFCRWINCTIEVVRDDEHEWGELYDNQTGVGVLGNVVKDRADLGITALYSWYEEYLELDFSGSGIRTAITCVAPSPRLLASWEMPLLPFSWYMWMALGFTFVYASFALAIAKGCSTDKVFLATFGMMVTQSQADVGATWRVRSITGWMLLTGLVLDNAYGGGLASVFTVPKYEKSIDTVQDIVDRGMEWGATHDAWVFSLTLSPEPLVKQLVSLFRVSSAEDMKIKSMQRSMAFSVERLPAGYFAVGDYITKDAMLGLTLMQEDFYYEQCVVMMRKSSPYTQKVSKLIGRLHESGLMLAWETQVALKYLNYEVQLEVRLSRFHKDVDNVEPLKLRHVVGVFIIYIIGVIISTMLFILEIIHKHKKRVY

>OfurIR64a

MDINYFLNFISIAEISLVIDLLKLKEIQNVVNINCDGQKSIFHHKILNDNNIHASYWSLNSTDQNMMQMSYHKTGVILDASCSNWEQALNNFDNSMFRNEFIWLIITEDLLSTARSLTNCPIEIDSDVTVALKTNGIFMLYEVFHTNYSSGVLSIRNVGYWDTTLHIATSSRRDLQGLKMRCPVVVTDKVVHQTFEEYLSKHQVFQVDSLHKLKFVALLNYIRDMYNMSYELQRTNSWGYMRNGSFDGVVGSLQRQHADFGGSPLFFRADRAELIDYIAETWQSRQCFILRHPKHPGGYYTIYTRPLTAKVWYCILAMLIFSGVILCLMLKTKVTQSHEKSTDSSFSLALLFAWSAICQQGMTVNRSSTSVKIVVIVTFVYAVTLYQYYNATVVSTLLREPPKNIRTLEDLLQSNLKAGAENVLYTKDYFKRTTDPVALRMYHKKITPKHQYNFYSPEYGMSLVKQGGFAFHVDSVVAYRIMRKTFTEREICEAHEVLLYPPQKMGMVVRKASPYKEHFTYGIRKIYEAGLMDRLQSVWDEPKPSCVHTPDSSVFSVSIVEFSTALLALVAGNVAAILVLFAEIVLHRCEMKKRIAFTH

>OfurIR68a

MWIAVLVVLLISGSIFYGLARHYMNLQEYIKTHENRNTNEKQLDTAEKPVGLYLFGEIINSILYTYAMLLVVSLPKLPTGWSIRLLTGWYWLYCVLLVVSYRASMTAILANPAPRVTIDTLKELVESKIACGGWGMETKKFFQESSDDIQTIGQRFETINDPFVAANKVAKGVYAYYDNENFLKYIRVKRKNIDMNIQSDMVNATSNTTDVFAGEMERNLHIMSDCVVNTPISIGFHKNSPLKPLADIYLRRIVEVGLVEKWLNDAMHPIKSLETNEDEVKALMNLKKLYGAFIALAIGYSISTIGLIGELIHWHLIVKRDPKFDKYAIDLYYLSKNKKQ

>OfurIR75

MNLRDLLLVFILVYFVCTFSKSSDEISIITDLIQSSDKLTSVVAHACWKPSKQIQLASRLGNKNRPMTVRFVNKNWAGIVEPQHRERLLIVADLNCPSTKVFFKLANTTNKFSFPYRWIVIGKAVNESVAVTSNFQNIPLLPDSDVIIAQKNDKNSYILTTIYKIQIKGKWIVEQFGIWTSANGLKKFEAVKHPISTRRKNFQRAPIKMAMVILDNRTISNPYDLSDILTDTVSKSSFRQTDPIFGYLNASRTLIYSPTWGYYRNGSYGGMIADMTIGDAELAGTVLIATQDRMEVVEYLSCPTPISIKFVFRQPPLSYQNNLFLLPFKSTVWYCIGAFVLVLAFILYINALWENKKLESSEQNFEDPTVLRPNVGDIAILVISAISQQGSSTELKGTLGRIVMFILFLAFLLLYSSYSASIVALLQSSSNQIRTLSDLLNSKLELGVEDTPYNRYFFPIATEPVRRAIYQTKIAPKGTKPKFMSLEDGVKKLQKEPFAFNMNKGIGYRLVERYFHEHEKCGLQEIPYLYATKTYITCRKNSPYKEIFKIGLFRIQEHGLSDRENRLIYARKPPCQARGGSFGSVNMVDFHPILLMYLYGILLAFFFFFVEILAHKKLHPRQSQRR

>OfurIR87a

MCTIIFLPLFFALHVSATINENSLLTTTGNSEQTAKTAECVLKLSAKYFVEKKALSGSIVIININSYASTTQVLLLQTIHGGIKYSVMVKDSFYPHANASHFPEKAKNYMLILEEKSELTRNILQLNKLPTWNPLAKAIVFYQLNKTEDAEQTAIEFINELRHYKLFKSIIFIYSPEEKEVISYTWTPYSDTNCGGKCDSVYILDTCKDNVIHELATQKEMFPLDMKGCPLVTYAIVSEPYVLPPAMKLSNTSYNDAYVFQKGGEINLVKIITQFTNMSLVMRTSDVPENWGNVYWNGTATGAYGVLRNDEVDMVIGNIEVTRTIRRWFHPTVSYTQDEMTWCVPKAGQASTWNNLVIIFQWSTWVATFGSLFVMGLLFHYMYYRENNQKVTKWPTNSLLMTFSMLLGWGASFEPKSATFRILIFGWLCFSVNMGISYESFLRSFLMHPRFEKQISTETDLIQSRIPLGGREIYRSYFETNNASSFYLYRKYNSTTFAEGIKRAAKDRNFAVVSSRRQAAYADQRLGKGKPLIYCFPESDNLYKYGVVLLARKWFPMLER

FNTIIRSVSENGLIDKWNQELLIHTANAEGASEIEPLSIQHLLGAFMFIGFMYAASIAVFIAEICIGVFQKWKARKNWQSLYKHIRFRNAK

>OfurIR93a

MRICLFVSLYLLRVSGEEFPSLITANASIAVVLDRQFLGEQYQATLDELKDYIKELARVELKHGGVVVHYFSWTTISLKKGFLAVFSVASCEDTWSLFSRTEEEELLLFALTEVDCPRLPTDSAITVTNVMPGEELPQILLDMRTEMAFKWKSAVILHDDTLSRDMVSRVVQSLTVQIDEGASTSPVSVSVYKMKHEINEYLRRKEITRVLSKLPVKYIGENFMAIVTTEVMTTMAEIARDLVMSHTLAQWLYVISDTDAQNGNLSSLINALYEGENVAFMYNITESNPECKNGLMCYCQEMMNAFISALDAAVQDEFDVAAQVSDEEWEAIRPNKIQRRGMLLKHMQQHISTKSSCGNCSTWRALAADTWGATYRSYGDSDQLFKEPDNATTKGVIEHVDLLQVGYWRPIDALRFEDVLFPHVEHGFRGKALPIITYHNPPWTILQVNESGSVVSCSGLIFDIVNQLAKNKNFTVKVILPSHVKNLLSNDTTADMMHSQDAALTLIAVAKGQAAIAAVAFTVLSDPPSGINYTLAVSTQPYSFMIARPRELSRALLFLL

PFTTDTWLCLGLGVILMGPTLYIIHRLSPYYEAKEITRQGGLSTIHNCLWYVYGALLQQGGMYLPRADSGRLVVGTWWLVVLVVVTTYSGNLVAFLTFPKQEIPVTTIGELLENQLTYTWSIQKGSYLEMELKNSDEPKYAALLKGAELSGAGASGNLSSWKKQLIRIREQRHVIFDWKLRLSYLMRAEHMLTDTCDFALSAEEFMDEQLAMVLPAGSPYLPVINKEINRMQKAGLISKWLFAYLPKRDRCWKTSSIAQEVNNHTVNLRDMQGSFFVLFLGFFSASVVLLLEWFCNRRRRRSEDVIIKPYVE

>OfurIR1

AAATSLYHKHQDITPILRWENVIDKVDLVHPPVTSIETRYFYRIPTYGAGKFENQFLRPLSYGAWISVVIVITLCACVLLISAKLERRRSAGQYAIFSVLASMCQQFFEDNTSFTPRVSAARQLTIFVTGISCVLIYNYYTSSVVSWLLNGPPPSINSLQELLESPLELIFEDIGYTRSWLQKKSYYYNIRNIEIEDELRKKKVFNKKPNAPLLVPVEEGIKLVKAGGYAYHTDTNNANRLISQTFTQSELCELGSLQSMAKAELYPALQRNSPYKEFFVWGSIRLYERGVVKFVQRRISSPAVECEGSSPRALALGGAAPAFLLLAAGYLLATVIMLVERAIWRRKYKSKVVLKLKK

>OfurIR2

RVLVPAKLWYSTQDVLRSESADLNAGVLRIMETRMEYLDYIMPIWLFSVGFTYLAERESSSNMYVEPFTAACWWTCLGIGVTLALAQRVAARGKQEKEGAFMAVLATWLQQDAAAVPDGVAGRWTFTVMSICAMLVHAYYTSAIVSALMSTGRGGPNTLRELADSRYAIASEDHDFIRNGMFNVETDWEELEYLKKKKMTSKLFQDMEYGVQLIQQGYTAYHAEYHQLYHFLEKFSDDEICKMQHVDTIPEIMTWVSASARGQWTEIFRSTGAWLYETGLARQLLSSIQTHPPPCRAAMLAERVTFWDVAPLLGLTTIGAIASIGLLGLEIVLHRWTEKNRREKKWGSSEASLVALE

>CpunIR75p

MNQEFSEEDEDREGDAVGHEDDDWPDVQQTHAAEGGAVGDAVRWWYGETADHRALDA

>CpunIR8a

MEIPLFLLIFFINLGCVVSELSLRFVFIIENHEPELGQLVGRALKVAEEQQDVRVDDSIVLLDRENEEESYGRFCSAISKGVSLIVDLSWSPWELAESVASGGGVPLVRTALSMQRLLSAVASHCASRNATDAALITESEADVDRALYELLGRSNIRLWVHAGLTRDSARALKNMRPDPSFYIIVGESGFVMDTYRRAVKEKLVRRDFRWYLLLTDYSGDSFDTSQLVLPTMMLHVDANECCKLLGSKDDCSCPSDLKRKQYILSGLMTYLAETYSKLENDLSVVTAKMDCDNIQASEMNVTRERVVRQFADDEQISNDTLFYWDGERSALYLRSTFVLSTFKPDSGLETVASWSANEEYKLLPGVTLEPLRPFFRVGTSPAVPWTLPKLDPDTGEQVYNEDGQPEYEGYCIDLIARIAETMEFDYEIITPKSGTFGKKLPNGSWDGVVGDLMRGETDLAVAALTMTAEREEVIDFVAPYFEQTGILIAIRKPIRKTSLFKFMTVLRTEVWLSIVAALVLTGLMIWLLDKYSPYSARNNPTAYPYPCREFTLKESFWFALTSFTPQGGGEAPKALSGRTLVAAYWLFVVLMLATFTANLAAFLTVERMQTPVSSLEQLARQSRINYTVVEGSTIHQYFINMKFAEDTLYRVWKEITLNATSDQAQYRVWDYPIREQYGHILLAINASGPVPDAKTGFEQVNEHTDADFAFIHDSAEIKYEVTRNCNLTEVGEVFAEQPYALAVQQGSRLQEQLSRALLDLQKERFLEQLTSKYWNESARQACPDADESEGITLESLGGVFIATLFGLGLAMITLAWEVFYYKRKEKNKVQTIDAKMEKAAFTEPKNAEKTGVRFRKKEKKSKLGKISKLGKVEEGKLGKRVTIGDSFKPASEGAGVSYISVFPKGEYRP

>CpunIR1

MTYVIMGIGVAISIITFLGELLIRRFTLTYDQSYKPKRKKTKFKKHPHIYTYNQSRPPSYDSIFGRSSKIKVNDTVKRK

>CpunIR41a

MINILRIIFTIQIDWVNKFILFFKYKNFISYLFTMMLDSVLSLMPIEILLQTIFNEYLSNSYCLTVVSEKPLDLHVNISYAYISVENGELSPDQMLKLSENGCSDYIVQVKNPQKFMGAFETVNLLGNVRRGDRKIVFLPYREDNATTTLLLEILTLKETSFIANILLILPSPEQSTCSYYDLVTHKYVGQDNEINQPYYIDRWNACTLNFEKNVSLFPHDMTNLYGKTLKVACFTYMPYVLLDLSEAQEPFGRTGTEIKIVDEFCRWVNCTVELVREDEHMWGEIYDNLTGVGVIGNLVEDRADIGITALYSWYEEYVVLDFSAPGVRTAVTCIAPSPRLLASWEEPLLPFSWYMWLALIFTFVYASLALTIAQGFTTDNAFLTTFGIMIAQSQHDVGASWRVRSVTGWMLLTGLVIGNAYGGGLASVFTVPKYEKSIDTVQDIVDRKMEWGATHDAWVFSLTSSNEPLIKKLVNQFKVYPADVLKKKSLDRSMAFSIERLPSGYYAIGDYITKEAMLDLTVMLEDFYFEQCVAMLRKSSPYTKKISQLIGRLHESGLLLVWETQMALKYLNYEVQLEVRLSRSQKDINTEALSLRHVVGVFILYLIGMIFSVIIFTLEVMNVNKKRNTSSF

>CpunIR2

MLNKMQDMKTYEVLFILLMSCFSLIIANPINEFRMIADVIKDSNKSTSVVAHLCWNPSKQIQMASYLHNSELTQLVLLVNESWADIKEPQHRERLLLIADIDCPSTTAFFKMANETKKFSLPYRWLIIGKAVNKSTDVTADFDGLHLLPDSDVIIAQKNDSNSFYMNMIYKIKIKSKWIIEDFGTWTTNTGLIKSDLAQYSTSTRRKNFHGESFTTAMVIFDNKTISNLFDLSDILTDVVTKSSFRQIVPLYGYMNASQQHIYSKTWGYYRNGTFDGMIAELTVGDADLGGTVLIVTWDRMQVVDYLSKPGSITVKFVFREPPLSYQNNLYLLPFKVTVWYCMGAFVLVMGFILYITALWENKKMGENQEISNDPTVLKPNVSDIAILIISAVSQQGTTLELKGTLGRIVMIIQFIAFLLLYASYSASIVALLQSSSNQIRTFSDLLNSKLELGIEDTPYNRYFFPIAVEQVKKEIYKSKTPPRWTEPKFMSLEDGVKKLQKKPFAFNMLQGIGYKLVERYFHEHEKCGLQEIELQYGTKTYIASRKNSPYKEIFKIGLFRIQEHGISDREFRLLYARKPTCQVRGGNFDSVNMVDFHPVLLMYLYGILLAIALLVIEILVFKKQQLMCSAASRRQRSGSC

>CpunIR3

MKATVLLLFCLKYLNVKSHANTNSVMHMVGDIIRAMEKPSSVVATLCWLTDEKVQFYYAVTASDRFSRVNTAQFVDMRHVSEDHGQEQHIVFVADLSCPNISAYFDEKRAQNYFRGPFRWILIGNVVEEDIVPNSIAHIDALPDSQVIVARQIDEESYDLYTIYKINANDDWRTKLYGKWNQQTRFTITNPHMESIALERLDLLGSEISVCYVLTDKDSINHLTDEVNDHIDTITKVNFPTTNHLLDIVNASRKYIFADTWGYRVNGTWNGMTGYLIREEVEIGGSPMFFTSERISVVDYIASPTPTRSKFVFQQPKLSYENNLFLLSFRTSVWYSSTGLIFLLLLALFVVAAWEWKKHTNDNQVSFQRENDAGTLRPNFVDVIVLIFGAICQQGSPVELKGSLGRVVMLILFLALMFLYTSYSANIVALLQSSSTKIRNLDDLLHSRLKFGVHDTVFNRYYFSTATEPVRKAIYEKKVAPPGTTPRFISMEEGVKKMRKGLFAFHMETGVGYKFVGKYFDEGEKCGLQEIQYLQVIDPWLAVRKHTPYKEMFKIGMKRIQEHGLQSRENLLLYEKRPKCSGRESNFVSVSMVDCYPALLILSYGILVALFFLAFELLIHKRQTIVHRLSHCRRNSIDSRFI

>CpunIR21a

MSHSMIHLRCIVAYTLLLYYTVWCEDVEYYPSQYTIDNHNIAKRSSNYLQSTDNKWNIKEKVTQMKLRYFNDNDIKAKDNKTKRAVDPVFHGHPKTREQLWHEHFLNKSTAFDQNPSLIKLIHKITLKYLNDCIPVILYDSQVKSKESYLFQNLLKDFPVSYVHGYIDDSNNLKEPELLVPVKQCLHYIIFSTEVKSSAKVLGKQSESKVVVVARSSQWAVQEFLASPESRMFINLLVIGQSFKDDDDETMEAPYILYTHKLYTDGLGASKPVVLTSWTHGKYSREVNLFPAKMTEGYAGHRFIVSASNQPPFVFRRIKSDLDGGNPRVVWDGVELRLLSMLAERNNFSIEIKEPQEPSLGPGDAVSKEVAMGRADIGVAGMYFTSERTYGLDMSFSHSQDCAVFITLMSTALPRYRAILGPFHWHVWVALTFTYLIGILPLAFSDKHTLRHLLHNSGEIENMFWYVFGTFTNCFTFLGKNSWSKTTKITTRLLIGWYWIFTIIITSCYTGSIIAFVTLPVFPETVDTIQQLLAGFYRVGTLDRGGWERWFFNSSDPNTNKLFKKLELVPNVEAGIRNTTKAFFWPYAFLGSQAELEYIVQANFSMAKSKRAMLHISDECFVPFGVSMAFPSNSLYSSKLSGDLRRMFQSGLIYKIVDEVRWEMQRSSSGKLLSAGAGSLKIVSAEEKGLTLEDTQGMFLLLAAGFLLAASALISEWMGGIGRRCRQLRNKLPSSANSKEQLVISSPDLESEVNDGTESRLQFGTRSTSAGSRDTLDGQVINVTEENIIVHELMVEGLDSRRSSSVDLDREVQEIFERDLRRRKIVTGDSIEVSEEKREPTASKGAFGDPLS

>CpunIR4

MATGLELILSSICNATFCEPIFDNPLLGRQDSPKDVKYNDMVNEINGKHLKIATYDNRPMSWVEKGENGTIIGKGVAFVIVNILQKKYNFTYEVVVPEKNFEMGGDNPQDSLVGLANSSLVDMVAAFLPKVNKYREKVSFSYDLDEGVWMMMLKRPKESAAGSGLLAPFDNAVWYLILIAVLSFGPCITLLTRLRNKMVPDGEKFIPLSPSFWFVYGAFIKQGTNLAPEANTTRVLFTTWWIFIILLSAFYTANLTAFLTLSKFTLDIETPQDLYKKNYRWVSPEGSAVQYVVNSPNEDLYYLSRMIGTGRAEFRTVPNSQDYLPLVDGGAVLVREQIGIDELMYGDYLKKAREGVAEADRCTYVVAPNNFMTKLRGFAYPRDSKLQYFFDSILTYILQAGIIDFLEKKDLPSTKICPLDLQSKDRQLRNSDLMMTYMIMVTGLAAAVAVFIGELFIKRYICKTKDEVTKPKRKKTKFEKRLRIHTYDDSQPPPYDAIFGRNPKIKVTERAQRKIINGREYLVIDVSNGETRLIPVRTPSALLYQLDK

>PintIR1

MEIVYIFFFILLFNMACVASELSLRFVFIIENHEQDLVRQVGRGLKQAEETHPDIRITDEIVLLNREDDTESYSKLCAAVSRGVSLVIDLSWSPWPAADGLCSSAGLPLVRCQLGTQNLITALDDYLETRNATDAAFLLETESEVDKTLYELLGRSNIRVWVHAGLSRDSARSLKTMRPEPSFYVIVGSSSFVMDTYRRAVKEKLVRRDYRWNLVFTDYTTNVDVTQLVLPTVLLAVDQSECCELLARREECGCSEIQRSQHILAALLQYITETYNKLEHDLQQLPAKLDCGSAAGSELNGTRERLYRQLADDSDITNDTLFYWNADRSGVFLRSRFVLSTYSAESGRRSIATWRAGDQYRLLPGVELEPLKLFFRIGTSPAVPWTLPKLDPVTGEQMEDEDGRPLYEGYCVDLIAKLAETMNFDYQIVTPKTGGFGKKLPNGSWDGVVGDLMRGETDIAVAALTMTAEREEVIDFVAPYFEQTGILIAIRKPIRKTSLFKFMTVLRTEVWLSIVAALLLTGFMIWFLDKYSPYSARNNPDAYPYPCREFTLKESFWFALTSFTPQGGGEAPKALSGRTLVAAYWLFVVLMLATFTANLAAFLTVERMQTPVSSLEQLARQSRINYTVVEGSTVHQYFINMKFAEDTLYRVWKEITLNATSDQAQYRVWDYPIREQYGHILLAINASGPVPDAKTGFHQVNEHTDADFAFIHDSAEIKYEVSRNCNLTEVGEVFAEQPYAIGVQQGSRLQEALSRALLELQKERFLEQLTAKYWNESARQACPDADESEGITLESLGGVFIATLFGLGLAMITLAWEVFYYKRKEKNKVKSLENVEKPAFASEKIPEKKIDNFTKARKRGKTDKGKKMKKESKGVTIGDSFKPAADKISYISVFPKGDFRP

>PintIR2

MRFKLHFVFISIHLTTTTCEMTEYYPSQSLLNTKLKKLFKDNWNITEYDKSVNFFNNKAYGKIEWRHFGEQENVVKNITKRAIDPVFHGHPKTREELWNEHFLNKTKTFDQNPSLITLIHNITMTYLNDCIPVILYDDQIKSGDNYLFEDLLMDFPISYVHGYINQDNRLKEPRLLFGTEECLHFIVFLTDIMRSAKVLGKQSVCKVVIVARSSQWAVQEFLFSPLSRKFVNLLVIGQSFKDDDDETIEAPYILYTHKLYTDGLGASKPVVLTSWSHGKFSRHVNLFPMKMTEGYAGHRFVVAAAHQPPFVFRRIITDLDGGNPRIKWDGIEIRLLKLLAEKNNFSIEVIEPREPNLGSSDAVLKDIAKGRADIGIAGIYLTSERAIQVDISFSHSQDCAVFVTLMSTALPRYRAILGPFHWHVWVALTFTYLIGIFPLAFSDKHTLKHLLHNSGEVENMFWYVFGTFTNCFTFVGKNSWSKTTKVTTRLLIGRLFFIMFVYLCTKSAMIFKLIFILGWYWIFTIIITSCYTGSIIAFVTLPIFPETVDTIDQLLSGFYRVGTLGRGGWERWFLNSSDTKAKKLFKKLELVPNVESGIRNITKAFFWPYAFLGSQAELQYIVQSNFSKTSSKRALLHIADECFVPFGVSMVFPNNSLYSAKLSDDMRRVFQSGLMDKIVDEVRWDIQRSSTGKFLAVIPGFHITSAEEKGLTLEDTQGMFLLLAAGFLLAAAALISEWMGGISQRCRRKKPPSAKSQEHLIPSEIREVSNVDPRNSSAESRNTLDGEIINITEDDIMVHENFNTDVLESRRSSSVDLDKEVQEIFEKDMMRRNIIRGDTIEFDDDREPTASKENFGDRIKL

>PintIR3

MVFSTVIKLFLFYHLLHGCDAKLSPIIKDLHESKDLQLVLIDMLNGQARRHDVTCVVVICDTVYLNVFDGAMFKRILSVPMVMIVVEEYEDLLSPNFDTLESLREARMDGCNIYIILLANGLQVSRLLRFGDRYRILDTRAKYVMLHDYRLFHSDLHYIWKRIVNVIFLRYHRKITGVLKSKAWFDLSTVPFPNPIKTVFVSRRVDIWKNGRFHYNRTLFADKTSNLNGEALNVVYFDHAPSVVIMKMNDSSKVSGVEIEILNTLSQQMNFKPKLYQPNNIEVHKWGQKLANGTFSGLLGEMVNGKADVALGNLQYNPYHLELTDLSIPYTSQCWTFLTPEALTDNSWKTLILPFKLYMWIAVLLVLLITGIIFYGLAKYYMNLQELKKEKPFLESNKNVEKQEYVFEPSEKPVGLYLFGEIINSILYTYGMLLVVSLPKLPTGWSIRLLTGWYWLYCILLVVSYRASMTAILANPAPRVTIDTIKELVDSKVTCGGWGMETKKFFEKSTDETQKIGDRFELVDEPFEAANKIAKGVYAYYDNKDFLKYLSVKRKNSLINVDKAVNATINDTDIVSTDTERNLHIMTDCVVNIPISIGFHKNSPLKPLADIYLARIVEVGLVEKWLNDAMNPIRVQETNENEIKALMNLKKLYGAFIALAIGYILSLIGLIGELIHWHCVVKRDPQFDKYALDVYYAKNKKQ

>PintIR4

MFIKSRNTERKLYDVTSIPIEPKMNILYTFFSIIHLRTILGIDMITIDFIKQFVENEQAPTFLILCNICWRPDLQVTLMKNLTRSGCSSSSLDTKSKYHDHYTMFLLDAHCPGFDDVIASAISRKLFATPYRWLILDDRQHELLPDWPMYSDSDVVVAQRTEDGYKMTEIHKPSNSSSMVYYPRGYYNATTATLADTRPSRTLFRRRRDVMGAPITMSNVIQDSNSTQYHLPREDRLELQYDVVAKVCWVNVKIAFEMLNATPRYIFSHRWGYKQRGEWDGMINDLHTGNAELGTNCLVSDPQRLSVITYTDTLSSFRVRFIFRQPPLSYVSNIFSLPFSSSVWVAVAVCAAIATAAFYVTSKWEAKGGTSPSQLDGSMGDALLLTMSAVSQQGCVLEPRRVSGRIMLWIFFAALMALYAAYSANIVVLLQAPSKSIRTLAQLANSKLTIAANDVDYNHFVFKLYPDPVRVAISKRLEPEKGRGQFYDIKDGVERIRQGLFAFHSIVEPVYRQIEKTFLEYEKCDLVEVDYLNGFEPLVPVKKDSPYLELLRIVFKQIRESGIQSAVNRRMQIPKPHCSGQVAAFSSVGILDLKPVMLLMVYGTLLSVGILFVEILFGRLMKSDKLKHLKSKYLIKNTKVPLLKI

>PintIR5

MQLWVIYVSCFLLSVSGEEFPSLITANASIAVVLDRQYLGEQYQPILDDLKDYIKELARVELKHGGVVVHYFSWTAINLKKGFLAVFSIASCEDTWSLFSRTEEEELLLFALTEVDCPRLPLQSAITVTYMDQGQELPQILLDLRTTKAFKWKSAVILHDDTLNRDMVSRVVQSLTLQVEDNAVSSISVTVFKMRHEVNEYLRRKEMYRVLSKLPVKYIGENFVAIVTTEVMTTMIEVARDLGMTHTMAQWFYVISDTNSHSGNFSNLINALYEGENLAFMYNVTDNSPDCQNGILCYSQEMLNAFISALDMAVQDEFDVAAQVSDEEWEAIRPSKLQRREMLLKHMQQHISSKSRCGNCNTWRGLAADTWGATYRQFTDDQGSQPSAQEVKENHQSYINMTTSVIEQIELLQVGNWRPIDAMRYTDVLFPHVEQGFRGKELPIITFHNPPWSILQVNESGMVSSYAGLMFDIVEQLAKNKNFTIKILLPGNVKHDFSNDSSSDSMHSRSAMLAVSAIAKGQAALAASSFTVLPNPIPGINYTMAVSTQPYAFIIARPRELSRALLFLLPFTTDTWLCLGL

>PintIR6

MAAGVDLIISAVCNATFCEPIYDNPALETTHSKSRAVLNTLAKEVNGAHLKIATFNNFPMSWVERQENGTHVGRGVAFTIVDILREKFNFTYEIVVPEKNFEMGGKMPEESLVGMVNVSNVDMAAAFVPKVWQYQRKVDFSTDLDEGVWIMMLKRPKESAAGSGLLAPFTSHVWYLILVAVLAYGPCITLLTRLRSKIMPEDEQPIKLSPSFWFVYGAFIKQGTNLAPEANTTRVLFTTWWLFIILLSAFYTANLTAFLTLSKFTLDIEYPKDLYKKNYRWVAAEGSAVQYIVAAPNENLYYLNAMVKNGRAEFRSFQNHSEYLPMVKNGAVLVKERDAIEHTMYADYLQKAREGVAEADRCTYVVAPNKFMAKRRGFIYPRNSKLKPLFDSILWFINQAGLVNFLKSRDLPSTKICPLDLQSKDRQLRNSDLIMTYMIMIVGLCAASAIFIGEVVVKRYVRIKFRKPADKTKEKRKKTKFNKSSTVHYYYDSKPPPYDAIFGKSKPKLLEGRQLKVVNGREYLVVEFNGGSRLVPVRTPSAFLYNLEK

>PintIR7

NIFTISVWFKRQRKRNKGHHIEIMMKMVPTFLFLIFLTSSNADTDFQATMIADLVRSMERPSLVIATLCWPAYKKLKLYSMLDGWYQEWESFDLHLEKYAQMQSIVFLTDLNCPNVSNHLQKSLEKKYFRSPYRWLILKTTESNKENKIVPEAIYDFDIFPDSEVMVIFPVGDTSDIYFIYRVGAGEAWKTEFYGTWDVQNKVRKSSAMSLSTALRRQDLSGHEISICYVLTDSDSINHLTDEVNDYIDTITKVNFPTTNHLLDFLNANRKYLFANTWGYHVNGTWDGMTGYLVREEVEIGGSPMFFTSERMSVVEYIASPTPTRSKFVFQQPKLSYENNLFLLSFKSAVWYSCIALVFIMLLAIFVVAVWEWKKHADTFDSRNKDAGVLRPSIADIVILIFGATCQQGSPVELKGLLGRIVMLILFLALMFLYTSYSANIVALLQSSSSKIKSLEDLLNSRIKFGVHDTVFNRYYFATATEPIRKAIYEKKVAPPGSKPRFMSMEEGVKLMQKVGN

>PintIR8

MIHIAFVSPIEVLLQTIINQYLGISYCLTVVAETPINCMYPVSFTYIVPNDNLTGQMLDVSEKGCSDYIVRVREPNLFMVSFDEVNQLGNTRRSNRKIVFLPAENDEYDTNKLLDILSLKQTSFVPHLLMIVPSANISGDECQSYDLVTHNYVGSDDESSQLVNLDQWNSCTDKFHGNVNLFPHDMSNLYGKTFKIACFTYKPYTLLDIDSIEHEITGRDGTEMRVMDEFCKWVNCTVKIIRDDDHEWGEIYENRTGVGILGHVVEDRADAGISALYSWYEEYVQLDFSIPTVRTAITCVAPSPRLLASWEMPLMPFNLNMWIALIFTFIFACICLTIAKRCSSDRVGLTTFGMLITQPLIKQLVSLFRVMTAEDLKEKSFTRKLAYSIEKLPAGNFAIGDYITQEALIDLTVMQEDFYYEQSVVMLRKSSPYTEKISQFLGRLHASGLILAWETQVVLKHMNFEVQLEVRYSRARKEVEAFEPLNFRHVFQIKMKI

>PintIR9

MPKMKTRYNSSEDTLKMKISPLNWFATYLVGIIVSKKKLMIELFTIGVRLSALLRLPSKYFYHDLLFLVDLRCHGSEQVLLNATLHKLFVWPFRWLIIADPVTSQSMIWELPAMPDSDVVLAENRGEGFTLTELHKPAANYSMISTPKGFYNGTFVDVRPYRQMFRRRRDLMGHALTISNVIQDSNTTKYHMLQENRLELQYDAVAKICFISARHAFEMLNVTTRYIFSYRFGYKVNGKWTGMIHDLQENKADLGTNCVVFLNRLEVAVYTDTVAPMRMRFVFRQPPLSYVSNIFALPFSTNVWLAIIICSLACTATLYCTSNWEVQIERASTQLNGSVGDALLLTLSAITQQGCAIEPRRAPGRIMEWVFFAAVMALYAAYSANIVVLLQAPSNSIKTLAQLANSKLELGANDLDYNRFVLSVILFFACRQNIRCSWPQKRERVRERGTKIVVN

>PintIR10

RPWMPYTKMVFVKFALIFLLIGEGSAKVNPHGPTVVNDLASCVTNVIEINFKRPGILVFANTNNFSTSVSRIRSQLLSYLHEDIKYSIEITSPDGQETICGENNEYDIGVLHRDHFEPVIEADYYVLIIDDYKDFTGLASKIIRSRRWNSRAKFIVLLLNFARIQTNVNYVERIITCLYNFYVLDIIIILPHESNIRNALIYGWRAYEPPKYCGYFNETAKDRSILINTCEKGRLKNNVSVFPDQIPKSMKGCILHILALKREPFVSGNENEFNMEEYLVNEVFKTFHFTVEYDVIHSFRGERYDGEWNGALKLLSNKRGHILLGGIFPDFDVHEDFESSTFYLSDSYTWVVPRAPASPRWVALFIIFSNFVWLCVCITFLICVMSWIFLGFLSRDTSYNRYFGHCFLNSWACTLGFCSYIRPKKESLRLFLCVF

>PintIR11

MLNLIYKIKMANEDWHVEYYGNWEASGGLIKSMQMEVTTAMRRRNLERNVITTSLIAVDNRTKENLYDLRNVEIDVTTKANIRHIDVLYDFINATKAIKFTNTWGYFINGSWNGIVGHFVRGETDIGGTVMFINLERIRLLKFICHPTNIVVEFVFREPPLSYHSNLFLLPFTRTVWMCIAAFVLIFLFITYLNVYWESIKIDKEIMKEAPNHPALRANVSDIVVLLISAMCQQGSSIELKGGVGRLIMFLIFLSFLFLYTSYSASIVALLQSSSSHIRTLADLYNSGMELGIENLPYNIYYFTSAKDPLRKAIYQNRVAAKGRKPNILSIEEGVKRVQTVSTYLSGILM

>PintIR12

CVLGCAVFLYFTSRWEATVSMNQFQLDGSSADVLILIIGAVLQQGCTLEPRYAAGRTVTLLLFIALTILYAAYSANIVVLLRAPSSSVRTLPDLLNSPIKLGASDFEYNRYFFKQLNDPIRKAIYDKKIAPQGKKPNFYNMKEGVEKIRKGLFAFHMESNPGYRLIQETYHEDEKCDLVEIDYINEIEPWVPGQKRSPFRDLFKINFIKIRESGIQANIHQRLNVPRPKCSGAVAAFSSVGIADMYPALLATLYGVLLAPAVLILEITYHRLMVIRKKRAIT

>PintIR13

MTSMARRLATSGTNIAVSLQNNKSEYTFHHVIILVDFACPGVDTFLKQASDRGFFKSPYRWLLLNLNHDEESILDELEFVVDSDVVIARKFGEQIVLTEAYKVSKNTEIIYTTRAKWYPSNTNKHLLEDSEGIIKKSTNDSDTTENSDFIKMIPNAMTLSKFGVIEDYRRSKVLSYRRKDLRKHTLTMVNVITDTNETRKHMDDRLFLHQDGIAKMSY

>PintIR14

MRADYLATDTCDFALSGDEFLDEQVAMIMPAASPYLSVINKEINRMQKAGLITKWLSAYLPKRDRCWKTSSITQEVNNHTVNLRDMQGSFFVLFLGFFSATTVLLIEFFYNRRKRKNENVVIKPYVE

>BmorIR87a

MTTGNSDQIAKTAECVLKLSAKYFVERKALSGSIVIINVNSYSSTTQGLLLKTIHSSIKYSVMAKDSFYPHANASHFPEKAKNYMLILEERTELKRNIFQLNKLPSWNPLAKAVVFYQIKGNESAQRIAIEFINELREHKFFRSIIFINNGTESGVTSYTWRPYSENNCGGKCDSVYVLDRCKNNIVEQIEPQPEWFPSNMNGCPLTTYAIVSEPYVMPPIRKIPNAKFDDVYEFQKGGETNLVKTIAEFSNMTLIVRLSAIEENWGIIYANGTATGAYGVLRNDSVDIVFGNIEVTKQIRKWFHPTISYTQDEITWCLPKAGQASAWDNLVIIFQWTIWVATFTSLILMGLLFHYMYYREKNKKITKWPTNSLLMTFSMLLGWGSHFEPKTATFRILIFGWLCFSINMGISYESFLRSFLMHPRFEKQIATESDLIQSGIRFGGREIYRTYFESNDASSSYLHTEYSSTTFSEGIRRAALNRDFAVVSSRRQAEYQDQKLGKGASLIYCFPESDNLYKYSVVLLARKWFPMLERFNGIIRSVSENGLINKWNDEMFIHRVSLEGASTIVPLSIQHLLGAFMFIGFMYGTSAFIFLVEVFVGFVQRRAFLSAFFCGKKKRFSAVFKVKV

>BmorIR7d.3

MRTEPEDITLFLQHFHGSAVIVPLDYQNMKAVSELNKATGFKQTVLFAVSVEEFILFITTLNLDLIVPIRMVLVLTTQLTDLAMITKEAWKHDLAEIIIISKDENEEIRLTTYFPYKNGICGDYTPHSISNEKELFPEKFKNLHGCPIKVTLLNFLPYVGLQKVNGTITFIFGIDGSVFILLIKELNAIMDIVSSTDHGGMGVFVNGSWKGSFGDIVRREADIFAPAGIITQKRFSVAQMSHTYETLNIHWCAPPRREIYAWAKVLLPFLTNITPFLVLAFTVFVITIVLVKRSKLHGIKSNKNVFLQSFMIFLGQGVKFETKSSVINSFFVAWLWFCLIVRIAYQGDLVNGLQKKIYEPPFESVEQALQELDGYGGTELFREYYAGSPIADNYQVIKIGDLPRYIRDVIAGKRFLIATDILMHQYAKKFQILQEPLTHSPTCLFMRPGWPVSRRVDVIIIRAIEAGLVQKIIYDFHYTVRLRRHEKEEETGTRPLGMSTMFACYYGLILLWIFSFVIFLFEVLYYNWKHKIAYIKRKRNKLFKFHH

>BmorIR7d.1

FNLPLKLTILSLFLGIILLNMLRKTIFFNNIRRVCNITPPKRNSLFYAWLLFLGLPLEKFSSRKHFKIIILAWIWFSFVIRCAYQVTLVTSLKSITYNYNLRYDSDILKYPFGGMSSIRDYFIEDKDFYENWTSVDMQKAYKLLDEIMEEKTDFVLALNKDTILHHAAEHIGSKRIQVIDNCIVNSPIVLYFRKHSPMTDPIAKIMNAALECGFIQYSYQTNWKRQKHLLNSHYAYNLQPLTLDNFSGCFFLLIIGYGISILYFVLEVVCHKIDKTNQRIDLRVDQE

>BmorIR7d.2

MSPRNLSDASHFCEENSNEITTAALNIALHNFKWRILTYVFFNATFLCNLNIFLKTYNKGVVVGNGLVEPRIDGKIQQLVLFCDDIVGITLALNSLPNQFDETGKVIVICQSPISWKCSAEEAMRSFWSVKITNVVFLKKDVFVMAYTYMPVYNEQCEISDPIPLFGLKPCIINATKCGVFDKKLDNLNKCKIVVSTLIRRPFMIINNGIPEGADGDLLLLIMERLNATLEVIIPGDHNYWGKLDSNGTWSGSLGDVYYGAADISMTSAALTASIISYFKISIPYRSTNVVWISHPPKALSPALKLLHPFKPSTQIALGIIFFIVIACVLFVSSKKMWLLCCRRVRPTKKKPSLLFNTWMICIGVPIAHLPSTSTFLSLIVLWIWYCFLIRTFYQVWLINSLQGKFYLDGFEKIDEAIEAGYDIGGGIFLKEYFVDYPYIYNNWKETVSLNVTLHEISEGSNFIAATIYDLAKSLTNFEKINVHFLAEKVVVSPSVLFFNKNSPLVAPINELLQQLTESGFVEKISRNYFTHNVTNWKRQKHLLNSHYAY

>BmorIR41a

WINCTVQGVIGSVVEGRSDFGIAALYSWYEEWKAMDFSVSVVRSAVICLVPAPRVWELPFLPFKSIWIAVVITFVYASIGLTIAQ--GCKLLIVFGTIISQSQYIVSDSWRIRSVIGWLLVSSLILVSAYGAGLASTFTVPPSIDTVQDLLNSRMEWGFEELQRRSAFSLEQDMQMFYFDCVAMLHKNSPYTEKLSELIGRLHQSGLLWESQVSLNFNHVEGIFLIFITGTILSTLFFALE

>BmorIR68a

KMNFRPKGLLGEMVNGRADLALGNLQYTPYHLELIDLSIPYTSQCWTFLTPEALTWKTLLLPFLYMWIAVLLVLIT-GTIFYGLARYFGNILYTYGMLLVVSLPKLPTGWSIRFLTGWYWLYCILLVVSYRASMTAILANPVTIDTLVELAASKLTCGPNIAADKVAYYDNRNLHMVVNIISIGFHKNSPLKPLTDIYITRIVEVGLVWLNDAMMNLKKLYGAFIALAIGYFLSVMCLIGE

>BmorIR21a

KNNFSIEAVAKEIAKGRADIGVAGMYLTIDRTREMDVTFAHSQDCAVFITLMSTAYQAILGPFWHVWVALTLTYLFGMFPLAFSDKHSGNFWYVFGTFTGRNSWSKTDKITTRLLIEMVLDFTIIITSCYTGSIIAFVTLPETVDTIHQLLAGFYRVGVEAGIMNTAFLGSKVLHSFVPFVTIGFPNNSLYTAKLNNDLRRMVQSGIVIVDEVRLTLEDTQGMFLLLAAGFLIAATALISE

>BmorIR64a

MYNITYDGMVGSLQRHEADVGGSPIFFKTDRAYVVDYVAETWPSKQSFIFRHPKHHTVYSRPLNSVWYCVIAFLFVTASTVFFMLKFETLFLFAWSAICQQGMSLRRNSLALKVVVFVTFVCSITLYQYYNATVVSTLLKEITIRTLKDLLQSDLKVGPEYGMSLVAFHVDCEIHVYPPQMGAVLKKNSPYRNYFAIGIRRLWETGLMMKHIWDVSILEFSTPLFIVVFGVIASVVVLLCE

>BmorIR75d

DLRFRFNGTVGLLQRGRAELGVASMFMRSDRWRVLHFSSATVALLNAFMLRAPAQSNIFLLPLRGVWCCAAALLCGSAVLLAVLSCLLLEFVFSIGTVCQQGFYIMPKLSSIRMIMFLTLLTSLFTFTAYSAKIVAILQTPAAVRTVADLADSHMDVGVVEGVERMAFQVECGLMIFKLPVAVPLRKHSGYRELFGTRLRWQREVGLMVRAIWLVRLLDMLPALQMLAAGGLVAVVLLILE

>BmorIR75p

MLNATPRGMINDLHTSKADLGTNCVVSDVERLSVVTYTDMLAPFRVRFVFRQPPLANIFYLPFGRVWAAVAVCAMVYTAAIYWASKWDGDMLLTMSALSQQGCFIEPKRAPGRIMLFVLFTALMALYAAYSANIVVLLQAPNSITSLAQLAASKVTLALEDGVDMIAFHSICDLTVLSSFPFVPVKKDSPYLELLRVSFKQIRESGIQLNRRYQVGIVDLRPVLIMMIYGIISSCLILIME

>BmorIR75q1

FMNASHKGMMGDLAKGTVDFGGTIAFLTSQRLQVVDYLSSPVPINAKFVFREPPLNNLFLLPYANVWYCTAAFVVLLVIILYINAKWQPDTILVISAISQQGSSNELKGTLGRAVLFLLFLTFLFLYISYSANIVALLQSNKQIRTLQDLLNSNLNIGIEEGVKKLAFNMNCGLQIIESSPWMSCRKNSPFREIYKLGLFKLQEHGITENRLLFVNMVDVYPVILMFLYGLFLAFLILLVE

>BmorIR75q2

FLNAERKGLTGFLVNGDVEIGGSPMFFTAERTAVVDFISSPTPTRSKFVFQQPKLNNLFLLSFTAVWYSTLALISLIFTMLLSVTAWRPDTMLVFGATCQQGSTVELKGSLGRVVMLILFLTLMFLYTSYSANIVALLQSSSQIKTLEDLLHSRLKFGMEEGVKKMAFHMECGLKILQVIPWLAVRKNTPYKEMFKIGMKRIQEHGLQENRLLYVSMVDCYPALLVLSYGIIIAIALVIME

>BmorIR40a

KLNFRYRGTLGLIWKRQADFFLGDVTMTWERLQAVEFSFLTLADSGAFLTHAPAKTLAIIRPFWEVWPLVCATLFITGPALWIVIAAMGNCWFTVTLFLRQSSTKPSSTHKARLVTVLISLATYVIGDMYSANLTSLLARPPPIGTLPALEEAMREHGVEAGVRLVAVLGGHNFHLLYTRSAIAFQIGSPYLETINNVVMTLFEAGILMTTDEYVSLTMLRGAFCLLGIGHLLAGVTLLIE

>BmorIR76b

KFNFTYESLIGLTNTSKVDMIAAFIPRLVRFRKLVTFSRDLDEGVWMMMLRRPKEGSGLLAPFNFVWYVTLASVLCYGPCICFLTHVPLPFWFVYSAFIKQSTNLAPEANTTRVLFATWWLFIILLSAFYTANLTAFLTLSLDIETPEDLYKKNYRWVPDQEYLPIAVLVKCTYVAFMKKRAFVYPVGSKLKSLFDPTLAYILQSGIILEHKDLLTNSHLMMTYYIMCVGLASGLAVFVVE

>BmorIR93a

NKNFTIRYNNIPLYFRAVFIHQAGVNLKNNYYRCINYTIPVSTQPHTFIVARPREALLFLLPFTDTWLCLGFAVILMGPMLYIVHRLLANLWYIYGALLQQGGMYLPRADSGRLVIGTWWLVVLVIVTTYSGNLVAFLTFPAPVTTISELLKNSYTWSARGTLDRVLIFDWCDFAAFMEEVAMIVPAGSPYLPVINKEINRMHKAGLIWLSAYLVNLSDMQGSFFVLFLGNDKIVYMYIAE

>BmorIR8a

AMNFDYEGVVGDLTTGETDIAVAALTMTAEREEVIDFVAPYFEQGILIAIRKPIRLFKFMTVLTEVWLSIVAALVLTGFMIWLLEKYDFEFWFALTSFTPQGGGEAPKALSGRTLVAAYWLFVVLMLATFTANLAAFLTVETPVSSLEQLARQSINYTAETGFKQVAFIHDCNLTVFAEQYAIAVQQGSRLQEDISRALLELQKERFLLTSKYWITLESLGGVFIATLFGLGLAMITLAWE

>BmorIR25a

IVKFDYEGIIKELIEKRADIALTSLSVMAERENVVDFTVPYYDVGITIMMKLPRTLFKFLTVLNDVWLSILAAYFFTSFLMWVFDKWEFELWFCMTSLTPQGGGEAPKNLSGRLLAATWWLFGFIIIASYTANLAAFLTVSTPIESLDDLSKQYIQYAIEEAVQRVAWLGDCDLQVFSRKYAIAVQQGSPLKDQFNNAILQLLNRRRLLKENWWISIQNIGGVFIVIFMGIGLACITLGVE
